
JOURNAL INFORMATION
==============================
NLM Title Abbreviation: Behav Neurol
Journal ID: Behav Neurol
NLM Journal ID: 8914585
Journal ID: BN

Behavioural Neurology

ISSN: 0953-4180
EISSN: 1875-8584
Publisher: Wiley

ARTICLE INFORMATION
==============================
PMCID: PMC5215690
PMID: 28149002
DOI: 10.3233/BEN-139900
Article ID: 976782
Article version: 1
Subjects: Other

Joint Meeting of the FESN (Federation of the European Societies of Neuropsychology)/GNP (Gesellschaft für Neuropsychologie) September 12–14, 2013, Berlin, Germany

Print publication date: 2013
Volume: 27
Issue: 3
First page: 297
Last page: 461
Received 2013 Aug 6; Accepted 2013 Aug 6
Copyright: Copyright © 2013 Hindawi Publishing Corporation and the authors.
Copyright year: 2013
License: This is an open access article distributed under the Creative Commons Attribution License, which permits unrestricted use, distribution, and reproduction in any medium, provided the original work is properly cited.
License URL: https://creativecommons.org/licenses/by/3.0/

==============================
Abstracts from the

### Joint Meeting of the FESN (Federation of the European Societies of Neuropsychology)/GNP (Gesellschaft für Neuropsychologie)
### September 12–14, 2013
### Berlin, Germany

4th Meeting of the FESN
28th Meeting of the GNP

Conference president
Michael Niedeggen

Behavioural Neurology 27 (2013) 297–461
DOI 10.3233/BEN-139900
IOS Press

### Symposia

### Keynote 1: Functional neuro-anatomy of spatial neglect

Patrik Vuilleumier[1,2,3]
[1]*Laboratory for Neurology and Imaging of Cognition, Department of Neurology and Department of Neuroscience, University Medical School and Hospital of Geneva*
[2]*Center for Neuroscience, University of Geneva*
[3]*Swiss Center for Affective Sciences, University of Geneva*

Spatial neglect is generally defined by various deficits in processing information from one (e.g. left) side of space contralateral to focal (e.g. right) hemisphere damage. The talk will review evidence that the clinical syndrome of neglectis likely to reflect a combination of disorders that may co-occur due to concomitant damage affecting juxtaposed brain areas and their connections, and that such lesions may induce functional disturbances in structurally intact areas through losses of top-down control from fronto-parietal cortical areas. Improved knowledge in these functional anomalies at the network levelalso points to new approaches for rehabilitation strategies. In particular, I will illustrate how theattentional deficits associated with neglect can at least partly be alleviated by manipulations influencing perceptual processing throughpathways outside the damagedfronto-parietal network, including emotional conditioning or reward learning, or by using methods that may help restore top-downattention signalssuchprism adaptation or neurofeedback.

Correspondence address: Professor PatrikVuilleumier, Labnic/Neufo, CMU, Michel Servet 1, University of Geneva, 1205 Geneva, Switzerland. E-mail: patrik.vuilleumier@unige.ch

### Keynote 2: Modulating the neural mechanisms of visuo-spatial attention

Gereon Fink
*Department of Neurology, University Hospital Cologne, Cologne, Germany*

### Keynote 3: Cognitive rehabilitation for people with early-stage dementia

Linda Clare
*School of Psychology, Bangor University, UK*

People in the early stages of Alzheimer's disease (AD) face the challenge of adjusting to living with progressive cognitive impairment. Methods and techniques from neuropsychological rehabilitation can be applied to assist in managing the impact of memory difficulties. This talk describes the initial application of neuropsychological rehabilitation methods for people with AD and the development of an individually-tailored, client-centred cognitive rehabilitation (CR) approach. CR involves identifying meaningful goals relating to areas of everyday activity where the person with AD experiences difficulty due to changes in memory and other cognitive abilities, and wishes to see improvements. The person with AD and carer work with a therapist over a number of sessions to address these goals, for example by improving the use of memory aids and strategies, relearning skills, resuming previously-enjoyed activities or developing new activities. The sessions take place in participants' homes and involvement of a carer helps to ensure that the benefits are carried over into daily life. Evidence from the first randomised controlled trial of CR will be presented. Participants with early-stage AD were able to identify personally-relevant goals and to rate their performance in these areas. Goals reflected a range of domains, with memory-related goals featuring most prominently. Participants who received the CR intervention significantly improved their self-reported goal performance

ISSN 0953-4180/13/$27.50 © 2013 – IOS Press and the authors. All rights reserved
This article is published online with Open Access and distributed under the terms of the Creative Commons Attribution Non-Commercial License.

and satisfaction with performance following treatment, compared to participants in control conditions. CR also had beneficial effects on cognition, mood and quality of life for participants with AD, and led to reduced stress and better quality of life for carers. These findings indicate that individual, goal-oriented neuropsychological rehabilitation is feasible in early-stage AD and provide preliminary support for the clinical efficacy of CR. A larger-scale, definitive trial is now in progress.

**Keynote 4: Neuropsychological predictors of conversion from Mild Cognitive Impairment to Alzheimer's Disease**

Guido Gainotti
*Center for Neuropsychological Research of the Catholic University of Rome, Italy*

The MCI construct has been proposed by Petersen et al. (1999) to identify patients at risk of developing a dementia of the Alzheimer's type (DAT). A simple set of criteria has been proposed for the identification of MCI patients, but only a limited part of patients defined as MCI on the basis of these criteria evolve to a DAT, whereas many more patients remain stable and some revert to a normal status. Two solutions have been proposed to solve this unsatisfactory situation. The first has consisted in distinguishing 4 different forms of MCI: (a) amnestic single domain; (b) amnestic multiple domain; (c) non-amnestic single domain and (d) non-amnestic multiple domain. This distinction has allowed to show that the highest percentage of conversions to DAT is observed in amnestic multiple domain MCI patients, whereas non-amnestic MCI patients tend to convert to other forms of dementia. The second solution has consisted in substituting the construct of MCI (based on clinical and neuropsychological criteria) with the construct of 'Prodromal Alzheimer's disease' based on the integration of these criteria with other neurogenetic, neuroimaging and CSF biomarkers, that take into account both a specific pattern of cognitive changes and structural/biological evidence of Alzheimer's pathology. Since these pathophysiological criteria are more useful for 'research' than for 'clinical' purposes, we think that the predictive value of the amnestic MCI construct could be increased with a more strict definition of the memory tests (in particular of delayed recall measures) and of the cut-off points most appropriate to identify patients who will probably progress to AD. Furthermore, since long-term longitudinal studies have shown that mild cognitive disorders can be observed even before the MCI stage, it could be useful to characterize the nature of these pre-clinical subtle cognitive disorders, to identify the targets of possible disease-modifying pharmacological trials.

**Symposium 1: Advances in the neuropsychological assessment of cognitive impairment in the elderly**

Andreas Monsch, Michael Ehrensperger, Kirsten Taylor, Panagiota Mistridis and Irene Beck
*Memory Clinic, Dept. of Geriatrics, Basel, Switzerland*

Neuropsychological assessments play a critical role in early detection and diagnosis of cognitive impairments in the elderly. The first talk will present a new screening tool for cognitive impairment (ie, BrainCheck) as an iPhone/iPad app to be used by health care professionals. Alzheimer's disease neurofibrillary pathology begins in perirhinal cortex before entering the entorhinal cortex and hippocampus. Thus, functional and structural measures of perirhinal cortex functioning may identify AD patients earlier than corresponding measures of the entorhinal cortex and hippocampus. The second talk will describe the results of innovative cognitive neuroscience research, which has tested this hypothesis. The third talk will address the question whether dissociable MTL regions (i.e. hippocampus and perirhinal cortex) support recall and recognition performance, respectively (dual process models), and whether the left and right prefrontal cortices differentially support learning and memory performance, respectively (HERA model) by correlating behavioral measures of verbal episodic learning and memory performance with VBM measures of regional whole brain gray matter integrity and a priori defined anatomical regions of interest in patients with different forms of dementia. The fourths talk will present the results of analyses to determine the base rates for the German Consortium to Establish a Registry for Alzheimer's Disease – Neuropsychological Test Battery (CERAD-NTB) – one of the most widely used dementia assessment instruments in the United States and in German-speaking Europe – in the norming sample using different cut-off scores.

Correspondence address: Prof. Dr. Andreas Monsch, Memory Clinic, Department of Geriatrics, Schanzenstrasse 55, CH-4031 Basel, Switzerland. Tel.: +41 612653193; Fax: +41 612653794; E-mail: Andreas.Monsch@unibas.ch

### Lecture 1: BrainCheck: A new screening tool for cognitive impairment in the elderly – combining patient assessment with caregiver information

Michael Ehrensperger, Andreas Monsch and Stefan Bläsi
*Memory Clinic, Dept. of Geriatrics, Basel, Switzerland*

Cognitive disorders are frequently underdiagnosed. General practitioners (GPs) are in need of an effective screening instrument for cognitive change, which would help them GPs decide whether patients should be evaluated more extensively or 'watchful waiting' is indicated. The current study developed a new, quick and easy-to-administer screening tool, BrainCheck, which combines patient and informant assessments, and detects early cognitive decline associated with MCI and early stages of dementia. Patients with early Alzheimer's disease (AD) ($N = 185$), MCI ($N = 80$), and cognitive impairment related to major depression ($N = 23$) were recruited from six memory clinics in Switzerland (54% w, age = 76.6 ± 7.9 y, education = 12.0 ± 2.6 y). These patients and cognitively healthy individuals (NC; $N = 126$, 40% w, age 75.2 ± 8.8 y, education = 12.5 ± 2.7 y) completed a patient-directed tool ('MEMENTool') consisting of three self-reported questions about the participant's memory and the Clock Drawing Test. Correct classification rates (CCRs) of the MEMENTool were calculated from this sample. Additionally, a subsample of 113 patients with MCI or early AD (53% w, age = 77.3 ± 7.2 y, education = 11.7 ± 2.5 y) and 70 NC subjects (29% w, age = 77.2 ± 8.9 y, education = 12.5 ± 2.9 y) were administered the very short Informant Questionnaire on Cognitive Decline in the Elderly (IQCODE, 7 item version). BrainCheck CCRs were calculated combining MEMENTool and IQCODE data. The patient-directed MEMENTool demonstrated a sensitivity of 83.3%, a specificity of 79.4%, and a CCR of 81.3%. In the NC, MCI and AD subsample, the BrainCheck (ie, combined MEMENTool (subsample CCR = 79.9%) and IQCODE (subsample CCR = 78.6%)) achieved a CCR of 89.4%.
BrainCheck, a screening tool combining patient and informant information, can be administered within a few minutes and achieved a CCR of 90% accuracy.

Correspondence address: Dr. Michael Ehrensperger, Memory Clinic, Dept. of Geriatrics, Schanzenstrasse 55, CH-4031 Basel, Switzerland. Tel.: +41 612653731; Fax: +41 612653794; E-mail: Michael.Ehrensperger@usb.ch

### Lecture 2: Perirhinal cortex functioning

Kirsten Taylor and Andreas Monsch
*Memory Clinic, Dept. of Geriatrics, Basel, Switzerland*

Alzheimer's disease (AD) neurofibrillary pathology begins in perirhinal cortex (PRc) before entering the entorhinal cortex and hippocampus. Thus, functional and structural measures of perirhinal cortex functioning may identify AD patients earlier than corresponding measures of the entorhinal cortex and hippocampus. This talk describes our cognitive neuroscience research which has tested this hypothesis.

Correspondence address: Dr. Kirsten Taylor, Memory Clinic, Dept. of Geriatrics, Schanzenstrasse 55, CH-4031 Basel, Switzerland. Tel.: +41 612658942; Fax: +41 612653794; E-mail: Kirsten.Taylor@usb.ch

### Lecture 3: Functional neuroanatomy of verbal episodic learning and memory impairments in dementia

Andreas Monsch, Irene Beck and Kirsten Taylor
*Memory Clinic, Dept. of Geriatrics, Basel, Switzerland*

Episodic memory impairments are a cardinal feature of the majority of dementia syndromes. Many voxel-based morphometry (VBM) studies have shown that dementia patients' verbal episodic learning and memory performance is significantly associated with medial temporal lobe (MTL) atrophy. However, controversies exist as to whether dissociable MTL regions (i.e. hippocampus and perirhinal cortex) support recall and recognition performance, respectively (dual process models), and whether the left and right prefrontal cortices (PFC) differentially support learning and memory performance, respectively (HERA model). The present study aimed to address these issues by correlating behavioral measures of verbal episodic learning and memory performance with VBM measures of regional whole brain gray matter integrity and a priori defined anatomical regions of interest (ROI) in patients with different forms of dementia. The whole-brain analyses demonstrated that the entire MTL system is associated with all aspects of verbal episodic learning and memory performance, consistent with the declarative memory model. ROI analyses further demonstrated that the integrity of the hippocampus and perirhinal cortex were significantly related to recall and recognition performance, respectively, as hypothesized by dual process models. Finally, the distributed network underpinning recognition memory performance appeared to extend to a disproportionate reliance on the right over the left PFC, in partial support of the HERA model. These brain-behavior relationships allow inferences to be made about the sites of brain atrophy, and thus the potential cause of dementia, based on the pattern of an individual patient's episodic learning and memory performance.

Correspondence address: Prof. Dr. Andreas Monsch, Memory Clinic, Dept. of Geriatrics, Schanzenstrasse 55, CH-4031 Basel, Switzerland. Tel.: +41 612653193; Fax: +41 612653794; E-mail: Andreas.Monsch@unibas.ch

### Lecture 4: Base Rates for the Consortium to Establish a Registry for Alzheimer's Disease, Neuropsychological Test Battery (CERAD-NTB)

Panagiota Mistridis[1], Andreas Monsch[1], Michael Ehrensperger[1] and Grant Iverson[2]
[1]*Memory Clinic, Dept. of Geriatrics, Basel, Switzerland*
[2]*University of British Columbia, faculty of Medicine, Dept. of Psychiatry, Vancouver, Canada*

Early and accurate diagnosis of mild cognitive impairment (MCI) and dementia can be achieved with comprehensive neuropsychological test batteries. The Consortium to Establish a Registry for Alzheimer's Disease – Neuropsychological Test Battery (CERAD-NTB) is one of the most widely used instruments in the United States and in German-speaking Europe. The CERAD-NTB consists of five subtests yields ten demographically-adjusted standard scores. The German version has been normed with over 1,000 healthy elderly individuals. The aim of the current study was to determine the base rates for the German CERAD-NTB subtests in this norming sample. The prevalence rates of 'pathologic' scores on the German CERAD-NTB subtests defined by using a range of different cut-off scores (i.e., −0.67 (25%ile), −0.84 (20%ile), −1.00 (16%ile), −1.13 (13%ile), −1.28 (10%ile), −1.48 (7%ile), −1.64 (5%ile), −1.96 (2.5%ile), −2.32 (1%ile)) were calculated from the normative sample ($n = 1{,}081$; 407 women, 674 men; age: 68.6 ± 7.83 (range: 49–92); years of education 12.51 ± 3.0 (range 7–20)). Relatively high percentages of one or more 'pathologic' scores in the normative sample were found. For example, when a cut-off score of −1.28 (i.e. the 10%ile) was used, 60.6% of healthy individuals obtained ⩾ 1/10 'pathologic' scores. These findings illustrate the importance of considering the prevalence of low ('pathologic') scores in normal individuals.

Correspondence address: M.Sc. Panagiota Mistridis, Memory Clinic, Dept. of Geriatrics, Schanzenstrasse 55, CH-4031 Basel, Switzerland. Tel.: +41 612653193; Fax: +41 612653794; E-mail: panagiota.mistridis@usb.chch

### Symposium 2: Social cognition deficits and behaviour in brain damaged patients

Maarten Milders
*Heriot-Watt University, School of Life Sciences, Edinburgh, United Kingdom*

Deficits in social cognition have been observed in patients with brain damage due to various etiologies. Social cognition refers to those cognitive functions involved in understanding other people (e.g. their intentions or emotion) and the self. An important possibility is that deficits in social cognition, unlike deficits in standard cognitive functions, could underlie and explain changes in social behaviour following brain damage. Such behavioural changes are linked to poor psychosocial outcome.

This symposium will bring together research in different populations of brain damaged patients that attempts to relate deficits in social cognition to patients' social behaviour. Williams et al. will show that alexithymia is associated with aggressive behaviour in patients with traumatic brain injury (TBI). Patients who were less able to identify and describe their feelings had stronger aggressive tendencies. Allain will report impairments in the ability to understand other people's intentions and feelings in patients with Huntington's disease (HD). These impairments could be the basis for the behavioural changes seen in patients with HD. Spikman et al. will present one of the first reports of an association between emotion recognition in patients with TBI and post-injury behaviour, as rated by a significant other of the patient. This finding is in line with suggestions that social cognition deficits underlie changes in behaviour following brain injury. Milders will continue on this theme and shows, in a different sample of TBI patients, that the correlation between emotion recognition and behaviour is selective, i.e. is not explained by injury severity or other cognitive deficits. Finally, Yeates will discuss current approaches in the rehabilitation of social cognition deficits in patients with acquired brain injuries with the view to prevent or reverse maladaptive social behaviour and negative psychosocial outcome.

Correspondence address: Dr. Maarten Milders, Heriot-Watt University, School of Life Sciences, Riccarton, EH14 4AS Edinburgh, United Kingdom. E-mail: m.milders@hw.ac.uk

### Lecture 1: The relationship between alexithymia and aggression following Traumatic Brain Injury

Williams Claire, Rodger Woods and Mandy Saville
*Swansea University, Department of Psychology, Swansea, United Kingdom*

Aggression is a frequent legacy following TBI which can have an adverse impact on interpersonal relationships and psychosocial recovery. The nature and severity of aggression has been linked to a variety of factors, but very little research has examined the relationship between aggression and deficits of social cognition. To this end, the aim of the study was to examine the relationship between alexithymia and aggression following TBI. A total of 39 individuals with TBI and 60 demographically matched controls completed the 20-Item Toronto Alexithymia Scale (TAS-20) and The Aggression Questionnaire (TAQ). In the TBI group, significant other ratings on the TAQ were also obtained. Results revealed significantly higher alexithymia scores in the TBI than control group. TAQ ratings were also higher in TBI group, with 'Anger' subscale scores differing significantly across the TBI and control groups. Significant positive correlations were found between TAS-20 and TAQ total scores in both groups, with TAS-20 total scores explaining a significant amount of variance in TAQ scores. However, the relationship between alexithymia and aggression was strongest in the TBI group. To conclude, the results of this study found evidence to suggest that the presence of alexithymia is an important factor affecting aggression following TBI. Therefore, rehabilitation workers need to remain alert to the possibility that individuals with TBI may exhibit some form of emotion deficit disorder that can have an adverse impact on behaviour, which in turn, may increase the risk of relationship failure, social isolation and poor psychosocial outcomes.

Correspondence address: Dr. Williams Claire, Swansea University, Department of Psychology, Swansea, SA2 8PP Swansea, United Kingdom. E-mail: Claire.Williams@swansea.ac.uk

### Lecture 2: Huntington's disease is associated with deficits in cognitive and affective theory of mind

Philippe Allain
*Université d'Angers – Laboratoire de Psychologie des Pays de Loire, Angers, France*

Huntington's disease (HD) is a neurodegenerative disorder, with predominant involvement of the striatum. This condition gives rise to altered social conduct and breakdown in interpersonal relationships, although the factors underlying these changes remain poorly defined. This study investigated whether individuals with HD exhibit impairments in the understanding of other people's cognitive and affective mental states, two main aspects of Theory of Mind (ToM). Eighteen HD patients, early in the course of the disease, and eighteen healthy volunteers matched by age and educational levels were given a non verbal cognitive ToM task assessing attribution of intentions to others and a revised version of the 'Reading the Mind in the Eyes' test, which is an affective ToM task assessing the understanding of other people's feelings from their eyes. To analyze relationships between executive functions and ToM, participants also completed seven executive measures tapping spontaneous and reactive flexibility, inhibition, set shifting, executive planning, rule detection and working memory. The two measures of ToM were indicative of a significant impairment of ToM abilities in patients with HD patients. HD patients also exhibited executive dysfunction, but there was reduced evidence that executive impairments were related to cognitive and affective ToM deficits. Our results are consistent with the idea that both cognitive and affective aspects of ToM are impaired in HD patients, indicating that cortico-subcortical circuits participate to the mediation of higher social functions such as ToM. Our results are also consistent with the idea that ToM difficulties appear relatively independent of executive processes dysfunction. They also provide a basis for the understanding of disorganised behaviour and breakdown in interpersonal relationships in daily life in HD.

Correspondence address: Dr Philippe Allain, Université d'Angers, Laboratoire de Psychologie des Pays de Loire, Angers, 49045 Cedex 01 Angers, France. E-mail: PhAllain@chu-angers.fr

### Lecture 3: Impaired emotion recognition in TBI in relation to behavioral changes and impaired self-awareness

Joke Spikman[1], Maarten Milders[2] and Annemarie Visser-Keizer[3]
[1]*University Medical Center Groningen, Department of Clinical Neuropsychology, Groningen, Netherlands*
[2]*Heriot-Watt University, School of Life Sciences, Edinburgh, United Kingdom*
[3]*Groningen University, Department of Clinical and Developmental NeuropsychologySciences, Groningen, Netherlands*

OBJECTIVE: After moderate to severe TBI behavioral changes, involving inadequate social-emotional behavior, are common. It has been put forward that deficits in social cognition, of which the ability to recognize facial affect is an important element, might be an underlying mechanism. In a range of studies deficits in emotion recognition were demonstrated in TBI patients, but to date, these have not been related to behavioral deficits. Measurement of behavioral changes can be complicated because another frequent consequence of TBI is impaired self-awareness, causing patients to provide an unrealistically positive report of their own functioning. We investigated whether impaired emotion recognition after TBI was related to behavioral changes and impaired self-awareness.
METHOD: 51 patients with moderate to severe TBI and a group of 31 healthy controls were assessed with the FEEST (Facial Expressions of Emotion-Stimuli and Tests) testing perception of emotional expressions on faces, as well as with the DEX (Dysexecutive Questionnaire) measuring behavioral problems. For all participants, there was also a proxy rated version of the DEX. The difference score between DEX-Self and DEX-proxy was used as indication of self-awareness.
RESULTS: Patients performed worse on the FEEST total score as well as on the Anger, Disgust, Fear and Sadness subscores than healthy controls. They also had higher scores on the DEX self and proxy version, and a higher DEX-difference score than the controls. Correlation analyses showed that there were no significant correlations of the FEEST scores with the DEX self rating. However, there were significant correlations between the FEEST total score and the DEX proxy and DEX difference scores. In particular, the perception of Sadness was significantly related to these latter DEX scores.
CONCLUSION: Deficits in emotion recognition after TBI are related to behavioral problems, rated by significant others, as well as to impaired self-awareness.

Correspondence address: Dr. Joke Spikman, University Medical Center Groningen, Department of Clinical Neuropsychology, Hanzeplein, 9700 RB Groningen, Netherlands. E-mail: j.m.spikman@rug.nl

### Lecture 4: Social cognition neuro-rehabilitation strategies: An overview

Giles Yeates
*Buckinghamshire NHS PCT, Community Head Injury Service, Aylesbury, United Kingdom*

BACKGROUND and AIMS: Social cognition deficits are now recognised as ubiquitous sequelae of many forms of acquired brain injury (ABI), and have been linked to a range of negative psychosocial outcomes (including unemployment, family and couples relationship problems and individual survivor distress). However the current interest in the neurobiological basis of social cognitive processes can be contrasted in a dearth of fully developed and evaluated rehabilitation strategies.
METHOD and RESULTS: This presentation reviews the main social cognition intervention types that have been researched with ABI to date: generic social skills training, specific mentalising and emotional recognition skills training, embodied simulation training, interventions targeting adjunctive cognitive control processes and couples therapy interventions. These approaches are described (including video examples) and their theoretical assumptions contrasted.
CONCLUSIONS: A common underlying assumption that deficits can be remediated through explicit training or feedback protocols, which is incongruent with contemporary theoretical and experimental foci on non-explicit processes central to social cognition, such as resonance, contagion and autonomic responsivity, The impact of affective and relational factors has also been neglected. Candidates for new intervention approaches are considered.

Correspondence address: Dr. Giles Yeates, Buckinghamshire NHS PCT, Community Head Injury Service, Jansel Square, HP21 7ET Aylesbury, United Kingdom. E-mail: Giles.Yeates@buckspct.nhs.uk

### Lecture 5: The selectivity of the association between emotion recognition and post-injury behaviour

Maarten Milders
*Heriot-Watt University, School of Life Sciences, Edinburgh, United Kingdom*

BACKGROUND: Recognition of emotions in other people has been proposed as a condition for adequate social behaviour. Impairments in emotion recognition are frequently found in patients with traumatic brain injuries (TBI) and these impairments might underlie the changes in social behaviour post-injury. In line with this suggestion, there have been some reports of associations between emotion recognition and post-injury behaviour. However, questions remain regarding the causal relationship. One requirement for a causal relationship is that the association between emotion recognition and behaviour is selective and no manifestation of other deficits.
METHODS: To examine the selectivity of this association data from 67 patients with TBI and 69 matched healthy controls, were analysed. All participants had completed tests of emotion recognition and various cognitive tasks, including processing speed, which is particularly vulnerable to brain injury. Self and proxy ratings of post-injury behaviour were obtained using the Neuropsychology Behavior and Affect Profile.
RESULTS: Emotion recognition and cognitive task performance were significantly impaired in the TBI group. Proxy ratings of behaviour revealed more problems in the patients than in the controls. Emotion recognition was significantly correlated with communication behaviour and inappropriate behaviour within the patient group. Importantly, these associations could not be explained by injury severity, cognitive impairments or face perception deficits.
CONCLUSION: Emotion recognition was associated with behavioural problems following TBI. The association between emotion recognition and behaviour was selective and not simply a reflection of other impairments. This finding strengthens the possibility that emotion recognition deficits may underlie problems in social behaviour after brain injury.

Correspondence address: Dr. Maarten Milders, Heriot-Watt University, School of Life Sciences, Riccarton, EH14 4AS Edinburgh, United Kingdom. E-mail: m.milders@hw.ac.uk

### Symposium 3: Traumatic Brain Injury: Neuropathology, memory and outcome

Eli Vakil
*Bar Ilan University, Psychology, Ramat Gan, Israel*

Symposium Description: Traumatic brain injury (TBI) is highly prevalent in industrialized countries, particularly among young adults. It is the most common cause of brain damage resulting in long-term disabilities. Characterization of the neuropathology following TBI as well as the short and long term emotional, behavioral and cognitive effects of TBI, could contribute to the assessment and rehabilitation of this patient population. In this symposium, based on cutting-edge research, new empirical findings will be presented that highlight the complexity of the brain injury and its consequences. Dr. Bigler will discuss the utilization of contemporary neuroimaging methods which provide us with a better understanding of the neuropathology following TBI. These methods include T1, T2, fluid attenuated inversion recovery (FLAIR), susceptibility weighted imaging (SWI), magnetic resonance spectroscopy (MRS) and diffusion tensor imaging (DTI). Memory is one of the most (if not the most) common and disabling impairments caused by TBI. It is well known now that the memory system is complex and is composed of various types of memory (e.g., explicit vs. implicit) which are subserved by various brain systems. Dr. Vakil will present empirical data attempting to characterize the nature of the memory impairment following TBI. Finally, Dr. Ponsford will report on a long-term outcome study of patients with moderate to severe TBI that have been followed up at 1,2,3,5, 10 and/or 20 years post-injury. The profile of outcome created based on this study will be described.

Correspondence address: Mr Professor Eli Vakil, Bar Ilan University, Psychology, Ramat Gan, D-52900 Ramat Gan, Israel. E-mail: vakile@mail.biu.ac.il

### Lecture 1: Neuropathology of traumatic brain injury: Neuroimaging findings

Erin David Bigler
*Brigham Young University, Psychology, Provo UT, USA*

Advanced neuroimaging methods continue to provide additional insights into the neuropathology of traumatic brain injury (TBI) and the relationship of pathological changes to neuropsychological outcome. Both human and animal studies integrating in vivo neuroimaging with post-mortem histological analysis have greatly improved our understanding of what types of pathologies are detected with neuroimaging techniques. While neuroimaging methods readily identify obvious acute neuropathologies such as contusions, intraparenchymal hemorrhages, and edema these are but gross indicators of initial pathological change. Neuroimaging abnormalities from TBI especially in the first few months post-injury are dynamic and ever changing. Fortunately contemporary neuroimaging is now capable of detecting and tracking TBI-related neuropathology. Using day-of-injury neuroimaging, which is typically based on computed tomography (CT) as baseline information, a variety of neuropathological changes, if present, may be subsequently identified and quantified using magnetic resonance imaging (MRI). Classic neuropathological indicators as identified at different timeframes post-injury will be reviewed including T1, T2, fluid attenuated inversion recovery (FLAIR), susceptibility weighted imaging (SWI), magnetic resonance spectroscopy (MRS) and diffusion tensor imaging (DTI). Using contemporary automated image analysis techniques, like FreeSurfer, all major brain areas may be quantified, yielding objective anatomical information about any region of interest. DTI permits identification and isolation of all of the major tracts of the brain. However, what may be most important for neuropsychological outcome is how these neuroimaging identified lesions and associated pathologies disrupt neural networks. Contemporary neuroimaging provides a variety of methods that permit the identification of neural networks as well as their disruption from brain injury. Using DTI, functional MRI (fMRI) and resting state fMRI (rs-fMRI), functional connectivity maps of the normal and damaged brain can be derived providing information about not just where lesions may be but how connections and networks are damaged. All of these methods will be reviewed, including 3-D imaging techniques that provide real-time integrative methods to display lesion and network damage to assist the researcher and clinician in better understanding neuropsychological outcome.

Correspondence address: Mrs Professor Erin David Bigler, Brigham Young University, Psychology, 1001 SWKT, 84602 Provo UT, USA. E-mail: erin_bigler@byu.edu

### Lecture 2: Memory impairment following traumatic brain injury

Eli Vakil
*Bar Ilan University, Psychology, Ramat Gan, Israel*

Deficient learning and memory are frequently reported as a consequence of traumatic brain injury (TBI). Characterization of the memory breakdown following TBI could contribute to the assessment and rehabilitation of this patient population. TBI frequently leads to widespread, diffuse axonal injury. Nevertheless, the most common vulnerable memory processes following TBI very much resemble the memory deficits reported in patients suffering frontal lobe damage, e.g., difficulties in applying active or effortful strategy in learning or retrieval process. Because of the diffuse nature of the injury, patients with TBI are not the ideal group for studying brain-behavior relations. But yet, studying this group could contribute to our understanding of the configuration of cognitive processes, which is a critical step towards better understanding of brain-behavior relations.
Breakdown of memory processes could help reveal underlying independent sub-processes that seem inseparable in normal memory functioning.
Numerous studies demonstrate impaired performance on one memory type in some patients with TBI though their performance is preserved on other different memory types. Such findings are an indication of dissociation between these memory types. Examples of such dissociations are: declarative, priming and saving task dissociation; preserved perceptual and impaired conceptual priming; preserved implicit and impaired explicit measures of contextual information; impaired baseline performance and learning rate preserved in skill learning tasks; and preserved tasks that require automatic processes and impaired tasks that require effortful processes. With the continuing improvements of neuroimaging techniques, it is most probably become possible to apply the method of double dissociation with cognitive processes and corresponding brain regions even on this population of individuals with TBI.

Correspondence address: Mr Professor Eli Vakil, Bar Ilan University, Psychology, Ramat Gan, D-52900 Ramat Gan, Israel. E-mail: vakile@mail.biu.ac.il

### Lecture 3: Long-term outcome following traumatic brain injury

Jennie Ponsford
*Monash University, Monash-Epsworth Rehabilitation Centre, Melbourne, Australia*

Traumatic brain injury (TBI) creates many unique challenges for several reasons. It occurs most often in young people who are still establishing their independence and relationships and completing educational or vocational training, and who may have pre-existing social or psychiatric problems. It has diffuse and variable effects on the brain. And recovery occurs over a very lengthy timeframe. This means that the measurement of outcome and the rehabilitation process are exceedingly complex. Through its Longitudinal Head Injury Outcomes study the Monash-Epworth Rehabilitation Research Centre has created a profile of outcome in a series of patients with moderate to severe TBI receiving rehabilitation at Epworth Hospital and followed up at 1,2,3,5, 10 and/or 20 years post-injury. Whilst these patients show good independence in mobility and personal activities of daily living, a significant proportion experience problems with balance and fatigue. About 25–30% require supervision in community based activities such as shopping or financial management due to impairments of memory, attention, processing speed and executive function which, along with irritability and fatigue are the most common long-term cognitive and behavioural problems reported by over 60% of these patients. Age, genetic status and duration of post-traumatic amnesia and cultural background impact on functional outcomes. Psychiatric disorders, particularly anxiety and depression are also common and these are associated with poorer outcomes. In order to improve patient outcomes it is essential that we develop evidence-based methods of addressing each of these issues, whilst also focusing on individually meaningful goals. Our work towards this aim will be outlined.

Correspondence address: Mrs Prof Jennie Ponsford, Monash University, Monash-Epsworth Rehabilitation Centre, Epsworth Hospital, Melbourne, Australia. E-mail: Jennie.Ponsford@monash.edu

### Symposium 4: Causes of cognitive and behavioural problems in Parkinson's disease: Implications for rehabilitation?

Andrew Mayes[1] and Nicky Edelstyn[2]
[1]*Universirty of Manchester, Psychology, Manchester, UK*
[2]*Keele Univeristy – Psychology, Keele, UK*

Parkinson's disease (PD) is defined in terms of specific motor symptoms but PD patients also suffer from a variety of mood, motivational and cognitive disorders that vary in severity, but tend to be progressive and impair quality of life. As well as depression, these disorders include apathy and problems with impulse control, and deficits of executive functions, such as set shifting, attention and working memory, and of long-term memory for events and specific stimuli. The causes of the disorders must be the progressive degenerative changes that underlie PD, which most notably involve the dopaminergic neurons of the midbrain. Degeneration of these dopaminergic neurons may adversely affect functional efficiency of the regions to which they project, such as the prefrontal cortex and medial temporal lobes as well as the basal ganglia. In addition to dopaminergic drugs improving PD motor functions, they can also improve motivational and cognitive functions. However, given that PD patients' various functional deficits have partially non-overlapping pathologies and can differ in severity, it is unsurprising that, when drugs are optimized for their effects on motor functions, they not only also improve some motivational and cognitive functions, but impair others. Optimization of treatment regimens depends on systematic study of the deficits, their neural bases and the effects of different dopaminergic drugs. The three talks in this symposium address these issues. Husain will focus on inattention, impulsivity, apathy and working memory deficits in PD in the context of drug effects and genetic risk in asymptomatic people. Edelstyn and Mayes will focus on recognition memory deficits, their functional causes, disruptive drug effects on recollection and how to avoid them. Duezel will focus on both structural and functional imaging, particularly of basal ganglia and midbrain, both in PD and in healthy ageing in relation to impaired memory and decision making.

Correspondence address: Mr Professor Andrew Mayes, Universirty of Manchester – Psychology, Oxford rd, M13-9PL Manchester, UK. Tel.: +44 161 2752579; E-mail: andrew.mayes@manchester.ac.uk

### Lecture 1: Inattention, impulsivity and apathy in Parkinson's disease

Masud Husain
*University of Oxford, Nuffield Dept of Clinical Neurosciences and Department of Experimental Psychology, Oxford, UK*

Inattention, impulsivity and apathy are recognised as important syndromes associated with Parkinson's disease (PD) that have a significant impact on patients' lives. Although there have been several experimental studies that have investigated attention deficits in PD, we understand very little about the cognitive mechanisms underlying impulsivity and apathy. The goal of this talk will be to consider the relation of attention to working memory deficits in PD. I will present new findings which show how impairments in precision of working memory recall are present at diagnosis (when conventional clinical tests may be normal); are improved by dopaminergic medication; and can be detected in asymptomatic individuals who have a genetic risk of developing PD. In addition, I will present data on impulsivity and apathy which shows that these aspects of behaviour can, respectively, be manifestations of heightened sensitivity to reward or insensitivity to it. Moreover, they are also highly sensitive to dopaminergic modulation. These considerations demonstrate that cognitive deficits in PD are important aspects of the condition which can, crucially, be altered by dopamine levels in the brain.

Correspondence address: Professor Masud Husain, University of Oxford, Nuffield Dept of Clinical Neurosciences and Department of Experimental Psychology, John Radcliffe Hospital, OX3-9DU Oxford, UK. E-mail: masud.husain@ndcn.ox.ac.uk

### Lecture 2: Challenging assumptions about memory in Idiopathic Nondementing Parkinson's disease: Implications for rehabilitation

Nicky Edelstytn[1] and Andrew Mayes[2]
[1]*Keele University, School of Psychology, Staffordshire, UK*
[2]*University of Manchester, School of Psychological Sciences, Manchester, UK*

Two assumptions about the causes of impaired recall and recognition of past episodes and stimuli (episodic memory) in medicated Parkinson's Disease (PD) are examined. The first assumption is that the progressive decline in episodic memory frequently reported in PD results from a breakdown in prefrontally-mediated strategic memory circuits implicated in the generation of encoding and retrieval strategies. The second assumption is that dopaminergic drugs do not affect patients' episodic memory. These assumptions imply that (1) careful guidance in the use of externally provided encoding and retrieval strategies should reduce or remediate episodic memory deficits, and (2) PD recollection should be the same ON and OFF medication. Two studies tested these predictions. The first used a source recognition memory task (RMT) with 3 conditions that compared spontaneous encoding and retrieval (condition 1) with provision of externally provided guidance at encoding only (condition 2) and combined with retrieval (condition 3) on the assessment of familiarity, source recollection and subjective recollection of episodic details in medicated PD and demographically matched healthy volunteers (HVs). In the second study, patients completed 2 matched yes/no RMTs ON and OFF medication (dopamine agonists), and their recollection and familiarity performance was compared to that of HVs. Study 1 showed that "acting as the frontal lobes" by providing optimal encoding and retrieval strategies did not improve recollection significantly more in PD than in HVs. In study 2, one type of dopamine agonist increased the severity of a PD-dependent decline in episodic memory. The studies challenge both assumptions and instead suggest that not only may recollection deficits in PD be 'amnesia-like' but that such deficits may be exacerbated by specific medications. Future memory rehabilitation in PD will need to control expectations and drug regimen carefully and consider drugs that reduce amnesia.

Correspondence address: Professor Nicky Edelstytn, Keele University, School of Psychology, Keele, ST5-5BG Staffordshire, UK. E-mail: n.edelstyn@keele.ac.uk

### Lecture 3: Imaging structural and functional abnormalities of the SN/VTA: Implications for cognitive deficits and rehabilitation

Emrah Duezel
*University College London, Institute of Cognitive Neurosciences, London, UK*

Senescence and Parkinson's Disease affect the ability to utilize information about the likelihood of rewards for optimal decision-making. In a first study, we show that PD patients and older adults have an abnormal functional magnetic resonance imaging (fMRI) signal of expected reward value. In a second study in older adults we show that structural connectivity between the SN/VTA and the striatum measured with diffusion tensor imaging (DTI) was linked to interindividual differences in the fMRI expected reward value signal and that the dopamine precursor L-DOPA restored the neural signals associated with reward prediction errors and improved decision making performance. Under more complex cognitive demands, when appropriate behaviors to obtain rewards and avoid punishments need to be learned concurrently, older adults are better at learning to act ('go') for reward, but better at learning to not act ('no-go') to avoid punishment. However, participants with greater structural integrity of the SN/VTA and the adjacent subthalamic nucleus can overcome this asymmetry. Finally, we show that the dopamine precursor L-DOPA given to older adults during encoding improves episodic memory performance, i.e. the ability to recollect information, after a long retention interval of 6 hours. However, this effect is confined to a narrow dose-range whereby

Correspondence address: Mr Professor Emrah Duezel, University College London – Institute of Cognitive Neurosciences, Queen Square, WC1N-3AR London, UK. E-mail: Emrah.Duezel@dzne.de

### Symposium 5: Managing the psychosocial consequences of dementia

Managing the psychosocial consequences of dementia

Katja Werheid[1] and Alexander Kurz[2]
[1]*Department of Psychology, Humboldt Universität zu Berlin, Berlin, Germany*
[2]*Department of Psychiatry and Psychotherapy, Technische Universität München, München, Germany*

The psychosocial consequences of dementing disorders are far-reaching, both for the patients themselves, and for their families and friends. Whereas diagnostic strategies have been improved and standardized over the past 25 years, psychosocial intervention strategies were only recently considered in clinical research. As no cure is currently available for the neurodegenerative dementias, there are, in the face of ongoing demographic change, two main desiderates. First, the development or adaptation of interventions that meet the patients' and their caregivers' needs, and second, the development of appropriate methods to quantify the effects of these interventions.
In our symposium, we will discuss recent advances in research on psychosocial interventions. In the first lecture, a patient-centered intervention for anxiety in dementia is presented. The two following lectures present studies on caregiver interventions, using modern communication tools and therapy techniques. The two final lectures address methodological issues that are relevant for research on psychosocial interventions in dementia, the choice of outcome measures and gender-fair sampling.

Correspondence address: Prof. Dr. Katja Werheid, Humboldt Universität zu Berlin, Department of Psychology, Unter den Linden 6, D-10099 Berlin, Germany. Tel.: +49 30 2093 9360; Fax: +49 30 2093 9361; E-mail: katja.werheid@hu-berlin.de

### Lecture 1: CBT for anxiety in dementia: A pilot randomised controlled trial

Aimee Spector
*University College London, Clinical Psychology, London, Germany*

Many people with dementia experience anxiety that can cause or exacerbate problems including decreased cognitive function, high physical dependency, relationship difficulties, behavioural problems and increased admittance to care homes. Nonetheless, there is a paucity of research and lack of understanding of psychological approaches for anxiety in dementia, often leading to inappropriate and sometimes problematic use of medication. Cognitive Behavioural Therapy (CBT) focuses on the interplay between thoughts, feelings and behaviour. Small studies have previously evaluated CBT for anxiety in dementia, all reporting clinically meaningful reductions in anxiety and other changes including improved mood. However, to date there are no robust RCTs evaluating its effectiveness. Our team at UCL developed a CBT for anxiety in dementia manual, through identifying the existing evidence; which was modelled through a consensus process. We then assessed the feasibility of the intervention through a single-blind, pilot RCT. 50 people with mild to moderate dementia and anxiety, and their carers; were recruited to a two-armed RCT and receive either CBT or treatment as usual. The adapted CBT intervention involved 10 weekly sessions that teach people new skills to manage their anxiety, with the support of their carer. Blind assessments were conducted at baseline, 15-weeks and at 6 months follow-up, to detect changes in anxiety, quality of life, cognition, behavioural function, mood and relationship with carer. Qualitative interviews were used to gather service-users' perspectives on the intervention. Issues including acceptability, compliance, recruitment and retention were explored. This presentation will describe the CBT for anxiety in dementia programme, illustrate it using case examples, and report the quantitative and qualitative findings.

Correspondence address: Dr. Aimee Spector, University College London, Clinical Psychology, 1-19 Torrington Place, GB-WC1E 7HB London, Germany. Tel.: +49 02076791844; E-mail: a.spector@ucl.ac.uk

### Lecture 2: Long-term evaluation of a randomized-controlled telephone therapy for family caregivers of persons with dementia

Gabriele Wilz[1], Denise Schinköthe[2] and Renate Soellner[3]
[1]*Friedrich-Schiller-Universität Jena, Department of Psychology, Jena, Germany*
[2]*Friedrich-Schiller-Universität, Jena*
[3]*Universität Hildesheim*

GOALS: Meta-analyses indicate that interventions for family caregivers of persons with dementia show heterogeneous and moderate to low positive effects and investigations of long-term effects are rare. Thus, the development and long-term evaluation of adequate and effective interventions is still needed to prevent physical and mental illness of the caregivers. Therefore, in this study we focused on the evaluation of long duration effects of a cognitive-behavioral (CBT) telephone intervention adapted to the special situation of family caregivers.

METHODS: A randomized controlled trial ($N = 229$) was conducted to compare treatment group CBT intervention, treated control group (relaxation techniques) and untreated control group. Outcome measures were physical complaints, depressive symptoms, caregiver's perceived health and well-being. All measures were administered before treatment (pre), after treatment (post), six month and two years after treatment (follow-up).

RESULTS: Caregivers of the CBT intervention group show positive long-term effects on health outcomes such as significant increases perceived health and well-being and reduction in body complaints and depression. Taken into account participants' evaluation of the intervention, CBT participants reported a higher effect concerning overall satisfaction with the treatment, pointing to intervention specific effects compared to the general effects of an intervention, which have been controlled by the introduction of an active control group (PMR).

CONCLUSION: Discussion focuses on limitations and challenges of the investigation of long-term-effects in this population. Considerations will be undertaken about the evaluation of specific intervention modules and the implementation of this specific psychotherapy for caregivers into the health system.

Correspondence address: Prof. Dr. Gabriele Wilz, Friedrich-Schiller-Universität Jena, Department of Psychology, Humboldtstraße 11, D-07743 Jena, Germany. Tel.: +49 3641 945170; Fax: +49 3641 945172; E-mail: gabriele.wilz@hu-berlin.de

### Lecture 3: Internet-based supportive interventions for caregivers of people with dementia

Marjolein De Vugt[1], Lizzy M.M. Boots[2], Gertrudis I.J.M. Kempen[2] and Frans Verhey[2]
[1]*Maastricht University, Alzheimer Center Limburg, School for Mental Health and Neuroscience, Maastricht, Germany*
[2]*University of Maastricht*

BACKGROUND: Due to the expected increase of dementia patients, no cure for the patient in sight, and the rising cost of care, there is a growing need for effective caregiver interventions. Internet-based interventions hold a considerable promise for meeting the educational and support needs of dementia carers at reduced expenses.

OBJECTIVES: The current study aims to (1) give a systematic overview of the literature on Internet interventions for caregivers of people with dementia (PwD) (2) develop a prototype of an Internet-based self-management programme to provide dementia caregivers the skills they need to undertake and succeed in the caregiver role.
METHODS: The first objective was addressed by a systematic search of the literature published up to January 2013. Twelve studies were identified. The quality of the included studies was assessed according to the Cochrane Level of Evidence (LOE) and Cochrane Back Review Group criteria. For the second objective an iterative process was used with expert and consumer input to develop the initial prototype of a self-management program for caregivers of PwD.
RESULTS: The included studies in the review differed widely in terms of intervention type, dosage, duration, and methodological quality. Outcomes showed that Internet interventions can improve various aspects of caregiver well being, e.g. burden, depression and self-efficacy, provided they comprise of multiple components, are individually tailored, and include interaction with other caregivers. Nine thematic modules were selected and included in a phase I prototype: 'acceptance', 'insecurity', 'focus on the positive', 'communication', 'balance in activities', 'stress and relaxation', 'social support', 'a changing partner', and 'care management strategies'.
CONCLUSION: Results from the literature study and development study provide support to move forward with a feasibility and cost-effectiveness study of an Internet-based support program for caregivers in dementia.

Correspondence address: Dr. Marjolein de Vugt, Maastricht University, Alzheimer Center Limburg, School for Mental Health and Neuroscience, Dr. Tanslaan 12, D-6229 Maastricht, Germany. Tel.: +31 43 3877445; Fax: +31 43 3875444; E-mail: m.devugt@maastrichtuniversity.nl

### Lecture 4: Outcome measures for patient- and carer-centered interventions in dementia

Alexander Kurz
*Technische Universität München, Department of Psychiatry and Psychotherapy, München, Germany*

Dementia is multi-dimensional concept defined by impairment of cognition, alterations of behaviour and reduced abilty to carry out usual activities of daily living. The measures that are commonly used to capture these dimensions constitute the classic oucome triad of clinical drug trials and longitudinal studies. The psychosocial consequences of dementia involve both the patient and the informal carer. On both sides, subjective and objective impacts can be distinguished. Subjective consequences are similar for patients and carers and include mood, quality of life, self-efficacy, sense of mastery and marital satisfaction. Objective consequences, on the other hand, are partly different between dementia sufferers and carers. On the patients' side these refer to independence, attainment of personally relevant goals, activity and participation and legal capacity. On the carers' part consequences refer to time spent on caring, working hours, financial income, leisure time and social activity. For each one of these consequences assessment instruments are available that can be applied in studies. Obviously, the appropriate set of outcomes must be selected in relation to the study objectives.

Correspondence address: Prof. Dr. Alexander Kurz, Technische Universität München, Department of Psychiatry and Psychotherapy, Möhlstr. 26, D-81675 München, Germany. Tel.: +49 8941404201; Fax: +49 89414 04860; E-mail: alexander.kurz@lrz.tu-muenchen.de

### Lecture 5: Effectiveness of psychosocial interventions in early dementia: Evidence of gender bias

Katja Werheid[1], Stefanie Baron[2] and Ingun Ulstein[3]
[1]*Humboldt Universität zu Berlin, Department of Psychology, Berlin, Germany*
[2]*Humboldt-Universität zu Berlin*
[3]*National Center of Aging and Health, Oslo, Norway*

BACKGROUND: Alzheimer's disease affects twice as many women than men. Gender differences in symptom profile, living conditions, coping style and treatment response might affect the outcome of psychosocial interventions for patients with Alzheimer's disease (AD) or Mild cognitive impairment (MCI). Neglecting this variable in clinical trials could lead to biased results and thereby diminish the external validity of findings on treatment effectiveness.
METHODS: A systematic review of high-ranking, randomized-controlled clinical trials on psychosocial interventions in AD and MCI was conducted by searching electronic databases (PubMed, Medline and PsycINFO, 2000–2012). The studies were analysed for their gender proportion and for gender differences reported.
RESULTS: Sixty-two percent of the available studies reported the male:female ratio for each treatment group and were included in a stepwise analysis. They deviated from the prevalence-based gender ratio in the control groups, with significantly less females than expected. Nineteen percent of the reviewed study pool reported analyses of gender differences. In this subgroup of studies, females were underrepresented in treatment and control groups. Six of these studies showed significant differences in out-come between the genders. In these studies, the gender ratio was in accordance with prevalence rates, in contrast to the studies in which no gender effects were found.
CONCLUSIONS: Our review provided evidence for a selection bias resulting in female underrepresentation in high-ranking studies on psychosocial interventions in AD/MCI. Moreover, a systematic linkage of female underrepresentation and reports of analysed gender differences suggests a reporting bias.

Correspondence address: Prof. Dr. Katja Werheid, Humboldt Universität zu Berlin, Department of Psychology, Unter den Linden 6, D-10587 Berlin, Germany. Tel.: +49 30 2093 9360; Fax: +49 30 2093 9361; E-mail: katja.werheid@hu-berlin.de

### Symposium 6: Neuropsychology of metacognition

Siegfried Gauggel and Effi Volz-Sidiropoulou
*University Hospital RWTH Aachen, Medical Psychology and Medical Sociology, Aachen, Germany*

The interest in the investigation of metacognitive processes has been considerable increased in recent years. Metacognition refers to higher order thinking which involves active control over one's cognitive processes. Research in metacognition have been carried out by (a) cognitive and social psychologists who have addressed the mechanisms of metacognitive processes, and (b) neuropsychologists and neurologists who have highlighted the associations between impairments in specific areas of the brain or limitations in the awareness of cognitive functioning and deficits in specific cognitive processes (e.g., executive functions). Synthesizing the outcomes of both approaches would be very fruitful for understanding the nature and neural basis of (meta-) cognitive processes. The investigation of metacognitive processes is also important for clinical work. Current research has repeatedly shown a clear association between metacognition and cognitive functioning with poor metacognitive ability to have prognostic validity for impaired cognitive functioning. The present symposium provides an introduction in the neuropsychology of metacognition and four empirical studies investigating different aspects of metacognition.

Correspondence address: Mr Prof. Siegfried Gauggel, University Hospital RWTH Aachen, Medical Psychology and Medical Sociology, Pauwelsstrasse 19, D-52074 Aachen, Germany. Tel.: +49 0241 8089000; E-mail: sgauggel@ukaachen.de

### Lecture 1: Neuropsychology of metacognition: An introduction

Siegfried Gauggel
*University Hospital RWTH Aachen, Medical Psychology and Medical Sociology, Aachen, Germany*

OBJECTIVE: In this introduction a short overview of current research in the neuropsychology of metacognition is provided.
METHODS: A systematic review of the research literature is performed considering major studies and theoretical papers.
RESULTS: A large number of empirical and theoretical paper could be identified focusing on the (a) neural basis of unawareness of cognitive, behavioural and motor impairments, (b) functional architecture of metacognitive processes, (c) development of reliable and valid assessment procedures to measure metacognitive processes and disorders of unawareness, and (d) treatment of disorders of awareness of deficits in patients with an ABI and other mental disorders.
CONCLUSION: Many theories have been proposed to account for disorders of awareness (especially anosognosia). However, none of these theories appear satisfying. Further research is needed integrating not only neuropsychological methods and findings, but also concepts like self-concept, self-serving bias, social cognition, self-enhancement, self-protection etc.

Correspondence address: Prof. Siegfried Gauggel, University Hospital RWTH Aachen, Medical Psychology and Medical Sociology, Pauwelsstrasse 19, D-52074 Aachen, Germany. Tel.: +49 0241 8089000; E-mail: sgauggel@ukaachen.de

### Lecture 2: Self-Awareness in children and adolescents with attention deficit hyperactivity disorder: Further evidence of the positive illusory bias

Effi Volz-Sidiropoulou, Maren Böcker and Siegfried Gauggel
*University Hospital RWTH Aachen, Medical Psychology and Medical Sociology, Aachen, Germany*

OBJECTIVE: This study aimed to examine the accuracy of self-reports of children and adolescents with attention-deficit/hyperactivity disorder (ADHD) in evaluating their competence in every-day activities.
METHOD: Self-reports of children and adolescents with ADHD ($n = 89$) were compared with those of non-referred children ($n = 94$), and also to parental reports of their children's competence in performing daily activities. Competence was measured with a 34-item rating scale. Behavioural disorders were documented with the Child Behaviour Checklist.
RESULTS: Children and adolescents with ADHD were much more likely than controls to overestimate their competence in certain daily life activities relative to their parents' reports, demonstrating a positive illusory bias. The positive illusory bias was found to be moderated by coexisting behavioural problems and was pronounced in activities that were expected to be affected by ADHD symptoms. Overestimated self-perceptions of competence were more likely to be accompanied with externalizing problems such as aggression.
CONCLUSION: Results support the presence of the positive illusory bias also in the domain of everyday life activities. In behavioural treatment programs, improvement of self-evaluation of competencies should also become a focus of treatment.

Correspondence address: Dr. Effi Volz-Sidiropoulou, University Hospital RWTH Aachen, Medical Psychology and Medical Sociology, Pauwelsstrasse 19, D-52074 Aachen, Germany. Tel.: +49 0241 8089002; E-mail: evolz-sidiropoulou@ukaachen.de

### Lecture 3: Do subjective measures of attention and memory predict actual performance? Metacognition in older couples

Effi Volz-Sidiropoulou and Siegfried Gauggel
*University Hospital RWTH Aachen, Medical Psychology and Medical Sociology, Aachen, Germany*

OBJECTIVE: Older individuals who recognize their cognitive difficulties are more likely to adjust their everyday life to their actual cognitive functioning, particularly when they are able to estimate their abilities accurately.
METHODS: We assessed self- and spouse-ratings of memory and attention difficulties in everyday life of healthy, older individuals and compared them with the respective test performance. Eighty-four older individuals (women's age, M = 67.4 years, SD = 5.2; men's age, M = 68.5 years, SD = 4.9) completed both the self and the spouse versions of the Attention Deficit Questionnaire and the Everyday Memory Questionnaire and completed two neuropsychological tests. Using the residual score approach, subjective metacognitive measures of memory and attention were created and compared with actual test performance.
RESULTS: Significant associations between subjective and objective scores were found only for men and only for episodic memory measures. Men who underreported memory difficulties performed more poorly; men who overreported memory difficulties performed better. Men's recognition performance was best predicted by subjective measures ($R^2 = 0.25$), followed by delayed recall ($R^2 = 0.14$) and forgetting rate ($R^2 = 0.13$).
CONCLUSION: The results indicate gender-specific differences in metacognitive accuracy and predictive validity of subjective ratings.

Correspondence address: Dr. Effi Volz-Sidiropoulou, University Hospital RWTH Aachen, Medical Psychology and Medical Sociology, Pauwelsstrasse 19, D-52074 Aachen, Germany. Tel.: +49 0241 8089002; E-mail: evolz-sidiropoulou@ukaachen.de

### Lecture 4: Content matters: Degree of patient-proxy agreement is moderated by the measured construct

Maren Böcker, Effi Volz-Sidiropoulou and Siegfried Gauggel
*University Hospital RWTH Aachen, Medical Psychology and Medical Sociology, Aachen, Germany*

OBJECTIVE: Impaired awareness of motor, cognitive and behavioral impairments and disabilities is a quite serious and prevalent problem in patients acquires brain injuries (ABI). As reduced awareness has been shown to negatively influence rehabilitation outcome, the assessment of possible awareness deficits is of high importance. One way to measure lack of awareness is the comparison of a patient's self rating with a rating made by significant others or staff members. However, it has been shown that the rater agreement can be very inconsistent and might be influenced by factors as e.g. intrahemispheric locus of the lesion or the construct itself.
METHODS: In the present study we investigated the degree of patient-proxy agreement for five different aspects of the ICF-domains activities and participation. For this purpose a consecutive sample of 94 ABI outpatients (39 female, 55 male; mean age: 45.5 (14.1)) was assessed in four different German outpatient rehabilitation facilities. Self and proxy (spouse or next of kin) ratings were assessed by means of the five newly developed subscales of the Aachen Functioning Item Bank (AFIB), namely mobility, fine motor function, gross motor function, cognition I (attention, memory, orientation, communication) and cognition II and participation (executive functions, emotion regulation, interpersonal interactions, work). All five AFIB-subscales were developed conforming to the Rasch model and items for this study were selected in a way that no Rater-Differential Item Functioning existed.
RESULTS: Results indicate that patient-proxy agreement was good and of similar size for the subscales mobility, fine and gross motor function with correlations between self and proxy rating ranging from 0.68 to 0.72. However, patient-proxy agreement was substantially lower for cognition I ($r = 0.52$) and especially for cognition II and participation ($r = 0.39$).
CONCLUSION: Implications of these results for the clinical assessment disorders of awareness are discussed.

Correspondence address: Dr. Maren Böcker, University Hospital RWTH Aachen, Medical Psychology and Medical Sociology, Pauwelsstrasse 19, D-52074 Aachen, Germany. Tel.: +49 0241 8088235; E-mail: mboecker@ukaachen.de

### Lecture 5: Metacognitive monitoring and somatic awareness: Same or different?

Thomas Forkmann, Judith Meessen, Verena Mainz and Siegfried Gauggel
*University Hospital RWTH Aachen, Medical Psychology and Medical Sociology, Aachen, Germany*

OBJECTIVE: Metacognitive monitoring has repeatedly been studied, especially concerning memory encoding and retrieval and deficits. Regarding the monitoring and accurate of the perception of body processes, especially one's own heartbeat, it has been shown that better cardiac awareness (i.e., higher congruence between subjectively experienced and objectively measured heart rate) is related to better performance in cognitive tasks. The relation between metacognitive monitoring of memory processes and monitoring of body processes such as heartbeats has not been studied yet. In this presentation the available literature on metacognitive monitoring of memory processes and cardiac awareness will be reviewed and first empirical data on its relationship will be presented.
METHODS: Healthy participants performed the heartbeat perception task and a Judgment of Learning paradigm which measures the metacognitive accuracy regarding memory processes. The sequence of the two paradigms were be balanced and results were controlled for age, sex, body mass index, physical fitness, etc. Until now, pilot data from 9 participants have been collected.
RESULTS: Preliminary data show a positive correlation between cardiac awareness and metacognitive accuracy for memory processes ($r = 0.42$).
CONCLUSION: Findings suggest a general and not domain specific metacognitive ability provided that this result will be confirmed in the larger sample with sufficient power.

Correspondence address: PD Dr. Thomas Forkmann, University Hospital RWTH Aachen, Medical Psychology and Medical Sociology, Pauwelsstrasse 19, D-52074 Aachen, Germany. Tel.: +49 0241 8089003; E-mail: tforkmann@ukaachen.de

### Symposium 7: Symptom Validity Tests In Neuropsychology: No Excuse For Not Using Them.

Brechje Dandachi-FitzGerald
*University of Maastricht, Maastricht, Netherlands, School for Mental Health and Neurosciences (MHeNS), Maastricht, Netherlands*

An important aspect of neuropsychological assessment is its contribution to both clinical and forensic decision making. This decision making can become distorted when patients or defendants produce non-credible symptoms. Over the past years, the body of knowledge about symptom credibility has grown to a degree that ignoring the issue of symptom credibility is incompatible with evidence-based practice. The focus of this symposium will be on symptom credibility in neuropsychological assessment. Currently, neuropsychologists have several tools for examining the credibility of reported symptoms and test performance at their disposal. When it comes to symptom credibility, there are no arguments left to rely on clinical impression alone.

How European neuropsychologists address symptom credibility in their neuropsychological assessment will be discussed in the paper of Brechje Dandachi-Fitz Gerald et al. This paper presents data of a large-scale survey among neuropsychologists in six European countries. An elegant experimental study on detecting feigned amnesia by Peter Giger et al. illustrates the importance of using a combination of tools for evaluating symptom credibility. This multi-method approach is also an important feature in the research of Rudolf Ponds et al., which indicates that symptom credibility is not only relevant to the forensic, but also to the clinical domain. In his presentation, he will discuss the prevalence of non-credible symptoms in general hospital patients, and how this impacts standard clinical test results. In the final presentation, Harald Merckelbach will present data illustrating that non-credible symptoms do not only represent a complication of diagnostic assessment, but may eventually become real for the person who engages in feigning of symptoms.

Correspondence address: Mrs Brechje Dandachi-Fitz Gerald, University of Maastricht, Maastricht, Netherlands, School for Mental Health and Neurosciences (MHeNS), Kapelstraat 12, NL-6226 EB Maastricht, Netherlands. E-mail: b.fitzgerald@maastrichtuniversity.nl

### Lecture 1: Symptom validity and neuropsychological assessment: A survey of practices and beliefs of neuropsychologists in six European countries

Brechje Dandachi-FitzGerald
*University of Maastricht, School for Mental Health and Neurosciences (MHeNS), Maastricht, Netherlands*

Symptom validity has become an important topic over the past decade in the neuropsychological and psychiatric literature as it relates to malingering, factitious disorder, and somatoform complaints. We conducted a survey among neuropsychologists ($N = 515$) from six European countries (Germany, Italy, Denmark, Finland, Norway, and The Netherlands). Respondents were asked questions about the tools they use to evaluate symptom credibility in clinical and forensic assessments and about other issues related to symptom validity testing. The majority of respondents exhibited technical knowledge about symptom validity, although a sizeable minority was found to rely on outdated notions (e.g., the idea that clinicians can determine symptom credibility on the basis of intuitive judgment). Little consensus exists among neuropsychologists as to how to instruct patients when they are administered symptom validity tests (SVTs) and how to handle test failure. Our findings indicate therefore that the issues of how to administer SVTs and how to communicate their results to the patients warrant systematic research.

Correspondence address: Mrs Brechje Dandachi-Fitz Gerald, University of Maastricht, School for Mental Health and Neurosciences (MHeNS), P.O. Box 616, NL-6200 MD Maastricht, Netherlands. E-mail: b.fitzgerald@maastrichtuniversity.nl

### Lecture 2: The assessment of feigned memory impairment: An example of a multi-method approach

Peter Giger
*University of Bern, Switzerland, Section of Psychology and Law, Maastricht, Switzerland*

The assessment of reported memory impairment is a common task for neuropsychologists. In cases where secondary gain is at stake the possibility of symptom exaggeration or malin-gering has to be always kept in mind. This is particularly true in criminal forensic settings where feigned crime-related amnesia is a strategy of offenders to avoid criminal responsibil-ity. There are numerous tests available that can help experts to identify false amnesia claims (so-called symptom validity tests; SVTs). However, there are two main problems that cannot be ignored: First, malingering is a multidimensional phenomenon, and consequently different strategies may be employed by individuals who feign mental disorders, and second, the question of correct classifications or diagnostic accuracy. The primary goals of the current study were to investigate how well feigned amnesia can be detected using a multi-method approach and to what extent this approach can reduce false classifications. In an experi-mental study, 60 participants were instructed to commit a mock crime and were then as-signed to three conditions: responding honestly; feigning amnesia; feigning amnesia with a warning not to exaggerate. A test battery consisting of tests of underperformance and symp-tom exaggeration was administered. High specificity rates were found for all tests, with sensi-tivity rates ranging from 45 to 88 percent. Five of seven tests correctly classified all honest participants, and not one naïve malingerer passed all SVT. Only three warned malingerers went undetected throughout the test battery. The results demonstrate that promising instru-ments exist to support decision making about feigned memory problems. Moreover, a multi-method approach increases the diagnostic accuracy and keeps the risk of misclassifying honest responders at an adequate level. The findings may serve as an example of how two disciplines, neuropsychology and forensic psychology, can learn and benefit from each other.

Correspondence address: Mr Peter Giger, University of Bern, Switzerland, Section of Psychology and Law, Hochschulstrasse 4, Swiss-3012 Maastricht, Switzerland. E-mail: p.giger@gmx.net

### Lecture 3: Non-credible symptom reports of patients referred for neuropsychological assessment in a general hospital

Rudolf Ponds
*Maastricht University Medical Center, Department of Medical Psychology, Maastricht, Netherlands*

The current study looked at the prevalence of non-credible symptom reports during neuropsychological assessment in a general hospital setting. Patients with obvious cognitive impairment (e.g., dementia, acute psychosis, M. Parkinson) were excluded. All patients ($N = 93$) were routinely referred for neuropsychological assessment. Neuropsychological assessment included two symptom validity tests (SVTs): the Amsterdam Short Term Memory test (ASTM) measuring underperformance and the Structured Inventory of Malingered Symptomatology (SIMS) tapping into over-reporting symptoms. Using the recommended cut-offs, prevalences of SVT failure were as follows: 45% of the patients passed both tests, 30% failed either the ASTM or the SIMS, and 25% failed both tests. We examined how groups performed on the Rey's Verbal Learning Task (VLT) and the Brief Symptom Inventory (BSI). The group that failed the ASTM ($n = 44$) recalled on average 39 words over trial 1–5 of the VLT, whereas the group that passed the ASTM ($n = 48$) obtained a mean total recall trial 1–5 of 48 words ($t = 3.58$, $p < 0.01$). Also, the delayed recall differed significantly between the ASTM fail group (mean = 9.9) and the ASTM pass group (mean = 7.3) ($t = 3.22$, $p < 0.01$). The group that scored deviant on the SIMS obtained a higher average BSI item score ($n = 24$, mean item score = 0.68) than the group that scored within the normal range ($n = 66$, mean item score = 1.67) ($t = 5.4$, $p < 0.01$). These results will be further discussed in light of the demographic characteristics (e.g. diagnosis upon referral, social situation, involvement in legal procedures).
Our results indicate that in a general hospital setting, non-credible symptom reports occur in a substantial minority of neuropsychological evaluations and when it occurs it has a significant impact on test outcomes. Our findings call for routinely assessing symptom validity in a neuropsychological evaluation in hospital patients without obvious cognitive impairment.

Correspondence address: Mr Rudolf Ponds, Maastricht University Medical Center, Department of Medical Psychology, P.O. Box 5800, NL-6202 AZ Maastricht, Netherlands. E-mail: r.ponds@maastrichtuniversity.nl

### Lecture 4: Residual effects of symptom fabrication

Harald Merckelbach
*Maastricht University, Forensic Psychology Section, Maastricht, Netherlands*

In this presentation, I will argue that symptom fabrication is more than just a complication of the diagnostic process. That is, symptom fabrication itself tends to produce residual symptoms that the person experiences as genuine. In one recent experiment, we had two experimental groups ($n = 15$ each) that were given a legal scenario with the option to exaggerate symptoms. Compared to a control condition ($n = 15$), both groups scored significantly higher on the Structured Inventory of Malingered Symptomatology. Next, one group was confronted in a sympathetic way about their symptom validity test failure, whereas the other group was confronted in a neutral manner. Both groups subsequently completed the Brief Symptom Inventory (BSI). BSI scores of both feedback groups remained significantly higher than those of control participants. Participants who had been provided with sympathetic feedback or neutral feedback did not differ in their BSI scores. Even participants who indicated during the exit interview that they had given up symptom exaggeration attained significantly higher BSI scores than controls, indicating that exaggeration has residual effects that are resistant to corrective feedback. Our findings accord well with various research lines that show that other-deceptive acts (e.g., symptom fabrication) tend to develop into self-deception (e.g., believing that you do suffer from symptoms). Why is that the case? I will discuss cognitive dissonance as a model for understanding residual effects of symptom exaggeration and also address the implications for assessment and follow-up interventions.

Correspondence address: Harald Merckelbach, Maastricht University, Forensic Psychology Section, P.O. Box 616, NL-6200 MD Maastricht, Netherlands. E-mail: h.merckelbach@maastrichtuniversity.nl

### Symposium 8: Progress in rehabilitation of visual deficits after brain injury

Arash Sahraie
*University of Aberdeen, School of Psychology, Aberdeen, UK*

Visual deficits following brain injury range from minor disturbances of brightness perception to full blindness in one or both hemifields, often affecting oculomotor control, reading, and visual attention. Similar to language and motor deficits after brain injury, vision can benefit from clinical intervention leading to partial or full recovery of the deficit, improving patients' quality of life. The sympsosium is intended to highlight the recent progress in this field. Results from clinical studies using home based eye-movement interventions will be presented. Following an introduction on the computer-based assessment of visual deficits (Niedeggen), recent findings on reading interventions and the effect of hemianopia on reading will be discussed (Schenk). Methods and recent findings of changes in visual sensitivity following systematic stimulation will be outlined (Sahraie) together with novel approaches on using multi-sensory integration for rehabilitation purposes (Ladavas).

Correspondence address: Mr Professor Arash Sahraie, University of Aberdeen, School of Psychology, Kings College, GB-AB23 8UL Aberdeen, UK. E-mail: a.sahraie@abdn.ac.uk

### Lecture 1: Computer-based assessment of visual functions

Michael Niedeggen
*Freie Universität Berlin, Experimental Psychology and Neuropsychology, Berlin, Germany*

The rehabilitation of visual deficits critically depends on a detailed examination of the different functions, i.e. color, object, or motion processing. However, clinical neuropsychological routine is often restricted to the examination of the visual field. We developed a computer-based assessment of visual functions (CAV) based on force-choice procedures in order to reduce mnestic and linguistic demands. Subtests can be summarized to estimate (1) apperceptive and associative object perception, and (2) perceptual and cognitive space perception. The CAV was applied to a sample of 30 neurological patients with posterior lesions, and data were compared with the normative data available ($n = 150$ healthy subjects). With respect to object perception, apperceptive deficits (PR $<$ 10: 6/30 patients) were more frequently observed as compared to associate deficits (PR $<$ 10: 4/30 patients). Visuo-spatial deficits with a perceptual component (i.e. line orientation) were less frequent (PR $<$ 19: 5/30 patients) than deficits with a cognitive component (i.e. line bisection, PR $<$ 10: 8/30 patients). The results indicate that the prevalence of object agnosia or visuo-spatial deficits is probably underestimated. In patients with posterior lesions these deficits might affect the success of neuropsychological rehabilitation.

Correspondence address: Mr Professor Michael Niedeggen, Freie Universität Berlin, Experimental Psychology and Neuropsychology, Habelschwerdeter Allee 45, D-14195 Berlin, Germany. E-mail: niedegg@zedat.fu-berlin.de

### Lecture 2: Efficacy and feasibility of a home-based computer training for individuals with homonymous visual field defects

Thomas Schenk[1], Lina Aimola[2], Alison R. Lane[2], Daniel T Smith[2], Georg Kerkhoff[3] and Gary Ford[2]
[1]*University of Erlangen, Cognitive Neurology, Erlangen, Germany*
[2]*University of Durham, Department of Psychology, Durham, UK*
[3]*University of Saarland, Department of Psychology/ Neuropsychology, Saarbrucken, Germay*

Homonymous visual field defects (HVFDs) are one of the most common consequences of stroke. Effective compensatory training has been developed which helps such individuals to function more successfully by encouraging them to develop more efficient eye-movements. However, such training is typically undertaken in clinical settings, or at home with supervision, which can be both time-consuming and costly. We developed and evaluate the efficacy and feasibility of an unsupervised home-based computer training for individuals with HVFDs, which includes both reading and visual exploration components. Seventy individuals with HVFDs were randomly assigned to one of two groups: experimental or control. The former received 35 hours of combined reading and exploration training, and the latter 35 hours of a control attention training. Visual and attentional abilities were assessed both before and after training using perimetry, visual search, reading, activities of daily living, the Test of Everyday Attention, and a Sustained Attention to Response task. Patients in the experimental group showed considerably more improvements in tests of exploration and reading than patients in the control group. We will discuss whether home-based training is a cheap and accessible rehabilitation option for patients with HVFDs.

Correspondence address: Professor Thomas Schenk, University of Erlangen, Cognitive Neurology, Schwabachanlage 6, D-91054 Erlangen, Germany. E-mail: Thomas.schenk@uk-erlangen.de

### Lecture 3: Effect of systematic training on vision loss caused by post-geniculate brain injury

Arash Sahraie[1], Amelia Hunt[1], Ceri T. Trevethan[1], Kay Ritchie[1] and Mary-Joan MacLeod[2]
[1]*University of Aberdeen, School of Psychology, Aberdeen, UK*
[2]*University of Aberdeen, School of Medicine and Dentistry, Aberdeen, UK*

In hemianopic patients, Blindsight type I refer to detection within field defect in the absence of any awareness, whereas type II blindsight refers to above chance detection with reported awareness, but without seeing per se. Systematic sensory stimulation is the principle approach in many sensory and motor impairments in brain damaged patients. The parameters for visual stimulation are crucial in mediating any change. In detailed case studies, evidence for dependency of awareness responses on the stimulus properties will be presented. In addition in a number of cases it appears that the detection ability at early stages of training varies as a function of distance of the stimulated area from the sighted field border. There is a lack of detection ability at retinal locations deep within the field defect. Nevertheless following repeated stimulation and after 5,000 to 10,000 trials, the detection performance improves. Therefore, there appears to be a continuum of performance from no detection, to blindsight type I, and eventually type II detection. Recent findings of eye-movement dependent visual sensitivity changes will also be presented together with discussion of possible mechanisms for recovery of function.

Correspondence address: Mr Professor Arash Sahraie, University of Aberdeen, School of Psychology, Kings College, GB-AB23 8UL Aberdeen, UK. E-mail: a.sahraie@abdn.ac.uk

### Lecture 4: Audio-visual perceptual learning in the hemianopic field

Elisabetta Ladavas, C. Bertini and N Dundon
*University of Bologna, Department of Psychology, Bologna, Italy*

The human brain possesses a flexible audio-visual system, which interprets and guides responses to external events according to spatial alignment, temporal synchronization and effectiveness of unisensory signals. Here we explore the possibility that such a system might represent the neural correlate of sensory compensation when a sensory modality has been damaged by a cerebral lesion (patients with hemianopia). We will examine on-line and off-line effects of audio-visual stimulation on the visual and spatial impairments following damage to the geniculo-striate pathway. We will demonstrate that an improvement in spatial orienting can be obtained not only when an on-line response is required, but also after either a brief or a long adaptation to audio-visual stimulation. These findings suggest that the mechanisms subserving audio-visual perceptual learning are still active after a damage to the geniculo-striate pathway; this perceptual learning is probably driven by recruitment of the retino-colliculo-extrastriate pathway.

Correspondence address: Mrs Professor Elisabetta Ladavas, University of Bologna, Department of Psychology, Bologna, GB-AB23 8UL Bologna, Italy. E-mail: a.sahraie@abdn.ac.uk

### Symposium 9: Pain in dementia: the miserable sequence of not seeing, not believing and not acting

Stefan Lautenbacher
*University of Bamberg, Physiological Psychology, Bamberg, Germany*

The symposium aims at providing a conception of the difficulties involved in assessing and treating pain in patients with dementia. The audience should become aware that the difficulties may arise at any stage of the process (i) starting from the failed detection of pain, (ii) continuing with misleading diagnosis and (iii) ending with the reluctance of using all available treatment options (e.g. erroneous prescription of only too weak analgesics or psychotropic drugs). In other words, a main objective is the acquisition of professional sensitivity to the miserable sequence of not seeing, not believing and not acting when faced with pain in dementia. Presented information should help to understand that each of the three stages contains particular problems, which may limit the quality of the next stage, but that each stage produces problems ex novo. According to the latter, not even valid diagnoses result necessarily into sufficient treatment. Improved awareness of these caveats will better know the prerequisites to reduce suffering from pain in demented patients but also how to ameliorate concurrent neuropsychological and -psychiatric symptoms and to avoid unnecessary psychotropic drug prescription.

Correspondence address: Prof. Stefan Lautenbacher, University of Bamberg, Physiological Psychology, Markusplatz 3, D-96045 Bamberg, Germany. Tel.: +49 0951 863 1851; Fax: +49 0951 863 1976; E-mail: stefan.lautenbacher@uni-bamberg.de

### Lecture 1: Not seeing the pain in patients with dementia

Miriam Kunz
*University of Munich, Biological Psychology, Munich, Germany*

Given the limited ability of patients with dementia to communicate pain verbally (due to the cognitive decline across the course of dementia), observation of non-verbal pain behavior (especially the facial expression) is essential when trying to assess pain. However, when pain assessment is solely based on non-verbal pain behavior, misjudgment often occurs. “Observers” mostly tend to not see the pain encoded in the facial expression of the individual suffering from pain and thus underestimate the pain. In this talk, experimental evidence will be presented on judging pain in patients with different degrees of dementia (as well as in young and older individuals) based on their facial expression. It will be discussed to which degree the age and the cognitive status of the individual in pain, affect the ability of others to see their pain. Moreover, data on different types of observers (e.g. young and old, elderly care nurses, male and females) will be presented that emphasize that even long-standing professional experience with elderly patients in pain does not necessarily improve the pain detection. Clinical implications of these experimental findings will be discussed.

Correspondence address: Prof. Miriam Kunz, University of Munich, Biological Psychology, Leopoldstr. 13, D-80802 Munich, Germany. Tel.: +49 089 2180 6927; Fax: +49 089 2180 5233; E-mail: miriam.kunz@lmu.de

### Lecture 2: How misjudgment and misbelieve prevents adequate pain diagnostic and treatment in patients with dementia

Sandra Zwakhalen
*Maastricht University, Contact Health Services Research, GT Maastrich, The Netherlands*

Previous research has identified knowledge deficits and misbeliefs about pain in dementia among nurses, who play an important role in decision-making for pain management. Such misconceptions may also prevent the adequate use of diagnostic procedures. It has yet been unclear as to what extent the use of standardized pain assessment relieves interventions undertaken by nurses. To determine the relationship between the initiation of intervention and the use of standardized pain diagnosis in demented patients, an observational scale named PACSLAC-D was used in a longitudinal study. Data were collected during a six-week period (twice a week). Interventions undertaken as result of a critical pain score were recorded. Compliance was evaluated with the nursing staff involved. In total, 264 pain assessments were conducted; 60 observations resulted in a critical pain score, which did however not necessarily lead to any intervention. In sum, although there was a high compliance with using the standardized pain scale, pain relieving interventions were not frequently applied when necessary; nurses' attitudes played an important role in decision-making about initiation of a pain treatment.

Correspondence address: Dr. Sandra Zwakhalen, Maastricht University, Contact Health Services Research, Duboisdomein 30, NL-6229 GT Maastrich, The Netherlands. Tel.: +31 043 38 8408; Fax: +31 043 38 8416; E-mail: s.zwakhalen@maastrichtuniversity.nl

### Lecture 3: Impact of pain treatment on cognitive deficits and behavioural disturbances in nursing home patients with moderate and severe dementia

Wilco Achterberg
*LUMC, RC Leiden, The Netherlands*

Pain is common amongst elderly due to the increased prevalence of age-related diseases like osteoporosis, arthritis and cardiovascular diseases. The prevalence of pain amongst elderly living in nursing homes varies around 50%. Patients with dementia seem to experience the intensity and affective component of pain different from patients with intact cognition. In addition, due to loss of language skills, pain is often not communicated as such in more severe stages of dementia. In these patients, pain is often expressed in specific behaviour, such as agitation or withdrawal. The presentation will point out this still prevalent disastrous situation, in which pain in dementia is mistaken as a psychiatric condition and treated consequently by psychotropic drugs. The combination of multiple morbidity and poly-pharmacy increases the chance of side-effects and complications. Meanwhile, there is some evidence that pharmacological and non-pharmacological pain interventions can be effective in these vulnerable elderly people. Not only are behavioral symptoms associated with pain, treatment of pain may indeed also decrease cognitive deficits and behavioural disturbances.

Correspondence address: Prof. Wilco Achterberg, LUMC, Postbus 9600, NL-2300 RC Leiden, The Netherlands. Tel.: +31 0715268412; E-mail: w.p.achterberg@lumc.nl

### Symposium 10: Neuropsychology and Neuroimaging of the bilingual brain

Marco Calabria and Alber Costa
*Center for Brain and Cognition, Pompeu Fabra University, Department of Technology and Communications, Barcelona, Spain*

Nowadays more than half of the world's population is bilingual and the number of people speaking more than one language is increasing. Therefore, the study of the 'bilingual brain' has become a relevant topic in the fields of neuroimaging and neuropsychology. The aim of this symposium is to bring together researchers who illustrate the neural aspects (structural and functional one) of language processing in bilinguals and the neural changes associated to the benefits of bilingualism. The five papers presented here cover different research fields of bilingualism using different approaches: from neuroimaging to clinical neuropsychology. Anna J. Simmonds will focus on cortical and subcortical functions associated with non-native vocal learning in English participants using functional magnetic resonance imaging (fMRI). Jubin Abutalebi will talk about the neurological basis for why bilinguals outperform monolinguals on many attentional control tasks, both in healthy individuals and in patients with cognitive decline. Brendan Weekes will present a review of published cases of bilingual aphasia by considering several variables explaining the language recovery patterns in bilingual speakers. Swathi Kiran will talk about three experiments examining the interaction between facilitation and interference in the context of bilingual aphasia rehabilitation and she will frame the results within the current models of bilingual production. Finally, Marco Calabria will present a case of pathological language switching due to neurodegenerative disease in a Catalan-Spanish bilingual individual.
Correspondence address: Mr PhD Marco Calabria, Center for Brain and Cognition – Pompeu Fabra University – Department of Technology and Communications, C. Tanger, 122-140 (room 55124), ES-08018 Barcelona, Spain. Tel.: +34 93 542 2629; E-mail: calabria.marc@gmail.com

**Lecture 1: The anterior striatum in non-native vocal learning**

Anna J. Simmonds[1], Robert Leech[1], Richard J.S. Wise[1] and Paul Iverson[2]
[1]*Imperial College, Division of Brain Sciences, Department of Medicine, London, UK*
[2]*University College London, Division of Psychology and Language Sciences, London, UK*

We investigated cortical and subcortical function associated with human vocal learning. Adult monolingual native English participants were scanned using functional magnetic resonance imaging (fMRI) while repeating non-native (Spanish, German and Mandarin) bi-syllabic words, with no feedback received. They then underwent training on the pronunciation of half the words for one week, before a second fMRI scanning session. The baseline condition was repetition of bi-syllabic native non-words: novel word forms comprised of familiar (English) phonemes. In the anterior striatum there was a Language (non-native words and native non-words) X Session (before and after training) interaction. Both trained and untrained non-native words showed a between-session decline in activity, even though improved accuracy of pronunciation only occurred with the former. Even within the first session, striatal activity for repeating non-native words declined after the first of three runs. In contrast, activity in sensory perisylvian cortex when repeating non-native words correlated negatively with improved accuracy and an increased signal-to-noise ratio (SNR); notably in a left high-order auditory area for Mandarin tones and in left ventral somatosensory cortex for German vowels, which depend on novel tongue movements. The response of the anterior striatum, unrelated to measures of accuracy of non-native word repetition, indicates its involvement is not related solely to activity in premotor and motor cortico-striatal loops, but is modulated by additional processes, which may be as diverse as motivation and self-perception of performance.

Correspondence address: Mrs PhD Anna J. Simmonds, Imperial College – Division of Brain Sciences, Department of Medicine, South Kensington Campus, UK-SW7 2AZ London, UK. E-mail: anna.simmonds08@imperial.ac.uk

**Lecture 2: Bilingualism across lifespan: Structural and functional brain consequences**

Jubin Abutalebi
*University San Raffaele Milan, Italy*

Culture, education and of other forms of acquired capacities act on individual differences in skill to shape how individuals perform cognitive tasks such as attentional and executive control. Of interest, bilingualism also appears to be a factor that shapes individual performance on tests of cognitive functioning. Indeed, researchers have shown that a bilingual can have better attention and executive control capacities than monolingual speakers and this is argued to be due the ability to inhibit one language while using another. Beyond behavioural differences, bilingualism seems to affect brain structure as well. During my presentation I will show how bilinguals develop more grey matter in crucial brain areas responsible for executive control, hence, providing a neurological basis for why bilinguals outperform monolinguals on many attentional control tasks. Furthermore, it has been postulated that this cognitive advantage offers protection to bilinguals against cognitive decline (dementia) in aging. Bilingualism affords a cognitive reserve (CR) in the form of a set of skills that allows some people to cope with cognitive decline such as mild cognitive decline (MCI) or Alzheimer's disease (AD) better than others. The primary aim of this presentation is to illustrate how the bilingual brain becomes resistant to cognitive decline. New structural and functional neuroimaging results from comparative studies between bilinguals and monolinguals during lifespan (i.e., covering children, adults and seniors) will be illustrated in order to provide an overview of how bilingualism induces a neurocognitive advantage and how this advantage offers protection against cognitive decline.

Correspondence address: Mr MD Jubin Abutalebi, University San Raffaele Milan, Via Olgettina, 60, I-20132 Milan, Italy. E-mail: abutalebi.jubin@hsr.it

### Lecture 3: Bilingual aphasia: A cognitive neuropsychological account

Brendan Weekes and Ekaterina Kuzmina
*Faculty of Education, University of Hong Kong, Speech and Hearing Sciences, Hong Kong, China*

Studying cases of bilingual speakers with aphasia allows tests of hypotheses derived from models of bilingual language processing by investigating the importance of endogenous variables (language status and dominance, mode of language acquisition, language proficiency and preference) relative to exogenous factors (language type, word frequency, concreteness, cognate status and grammatical class) in bilingual language processing. We present a review of published cases of bilingual aphasia including reports that are not published in English. Reports were analysed according to three dimensions: age of acquisition of the second language (L2), language dominance and linguistic distance between the spoken languages (e.g. Indo-European vs. Sino-Tibetan). Analyses found that approximately 50% of patients show a pattern of differential impairment whereby the first acquired language is better preserved relative to other languages spoken with 25% showing the opposite pattern. In all the other cases, there was no differential impairment. When the cases are grouped according to linguistic distance between spoken languages, the pattern of differential impairment was similar i.e. the majority of cases have more impairment in the later acquired language. For those patients with greater impairment in the first acquired language, L2 is the dominant language in daily life for all cases. One finding from the review is differential effects of task on performance i.e. impairment is greater in L2 on some tasks and in L1 for others. We argue all effects can be understood with reference to a computational model of bilingual language processing first proposed by Dijkstra and colleagues (2001) that assumes a shared architecture for all languages. Furthermore, we show that effects of psycholinguistic variables such as word class (nouns versus verbs), age of acquisition and imageability on performance can be explained with reference to a language independent cognitive framework.

Correspondence address: Mr Professor Brendan Weekes, Faculty of Education, University of Hong Kong, Speech and Hearing Sciences, Hospital Road, 34, CHN-999077 Hong Kong, China. E-mail: weekes @hku.hk

### Lecture 4: The nature of facilitation and interference in the rehabilitation of bilingual aphasia

Swathi Kiran, Teresa Gray, Mel Lo and Caitlin Keane
*Boston University, Speech Language and Hearing Sciences, Boston, US*

I will talk about three experiments examining the interaction between facilitation and interference in the context of bilingual aphasia rehabilitation. The first experiment investigated the nature of within- and between-language generalization in 17 participants with Spanish/English bilingual aphasia who received semantic naming treatment. Participants received therapy in either English or Spanish and treatment outcomes were compared for: trained items, within-language semantically related untrained items, between language translations of trained and between-language translations of untrained semantically related items. Treatment for naming on Set 1 items resulted in significant improvement on the trained items in 14/17 participants. Within-language generalization to semantically related items was observed for 10 participants. Between-language generalization to the translations of trained items was observed for 5 participants; whereas between-language generalization to the translations of the untrained semantically related items for 6 participants. In other study, a Chinese-English bilingual individual with aphasia was trained in Chinese and showed between-language generalization to the untrained English language. In a third study, a French-English-Amharic trilingual individual with aphasia, was trained first in French and then in English. This individual showed no within or between-language generalization and showed several instances of cross-language interference. Results of these studies illustrate that within- and between-language patterns are variable across participants and these differences are indicative of the interplay between facilitation (generalization) and inhibition. The discussion addresses these results within current theoretical models of lexical access and the influence of rehabilitation on these models. Rehabilitation outcomes also appear to be influenced by language proficiency, use, and the patient's current language environment.

Correspondence address: PhD Swathi Kiran, Boston University, Speech Language and Hearing Sciences, Commonwealth Aveneu, 635, US-02101 Boston, US. E-mail: kirans@bu.edu

### Lecture 5: Lost in translation: A case-study of pathological language switching

Marco Calabria[1], Paula Marne[1], Lucía Romero-Pinel[2], Montserrat Juncadella[2] and Alber Costa[1]
[1]*Center for Brain and Cognition, Pompeu Fabra University, Department of Technology and Communications, Barcelona, Spain*
[2]*Hospital Universitari de Bellvitge, Barcelona, Spain*

Bilingual speakers are usually quite good at restricting their lexicalization output to the desired language while preventing all sorts of language intrusions from the language not in use. However, brain damage can affect these abilities of language control, leading to striking and flagrant linguistic behaviors, such as pathological language mixing (pLS) and switching (pLM).
In this paper we report the performance a Catalan-Spanish bilingual individual (RRT) who, due to a neurodegenerative disease and subcortical lesions, shows pLS. We tested RRT in several tasks of language production and control, such as: picture naming (objects and actions), word translation, blocked naming and language switching task.
We found several interesting results. First, cross-language intrusions were present much more frequently when the RRT was asked to speak in her first (and dominant) language (Catalan) than when she was asked to do so in the non-dominant language (Spanish). Second, the results provide evidence suggesting that damage to certain subcortical structures may lead to problems in controlling the language output during verbalization in bilingual speakers. Finally, we observed that RRT seemed to show more difficulties in language control with verbs. The results are discussed in relation to other findings of pLM and pLS in published single-case reports.

Correspondence address: PhD Marco Calabria, Center for Brain and Cognition, Pompeu Fabra University, Department of Technology and Communications, C. Tanger, 122-140 (room 55124), ES-08018 Barcelona, Spain. Tel.: +34 93 542 2629; E-mail: calabria.marc@gmail.com

### Symposium 11: Neuropsychological impairments and driving performance

Alexander Brunnauer[1] and Hendrik Niemann[2]
[1]*kbo-Inn-Salzach-Klinikum, Neuropsychology, WasserBurg, Germany*
[2]*NRZ Neurologisches Rehabilitationszentrum Leipzig, Bennewitz*

For most people, driving is an integral part of their mobility and independence affecting physical, social and economic well-being. Thus professionals who are concerned with the quality of life of their patients are inevitably confronted with issues of fitness to drive. A comprehensive assessment and counseling of patients with neuropsychological impairments is of great relevance in clinical practice. In our symposium, we will focus on various topics of fitness to drive of patients with neuropsychological dysfunction. From a methodological point of view, there is a great debate whether laboratory or clinical tests can reliably predict on-road driving performance. Niemann and Hartje focus on this topic. A new approach in assessing fitness to drive is the use of driving simulators. Kenntner-Mabiala and co-workers present new simulation techniques for testing and training fitness to drive of patients with neuropsychological impairments. The demographic change in western industrial nations points to another field of importance. Especially for the elderly, mobility is essential. Much less is known about skills that are relevant for safe driving in older drivers (Köster and colleagues). Last but not least, psychopharmacological treatment is common in patients with a neuropsychiatric disorder. Among psychotropics, antidepressants are the most frequent ones prescribed. Brunnauer et al. present data of the effects of newer antidepressants on driving performance in patients with depressive symptoms.

Correspondence address: Dr. Alexander Brunnauer, kbo-Inn-Salzach-Klinikum, Neuropsychology, Haus 13, D-83512 Wasserburg, Germany. Tel.: +49 8071 71224; Fax: +49 8071 743; E-mail: alexander.brunnauer@iskl.de

### Lecture 1: Neuropsychological assessment of fitness to drive and on-road driving tests in the rehabilitation of patients with brain damage

Hendrik Niemann[1] and Wolfgang Hartje[2]
[1]*NRZ Neurologisches Rehabilitationszentrum Leipzig, Neuropsychology, Bennewitz, Germany*
[2]*NRZ Rehabilitationszentrum Leipzig, München*

Abstract: The data of 494 brain-damaged patients whose driving ability was examined during inpatient rehabilitation were retrospectively analysed. The sample was comprised of 394 males (mean age 51.0 yrs.) and 100 females (mean age 43.1 yrs.). Time since injury ranged from one month to several years. Most patients suffered from cerebrovascular accidents (49%) or traumatic brain injury (21%). The neuropsychological tests included measures of alertness, selective and divided attention, flexibility, and visual scanning. Patients, who did not meet the criteria of neurocognitive functioning based on German guidelines of fitness to drive, participated in a semi-standardized on-road driving test. Various aspects of driving behaviour and overall safety were rated by the driving instructor and a neuropsychologist. 28% of the patients failed the driving test. Discriminant function analyses based on neuropsychological test scores resulted in hit rates between 76% and 77% depending on variables included. Positive predictive values ranged from 64% to 69%, and negative predictive values ranged from 78% to 80%. Of 14 test scores, the following subtests of the Test of Attentional Performance (TAP) were selected for best discriminant function: Alertness (simple visual reaction time) and Visual Scanning (reaction time and omissions). In addition, the Trail Making Test Part A was also included. Test scores and aspects of driving behaviour loaded on separate orthogonal factors. The outcome of the on-road driving test could not be reliably predicted from the psychometric measures.

Correspondence address: Dr. Hendrik Niemann, NRZ Neurologisches Rehabilitationszentrum Leipzig, Neuropsychology, Muldentalweg 1, D-0428 Bennewitz, Germany. E-mail: niemann@sachsenklinik.de.de

### Lecture 2: New solutions for testing and enhancing fitness to drive of neuropsychological patients with driving simulation:

Ramona Kenntner-Mabiala, Yvonne Kaussner, Sonja Hoffmann and Alexandra Neukum
*WIVW Würzburger Institut für Verkehrswissenschaften, Veitshöchheim, Germany*

Due to the growing need for mobility in our ageing society, testing and enhancing fitness to drive of patients suffering from neuropsychological conditions becomes more and more important. Modern driving simulators combine the advantages of classical psychometric test batteries and on road drives and solve the shortcomings. Driving scenarios for testing and training can be systematically designed, presented and reproduced. As in real traffic, subjects can practice compensatory strategies. Neither subjects nor other road users are compromised. Due to high face-validity simulation is well-accepted by subjects. However, some methodological issues have to be specifically addressed when using simulators. Firstly, the test scenarios should be both representative for real driving and sensitive to the condition to be investigated. Furthermore, driving performance should be assessed as a whole by various endpoints that refer to the operational and tactical level of the driving task. Finally, the simulated driving test has to be validated. The software package "Driver Fitness and Ability" offers test scenarios that are representative for real driving, alcohol-validated and sensitive for psychotropic substances and neuropsychological conditions. Out of more than 50 scenarios, adaptive training sessions as well as test courses can be compiled comfortably via drag and drop. The assessment of fitness to drive is conducted holistic and semi-automatic by means of a tablet computer. The scenario package can be run on simple PC-simulations as well as on high-end solutions with motion system. A detailed demonstration of the scenarios and the procedure of assessing driving ability will be given in the presentation.

Correspondence address: Mrs Dr Ramona Kenntner-Mabiala, WIVW Würzburger Institut für Verkehrswissenschaften, Raiffeisenstraße 17, D-97209 Veitshöchheim, Germany. E-mail: kenntner@wivw.de

### Lecture 3: Abilities relevant for safe driving in healthy older drivers

Nina Köster, Tatjana Edelmann, Mehmet Gövercin and Elisabeth Steinhagen-Thiessen
*Forschungsgruppe Geriatrie der Charite, Universitätsmedizin Berlin, Germany*

Driving places is a highly complex and demanding task. Abilities essential for safe driving change due to normal aging, age-associated diseases, and medication. However, driving is an important aspect of mobility in old age, as it helps maintaining independent living and hence a higher quality of life. So far there is no consensus on which skills are most relevant, how exactly these abilities change, how they interact, and how deficits can be compensated for. This is also true for assessment tools. The DrivingHealth® Inventory (DHI)

is a computerized screening of visual, motor, and cognitive abilities associated with an increased crash risk. The objective of this study, conducted within the collaborative project SmartSenior was to collect normative data on senior drivers' abilities relevant for safe driving. Since there is also a controversy regarding seniors' ability to adequately assess their own driving performance, a secondary objective was to assess driving behavior and attitudes.

A German version of the DHI was completed by 116 healthy senior drivers (M = 67.7 years old, SD 3.23, 67.2% male, 67.2% with high level of education). A comprehensive medical and neuropsychological examination, including the Test of Attentional Performance – Mobility Version were also administered. Participants completed simulator drives in which they were confronted with unexpected hazard events. They also filled in questionnaires asking about their driving behavior, sense of security driving, attitudes towards regulations on driving in old age, and crash involvement.

According to the DHI results, 77.9% of participants had at least one deficit, 18.3% in two or more tasks. Most prevalent were difficulties concerning cognitive flexibility. 25% could not turn their head around to look over their shoulder. Variability of simulator performance was low. Simulator performance was lowly associated with assessment results.

Correspondence address: Mrs Nina Köster, Forschungsgruppe Geriatrie der Charite, Universitätsmedizin Berlin, Reinickendorferstraße 61, D-13347 Berlin, Germany. E-mail: nina.koester@charite.de

### Lecture 4: Driving simulator performance and psychomotor functions under newer antidepressants

Alexander Brunnauer[1], Gerd Laux[2] and Verena Buschert[1]

[1]*kbo-Inn-Salzach-Klinikum, Neuropsychology, Wasserburg, Germany*

[2]*kbo-Inn-Salzach-Klinikum, Wasserburg*

OBJECTIVES: Driving a car is vital for the functional autonomy of patients to take part in activities of daily living. Although the therapeutic efficacy of newer antidepressants has been demonstrated in various studies there is only little research available about patients' fitness to drive under newer antidepressants.

METHODS: A sample of 75 depressive inpatients diagnosed according to DSM-IV criteria were randomly assigned to treatment with either mirtazapine ($n = 25$), escitalopram ($n = 25$) or reboxetine ($n = 25$). Participants were tested before pharmacologic treatment (t0), and on days 7 (t1) and 14 (t2) with computerized tests related to car driving skills. Data were collected with the Act and React Testsystem (ART 90) and the Wiener-Testsystem (WTS) measuring visual perception, reactivity, stress tolerance, concentration and vigilance. Besides patients underwent various risk simulations on a static driving simulator.

RESULTS: Patients showed significant improvements in most functional domains related to driving ability skills and also in driving simulator performance after 14 days of treatment with newer antidepressants. Especially in concentration, reactivity and stress-tolerance a clear advantage could be demonstrated in contrast to the untreated phase. Statistically significant differences between treatment groups could not be shown.

CONCLUSIONS: Partly remitted depressive patients treated with mirtazapine, escitalopram or reboxetine showed a better test performance in driving related skills than untreated patients. Especially in the case of newer, selective antidepressant results have important implications for risk calculations within legal requirements.

Correspondence address: Dr. Alexander Brunnauer, kbo-Inn-Salzach-Klinikum, Neuropsychology, Haus 13, D-83512 Wasserburg, Germany. Tel.: +49 8071 71224; Fax: +49 8071 743; E-mail: alexander.brunnauer@iskl.de

### Symposium 12: To the left and to the right: How non-invasive brain stimulation techniques influence motor and cognitive functions

To the Left and to the Right: How Non-invasive Brain Stimulation Techniques influence motor and cognitive functions

Paola Marangolo[1], Agnes Floel[2], Friederich Hummel[3] and Giacomo Koch[4]

[1]*University Politecnica delle Marche, of Experimental and Clinical Medicine, Ancona, Italy*

[2]*Charité, Department of Neurology, Universitatsmedizin Berlin, Berlin, Germany*

[3]*Department of Neurology, University Medical Center Hamburg-Eppendorf, Hamburg, Germany*

[4]*University Hospital Tor Vergata, Neurology, Rome, Italy*

The past decade of neuroscience research has provided considerable evidence that the adult brain can undergo substantial reorganization following a stroke [1]. A growing body of evidence have already indicated that noninvasive brain stimulation techniques (NIBS), such as Transcranial magnetic stimulation (TMS) and transcranial direct current stimulation (tDCS), can modulate brain plasticity in the healthy and brain-damaged populations [2,3]. These methods have shown promising results in promoting functional recovery of the most disabling conditions after stroke such as motor impairment, aphasia and neglect [4,5,6]. However, there are still doubts regarding the areas to be stimulated in the context of compensatory activation and maladaptive plasticity in both the affected and unaffected hemispheres. Moreover, it is still an open issue if the effects found are persistent overtime and are specific for the treated function or generalize to other domains. This symposium will directly address these crucial issues reporting the state of the art in the most important neurological and cognitive domains in which NIBS is becoming a useful promising clinical tool.

References

[1] Dancause N, Nudo RJ. Shaping plasticity to enhance recovery after injury. Prog Brain Res, 2011.
[2] Floel, A. et al. Non invasive brain stimulation improves language learning. J. Cogn. Neurosci, 2008.
[3] Fiori, V. et al. Transcranial direct current stimulation improves word retrieval in healthy and nonfluent aphasic subjects. J. Cogn. Neurosci, 2011.
[4] Schulz R. et al (2013) Non-invasive brain stimulation in neurological diseases. Neuropharmacology, 2013
[5] Marangolo, P. et al. Differential involvement of the left frontal and temporal regions in verb naming: a tDCS treatment study. Restor. Neurol. Neurosci, 2013.
[6] Koch G et al. $\theta$-burst stimulation of the left hemisphere accelerates recovery of hemispatial neglect. Neurology, 2012.

Correspondence address: Mrs Professor Paola Marangolo, University Politecnica delle Marche – of Experimental and Clinical Medicine, Via Tronto 10A, 60020 Ancona, Italy. E-mail: p.marangolo@univpm.it

### Lecture 1: tDCS-enhances language recovery – new challenges in aphasia rehabilitation?

Paola Marangolo
*University Politecnica delle Marche, of Experimental and Clinical Medicine, Ancon, Italy*

Recently, the progress of new technologies have made new tools available for professional therapists. In the field of aphasia, one area is central for a positive outcome in language rehabilitation: the use of noninvasive brain stimulation techniques. A growing body of evidence have already indicated that TMS and tDCS can have beneficial effects in the treatment of aphasia [1,2]. However, some studies, although have shown an improvement of lexical deficits, do not always confirm a persistency of the effects found [3]. Moreover, many of these studies do not have a control condition to establish the specificity of the stimulated area [3]. More recent studies have suggest that long-term effects might be more easily obtained with repeated stimulations and during simultaneous specific language training [4,5,6]. Here, the development of these innovative approaches as a potentially promising tools for aphasia rehabilitation will be discussed together with an overview of the language deficits more suitable for these interventions.

References

[1] Naesser MA et al., Improved picture naming in chronic aphasia after TMS to part of right Broca's area: an open-protocol study. Brain Lang, 2005.
[2] Fiori, V. et al. Transcranial direct current stimulation improves word retrieval in healthy and nonfluent aphasic subjects. J. Cogn. Neurosci, 2011.
[3] Monti A. et al. Transcranial direct current stimulation (tDCS) and language. J. Neurol. Neurosurg. Psychiatry, 2012.
[4] Fridrikson J et al. Transcranial direct current stimulation improves naming reaction time in fluent aphasia: a double-blind, sham-controlled study. Stroke, 2011.
[5] Marangolo P et al. Electrical stimulation over the left inferior frontal gyrus (IFG) determines long-term effects in the recovery of speech apraxia in three chronic aphasics. Behav Brain Res, 2011.
[6] Marangolo P et al., Differential involvement of the left frontal and temporal regions in verb naming: a tDCS study. Restor Neurol and Neurosci, 2013

Correspondence address: Professor Paola Marangolo, University Politecnica delle Marche – of Experimental and Clinical Medicine, Via Tronto 10A, 60020 Ancon, Italy. E-mail: p.marangolo@univpm.it

### Lecture 2: tDCS-enhanced language training – proof- of- principle and neural correlates

Agnes Floel
*Charité, Universitatsmedizin Berlin, Neurology, Berlin, Germany*

The rising proportion of elderly people worldwide will yield an increased incidence of age-associated cognitive impairments and dementia. Consequently, growing interest emerged to evaluate new strategies to delay or counteract cognitive decline in aging. Here, I will discuss a series of studies aimed to assess the impact of anodal transcranial direct current stimulation (atDCS) on learning ability in young and older adults. In a study, atDCS versus sham over left temporoparietal areas enhanced learning of a novel vocabulary [1], while learning of an artificial grammar was enhanced by atDCS over Broca's area [2]. We then went on to test task-related activity of a semantic fluency paradigm and resting-state functional connectivity during atDCS over the ventral part of left IFG [3]. Improved behavioral performance was associated with selectively reduced task-related activation in the left ventral IFG and increased connectivity of the left IFG. In a group of healthy older adults improved behavioral performance and increased network efficiency and specificity was found4. These results have important clinical implications as they suggest the possibility to apply these protocols to elderly people to delay their cognitive decline.

References

[1] Floel A et al. Noninvasive brain stimulation improves language learning. J Cogn Neurosci. 2008; 20: 1415-1422.
[2] de Vries MH et al. Electrical stimulation of Broca's area enhances implicit learning of an artificial grammar. J Cogn Neurosci. 2010; 22: 2427-2436.
[3] Meinzer M et al. Electrical brain stimulation improves cognitive performance by modulating functional connectivity and task-specific activation. J Neurosci. 2012; 32: 1859-1866.
[4] Lindenberg R et al. Age-related effects of non-invasive inferior frontal cortex stimulation on rest- ing-state connectivity. Organization for Human Brain Mapping, 2013.

Correspondence address: Mrs Professor Agnes Floel, Chariteé – Universitatsmedizin Berlin, Neurology, Chariteéplatz 1, D-10117 Berlin, Germany. E-mail: Agnes.Floeel@charite.de

### Lecture 3: Non-invasive brain stimulation to support neuroregeneration of the motor system after stroke

Friederich Hummel
*University Medical Center Hamburg-Eppendorf, Neurology, Hamburg, Germany*

Non-invasive brain stimulation has provided evidence for its potential to modulate brain plasticity in humans [1]. Based on these findings brain stimulation has been used in neurological diseases to enhance neuroplasticity, adaptive processes and to prevent potential maladaptive ones to support functional recovery [2]. In stroke for instance sensorimotor and higher cognitive impairment, such as aphasia and neglect, has been addressed to facilitate functional recovery. In the present presentation an update of the field of transcranial magnetic stimulation (TMS) and transcranial direct current stimulation (tDCS) as non-invasive brain stimulation techniques to improve motor functions in patients suffering from focal brain lesions will be provided. Rather than attempting to be comprehensive in regard of the reviewed scientific field, this presentation may be considered as a present day's framework of the application of non-invasive brain stimulation on selected examples of common neurological diseases. At the end open controversies and future directions of the field which has to be addressed in upcoming studies will be briefly discussed [3].

References

[1] Hummel, F.C. & Cohen, L.G. Drivers of brain plasticity. Curr Opin Neurol 18, 667-674 (2005).
[2] Hummel, F.C. & Cohen, L.G. Non-invasive brain stimulation: A new strategy to improve neurorehabilitation after stroke? Lancet Neurol 5, 708-712 (2006).
[3] Hummel, F.C., et al. Controversy: Noninvasive and invasive cortical stimulation show efficacy in treating stroke patients. Brain Stimul 1, 370-382 (2008).

Correspondence address: Mr Doctor Friederich Hummel, University Medical Center Hamburg-Eppendorf, Neurology, Martinistraße 52, 20251 Hamburg, Germany. E-mail: f.hummel@uke.uni-hamburg.de

**Lecture 4: The hyperexcitability of the left intact hemisphere: non-invasive brain stimulation model of the unaffected hemisphere in neglect patients**

Giacomo Koch
*University Hospital Tor Vergata, Neurology, Rome, Italy*

The human brain is characterized by the lateralization of cognitive functions. Multiple lines of evidence suggest that the deployment of visuo-spatial attention is controlled by a fronto-parietal network, with right hemisphere dominance. Among cortical areas included in the network, the right posterior parietal cortex (PPC) has been proposed to be a crucial node and has also been implicated on clinical grounds. Here, I will provide an overview of the existent literature giving evidence to a functional asymmetry of the parietal cortices in directing visuo-spatial attention, focusing on those studies seeking to characterize the causal role of PPC, applying transcranial magnetic stimulation (TMS) and its combination with imaging techniques, such as EEG and fMRI [1,2]. I will then discuss studies involving neglect patients shedding light on the complex interplay between left and right PPC, strongly supporting the hemispheric-rivalry theory [3]. These evidence have important clinical implications as shown by recent findings demonstrating changes of neglect disorders following the non-invasive stimulation of the unaffected hemisphere.

References

[1] Koch G. et al. To the other side of the neglected brain: The Hyperexcitability of the left intact hemisphere, Neuroscientist, 2012.
[2] Koch et al., $\theta$ – burst stimulation of the left hemisphere accelerates recovery of hemispatial neglect. Neurology, 2012.
[3] Koch et al. Asymmetry of parietal interhemipheric connections in humans. J. Neuroscience, 2011

Correspondence address: Mr Doctor Giacomo Koch, University Hospital Tor Vergata, Neurology, Viale Oxford 81, 00133 Rome, Italy. E-mail: g.koch@hsantalucia.it

**Symposium 13: The Contributions of the prefrontal cortex to episodic memory**

Sarah E. MacPherson
*University of Edinburgh, Department of Psychology, Edinburgh, United Kingdom*

It is widely accepted that the frontal lobes play an important role in memory. Frontal lobe lesions have been found to produce impairments on both verbal and visual memory tests including free recall, memory for contextual information, temporal order memory and metamemory. Yet, some of the specific contributions of the prefrontal cortex to episodic memory still remain a matter of debate, such as whether prefrontal lesions result in both recall and recognition memory impairments and the role of anatomical frontal subregions on episodic memory. Therefore, the aim of this symposium is to present some of the recent research investigating the prefrontal lobes and episodic memory. Presentations will comprise studies comparing the role of the prefrontal cortex in recall and recognition, including findings which demonstrate that inhibition of the right dorsolateral prefrontal cortex modulates a domain general memory retrieval process. We will also present the results of work investigating the involvement of the ventromedial and orbitofrontal prefrontal regions on spontaneous confabulation and mental time travel into the past and the future. Finally, we will discuss some recent neuroimaging data which suggests that the medial prefrontal cortex supports the ability to make reality monitoring decisions. These presentations will provide an overview of the current state of the frontal lobes and memory literature.

Correspondence address: Dr. Sarah E. MacPherson, University of Edinburgh, Department of Psychology, 7 George Square, UK-EH15 2QN Edinburgh, United Kingdom. Tel.: +44 131 650 9862; Fax: +44 131 651 3230; E-mail: sarah.macpherson@ed.ac.uk

**Lecture 1: The Doors and People Test: The effect of frontal lobe lesions on recall and recognition memory performance**

Sarah E. MacPherson[1], Martha S. Turner[2], Marco Bozzali[3], Lisa Cipolotti[4] and Tim Shallice[5]
[1]*University of Edinburgh, Department of Psychology, Edinburgh, United Kingdom*
[2]*University College London, Institute of Cognitive*

*Neuroscience, London, United Kingdom*
[3]*Santa Lucia Foundation, IRCCS, Neuroimaging Laboratory, Rome, Italy*
[4]*National Hospital for Neurology and Neurosurgery, Department of Neuropsychology, London, United Kingdom*
[5]*SISSA, Cognitive Neuroscience Sector, Trieste, Italy*

Memory deficits in frontal patients are most apparent on recall tasks that require the selection, initiation, and implementation of strategies, while recognition memory impairments are less well reported. However, few studies have compared recall and recognition within the same frontal group and those that have report inconsistent findings. The Doors and People Test was devised to directly compare recall and recognition performance within the same test, and yet, no studies have investigated frontal patients' performance on this memory battery. In the current study, 47 patients with frontal lobe lesions due to tumour or stroke and 78 healthy controls performed the verbal and visual recall and recognition subtests from the Doors and People Test. The results demonstrated that the frontal group was significantly impaired on the verbal and visual recall subtests as well as the verbal recognition subtest compared to healthy controls. The comparison between frontal patients' and healthy controls' performance on the visual recognition subtest approached significance. The effect sizes for each subtest showed that the magnitude of the difference between the frontal and control groups did not significantly differ between verbal recall and recognition or visual recall and recognition. Therefore, when recall and recognition memory tests are matched in terms of difficulty, they do not differ in their ability to identify frontal patients.

Correspondence address: Dr Sarah E. MacPherson, University of Edinburgh, Department of Psychology, 7 George Square, UK-EH15 2QN Edinburgh, United Kingdom. Tel.: +44 131 650 9862; Fax: +44 131 651 3230; E-mail: sarah.macpherson@ed.ac.uk

### Lecture 2: The contribution of the PFC to recognition memory: Evidence from TMS studies

Lisa Cipolotti
*National Hospital for Neurology and Neurosurgery, Department of Neuropsychology, London, United Kingdom*

This study investigated the effect of repetitive transcranial magnetic stimulation and inhibitory theta burst stimulation on recognition memory performance of healthy subject, MCI and AD patients. We applied magnetic stimulation over left and right dorsolateral prefrontal cortex (DLPFC) at retrieval in recognition memory tests. We found that rTMS applied to the right DLPFC improved recognition memory performance in healthy controls (HC). In contrast rTMS applied to the left DLPFC had no effect on the HC's recognition memory performance. Interestingly, iTBS excitation of the right DLPFC of HC impaired recognition memory performance whilst iTBS excitation of the left DLPFC had no effect on HC's recognition memory performance. Interestingly, rTMS inhibition of the right DLPFC also improved the recognition memory performance of MCI and mild Alzheimer's Disease. In contrast, and similarly to the healthy control, the recognition memory performance of the MCI and mild AD patients did not improve following inhibitory rTMS over the left DLPFC. rTMS of the right or left DLPFC have no effect on the recognition memory performance of moderate Alzheimer's Disease patients. These findings suggest that the inhibition of the right dorsolateral prefrontal cortex modulates a domain general memory retrieval process. Moreover, it suggests that rTMS may represent a promising therapeutic tool for memory impairment.

### Lecture 3: Confabulation and the frontal lobes

Michael D. Kopelman
*King's College London, Institute of Psychiatry, London, United Kingdom*

This talk will briefly review the evidence that spontaneous confabulation is related to ventro-medial frontal and orbito-frontal lobe damage, as well as current theories of confabulation. It will report new findings examining Schnider's theory of confabulation, and also the role of affective and motivational factors in confabulation. Twenty-four confabulating brain-injured patients were compared with 11 non-confabulating brain-injured patients and 6 healthy controls with follow-up over 9 months. Findings will be reported, and their implications for current theories discussed.

### Lecture 4: Mental time travel following lesion to the ventromedial prefrontal cortex

Elisa Ciaramelli, Chiara Tesini and Elena Bertossi
*University of Bologna, Department of Psychology and Centre for Studies and Research in Cognitive Neuroscience, Cesena, Italy*

Functional neuroimaging studies have suggested that both remembering the past and imagining the future activate a 'default' network of brain regions including the ventromedial prefrontal cortex (VMPFC). Although patients with damage to the prefrontal cortex are often described as impulsive, "blind to the future consequences of their choices", and "locked into immediate space and time", it is unclear whether and how the VMPFC mediates mental time travel into the past and the future. Six patients with lesion to the VMPFC (VMPFC patients) and 12 healthy controls remembered past events and imagined future events in response to cue words. Event transcriptions were segmented into distinct details, and classified as either internal (episodic) or external (non-episodic) according to the scoring method of the Autobiographical Interview. Preliminary results show that VMPFC patients exhibit deficits in both remembering past events and imagining future events, generating fewer internal details than healthy controls. In contrast, external details were comparable between patients and controls. These results indicate that the VMPFC is a critical node of a system allowing transcending the present to re- or pre-experience alternative scenarios.

### Lecture 5: Getting a grip on reality: The role of medial prefrontal cortex in source recollection

Jon Simons
*University of Cambridge, Department of Psychology, Cambridge, United Kingdom*

Much previous research has characterised the involvement of lateral prefrontal cortex in recollecting the context in which previous events were experienced. In this talk, I will present evidence addressing the role that medial prefrontal cortex might play in such source recollection. Our data suggest that regions of medial prefrontal cortex support the ability to make "reality monitoring" decisions, distinguishing information that was generated by internal cognitive functions such as thought and imagination from information that was derived from the outside world by perceptual processes. Healthy volunteers exhibit greater fMRI activity in this region when performing reality monitoring tasks than other tests of recollection, evidence that we have related both to clinical conditions in which the ability to distinguish reality from imagination appears to be disturbed, and to inter-subject variability in the general population. The medial prefrontal region identified overlaps closely with one of the areas that tends to be functionally disrupted in schizophrenia, and reduced activity in the region in healthy individuals is associated with the kinds of specific misattribution errors often seen in the disorder. I will discuss evidence suggesting that variability in medial prefrontal cortical folding patterns might provide a specific structural basis for these performance differences.

### Symposium 14: Psychotherapy after aquired brain injury

Cornelia Exner
*University of Leipzig, Institute of Psychology, Department of Clinical Psychology and Psychotherapy, Leipzig, Germany*

Acquired brain injury (ABI) often results in emotional and behavioural adjustment problems and is associated with an increased risk for comorbid mental disorders. Emotional and behavioural disturbances interfere with rehabilitation efforts and decrease chances of ABI survivors for independence, participation and wellbeing in everyday life. Many comprehensive neuropsychological programs today incorporate psychotherapeutic strategies and techniques to ameliorate psychological functioning after ABI. Psychotherapy techniques are mostly based on standard cognitive-behaviour therapy protocols. Although the use of these techniques seems plausible there is a need for controlled trials to evaluate the merits of additional psychotherapy components for rehabilitation outcomes.
Contributions in this symposium from four different European countries and thus four different health care systems will focus on the combination of neuropsychological and psychotherapeutic approaches in rehabilitation efforts after ABI. The symposium program will include an overview on key clinical issues relating to the use of additional psychotherapy techniques in neuropsychological rehabilitation with special emphasis on which patients will benefit from it and which modifications to standard psychotherapy practice are necessary. Four different controlled clinical trials will be presented that use psychotherapy within neuropsychological rehabilitation programs to reach individual treatment goals, to foster self-awareness and emotional adjustment and to reduce symptoms of depression and anxiety. Results will show that incorporating psychotherapy in neuropsychological services presents a valuable treatment options for persons with long-term emotional and behavioural adjustment problems after ABI. Methodological and clinical issues of these combined treatment approaches will be discussed.

Correspondence address: Prof. Dr. Cornelia Exner, University of Leipzig, Institute of Psychology, Department of Clinical Psychology and Psychotherapy, Seeburgstr. 14-20, D-04103 Leipzig, Germany. Tel.: +49 341 9735931; Fax: +49 341 9735939; E-mail: exnerc@uni-leipzig.de

### Lecture 1: The role of psychotherapy in long-term brain injury rehabilitation: key clinical issues

Rudi Coetzer
*North Wales Brain Injury Service, Betsi Cadwaladr University Health Board NHS Wales and Bangor University, Colwyn Bay, UK*

Over the past decades psychotherapy has increasingly been incorporated as an aspect of some multidisciplinary rehabilitation programmes for persons with acquired brain injury. An overview of a few key psychotherapy approaches currently used to augment long-term neuro-rehabilitation after acquired brain injury is provided. The design and development of rehabilitation services conducive to incorporating psychotherapy approaches are discussed. While some may be helped, not all persons with acquired brain injury benefit from psychotherapy as an additional approach to their rehabilitation. Clinical case vignettes are used to illustrate some of the obstacles encountered in practice. Specific emphasis is placed on potential modifications to clinical practice that may sometimes be used by the clinician working in this continually developing field within neuropsychological rehabilitation. Psychotherapy may be one of the few available treatment options for persons with long-term emotional adjustment difficulties after brain injury.

Correspondence address: Dr. Rudi Coetzer, North Wales Brain Injury Service, Betsi Cadwaladr University Health Board NHS Wales and Bangor University, Hesketh Road, LL29 8AY Colwyn Bay, UK. E-mail: Rudi.Coetzer@wales.nhs.uk

### Lecture 2: Does an integrative neuro-psychotherapy program foster the adjustment in depressed patients with an acquired brain injury?

Helene Hofer[1], Martin Grosse Holftforth[2], Franziska Stalder-Lüthy[3], Eveline Frischknecht[3], René Müri[4] and Hansjörg Znoj[3]
[1]*University Hospital of Bern, Department of Neurology, Bern, Switzerland*
[2]*University of Zürich, Department of Psychology, Zürich, Switzerland*
[3]*University of Bern, Department of Psychology, Bern, Switzerland*
[4]*University of Bern, Department of Neurology, Bern, Switzerland*

GOALS: The main goal of this study was to investigate whether an integration of neuropsychological and psychotherapeutic techniques offers benefits in the process of coping with the effects of an acquired brain injury.
METHOD: The study was conducted in an outpatient setting. Patients meet the diagnosis of an adjustment disorder. The patients were randomly assigned to two treatments. One treatment, the so-called standard neuro-psychological treatment based on conventional recommendations for neurorehabilitation. The second treatment, the integrative program, added techniques from cognitive behavior therapy, the clarification-oriented and emotion-focused psychotherapy in addition. Whereas the research question and design have remained unchanged, some adjustments became necessary. On the basis of these context factors and developments, the former standard neuropsychological treatment was modified.
RESULTS: In total, 25 patients were recruited into the study. Time since injury ranged between 4 and 80 months ($M = 17$). The mean value of the BDI of the pre-measurement was 20. The patients received an average of 20 therapy sessions. The results show large effects across all treatment groups on symptoms of depression, quality of life, therapy goals, and fatigue and a small to medium effect regarding acceptance of the consequences of the disability.
CONCLUSION: Neurorehabilitative interventions aim at actively helping patients to cope with their impaired functions and can be seen as instances of optimizing the patient's mastery efforts. However, a change factor that is not yet systematically represented in neurorehabilitative programs is motivational clarification including a systematic focus on the emotional aspects of coping with the adjustment. The findings of our study suggest that the integration of neurorehabilitation with psychotherapeutic interventions can foster the process of coping with the adjustment to the consequences of an acquired brain injury.

Correspondence address: Dr. Helene Hofer, University Hospital of Bern, Department of Neurology, Freiburgstrasse, CH-3010 Bern, Switzerland. E-mail: helene.hofer@insel.ch

### Lecture 3: A therapeutic approach to improve self-awareness in brain injury patients with executive dysfunction

Jacoba M. Spikman
*University of Groningen, Department of Clinical and Developmental Neuropsychology and University Medical Center Groningen, Department of Neurology, Groningen, The Netherlands*

OBJECTIVE: Executive dysfunction is a frequent and disabling consequence of acquired brain injury, impairing patient's abilities to function independently in daily life. Consequently, there is a need for effective neuropsychological rehabilitation interventions that improve executive functions in daily life. However, many patients with dysexecutive problems have impaired self-awareness, which is associated with poor compliance with rehabilitation. We have developed a multifaceted treatment protocol for executive dysfunction and proved it to be effective in an RCT. An important ingredient of this treatment protocol was the Information and Awareness module. This consisted of psychoeducation and awareness-exercises, aimed to enhance self-awareness and hence motivation for treatment.
METHOD: The effects of the Awareness training were investigated in an RCT, comparing the multifaceted treatment with a control computer treatment. A group of 70 patients with acquired brain injury were randomly assigned to either treatment, resulting in 35 patients in each condition. Patients filled in the Dysexecutive Questionnaire (DEX) pre- and posttreatment, as well as their proxies (DEX-p) and their therapists (DEX-t). The discrepancy between self and other ratings on the DEX is considered to be an indication of self-awareness. We hypothesized that increased self-awareness after treatment would express itself in a decrease of the difference between self and other ratings only for those patients who underwent the multifaceted treatment.
RESULTS: Repeated Measures analysis showed a significant decrease of the difference between the self and therapist rating only, for those patients who underwent the multifaceted treatment, including Awareness training.
CONCLUSION: We conclude that a combination of psychoeducation and awareness exercises can improve self-awareness in patients with acquired brain injury and executive dysfunction, indicated by ratings of professionals only.

Correspondence address: Prof. Dr. Jacoba M. Spikman, University of Groningen, Department of Clinical and Developmental Neuropsychology and University Medical Center Groningen, Department of Neurology, Poortweg 4, NL-9700 Groningen, The Netherlands. E-mail: J.M.Spikman@rug.nl

### Lecture 4: Restore4Stroke: Augmented cognitive behavioural therapy for post-stroke depression with or without anxiety

Joyce Kootker[1], Luciano Fasotti[2], Sascha MC Rasquin[3], Caroline M Van Heugten[4] and Alexander CH Geurts[5]
[1]*Radboud University Nijmegen Medical Centre, Department of Rehabilitation, Nijmegen Centre for Evidence Based Practice, Nijmegen, The Netherlands*
[2]*Radboud University Nijmegen, Donders Institute for Brain, Cognition and Behaviour, Nijmegen, The Netherlands*
[3]*Adelante Rehabilitation Foundation Limburg, The Netherlands*
[4]*Maastricht University, Faculty of Health, Medicine and Life Sciences, Maastricht, The Netherlands*
[5]*Radboud University Nijmegen Medical Centre, Department of Rehabilitation, Nijmegen, The Netherlands*

Depression and anxiety are common after stroke. Their prevalence varies, but in general 25 percent of all stroke survivors experience depression with or without anxiety. Currently, there are no evidence-based treatments available. In a pilot study, Rasquin (2011)

showed that cognitive behavioural therapy positively influences depression complaints. Restore 4 stroke designed a multicentre randomized controlled trial to investigate the effectiveness of a similar treatment. We intend to include minimally $N = 53$. The treatment program was derived from a former depression protocol (Cuijpers, 2004), and specifically adapted for stroke survivors. The protocol consists of 5 stages that coincide with the motivational phases from the transtheoretical model of behavior change (Prochaska & DiClemente, 1998). The stages consist of building rapport; optimizing activity levels; recognizing negative thoughts and changing cognitions; consolidating and relapse prevention. The treatment is given by both psychologists and occupational therapists and consists of 13 to 16 sessions within 4 months. Occupational therapist or movement therapist are involved in the protocol to optimize activity levels taking into account individual capacity and needs. The inclusion phase ends June 2013. The outline and content of the treatment protocol will be presented. Next to that, preliminary results with regard to the trial will be discussed.

Correspondence address: M.Sc. Joyce Kootker, Radboud University Nijmegen Medical Centre, Department of Rehabilitation, Nijmegen Centre for Evidence Based Practice, Reinier Postlaan 2, NL-6525 Nijmegen, The Netherlands. E-mail: J.Kootker@reval.umcn.nl

### Lecture 5: Integrating neuropsychological and cognitive-behavioral treatment approaches for outpatients with cognitive and emotional-motivational difficulties after acquired brain injury

Cornelia Exner[1], Bettina Doering[2], Nico Conrad[2], Anna Künemund[2], Sarah Zwick[2] and Winfried Rief[2]
[1]*University of Leipzig, Institute of Psychology, Department of Clinical Psychology and Psychotherapy, Leipzig, Germany*
[2]*University of Marburg, Department of Clinical Psychology and Psychotherapy, Marburg, Germany*

After acquired brain injury (ABI) the risk for comorbid mental disorders is notably increased. Especially comorbid depression reduces psychosocial functioning and jeopardizes the progress of rehabilitation efforts. Treatment of these complex conditions requires the combination of neuropsychological and psychotherapy treatment approaches. However, evidence from controlled trials on the merits of standard psychotherapy protocols e.g. cognitive-behavioral therapy after ABI is still sparse. We report preliminary data from an ongoing randomized wait-list controlled trial of combined neuropsychological and cognitive-behavioral therapy for outpatients in the post-acute phase of recovery from ABI. 62 participants were included into the study and were randomly assigned to receive either the treatment at once or after a waiting period of five months. The treatment protocol included three neuropsychological modules aiming at compensation for deficits in attention, memory and executive functioning. Three additional cognitive-behavior psychotherapy modules targeted negative emotions such as anger and depression and supported adjustment of self-concepts and life goals after ABI. Based on a comprehensive diagnostic battery and establishment of shared treatment goals each subjects got assigned 3–4 modules depending on his individual rehabilitation needs. Participants received an average of 30 therapy sessions. Outcome variables included measures of neuropsychological functioning, mental disorders, daily activities, participation and quality of life and were administered to participants before and after treatment and after a 6-months follow-up. Preliminary pre-post analyses of 45 participants who have already completed treatment show medium treatment effects on symptoms of depression and general psychopathology, improvement of daily functioning and participation and increase in subjective quality of life.

Correspondence address: Prof. Dr. Cornelia Exner, University of Leipzig, Institute of Psychology, Department of Clinical Psychology and Psychotherapy, Seeburgstr. 14-20, D-04103 Leipzig, Germany. Tel.: +49 341 9735931; Fax: +49 341 9735939; E-mail: exnerc@uni-leipzig.de

### Symposium 15: Attention and executive functions in pediatric neurology

Karen Lidzba
*University Children's Hospital, Pediatric Neurology and Developmental Medicine, Tübingen, Germany*

Attention and executive functions are impaired in patients with a wide range of neurological disorders, and the dysexecutive syndrome is a well established concept for adult patients. In child neuropsychology, however, the pattern is not as clear: Due to the long developmental trajectory of executive and complex attention functions, it may be very difficult to identify deficits in these functions in young children. On the other hand, executive or attentional dysfunction may be an unspecific component of global intellectual impairment. Lastly, in clinical child psychology, problems in attention and executive functions are the hallmarks of attention deficit-/hyperactivity disorder (ADHD), which is a known comorbidity of a range of conditions in pediatric neurology.
In this symposium we will present data on attention and executive functions in a range of neurological conditions of children, illustrating commonalities, specificities and comorbidity. Two talks will present data on attention and executive functions in former preterm children, focussing on the developmental aspect by illustrating very early precursors of attention in toddlers (Reuner) and by examining age-effects on executive functions in school-children (Everts). The impact of attention deficit on the cognitive development of children with Neurofibromatosis type 1 will illustrate a "dual hit" situation, where a labile neural system is further hampered by attention problems (Lidzba). Similarly, in children with lesional brain damage, such as children with cerebral palsy (Di Lieto) or traumatic brain injury (Benz), executive functions seem to be a core deficit, which is, in the case of TBI, predictive of longterm cognitive outcome.

Correspondence address: Dr. Karen Lidzba, University Children's Hospital, Pediatric Neurology and Developmental Medicine, Hoppe-Seyler-Str. 1, D-72076 Tübingen, Germany. Tel.: +49 70712980881; Fax: +49 7071295236; E-mail: karen.lidzba@med.unituebingen.de

### Lecture 1: Focused attention in preterm and term born infants. Relationship with cognition and self-regulation in early and later infancy

Gitta Reuner[1], Andrea Weinschenk[1], Anna C. Fields[1], Sabina Pauen[2] and Joachim Pietz[1]
[1]*University Children's Hospital, Neuropediatrics, Heidelberg, Germany*
[2]*University of Heidelberg, Department of Psychology, Heidelberg, Germany*

AIM: Infants born preterm are at high risk for later deficit in attention but little is known about the early development of attention and the linkages between prematurity, specific attention functions, self- regulation and global cognitive performance. In our study we analysed the relationship between focused attention (FA), observed global cognitive performance and self-regulation derived from parental reports in preterm and term born infants.
METHOD: At seven months corrected age (ca) 93 infants born preterm and 40 term born infants were examined with an object examination task. FA was derived from the videotaped sequences as the duration of examination of the presented toy over three trials of 20 seconds each. Cognitive performance was assessed at 7 months and 24 months (ca) with the Bayley-II cognitive scale (MDI). Questionnaires on self-regulation were answered by the parents at both assessment times (Infant Behavior Questionnaire revised IBQ-R and Early Child Behavior Questionnaire ECBQ). Correlations between variables were analysed. A possible mediation effect of FA on the relationship between prematurity (gestational age) and cognitive performance was analysed.
RESULTS: Focused attention correlated significantly with both prematurity ($p < 0.05$) and MDI at 7 months of age ($p < 0.01$) but not with later MDI. With respect to parent rating of infant's self-regulation there were no significant interrelations in infancy, but those with higher FA were rated less impulsive at 24 months ca. Mediation analysis supports the hypothesized mediation effect of FA only in the preterm group ($p < 0.05$).
CONCLUSION: Findings suggest that individual differences in focused attention emerge very early and play an important role for later cognitive development and self-regulation in preterm born infants. Furthermore, results are discussed with respect to differences between behavioural and questionnaire data.

Correspondence address: Dr. Gitta Reuner, University Children's Hospital, Neuropediatrics, Im Neuenheimer Feld 430, D-69120 Heidelberg, Germany. Tel.: +49 62215632012; Fax: +49 6221565222; E-mail: gitta.reuner@med.uni-heidelberg.de

### Lecture 2: Executive functions in children born preterm – deficit or delay?

Regula Everts and Barbara Ritter
*Childrens University Hospital, Division of Neuropaediatrics, Development and Rehabilitation, Bern, Switzerland*

AIM: This study examined executive functions of 8 to12 year old children born very preterm or at very low birth weight in comparison to executive functions of same-aged term-born controls. In particular, we investigated whether preterm born children perform persistently poorer than controls in tasks of executive functions (deficit hypothesis) or whether preterm born children are able to catch up with increasing age (delay hypothesis).
METHODS: According to the executive function model of Miyake et al. (2000), three core executive functions were assessed in the preterm and term-born population in a cross-sectional study design: inhibition, working memory, and shifting. Seventy-two preterm born children with no or minimal neonatal brain lesions and no or minimal neurodevelopmental impairment and 50 healthy term-born controls were recruited. All children completed well-known and commonly used neuropsychological tasks of inhibition, working memory, and shifting.
RESULTS: Results revealed that preterm born children showed the same developmental pattern of executive functions as controls, with a large performance gain between the ages of 8 to 12 years in shifting, medium performance gain in working memory and only small performance gain in inhibition. Interestingly, the performance differences between preterm children and controls in inhibition, working memory and shifting declined with age.
CONCLUSION: These data point towards a catch-up in executive functions of preterm born children; between the ages of 8 to 12 years, preterm born children seem to steadily approach the performance level of controls. Consequently, we provide preliminary evidence that poor performance in inhibition, working memory and shifting of young preterm born children with no or minimal brain lesions and no or minimal neurodevelopmental impairment might reflect a delay of executive functions rather than a persistent executive deficit.

Correspondence address: PD Dr. Regula Everts, Childrens University Hospital, Division of Neuropaediatrics, Development and Rehabilitation, Inselspital, CH-3010 Bern, Switzerland. Tel.: +41 316329424; Fax: +41 316329500. E-mail: regula.everts@insel.ch

### Lecture 3: Neurofibromatosis Type 1: The complex interplay of cognition and attention deficit

Karen Lidzba[1], Sofia Granström[2], Jorge Lindenau[2] and Viktor-Felix Mautner[2]

[1]*University Children's Hospital, Pediatric Neurology and Developmental Medicine, Tübingen, Germany*
[2]*Department of Neurology, University Medical Centre Hamburg-Eppendorf, Hamburg, Germany*

AIM: A common comorbidity of Neurofibromatosis Type 1 (NF 1) is Attention Deficit with or without Hyperactivity (AD(H)D). While NF 1 is associated with problems in cognitive development, comorbid AD(H)D represents an additional risk factor for cognition, as shown in an IQ difference between children affected by NF 1 with or without AD(H)D. In this study, we tested the hypothesis that permanent medication with Methylphenidate can rescue cognitive problems in children with NF 1 and comorbid AD(H)D.
METHOD: We retrospectively analysed data of a clinical sample of children and adolescents with NF 1 with or without comorbid AD(H)D, who underwent standardized neuropsychological diagnostics (WISC, T.O.V.A.) twice (age range: T1 6 – 14 years; T2 7 – 16 years; mean time interval 49.09 months). 16 children without AD(H)D (7 males) were compared to 14 unmedicated children with AD(H)D (6 males) and to 13 medicated children with AD(H)D (11 males). A repeated measures analysis of covariance (rmANCOVA) was used to test for effects of medication and attention on cognitive outcome (full-scale IQ).
RESULTS: When controlling for gender effects, we found a significant interaction between time and group, i.e., medicated children with NF 1 improved significantly in full-scale IQ from T1 to T2 (IQT1 = 80.38, IQT2 = 98.38, CIdiff: −25.59 to −10.40, $p < 0.0001$), while this effect was not evident for the other groups (ADHD-/MPH-: IQT1 = 97.88, IQT2 = 96.63, 95% CIdiff: 6.13 to 8.63, $p = 0.723$; ADHD+/MPH-: IQT1 = 86.71, IQT2 = 90.64, CIdiff: −8.65 to 0.797, $p = 0.096$). The interaction remained significant when adding attention improvement (T.O.V.A.: change in reaction time variability from T1 to T2) as covariate.
CONCLUSION: Cognitive development of children and adolescents with NF 1 and comorbid AD(H)D may profit from MPH medication. As improvements in attention do not seem to be a very strong source of this effect, future prospective studies will have to evaluate the mechanisms behind it.

Correspondence address: Dr. Karen Lidzba, University Children's Hospital, Pediatric Neurology and Developmental Medicine, Hoppe-Seyler-Str. 1, D-72076 Tübingen, Germany. Tel.: +49 70712980881; Fax: +49 7071295236; E-mail: karen.lidzba@med.uni-tuebingen.de

### Lecture 4: Attention and executive functions in children with congenital spastic diplegia: Contribution of the NEPSY-II

Maria Chiara Di Lieto[1], Paola Brovedani[1], Chiara Pecini[1], Anna Maria Chilosi[1], Vittorio Belmonti[2], Franco Fabbro[3], Cosimo Urgesi[3] and Giovanni Cioni[1]
[1]*IRCCS Stella Maris, Department of Developmental Neuroscience, Calambrone, Pisa (Italy)*
[2]*University of Pisa, Department of Developmental Neuroscience, Pisa, Italy*
[3]*University of Udine, Department of Human Sciences, Udine, Italy*

AIM: The literature on executive functions and attention in children with congenital spastic diplegia is scant. Deficits in tasks measuring response inhibition, task switching, sustained attention and visual search have been reported and have not been systematically correlated with neuroimaging data and severity of motor impairment. The aim of this preliminary study is to describe the performance in the attentional and executive functions domain at the NEPSY-II (Developmental Neuropsychological Assessment) in relation to abilities in the other cognitive domains, in children with spastic diplegia and periventricular leukomalacia.
METHODS: NEPSY-II is a comprehensive battery comprising 33 tests measuring attention/executive functions, language, memory, sensory-motor skills, visuospatial processing and social perception. Twelve patients with spastic diplegia and periventricular leukomalacia, aged 7–13 years were selected from a larger sample of children with cerebral palsy referred to our tertiary care hospital. Inclusion criteria were: available brain MRI, absence of drug-resistant epilepsy, psychiatric disorders or severe sensory deficits, IQ $\geqslant$ 80 in either verbal or non-verbal domains.
RESULTS: The preliminary results on the one hand confirm the visual-spatial and visual-perceptual deficits consistently found in the literature in the presence of average performance in the language domain and in verbal memory subtests. More importantly, the NEPSY-II results reveal a specific deficit in performance in the attention/executive functions domain especially in terms of information processing speed.
CONCLUSION: Slow information processing could represent a core deficit in patients with spastic diplegia and contribute to the interpretation of the impaired performance in executive function and attention tasks, found by some authors. A larger sample size is needed to confirm this hypothesis and to correlate the results with neuroimaging data and degree of motor impairment.

Correspondence address: Maria Chiara Di Lieto, IRCCS Stella Maris, Department of Developmental Neuroscience, Via dei Giacinti 2, I-56018 Calambrone, Pisa, Italy. Tel.: +39 50886315; Fax: +39 5032214; E-mail: mcdilieto@inpe.unipi.it

### Lecture 5: "Growing into deficit" in young patients with Paediatric Traumatic Brain Injury (TBI) as a possible consequence of impaired executive functions?

Barbara Benz[1] and Annegret Ritz[2]
[1]*Goethe Universität Frankfurt am Main, Department of Developmental Psychology, Frankfurt am Main, Germany*
[2]*ZNS Hannelore Kohl Stiftung, Bonn, Germany*

AIM: In children, compromised cognitive development after early TBI has been suggested by recent empirical data. This presentation is focussing on the role of executive functions as part of a study of long-term outcome of paediatric TBI-patients.
METHODS: 141 subjects of an in-patient neurological rehabilitation unit for children and adolescents, 94% having suffered severe TBI according to international classification, were re-examined at the age of 16–23 years with a comprehensive neuropsychological test battery. Mean age at injury was 9.3 yrs (SD 2.3), assessment took place 9.95 yrs (SD 3.3) post trauma. Executive functions were assessed by the Controlled Word Association Test (COWA), Ruff Figural Fluency Test (RFFT), Wisconsin Card Sorting Test (WCST), and Trail Making Test (TMT-B/TMT-A ratio). Group means of subjects with intact vs. impaired executive functions were compared with respect to all other neuropsychological domains and effects of site of lesion, trauma severity and age at trauma were analysed.
RESULTS: Patients with scores less than 1 SD below the reference mean for executive skills were most severely impaired on all neuropsychological measures including IQ (German version of Wechsler Scales WAIS-R). When trauma severity was controlled, negative effects of early age at trauma persisted in the clinical sample in most neuropsychological domains, such as attention ($p < 0.042$), memory ($p < 0.005$), verbal competence ($p < 0.000$) and executive functions ($p < 0.012$). None of the other variables had a significant effect.

CONCLUSION: Type of persisting or emerging neuropsychological impairment has to be taken into account when calculating a patient's individual risk factor for long-term outcome. In this sample of paediatric TBI patients, the level of executive skills in late adolescence or early adulthood was found to be the most effective criterion for the separation of groups with good vs. poor cognitive outcome.

Correspondence address: Mrs Barbara Benz, Goethe Universität Frankfurt am Main, Department of Developmental Psychology, Grüneburgplatz 1, D-60323 Frankfurt am Main, Germany. Tel.: +49 6979835267; Fax: +49 6979876335267; E-mail: barbara.benz@unitybox.de

### Symposium 16: Implicit awareness in anosognosia: Clinical and experimental evidence

Implicit awareness in anosognosia: clinical and experimental evidence

Daniel Mograbi[1], Robin Morris[2], Paul Jenkinson[3] and Valentina Moro[4]
[1]*Institute of Psychiatry, King's College/PUC-Rio, Psychology, London, UK*
[2]*Institute of Psychiatry, King's College, Psychology, London, UK*
[3]*University of Hertfordshire, Psychology, London, UK*
[4]*Università di Verona, Phylosophy, Education and Psychology, Verona, Italy*

Unawareness of deficits caused by brain damage or neurodegeneration, termed anosognosia, has been demonstrated in a number of different neurological conditions. Clinical observation suggests that unawareness paradoxically can be accompanied by signs of understanding or representation of deficit, but not explicitly expressed. Such 'implicit awareness' is implied by or inferred from actions or statements of the person with neurological disorder. Although there is no consensus about the nature or cause of this type of awareness, there is convincing evidence to suggest its occurrence in a variety of neurological conditions. The current symposium will have 4 talks presenting experimental studies which explored 'implicit awareness' in either dementia or hemiplegia, followed by a discussion of the theoretical implications of the findings.

Correspondence address: Mr Dr Daniel Mograbi, Institute of Psychiatry, King's College/PUC-Rio, Psychology, De Crespigny Park, SE58AF London, UK. E-mail: daniel.mograbi@kcl.ac.uk

### Lecture 1: Implicit adaptation in Alzheimer's disease despite unawareness of performance

Daniel Mograbi[1], Richard Brown[2], Christian Salas[3] and Robin Morris[2]
[1]*Institute of Psychiatry, King's College/PUC-Rio, Psychology, London, UK*
[2]*Institute of Psychiatry, King's College, Psychology, London, UK*
[3]*Bangor University, Psychology, Bangor, UK*

Lack of awareness about performance in tasks is a common feature of Alzheimer's disease (AD). Nevertheless, clinical anecdotes have suggested that patients may show behavioural responses to the experience of failure despite expressing limited awareness. We explored this question experimentally using novel success/failure manipulation paradigms. Computerised tasks which expose participants to systematic success or failure were developed in which performance success was titrated for each participant and then difficulty was set either above or below this level to establish individually success and failure levels. Two experiments were carried out: the first, investigated immediate emotional responses to failure; and the second, long-term task avoidance in a follow-up session. Results of the first study indicated that, relative to controls, AD patients exhibited impaired awareness of performance, but comparable differential reactivity to failure relative to success tasks, both in terms of self-report and facial expressions. Long-term adaptation in the second study was correlated with awareness in the first session, even though AD patients had no memory of doing the tasks. These findings are discussed in relation to current theories of consciousness which emphasise access as its main feature.

Correspondence address: Dr Daniel Mograbi, Institute of Psychiatry, King's College/PUC-Rio, Psychology, De Crespigny Park, SE58AF London, UK. E-mail: daniel.mograbi@kcl.ac.uk

### Lecture 2: Dissociation between self-reported awareness and objective performance during reaching movements in anosognosia for hemiplegia

Paul Jenkinson[1], Catherine Preston[2] and Roger Newport[3]

[1]*University of Hertfordshire, Psychology, Hertfordshire, UK*
[2]*Karolinska Institutet, Department of Neuroscience, Solna, Sweden*
[3]*University of Nottingham, School of Psychology, Nottingham, UK*

The 'comparator' model of motor control and awareness proposes that an ongoing comparison of predicted and actual sensory feedback forms the basis of motor awareness, via the detection of sensorimotor discrepancies. This model has also been used to explain anosognosia for hemiplegia (AHP; i.e. unawareness of paralysis following stroke), in terms of a failure to detect such discrepancies combined with a pathologically exaggerated reliance on motor intentions to inform awareness. In this talk I will discuss recent work by Preston, Jenkinson and Newport (2010), which assessed the proposed failure to detect sensorimotor discrepancies in a patient with AHP (GG), using the non-paralysed arm. In so doing, we aimed to provide direct empirical evidence regarding the operation of the motor comparator(s). Goal-directed reaching movements made by GG using the non-paralysed (right) arm were either modified by a computer or left unperturbed. Comparison with hemiplegic and young, healthy controls revealed that GG was unable to detect computer-generated visual perturbations as large as 20°. Remarkably, GG claiming on all occasions that his movements were unperturbed, uncorrected or accurate, and he also failed to notice large corrective movements that he made when attempting to compensate for the visual perturbations. These results suggest that the comparators implicated in AHP are functioning at a sub-optimal level, and that monitoring of both the right and left arms might involve a common right-hemisphere network. Furthermore, the presence of large corrective movements, despite verbal reports of perfect performance, reveals a dissociation between subjective self-reports of motor awareness and objective performance in this case of AHP.

Correspondence address: Dr. Paul Jenkinson, University of Hertfordshire, Psychology, College Lane, Hatfield, AL10 9AB Hertfordshire, UK. E-mail: p.jenkinson@herts.ac.uk

### Lecture 3: Anosognosia and reactivity to dementia-related material in Alzheimer's disease

Robin Morris[1], Richard Brown[2] and Daniel Mograbi[3]

[1]*Institute of Psychiatry, King, Psychology, London, UK*
[2]*Institute of Psychiatry, King's College, Psychology, London, UK*
[3]*Institute of Psychiatry, King's College/PUC-Rio, Psychology, London, UK*

OBJECTIVES: Lack of awareness of illness or neuropsychological deficit in people with Alzheimer's disease (AD) has been shown to have some dissociation with awareness of symptoms when viewing the symptoms of other people. The presented study investigated emotional reactivity when viewing film material depicting a person with AD, comparing this to viewing other film material. The correlates with anosognosia were explored.
PARTICIPANTS/METHODS: A film depicting a person with Alzheimer's disease was shown. Emotional reactivity was compared to that following two other films, one including material from a UK light comedy television program that was predicted to produce positive mood and another film about a person with cancer, predicted to induce negative mood. Emotional reactivity in each case was measured using before and after measurement of mood state and also by filming participant facial expressions with Facial Affect Coding (FAC) ratings.
RESULTS: The level of emotional reactivity was slightly less in people with Alzheimer's disease than in normal control participants, including the negative mood state associated with viewing the film about the person with Alzheimer's disease. Facial reaction was negatively correlated with the degree of awareness the people with Alzheimer's disease had in relation to their own illness. Emotional reaction to other film material was not associated with this awareness.
CONCLUSIONS: In the early stage of AD, there was emotional reactivity to the film material inducing positive and negative mood, including the Alzheimer film. The negative correlation with awareness of illness is interpreted as suggesting that heightened emotional reaction on confronting Alzheimer's disease symptoms might be predictive of less awareness due to emotionally induced inhibition. Alternative explanations for this finding are also considered.

Correspondence address: Mr Prof Robin Morris, Institute of Psychiatry, King, Psychology, De Crespigny Park, SE58AF London, UK. E-mail: robin.morris@kcl.ac.uk

### Lecture 4: How many forms of anosognosia? Residual forms of awareness in anosognosia for hemiplegia

Valentina Moro
*Università di Verona, Phylosophy, Education and Psychology, Verona, Italy*

Anosognosia for hemiplegia (AH) is characterized by a lack of awareness of motor disorders and appears associated with fronto-temporal-parietal damage. It is a specific syndrome which cannot be explained by concomitant neurological deficits such as sensory deafferentation, presence of contralesional spatial neglect, mental confusion or deficits in frontal lobe functions. In addition, it appears as an "inconsistent" syndrome, which may concern different aspects of deficits and be fluctuating in time.
In this complex frame, we will show the existence of "dissociations" in anosognosia and the existence of possible "residual" forms of awareness in AHP. The specific neuroanatomical correlates will be discussed as well.
Finally, the impact of these results in neuropsychological assessment and rehabilitative training will be demonstrated by means of the results of specific experimental trainings.

Correspondence address: Prof Valentina Moro, Università di Verona, Phylosophy, Education and Psychology, Lungadige Porta Vittoria 17, 37129 Verona, Italy. E-mail: valentina.moro@univr.it

### Symposium 17: New advances in semantic memory: Neuropsychological and neuroimaging research

New advances in Semantic Memory: Neuropsychological and neuroimaging research

J. Frederico Marques[1] and Stefano Cappa[2]
[1]*University of Lisbon, Faculty of Psychology, LISBOA, Portugal*
[2]*Vita Salute San Raffaele University and San Raffaele Scientific Institute, Milan, Italy*

**SUMMARY:** This symposium presents new advances in semantic memory research. The main goal is to present new neuropsychological and neuroimaging data on different topics that have been signaled as most relevant for further directions on research about semantic memory (e.g. Kiefer and Pulvermüller, 2012). The presentations will cover the neural pathways of semantic processes, the contribution of the anterior temporal lobes to semantic memory and the behavioral and neural correlates of the representation of concrete and abstract concepts. Implications for cognitive models of semantic memory and for understanding different semantic memory disorders will be particularly discussed.

Correspondence address: Mr J. Frederico Marques, University of Lisbon, Faculty of Psychology, Alameda da Universidade, 1649-013 LISBOA, Portugal. E-mail: jfmarques@fp.ul.pt

### Lecture 1: Neural correlates of phonological versus semantic dichotomy explored with intra-cerebral electrodes and cortical stimulation

Jean François Démonet
*University of Lausanne, Lausanne, Switzerland*

GOAL: A striking feature of the functional organization of brain structures supporting language functions consists in their dual-route organization so that sublexical processing is supported by a parietal-frontal network while the lexical semantic one depends on a ventral temporal pathway. Direct evidence of this global functional pattern was brought up directly by invasive explorations in patients while performing language tasks.
METHODS: ERP and high-frequency oscillations recording were obtained from patients who suffered from intractable epilepsy and benefited from SEEG exploration at the pre-surgical stage. Direct cortical stimulation was used to map language functions in other patients before removal of low grade gliomas.
RESULTS: A consistent pattern of results observed at the single-subject level, showed that specific recording sites respond to either sublexical or semantic tasks. In the latter, cortical localizations were observed in the left middle temporal and fusiform gyri, close to the basal temporal language area.
CONCLUSION: The significance of these findings will be discussed in the framework of the recent conceptualizations regarding distributed versus centric conceptualizations of the neural correlates of semantic processes.

Correspondence address: Mr J. Frederico Marques, University of Lisbon, Faculty of Psychology, Alameda da Universidade, 1649-013 LISBOA, Portugal. E-mail: jfmarques@fp.ul.pt

**Lecture 2: Convergent evidence for the basal temporal semantic area**

Matthew Lambon-Ralph
*University of Manchester, MANCHESTER, United Kingdom*

GOAL: A considerable body of neuropsychological and neuroimaging research from semantic dementia has implicated the anterior temporal lobes in the representation of coherent, transmodal concepts. Given that the temporopolar region implicated in semantic dementia is relatively large and contains multiple cytoarchitectural and connectivity differences, it becomes important to explore the roles that each subregion has in semantic representation.
METHODS: In neurologically-intact participants, in vivo MR tractography was used to explore the intra and inter-temporal lobe connectivity to the rostral temporal region; distortion-corrected fMRI was used to investigate the semantic-task responsivity of various subregions and how these vary across modalities of input; in patients with temporal lobe epilepsy, implanted grid electrodes were used to explore task-related local field potentials and to assess the effect of direct cortical stimulation on semantic performance.
RESULTS: The convergent picture that emerges from these studies is that anterior superior temporal regions are implicated in semantic processing that is greater for auditory and verbal materials than picture-based tasks. In comparison the ventral anterior temporal area (anterior ITG and fusiform) is implicated in transmodal semantic processing for receptive and expressive tasks. These results fit closely with the differential pattern of white-matter connectivity to these regions and the distribution of ATL atrophy observed in semantic dementia (which disproportionately affects the ventral and polar regions).
CONCLUSION: The ventral temporal region – the “basal temporal semantic area” – is a centrepoint for the representation of coherent concepts.

Correspondence address: Mr J. Frederico Marques, University of Lisbon, Faculty of Psychology, Alameda da Universidade, 1649-013 LISBOA, Portugal. E-mail: jfmarques@fp.ul.pt

**Lecture 3: What about abstract concepts? Imaging and neuropsychological evidence**

Stefano F. Cappa[1] and Gabriella Vigliocco[2]
[1]*Vita Salute San Raffaele University and San Raffaele Scientific Institute, MILAN, Italy*
[2]*University College, London, United Kingdom*

GOAL: Abstract concepts are generally held to be linguistically coded, in line with imaging evidence of greater engagement of the left perisylvian language network for abstract than concrete words. According to a recent hypothesis, based on behavioural studies, abstract concepts, in addition, entail affective processing to a greater extent than concrete concepts.
METHODS AND RESULTS: In a fMRI experiment we found that abstract processing, compared to concrete processing shows greater engagement of rostral anterior cingulate cortex, an area associated with emotion.
CONCLUSION: Within the framework of embodiment views of semantic representation, the idea that affective experience is crucial in the grounding of abstract concepts is also starting to be tested in neuropsychological studies involving acquired and developmental brain disorders.

Correspondence address: Mr J. Frederico Marques, University of Lisbon, Faculty of Psychology, Alameda da Universidade, 1649-013 LISBOA, Portugal. E-mail: jfmarques@fp.ul.pt

**Lecture 4: The representation of abstract and concrete concepts: Insights from semantic dementia and a new ‘old’ task**

J. Frederico Marques[1] and Annik Charnallet[2]
[1]*University of Lisbon, Faculty of Psychology, LISBOA, Portugal*
[2]*University of Grenoble, France*

GOAL: The present study addresses the ongoing controversy of the contribution of the anterior temporal lobes to the representation of concrete and abstract concepts. In particular, it investigates how the nature of the associations to these concepts observed in healthy individuals is affected in patients with Semantic Dementia (SD).
METHODS: For this purpose we studied a group of SD patients and a group of matched healthy individuals using a standard free association task where we orthogonally manipulated concept imageability (more vs. less imageable) and concept frequency (low frequency vs. high frequency), while controlling for a number of other important variables.
RESULTS: The classification of the associations showed that while in healthy individuals more language associations were observed for abstract concepts and more sensorimotor associations were observed for concrete concepts, in SD patients language associations were strongly affected independently of concept type. In addition, SD patients made more non-related answers and omissions for low frequency concepts.
CONCLUSION: These results favor an interpretation of the role of the anterior temporal lobes in semantic memory that is independent of concept type and that sensorimotor associations are more robust than language associations in the face of general semantic degradation.

Correspondence address: Mr J. Frederico Marques, University of Lisbon, Faculty of Psychology, Alameda da Universidade, 1649-013 LISBOA, Portugal. E-mail: jfmarques@fp.ul.pt

### Symposium 18: Determinants of neuropsychological impairments in mental disorders

Thomas Beblo[1] and Nils Inge Landro[2]
[1]*Ev. Hospital Bielefeld, Clinik for Psychiatry und Psychotherapy Bethel, Bielefeld, Germany*
[2]*University of Oslo, Department of Psychology, Oslo, Norway*

Neuropsychological impairments in mental disorders have been increasingly investigated. Recent studies have demonstrated that these deficits were influenced by several factors. In our symposium, Determinants of neuropsychological impairments in mental disorders, we focus on the associations between neuropsychological functioning on the one hand and brain functioning, serotonin transporter polymorphisms and everyday cognitive functioning on the other. In the study of Fast et al., "Neurobiological underpinnings of cognition-emotion interaction in schizophrenia", the authors investigated brain activation during affective attachment, affective perspective taking and affective regulation and found dysfunctions of a fronto-temporal-occipital network. These findings may explain the patients' difficulties in self-other distinction and the psychopathological self-relevance of social and affective stimuli. The study of Toepper et al. focused on "fMRI correlates of spatial working memory in healthy subjects and MCI patients". Patients with mild cognitive impairment (MCI) showed decreased working memory performance and reduced brain activation in the left premotor cortex. In their study "The effects of 5-HTTLPR polymorphisms on cognitive control in healthy subjects at genetic risk for depression" Landrø et al. investigated healthy individuals with or without genetic risk for depression. Individuals at risk showed an impaired inhibitory control. The authors concluded that ineffective inhibitory control might be a promising intermediate phenotype for the genetic risk for depression. Beblo investigated "Subjective memory complaints and 'objective' memory performance in patients with borderline personality disorder (BPD)". While patients reported severe memory deficits in everyday life, they did not show memory tests impairments. The results may indicate that memory deficits in BPD are related to factors that are present in everyday life but not during neuropsychological testing.

Correspondence address: PD Dr. Thomas Beblo, Ev. Hospital Bielefeld, Clinik for Psychiatry und Psychotherapy Bethel, Remterweg 69-71, D-33615 Bielefeld, Germany. Tel.: +49 521 77278512; E-mail: thomas.beblo@evkb.de

### Lecture 1: Neurobiological underpinnings of cognition-emotion interaction in schizophrenia

Kristina Hennig-Fast[1], Dominik Meißner[2], Anna Buchheim[3], Sandra Dehning[2], Janosch Blautzik[4], Norbert Mueller[2], Peter Zill[2], Maximilian Reiser[4] and Hans-Juergen Moeller[2]
[1]*LMU Munich, Department of Psychiatry and Psychotherapy, München, Germany*
[2]*LMU Munich, Department of Psychiatry and Psychotherapy, Munich, Germany*
[3]*Department of Psychology, University of Innsbruck, Innsbruck, Austria*
[4]*LMU Munich, Department of Clinical Radiology, Munich, Germany*

Emotion theorists have long time posited the critical role for affect in the modulation of behavior and cognition. When considering the adaptive function of emotion and cognition and their interaction both can be posited to function as control systems to regulate behavior. Patients with schizophrenia often demonstrate profound functional dysregulation compared to healthy subjects especially in fronto-temporal-limbic brain areas. These functional anomalies are related to the clinical symptomatology of the patients. In the present fMRI study 3 experiments on affective attachment, affective perspective taking and affective regulation were conducted in patients with schizophrenia. In accordance to our hypotheses ocytocin level and behavioral socio-emotional functions in schizophrenia were reduced. When analysing conjunctive brain activation of all 3 experimental paradigms a fronto-temporal-occipital network was found to be dysfunctional in patients including brain areas that are critical for episodic autobiographical memory, mirroring other's emotions, self-reflection, affective risky decision-making and painful information processing. The findings are presented in relation to oxytocin levels in patients with schizophrenia (ICD-10: F20; PANSS $< 78$) and healthy subjects (each group: males, $n = 20$, IQ $< 85$). Our findings can explain the difficulties in self-other distinction and the psychpathological self-relevance of social and affective stimuli in schizophrenia.

Correspondence address: PD Dr. Kristina Hennig-Fast, LMU Munich, Department of Psychiatry and Psychotherapy, Nußbaumstraße 7, D-80336 München, Germany. E-mail: Kristina.Fast@med.uni-muenchen.de

### Lecture 2: Neural correlates of impaired inhibitory processes in mild cognitive impairment

Max Toepper[1], Hans J. Markowitsch[2], Leona Kater[2], Helge Gebhardt[3], Thomas Beblo[4], Eva Bauer[3], Stefan Kreisel[4], Friedrich G. Woermann[5], Christine Thomas[4], Martin Driessen[4] and Gebhard Sammer[3]
[1]*Ev. Hospital Bielefeld, Clinic for Psychiatry und Psychotherapy Bethel, Bielefeld, Germany*
[2]*University of Bielefeld, Department of Psychology, Bielefeld, Germany*
[3]*Justus-Liebig-University of Giessen, Cognitive Neuroscience at Center for Psychiatry, Giessen, Germany*
[4]*Ev. Hospital Bielefeld, Clinic for Psychiatry and Psychotherapy Bethel, Bielefeld, Germany*
[5]*Mara Hospital, Bethel Epilepsy Center, Bielefeld, Germany*

Alzheimer's disease (AD) is characterized by different structural and functional cerebral changes which lead to increasing cognitive impairment and severe deficits in daily routine. The prodromal phase of AD is most commonly defined as amnestic mild cognitive impairment (aMCI). Previous research points towards a specific inhibitory dysfunction in AD that already exists in aMCI. Due to the heterogeneity of the concept MCI itself and different methodological approaches, our knowledge about the functional cerebral mechanisms underlying this inhibitory dysfunction is far from complete. Functional magnetic resonance imaging (fMRI) was used to examine brain activation during the Corsi-Block-Tapping-Test (CBT; Corsi, 1972) and a modified version of the block suppression test (BST; Beblo et al., 2004). The CBT requires the encoding, maintenance and retrieval of serially presented target locations. The BST additionally requires the inhibition of spatial distractors during CBT encoding. Our study included 7 aMCI patients and 18 healthy volunteers matched with regard to age, gender, and education. All patients met the criteria of aMCI (Petersen et al., 2004). Activation differences were analyzed for BST retrieval (BST minus CBT activation) including all, and only correct trials. Compared to healthy controls, aMCI patients showed decreased BST performance accuracy and increased response durations. Moreover, aMCI patients showed reduced activation intensity during BST retrieval, particularly within left premotor cortex. Interestingly, group differences disappeared when only correct trials were included into brain data analysis. Our results reveal aMCI-associated changes within the cerebral network that is known to be recruited during BST performance. MCI patients particularly showed reduced left premotor cortex activation that seems to be at least partly associated with impaired inhibitory functioning on the behavioural level.

Correspondence address: Dr. Max Toepper, Ev. Hospital Bielefeld, Clinic for Psychiatry und Psychotherapy Bethel, Remterweg 69-71, D-33615 Bielefeld, Germany. E-mail: max.toepper@evkb.de

### Lecture 3: The effects of 5-HTTLPR polymorphisms on cognitive control in healthy subjects at genetic risk for depression

Nils Inge Landro
*University of Oslo, Department of Psychology, Oslo, Norway*

A number of studies have documented impaired cognitive control in patients with current major depressive disorder (MDD). It has also been shown that cognitive control impairments persist during remission from depression, suggesting that impaired cognitive control could be a trait marker for depression. However, such findings cannot be interpreted as evidence of a vulnerability factor because they might be the result of "scarring" or residual effects after the depressive episode. A logical approach to this important question is therefore compare healthy individuals with or without genetic risk. The gene that codes for the serotonin transporter is particularly relevant. A relatively common polymorphism (5-HTTLPR) in the promoter region of the human 5-HTT gene (SLCA4) results in 2 common alleles or variants. The short allele is associated with a nearly 50% reduction in 5-HTT availability, as compared to the long variant, and there are key reports of associations between the short allele and risk for depression. In this study 157 healthy subjects, carefully screened for psychopathology, were genotyped. They were tested on three tasks tapping distinct dimensions of cognitive control from CANTAB; Inhibitory control (Stop Signal Reaction Time; SSRT), Mental flexibility (Intra/Extra dimensional set shifting) and Working memory (Spatial Working Memory). The results showed that carriers with the short variant of the gene have significantly less ability to inhibit a pre potent response on the SSRT task, as compared to long allele carriers, whereas there were no significant gene effects on set shifting and working memory. This pattern of findings indicate that less effective inhibitory control might be a promising intermediate phenotype for the genetic risk for depression.

Correspondence address: Prof. Dr. Nils Inge Landro, University of Oslo, Department of Psychology, Forskningsveien 3A, Nor-0373 Oslo, Norway. E-mail: n.i.landro@psykologi.uio.no

### Lecture 4: Subjective memory complaints and "objective" memory performance in patients with Borderline Personality Disorder

Thomas Beblo
*Ev. Hospital Bielefeld, Clinic of Psychiatry and Psychotherapy Bethel, Bielefeld, Germany*

It is still a matter of debate whether patients with Borderline Personality Disorder (BPD) suffer from memory deficits. Existing studies indicate - if ever – small impairments of memory test performance. However, it was shown in patients with related disorders, such as depression, that self-reported impairment overtop test malfunction. The present study aimed at investigating memory performance of BPD patients as assessed by memory tests and a questionnaire for memory functioning in everyday life. Thirty-two patients with BPD and 62 healthy control subjects were included into the study. Both groups of subjects were similar with respect to age, education and gender. Subjects completed verbal and nonverbal memory tests as well as the everyday memory questionnaire (EMQ). BPD patients reported severe memory deficits in everyday life but did not show memory tests impairments. The results remained stable even when all BPD patients with acute or lifetime depression comorbidity were excluded from analyses. These data indicate memory impairment of BPD patients for everyday life. However, it can not to be ruled out that increased memory complaints result from patients' negative self perception. Future research needs to clarify reasons for memory complaints of BPD patients.

Correspondence address: PD Dr. Thomas Beblo, Ev. Hospital Bielefeld, Clinic of Psychiatry and Psychotherapy Bethel, Remterweg 69-71, D-33615 Bielefeld, Germany. Tel.: +49 521 77278512; E-mail: thomas.beblo@evkb.de

### Symposium 19: Neurofeedback training for children with ADHD: Recent advances and new applications

Renate Drechsler
*University Clinic of Child and Adolescent Psychiatry, University of Zurich, Zürich, Switzerland*

Neurofeedback training (NF) has found increasing acceptance as a non-pharmacological treatment for children with Attention Deficit Hyperactivity Disorder (ADHD). NF aims at the regulation of electrical brain activity patterns. In the first talk, a summary of the most influential multicentre training study of the recent years will be given, including long-term outcome data and results from new follow-up studies with the application of NF to Tic Disorder (H. Heinrich). "Classic" protocols of NF frequency training, which are aimed at the improvement of attentional control, feed back activity of theta or beta bands. The feedback of gamma-band activity is a new approach (T. Bogen). Though technically challenging, gamma-band NF promises to be an efficacious treatment for emotion regulation deficits, a frequent comorbidity of children with ADHD.
The third presentation introduces tomographic neurofeedback training for children with ADHD (D. Brandeis). In tomographic NF, the activation of a determined brain region is calculated and fed back to the individual, in contrast to conventional NF, which uses electrical activity at the scalp surface.
Finally, the importance of choosing an appropriate control condition for the analysis of NF treatment specificity will be discussed in the last talk (R. Drechsler). A differential EMG (electromyogram) biofeedback training is proposed as control condition that seems preferable from an ethical point of view to sham NF control.

Correspondence address: Dr. Renate Drechsler, University Clinic of Child and Adolescent Psychiatry, University of Zurich, Neumuensterallee 9, CH-8032 Zürich, Switzerland. Tel.: +41 43 499 27 66; E-mail: renate.drechsler@kjpd.uzh.ch

### Lecture 1: Standard neurofeedback protocols in children with ADHD: Methodical introduction and results from clinical studies

Hartmut Heinrich
*Heckscher-Klinikum, München, Dept. of Child and Adolescent Mental Health, University Hospital of Erlangen, Germany*

GOALS: Neurofeedback (NF) may be interpreted as a neuro-behavioural treatment for attention-deficit/hyperactivity disorder (ADHD) where children learn to modulate specific brain electrical activity patterns and how to apply their neuroregulation strategies in daily life. Training of slow cortical potentials (SCPs) and theta (4–7 Hz)/beta (13–20 Hz) training are considered as standard protocols.
METHODS: At the conference, the methodical basis of these protocols integrated into a hypothetical model of 'active ingredients' will be presented. NF studies of our group will be reflected with a focus on the up-to-now largest randomised controlled trial (about 100 children included) comparing NF (combination of SCP and theta/beta training) with a computerised attention training.
Results: In this trial, NF was superior to the control training in reducing severity of ADHD symptoms at post-training as well as at 6-month follow-up (medium effect size). Parents of both groups did not differ on evaluation scales which were applied to control for attributional effects. At the neurophysiological level, differential patterns for SCP and theta/beta training were obtained. In a second study on SCP training, a significant relationship between regulation skill and clinical outcome was obtained. In a third study, SCP training was superior to a theta/low-beta (12–15 Hz) training in reducing ADHD behaviour in children with tic disorders. Mainly, hyperactive/impulsive symptoms were observed to be reduced probably due to the fact that transfer into daily life focused on inhibition of behaviour (i.e. tic suppression).
CONCLUSIONS: There is clear evidence for specific effects of NF (SCP and theta/beta training) in ADHD so that NF based on these standard protocols may be supported as a treatment for ADHD. However, medium effect sizes indicate that NF should not be seen as a stand-alone intervention but rather as a module within a multimodal treatment tailored to the needs of a child.

Correspondence address: PD Dr. Hartmut Heinrich, Heckscher-Klinikum, München; Dept. of Child and Adolescent Mental Health, University Hospital of Erlangen, Schwabachanlage 6+10, D-91054 Erlangen, Germany. Tel.: +49 09131/8539122; E-mail: hartmut.heinrich@uk-erlangen.de

### Lecture 2: Gamma-band neurofeedback: Challenges in developing a new approach for the treatment of emotion regulation deficits

Thorsten Bogen, Tanja Legenbauer and Martin Holtmann
*LWL-University Hospital for Child and Adolescent Psychiatry, Ruhr-University Bochum, Hamm, Germany*

GOALS/BACKGROUND: Neurofeedback (NF) of slow cortical potentials (SCP) has been demonstrated to be an efficient treatment for symptoms of inattention and hyperactivity in attention deficit/hyperactivity disorder (ADHD). Despite the fact that ADHD during adolescence is often accompanied by deficits of emotion regulation, an efficient treatment for this comorbidity does not exist. In this study we are investigating whether NF can also be used for the treatment of emotion regulation deficits. The rationale for this approach was influenced by evidence from meditation research since high-amplitude gamma-band activity and long-distance phase synchrony were associated with high levels of emotional stability. In a pilot study, we use NF instead of meditation to induce high-levels of gamma-band activity. It is assumed that this allows accessing cognitive resources that are otherwise inaccessible for emotion regulation processes.
METHODS: The feasibility of gamma-band NF in a clinical population of 40 children and adolescents with ADHD shall be investigated. In particular its further use for the additional treatment of emotion regulation deficits in a multimodal inpatient treatment setting shall be evaluated. Before and after the NF intervention patients complete a comprehensive test-battery including measures of emotion regulation strategies, neuropsychological tests and behavioural parameters.
RESULTS: Difficulties exist particularly with regards to generating a robust feedback signal. The filtering of artefacts appears complex as muscle activity and saccadic eye-movements mask the gamma-band signal. First experiences with gamma-band NF will be reported on the basis of single-cases.
DISCUSSION: The implementation of gamma-band NF appears challenging. The talk shall present possible solutions for the problems encountered with this new protocol. Ideas for further application of gamma-band NF will be discussed.

Correspondence address: Mr M.Sc. Thorsten Bogen, LWL-University Hospital for Child and Adolescent Psychiatry, Ruhr-University Bochum, Heithofer Allee 64, D-59071 Hamm, Germany. E-mail: thorsten.bogen 1975@gmail.com

### Lecture 3: Tomographic neurofeedback in ADHD

Daniel Brandeis[1,2] and Renate Drechsler[3]
[1]*Department of Child and Adolescent Psychiatry and Psychotherapy, Cenntral Institute of Health, University of Mannheim, Germany*
[2]*University Clinic of Child and Adolescent Psychiatry, University of Zürich, Switzerland*
[3]*University Clinic of Child and Adolescent Psychiatry, University of Zurich, Zürich, Switzerland*

GOALS: Controlled EEG-neurofeedback studies reporting sizeable clinical effects on ADHD (attention-deficit/hyperactivity disorder) often fail to distinguish unspecific contributions from specific ones due to learned regulation of neurophysiological measures. Topographic EEG and source localization allows real time estimation and feedback of activity from specific dysfunctional regions. We targeted regulation in the anterior cingulate (ACC), a deep brain region affected in ADHD, as a new approach to increase specificity.
METHODS: In a tomographic neurofeedback (tNF) study, 13 ADHD children trained bidirectional regulation of their ACC brain activity during 36 lessons for theta and beta frequency bands and for slow cortical potentials (SCP). The ACC activity and its change over the course of the training and rest phases were computed using sLORETA (low-resolution electromagnetic tomography) of the 30-channel EEG.
RESULTS: Despite reducing ADHD symptoms and EEG-artefacts control over ACC activity was learned only in one SCP condition providing simple feedback. Instead, the ACC frequency distribution at rest normalized over the course of the training.
CONCLUSIONS: Clinical improvement and artefact/ movement reduction followed ACC-tNF without substantial learning of control, but with stabilisation and normalisation of oscillatory ACC activity at rest. Further studies need to test whether tNF can induce learning in brain regions implicated in ADHD. The assumption that targeting impaired brain regions in clinical groups for self regulation improves learning and outcomes also motivates NIRS- or fMRI-neurofeedback, and still requires critical evaluation.

References

[1] Liechti, M., Maurizio, S., Heinrich, H., Jäncke, L., Meier, L., Steinhausen, H. C., Walitza, S., Drechsler, R., & Brandeis, D. (2012). First Clinical Trial of Tomographic Neurofeedback in Attention-Deficit/Hyperac- tivity Disorder: Evaluation of Voluntary Cortical Control. Clin. Neurophysiol., 123, 1989–2005.

Correspondence address: Prof. Dr. Daniel Brandeis, Dep.of Child and Adolescent Psychiatry and Psychotherapy, Cenntral Institute of Health, University of Mannheim, Germany, University Clinic of Child and Adolescent Psychiatry, University of Zürich, Neumuensterallee 9, CH-8032 Zürich, Switzerland. E-mail: daniel.brandeis@zi-mannheim.de

**Lecture 4: Efficacy of neurofeedback training in ADHD: the importance of appropriate control conditions**

Renate Drechsler[1] and Daniel Brandeis[2,3]
[1]*University Clinic of Child and Adolescent Psychiatry, University of Zurich, Zürich, Switzerland*
[2]*Dep. of Child and Adolescent Psychiatry and Psychotherapie, Central Institue of Health, University of Mannheim, Germany*
[3]*University Clinic of Child and Adolescent Psychiatry, University of Zürich, Switzerland*

GOALS: Although the beneficial effects of neurofeedback (NF) training on ADHD symptoms are beyond doubt, the mechanisms leading to these effects remain unclear. Control conditions are critical to determine specific effects of NF in randomized controlled trials. The majority of controlled studies have included semi-active or active control conditions where NF is compared to treatments such as medication, computer-based cognitive programs or behavioural therapy. Only a minority of studies have used "sham" NF as control condition. The methodological and ethical problems of these studies, which failed to demonstrate the superiority of "genuine" compared to "sham" NF, will be discussed. Other biofeedback methods, such as electromyogram biofeedback (EMG-BF) are presented as alternatives.
METHODS: A differential EMG-BF protocol which feeds back activity from arm muscles involved in fine motor skills such as writing and grip was developed as a control condition for a complex tomographic neurofeedback protocol. Two groups of children with ADHD were trained with EMG-BF or NF over the same period of time, using the same training software and matching protocols.
RESULTS: A clinically relevant reduction of ADHD symptoms was observed in both groups of children. Specific effects were found for the EMG-BF group in neuropsychological tests related to fine motor skills and bimanual coordination. Only trends were found for specific electrophysiological treatments effects in the NF group.
DISCUSSION: EMG-BF represents a valuable control condition with a meaningful motor coordination and skill training for children with ADHD. Ethical as well as methodological aspects have to be considered when conducting clinical trials conform to standards of evidence-based medicine.

Correspondence address: Dr. Renate Drechsler, University Clinic of Child and Adolescent Psychiatry, University of Zurich, Neumuensterallee 9, CH-8032 Zürich, Switzerland. Tel.: +41 43 499 27 66; E-mail: renate.drechsler@kjpd.uzh.ch

**Symposium 20: Compensatory mechanisms in visual neglect**

Mario Bonato[1] and Paolo Bartolomeo[2]
[1]*University of Ghent, Department of Experimental Psychology, Ghent, Belgium*
[2]*INSERM, UMRS 975, Brain and Spine Institute, Department of Psychology, Catholic University, Paris and Milan*

Recent evidence suggests that neglect syndrome is intimately linked to compensatory mechanisms.
The mere fact that neglect can spontaneously improve with time from stroke onset raises the question of whether this improvement is substantial or to what extent it might be due to a more efficient implementation of compensatory strategies. A better understanding of the interplay of these compensatory mechanisms with neglect phenomenology would be conducive to a better understanding of neglect and of normal attentional processes, with the consequent deep theoretical and clinical consequences.
The present symposium encompasses three talks addressing these issues.
Paolo Bartolomeo (Inserm, Paris and Catholic University, Milan) will provide an introductory overview to the symposium briefly reviewing the past studies highlighting the role of compensatory strategies in neglect.

- Mario Bonato (Ghent University, Belgium) will focus on recent evidence that, in the post acute and chronic phases after a stroke contralesional spatial awareness can be severely impaired by the request to perform a concurrent, resource-demanding task, regardless of its nature (e.g. visual or auditory).
- Charlotte Russell (Brunel University, West London, UK) will present evidence for enduring deficits after the resolution of visuo-spatial neglect.
- Marine Lunven (INSERM, Brain and Spine Institute, France) will talk about anatomical predictors of neglect compensation.

The general discussion will address the clinical and theoretical relevance of these empirical findings.

Correspondence address: Dr. Mario Bonato, University of Ghent, Department of Experimental Psychology, Henry Duntalaan 2, B-9000 Ghent, Belgium. Tel.: +39 3284341010; E-mail: mariobonato@hotmail.com

### Lecture 1: Neglect: Diagnostic value of tasks where compensation is prevented

Mario Bonato
*University of Ghent, Department of Experimental Psychology, Ghent, Belgium*

Across a series of studies we have increased the attentional demands required by a spatial task. We asked right-hemisphere damaged patients either only to monitor visual space for the appearance of lateralized targets (single-task) or to perform also a concurrent task (dual-task conditions). The nature of the dual-task manipulation was either visual or auditory, and both led to similar, severe, impairments for contralesional hemispace with respect to the single-task condition, even in patients performing errorless in paper and pencil tests for neglect. A longitudinal study also showed that severe deficits for contralesional hemispace can still be detected, by reducing target size, also in the putative chronic phases (e.g. $>$ 2 yrs from stroke). In some patients, dual-task manipulations might also lead to the spatial mislocalization of the position of appearance of contralesional targets, in the form of alloesthesia and synchiria. Psychophysiological (ERP and pupil dilation) correlates characterizing the performance of healthy participants under dual-task conditions will be related to the clinical findings above described.
In summary, data show that contralesional spatial awareness can be severely impaired by the request to perform a concurrent, resource-demanding task, regardless of its nature (e.g. visual or auditory). These findings suggest that paper and pencil tests, allowing compensatory strategies to be systematically implemented, are not sufficiently sensitive to reveal the true degree of impairments suffered by patients in the post-acute and chronic phases after a stroke. Only resource-demanding tasks where no compensations is allowed seem to be an appropriate diagnostic option to unveil neglect at these later stages.

Correspondence address: Dr. Mario Bonato, University of Ghent, Department of Experimental Psychology, Henry Duntalaan 2, B-9000 Ghent, Belgium. Tel.: +39 3284341010; E-mail: mario.bonato@ugent.be

### Lecture 2: Evidence for enduring deficits after the resolution of visuo-spatial neglect

Charlotte Russell
*Brunel University, Centre for Cognition and Neuroimaging and Department of Psychology, London, UK*

Neglect frequently resolves spontaneously-however many patients continue to suffer from a number of important and frequently overlooked deficits. These residual deficits involve a number of critical functions associated with parietal cortex: attention; remapping of spatial information across saccades; spatial aspects of long-term memory. First, patients with resolved neglect appear to suffer from a pathological loss of attention capacity. We have shown this by modulating the difficulty of a task at fixation and assessing the impact of this manipulation on perception across the visual field. Patients with right parietal lesions – but without neglect – are impaired in detecting contralesional stimuli only when central task difficulty is high. Further, this impairment extends temporally leading to a spatiotemporal 'attentional blink' in which patients are unable to detect contralesional stimuli for a lag of up to 500 ms (Russell et al, 2004; Russell et al, 2012). Second, after neglect has resolved a considerable number of patients continue to suffer from constructional apraxia, such that they are unable to copy drawings or 3D constructions. We have assessed spatial remapping across saccades in these individuals and have shown a specific impairment in remapping location information, as compared to pattern information (Russell et al, 2010). Recently, in recovered neglect patients with right parietal damage we have assessed whether deficits in spatial representation can be revealed in tests of long-term memory. This has proved to be the case as, despite having no memory deficits in standard neuropsychological tests, when examined with a paradigm designed to examine personal spatial perspective within episodic memory, patients with right parietal damage are greatly impaired. The results of these three lines of research will be discussed in relation to enduring dysfunction following right parietal damage, and how this might relate to the neglect syndrome itself.

Correspondence address: Dr Charlotte Russell, Brunel University – Centre for Cognition and Neuroimaging and Department of Psychology, Uxbridge, UB8 3PH London, UK. E-mail: charlotte.russell@brunel.ac.uk

### Lecture 3: Anatomical predictors of neglect compensation

Marine Lunven[1], Michel Thiebaut de Schotten[2], Christophe Duret[3], Clémence Bourlon[3], Raffaella Migliaccio[4], Gilles Rode[5] and Paolo Bartolomeo[6]
[1]*INSERM-UPMC UMR S 975; Service de Neurorééducation, clinique Les Trois Soleils (Boissise-Le-Roi); Inserm U1028, Paris and Bron, France*
[2]*Inserm U1028 CNRS UMRS292, Equipe ImpaAct; Natbrainlab, Department of Forensic and Neurodevelopmental Sciences, Bron and London*
[3]*Service de Neurorééducation, clinique Les Trois Soleils (Boissise-Le-Roi), France*
[4]*INSERM-UPMC UMR S 975, Paris, France*
[5]*Inserm U1028 CNRS UMRS292, Equipe ImpaAct; Hospices Civils de Lyon, Hôpital Henry Gabrielle, Bron and Lyon, France*
[6]*INSERM, UMRS 975, Brain and Spine Institute; Department of Psychology, Catholic University, Paris and Milan*

Left neglect appears to spontaneously recover during the first weeks after a vascular stroke in about two-thirds of stroke patients. Are there anatomical predictors of spontaneous recovery from (or compensation of) neglect on paper and pencil tests? Previous studies have indicated various grey matter structures, such as the superior and middle temporal gyri, the inferior parietal lobule, the temporo-occipital junction or the basal ganglia, as lesional correlates of chronic neglect. More recently, alteration of microstructural proprieties of white matter in the middle branch of the superior longitudinal fasciculus (SLF II) was associated to persistent neglect (Thiebaut de Schotten et al, Cerebral Cortex 2012). We present a longitudinal study of 37 patients with right-hemisphere damage, tested at the acute/subacute phase and at more than 1 year after the stroke. Each patient underwent MRI with diffusion sequences. Voxelwise statistical analysis of the fractional anisotropy (FA) data was carried out using tract-based spatial statistics. Twenty-seven patients had signs of neglect in the acute phase, of whom only 10 had recovered at follow-up. In acute/subacute neglect, lower FA values occurred along the middle and ventral branches of the right superior longitudinal fasciculus (SLF II and III). In chronic neglect, there was an additional implication of the splenium and of the forceps major. A significant FA decrease in the posterior segment of the arcuate fasciculus also occurred in patients with persistent neglect when compared to patients without neglect in the chronic phase. Our data confirm a key role of fronto-parietal disconnection in the emergence and chronic persistence of neglect. Moreover, these results suggest a role for inter-hemispheric compensation in neglect recovery; on the other hand, chronic neglect may at least in part result from the activity of an isolated left hemisphere.

Correspondence address: Mrs Marine Lunven, INSERM-UPMC UMR S 975; Service de Neurorééducation, clinique Les Trois Soleils (Boissise-Le-Roi); Inserm U1028, 47, bd de l, 75013 Paris and Bron, France. E-mail: marine.lunven@icm-institute.org

### Posters

### P1.01

### Prediction in perception: How foreknowledge changes neural processing

Floris P. de Lange
*Donders Institute for Brain, Cognition and Behavior Radboud University Nijmegen, Netherlands*

One of the most fundamental functions of the human brain is to predict future events on the basis of the recent past. Prediction plays a prominent role in models of seemingly disparate cognitive functions such as perception, motor control, and language processing. We often only become aware of these predictions when they are violated. For example, when opening the door of our house does not result in the expected auditory squeak, we are immediately surprised, whereas if we hear the expected squeak at the expected time, we usually fail to notice it. Although there is ample behavioral evidence for the predictive nature of perception, the incorporation of prior knowledge in the neural processes underlying perception is still poorly understood. In my lecture, I will review recent work on how prior expectations ('priors') about the sensory world change the neural computations that give rise to perception. I will highlight recent neuroimaging data (MEG and fMRI) and methods (multivariate classification techniques) that show how stimulus expectations reduce neural activity in early sensory cortex, but boost the amount of information that can be "read out" from these areas. This suggests that predictions may sharpen the sensory representation, thereby speeding up perception of expected items.

Corresponding author: Floris de Lange, Donders Institute for Brain, Cognition and Behavior, Nijmegen, Netherlands. E-mail: floris.delange@donders.ru.nl

### P1.02

### Memory for Intentions Screening Test: Relationship to other tests of executive and memory functioning

Ondrej Bezdicek[1], Jiri Michalec[2], Tomas Nikolai[1], Sarah Raskin[3] and Evzen Ruzicka[1]
[1] *1st Faculty of Medicine, and General University Hospital in Prague, Charles University in Prague, Department of Neurology and Centre of Clinical Neuroscience, Prague, Czech Republic*
[2] *1st Faculty of Medicine, and General University Hospital, Charles University in Prague, Department of Psychiatry, Prague, Czech Republic*
[3] *Trinity College, Department of Psychology and Neuroscience, Hartford CT, USA*

Prospective memory (PM) tasks require complex mental activity, which is based on, at least, the following processes: monitoring, intention retention, time/place perception, intention recall and execution. Hence, prospective memory (PM) is not a unitary construct. The aim of our study is to test the hypothesis that a clinical tool for testing PM, the Memory for Intentions Test (MIST) will have significant relationships with other measures of executive and memory functioning.
Twenty healthy older adults were investigated using the MIST Czech version (11 F, 9 M; mean age 68 $\pm$ SD5, mean education 16 $\pm$ 2) and a neuropsychological battery: working memory using Digit and Spatial Span and Letter-Number Sequencing (DS, SS and LNS WMS-III); retention using the Auditory Verbal Learning Test (AVLT) and Family Pictures (FP WMS-III) and Memory Capacity Test (MCT); executive functions using the Victoria Stroop Test (VST), Trail Making Test (TMT), verbal fluency (COWAT), Wisconsin Card Sorting Test (WCST-64) and Tower of London (ToL).
There were no significant relationships between age and education and MIST measures. The correlation between Prospective Memory Total (PMT) was significant only with DS, SS forward, MCT and VST derived index C/D (rho > 0,5, all $p < 0{,}05$). The correlation between the retrospective component of the MIST (2- and 15-minute delay) was significant only with MCT and COWAT (rho > 0,5, $p < 0{,}05$). MIST errors scores, namely loss of content and loss of time were significantly related to COWAT (rho = 0,7, ps < 0,05).

These findings indicate that using the MIST Czech version, PMT is predominantly related to measures of interference and sustained attention. Regarding the PM retrospective component as well as PM errors, these are notably related to measures of mental flexibility. This conclusion supports further a non-unitary concept of PM and shows that MIST performance is based on basic memory (interference) and executive (mental flexibility) processes in healthy aging.

Keywords: Memory and Learning, Aging, Neuropsychological Assessment and Psychometrics

Correspondence address: M.A. Ondrej Bezdicek, 1st Faculty of Medicine, and General University Hospital in Prague, Charles University in Prague, Department of Neurology and Centre of Clinical Neuroscience, Katerinska 30, 128-21 Prague, Czech Republic. Tel.: +420 731 478 794; E-mail: ondrejb@yahoo.com

### P1.03

### Visuospatial Boostrapping in older and younger adults

Clara Calia[1], Stephen Darling[1], Richard J. Allen[2] and Jelena Havelka[2]

[1]*Queen Margaret University, Division of Psychology and Sociology, Edinburgh, UK*

[2]*University of Leeds, Institute for Psychological Sciences, Leeds, UK*

BACKGROUND: Recent studies on verbal immediate serial recall (Darling and Havelka, 2010; Darling et al., 2012) shows evidence of the integration of information from verbal and visuospatial short term memory (STM) with long term memory (LTM) representations. This so-called ‘visuospatial bootstrapping’ pattern, in which verbal serial recall is improved when the information is arranged in a familiar spatially distributed pattern, such as a telephone keypad, is consistent with the existence within working memory (WM) of an episodic buffer (EB; Baddeley, 2000).

OBJECTIVE: The present study investigated how visuospatial systems support verbal WM using the visuospatial bootstrapping paradigm across older and younger adult samples, an issue that has not yet been researched.

MATERIALS AND METHODS: Two different samples were recruited: younger adults (18–35 years) and older adults (55–75 years). Each participant was assessed with a brief neuropsychological battery of tests and the assessments of bootstrapping. The latter tasks compared immediate serial recall performance across three visual display conditions: single digit presentations, standard keypad arrays and random (unfamiliar) keypad arrays without suggestion as to what memory strategy to use.

RESULTS: 64 subjects were tested (N = 32 younger adults, N = 32 older adults). The results indicate the presence of a bootstrapping effect in the group of young people and a substantial main effect suggestive of bootstrapping across the two groups.

CONCLUSIONS: Visuo-spatial Bootstrapping is investigated for the first time in a group of older adults. Preliminary analysis indicates a significant improvement in memory in the standard keypad condition across young and older adults.

Keywords: Memory and Learning, Aging, Neuropsychological Assessment and Psychometrics

Correspondence address: Mrs Clara Calia, Queen Margaret University, Division of Psychology and Sociology, Queen Margaret University Drive, Musselburgh,, UK-EH21 6UU Edinburgh, UK. Tel.: +44 07906194175; E-mail: ccalia@qmu.ac.uk

### P1.04

### What are memory binding tests telling us about cognition in healthy ageing? Implications for neuropsychology

Mario Parra[1], Sara Fernández-Guinea[2], Mario A. Parra[1], Anna Frank[3], Maria Luisa-Delgado[3] and Sergio Della Sala[1]

[1]*University of Edinburgh, Psychology, Edinburgh, UK*

[2]*Complutense University of de Madrid, Psychology, Madrid, Spain*

[3]*Hospital La Paz, Health Research Institute, IDIPAZ, Madrid (Spain)*

BACKGROUND: Not all cognitive functions decline with age at the same pace. Cognitive functions which do not decline with age would be interesting for neuropsychologists assessing age-related diseases.
AIM: The present study addressed this issue by studying memory binding in healthy young participants and cognitively intact healthy older adults.
METHODS: Participants (21 younger and 19 older adults) were asked to perform a battery of traditional neuropsychological tests and a battery of tests which investigate binding functions in short-term memory and in long-term memory using recall and recognition procedures.
RESULTS: Traditional neuropsychological tests confirm that the impact of age on cognition is significant for memory, attention, speed of processing and executive control. Similarly, free and cued recall of bindings made of overlearned stimuli (e.g., objects or words), as wells as tasks assessing learning of bound stimuli (i.e., associations), all lead to a pronounced age effect. On the contrary, short-term memory binding tasks, particularly those assessing recognition of novel stimuli, proved insensitive to age.
CONCLUSIONS: These findings have important implications for the selection of memory tests to be used in the assessment of elderly populations at risk for dementia, and suggest that short-term memory binding tasks, being insensitive to age effects, would be an interesting candidate as aid to diagnosis of dementia.

Keywords: Memory and Learning, Aging, Neuropsychological Assessment and Psychometrics

Correspondence address: Dr. Mario Parra, University of Edinburgh, Psychology, 7 George Square, EH8-9JZ Edinburgh, UK. Tel.: +44 0 1316508385; Fax: +44 0 1316513230; E-mail: mprodri1@staffmail.ed.ac.uk

**P1.05**

**Influence of frailty and vulnerability factors in cognitive ageing and its consequences on neuropsychological assessment**

Frederique Bozon-Auclair[1], Audrey Rieucau[2], Malika Gmiz[1], Isabelle Fromentin[1], Samia Lakroun[1], Feriel Menasria[1], Henri Naga[1] and Jean-Philippe David[1]
[1]*Emile Roux Hospital, APHP, Geriatric Unit 3, Limeil-Brevannes, France*
[2]*University Paris Descartes and Emile Roux Hospital, Laboratoire de Psychopathologie et Processus de Santé, EA 4057, Paris, France*

We study here frailty and vulnerability factors identified in the "well-aging consultation" of the geriatric unit. Patients were aged from 47 to 85 years old and consulted in order to obtain advices on their health. During this geriatric consultation, the patient's initial complaint and his results to the IOWA Self-Assessment Inventory and to 5 neuropsychological tests (MMSE, Frontal Assessment Battery, 5 words memory test, clock drawing test and Stroop test) are systematically evaluated. According the frailty and vulnerability profile identified, a more complete neuropsychological assessment is proposed. The data presented here describes the characteristics of 222 patients. The median age is 64 years old and most of the patients ask questions about successful ageing around the beginning of their retirement. Sixty-three percent of patients have a high level of education (A-levels or more). Most of ageing concern is related to presence of a mnesic complaint in 38% of patients, to vascular and/or dementia family history in 26% of cases, to depressed symptoms in 9% of cases, and to being a family caregiver in 8% of patients. After the "well-aging consultation", a more complete neuropsychological assessment was proposed to 19% of the patients. The analyses comparing the groups with and without neuropsychological assessment show that the first group significantly differs from the second one on age (are older), mood (more frequently show depressive symptoms) and quality of life data (feel themselves in poor health). They also differ in all neuropsychological tests: their performances are worse and their processing speed is higher, excepted in the first free recall condition of the 5 words test and the interference Stroop condition. However, the two groups do not differ in educational level and in other IOWA scores. According to these results, we discuss the impact of frailty and vulnerability factors in cognitive ageing and in neuropsychological assessment.

Keywords: Memory and Learning, Aging, Neuropsychological Assessment and Psychometrics

Correspondence address: Dr. Frederique Bozon-Auclair, Emile Roux Hospital, APHP, Geriatric Unit 3, Avenue de Verdun, 1, F-94451 Limeil-Brevannes, France. E-mail: frederique.bozon-auclair@erx.aphp.fr

### P1.06

### The challenge of false memories in rehabilitation: A case report

Alberta Lunardelli[1], Mara Serra[2], Claudia Barbera[2], Marina Mascagni[1], Gianfranco Scrivo[1], Gianfranco Dalla Barba[3] and Valentina Pesavento[1]
[1]*Azienda Ospedaliero-Universitaria Ospedali Riuniti Trieste, Medicina Riabilitativa, Trieste, Italy*
[2]*Università degli Studi di Trieste, Dipartimento di Scienze della Vita, Trieste, Italy*
[3]*Centre de Recherche de l, UMR-S97, Paris, France*

Confabulation, or the production of false memories, is a pervasive disorder that may follow brain damage. Patients are typically unaware of their memory errors and may be so convinced of their truthfulness that they may act upon them. Given the patients' poor insight the persistence of confabulation in chronic cases is generally associated with lessened ability for independent living and thus with increased need for supervision (Mills, Karas, and Alexander, 2006). Despite the great burden posed on caregivers and confabulation's correlation with poor rehabilitation outcomes, there are no systematic studies focusing on its rehabilitation, possibly because most patients eventually stop confabulating,

Here we report a single case intervention study of a 61-year-old woman (IG) who developed a persistent confabulatory syndrome following the rupture of a right internal carotid siphon aneurysm. Consistent with hypotheses suggesting that confabulations may serve important functions of self-coherence and self-enhancement in spite of their poor correspondence to reality (i.e. Fotopoulou, 2008), treatment was tailored on the emotional content of false memories rather than directed on confrontation with her incongruent or inaccurate claims. To evaluate the influence of rehabilitation on IG's tendency to confabulate or on other cognitive domains, a modified version of the Confabulation Battery (Dalla Barba, 1993) together with a comprehensive neuropsychological assessment were administered before and after a 9-months period of individual training sessions. Even though IG maintained a qualitatively similar pattern of confabulations that focused on childhood or adulthood, the amount of false memories decreased over time (from 56.70 to 32.12 mean% of confabulations at the Confabulation Battery). Results may provide clinical support for the efficacy of rehabilitation interventions that take into account, alongside neurocognitive deficits, the significance of emotions in the pathogenesis of confabulation as well as the role of false memories in preserving self-identity and self-esteem.

Keywords: Memory and Learning, Amnesia, Neuropsychological Rehabilitation

Correspondence address: PhD Alberta Lunardelli, Azienda Ospedaliero-Universitaria Ospedali Riuniti Trieste, Medicina Riabilitativa, Piazza Ospitale N. 1, I-34134 Trieste, Italy. E-mail: alberta.lunardelli@gmail.com

### P1.07

### Prosopamnesia: a dysconnexion syndrome?

Claire Bindschaedler[1] and Philippe Maeder[2]
[1]*CHUV, Service de Neuropsychologie et de Neuroréhabilitation, Lausanne, Switzerland*
[2]*CHUV, Service de Radiodiagnostic et Radiologie Interventionnelle, Lausanne, Switzerland*

A few case reports suggest that episodic memory impairment can affect selective categories of visual materials while sparing others, independently of any problem in perceptual processing. Such dissociations have been attributed to either a dysconnexion between initial encoding mechanisms and domain-specific representations (Tippet et al., 2000) or differential damage to segregated paths within the hippocampus (Carlesimo et al., 2001). We report a case of prosopamnesia secondary to a small post-traumatic haemorrhage centred on the collateral sulcus within the right temporal lobe. Overall cognitive functions, face recognition (Benton face matching test, recognition of famous faces) and visual declarative memory (Rey complex figure, Doors test, Camden topographical recognition test, faces subtest of the Rivermead Behavioural Memory Test, faces subtests of the MEM-III, continuous recognition test for words, faces and landscapes) were assessed using mostly well-standardised tools at 3 weeks, 2 and 6 months post-injury. There was no impairment in object, colour and famous face recognition neither in matching of unknown faces. The patient performed normally on the memory tasks mentioned above except on various memory tests involving faces. Moreover, his difference score between faces and landscapes on the continuous recognition task was clearly outside the range of both a large sample of 180 control subjects and a subset of 20 controls matched for age and education (Bindschaedler et al., 1996). Careful MRI examination revealed that the fusiform face area (FFA) and the parahippocampal place area were spared, the lesion lying more anterior and affecting predominantly the parahippocampal cortex. We hypothesise that parahippocampal damage disconnected the FFA from the hippocampal formation while sparing incoming information from other cortical areas involved in processing landscapes or geometric drawings.

Keywords: Memory and Learning, Amnesia, Neuropsychological Assessment and Psychometrics

Correspondence address: Dr. psych. MER Claire Bindschaedler, CHUV, Service de Neuropsychologie et de Neuroréhabilitation, avenue Decker, CH-1011 Lausanne, Switzerland. Tel.: +41 21 3141313; Fax: +41 21 3141319, E-mail: Claire.Bindschaedler@chuv.ch

**P1.08**

**What memory binding functions is the hippocampus responsible for?**

Mario Parra[1], Mario A. Parra[1], Katia Fabi[2], Simona Luzzi[2], Roberto Cubelli[3] and Sergio Della Sala[1]
[1]*University of Edinburgh, Psychology, Edinburgh, UK*
[2]*Polytechnic University of Marche, Clinical Neurology, Ancona, Italy*
[3]*Department of Psychology and Cognitive Sciences, University of Trento, Trento, Italy*

BACKGROUND: The role of the hippocampus in binding information in working memory (WM) is little understood. When complex experiences comprise associations between different pieces of information such as objects and locations (relational binding), the function of the hippocampus is required to hold them in WM. However, recent evidence suggests that if the to-be-associated information leads to the formation of integrated objects such as coloured shapes (conjunctive binding), the hippocampus is less involved in holding temporary representations of these complex events in WM.
AIMS: We investigated the relational and conjunctive binding hypotheses of the hippocampal functions in a patient with right hippocampal damage. Methods. The patient and controls were asked to perform a battery of neuropsychological tasks and a battery of memory binding tasks. Memory binding was assessed by presenting visual arrays of stimuli which consisted of shape-colour relations (shape-colour pairs) or shape-colour conjunctions (coloured shapes). After a short retention interval they were asked to recall the relations or conjunctions or to reconstruct them by selecting the shapes and their corresponding colours from two separate sets.
RESULTS: As compared to healthy controls, the patient was impaired in holding relations of shapes and colours in WM whereas he could retain the conjunctions similarly to controls.
CONCLUSIONS: These results lend support to the role of the hippocampus in supporting WM for inter-item associations but not WM for conjunctions of features which define objects' identity.

Keywords: Memory and Learning, Amnesia, Neuropsychological Assessment and Psychometrics

Correspondence address: Dr. Mario Parra, University of Edinburgh, Psychology, 7 George Square, EH8-9JZ Edinburgh, UK. Tel.: +44 0 1316508385; Fax: +44 0 1316513230; E-mail: mprodri1@staffmail.ed.ac.uk

**P1.09**

**Categorization processing of natural categories in Autism Spectrum Disorder: The effects of typicality.**

Joana Carmo[1], S. Pinhho[2], C. Filipe[3] and J.F. Marques[1]
[1]*University of Lisbon, Faculty of Psychology, Lisbon, Portugal*
[2]*CADIN, Centro de Apoio ao Desenvolvimento Infantil, Cascais, Portugal*
[3]*New University of Lisbon, Faculty of Medical Sciences, Lisbon, Portugal*

Recently, several studies (e.g. Bowler, Graigg and Gardiner, 2008; Bowler, Limoges and Mottron, 2009; Sumiyoshi et al., 2011) show that semantic memory processes differ in key aspects from those of neurotypical individuals and cast some dough on whether semantic memory and categorization processes are truly intact in individuals with Autism Spectrum Disorder (ASD).
In this study we aim at understanding if deficits in semantic processing regarding memory tasks are due to an impairment of semantic processing per se, or due to the use of atypical conceptual categories. For that end we have tested a sample of high-functioning adults with ASD ($n = 22$) and a sample of control participants ($n = 22$) matched for age, IQ and education level on two tasks. First, in order to evaluate the content and composition of conceptual categories we adapted a task from Lambon-Ralph (2010) where errors on typical, atypical and pseudo-typical items can be observed. Additionally, in an implicit categorization task (Release from Proactive Interference paradigm) we have assessed whether recollection from memory can be disrupted by the activation of typical and atypical semantic stimuli.

In the task where the explicit access to the semantic categories is evaluated we have found that individuals with ASD make more errors not only in atypical items, but also in typical and pseudo-typical items. In the release from proactive interference task we found that, together with a overall decrease in memory performance for the ASD group, the amount of the build up (disruption of memory performance within a trial) for typical trials was smaller as compared to the control group, to the level of atypical trials.

Differences found between processing of conceptual categories in individual with ASD and controls are discussed, taking into account the implicit and explicit nature of the different tasks used.

Keywords: Memory and Learning, Autism, Neuropsychological Assessment and Psychometrics

Correspondence address: Dr. Joana Carmo, University of Lisbon, Faculty of Psychology, Alameda da Cidade Universitária, 1649-013-Lisbon Lisbon, Portugal. E-mail: joanacostadocarmo@gmail.com

### P1.10

### Typicality at superordinate and domain level: Implications for understanding category-specific deficits

J. Frederico Marques[1], Andrea Santi[2] and Ana Raposo[3]

[1]*University of Lisbon, Faculty of Psychology, Lisboa, Portugal*

[2]*University College, London*

[3]*University of Lisbon, Lisboa*

GOALS: The present study evaluates the role of typicality in explaining category-specific deficits. Typicality ratings demonstrate the graded nature of category membership; where the better the category exemplar the more features that are shared with other category members and the fewer that are shared with nonmembers. Moreover, knowledge of typical exemplars and shared features is relatively preserved in face of the degradation of semantic knowledge observed in dementia patients. This is at least the case at the superordinate level. This study extends the analysis of typicality data to the domain level, the level at which category-specific deficits are usually reported.

METHODS: We collected typicality norms across both superordinate and domain levels. Ten superordinate categories across 280 items were studied.

RESULTS: Results indicate that the brain categorizes according to animate/inanimate domains rather than living/nonliving and that the domain level of categorization is independent of the more specific superordinate domain, as domain typicality correlates with the prevalence of an item's feature types, but not feature sharing among category members.

CONCLUSION: Typicality at domain level supports a feature type explanation of category-specific deficits where the animate vs. inanimate distinction is better able to capture domain contrasts.

Keywords: Memory and Learning, Dementia, Other Methods

Correspondence address: Mr J. Frederico Marques, University of Lisbon, Faculty of Psychology, Alameda da Universidade, 1649-013 LISBOA, Portugal. E-mail: jfmarques@fp.ul.pt

### P1.11

### Abeta 1–42 diminish the capacity to benefit from performance enhancing memory strategies

Ingo Uttner, Jonas Kösel, Johannes Brettschneider, Hayrettin Tumani and Christine Von Arnim

*Universität Ulm, Neurologische Klinik, Ulm, Germany*

INTRODUCTION: Although Abeta 1–42 is believed to disrupt synaptic function in AD, traditional psychometric tests, however, do not correlate with this marker. Abeta deposits involve at first neocortical areas, especially the frontal lobes that have been associated with the ability to use strategies. This suggests that a strategy-based test procedure such as the Testing-the-Limits paradigm (TtL), which combines the classical measurement of cognitive state (pre test) with the one after exposure to performance-enhancing conditions (post test), should be suitable to depict the relation between the neuronal and the behavioral level.
METHODS: 44 subjects [23 females, 21 males; age (median, interquartil range): 72, 7.5 y] presented to the Memory Clinic with a suspected AD [Mini-Mental-State-Examination (MMSE) score (median, range): 26, 18-30] were assessed. A PC-based recognition paradigm consisting of a pre- and two post-test conditions with an interposed encoding instruction was administered. Cognitive plasticity was operationalized by the recognition failures made in one or both of the post tests. CSF t-tau and Abeta-1-42 levels were determined using a sandwich ELISA (Innogenetics, Zwijndrecht, Belgium).
RESULTS: Abeta ranged from 277 to 1778 (median: 833.5) ng/L, tau from 112 to 1426 (median: 336) ng/L. TtL-failure rates were (median) 11.5 (range: 3–24) in the pre test, 7 (0–24) in post test 1 and 9 (0–24) in post test 2. Correlation analysis (Spearman-Rho) reveals a negative association between abeta and the failure rates in post test 1 ($p = 0.023$). Pre test and post test 2 performance do not correlate with abeta ($p = 0.744$ and $p = 0.083$). No association was found between recognition performance and tau (p-range = 0.104–0.245).
CONCLUSION: Our findings underline the sensitivity of dynamic test procedures as an early indicator for a dysfunction of the frontal lobe networks and corroborate the influence of abeta as a state marker for synaptic plasticity.

Keywords: Memory and Learning, Dementia, Neuropsychological Assessment and Psychometrics

Correspondence address: Prof. Dr. Ingo Uttner, Universität Ulm, Neurologische Klinik, Oberer Eselsberg 45, D-89081 Ulm, Germany. Tel.: +49 0731 177 0; E-mail: ingo.uttner@uni-ulm.de

### P1.12

### Fantastic mnesic confabulation in content is not common in mild Alzheimer's dementia.

Ana Llorente[1], Claire Bindschaedler[2] and Adolfo Jarne[3]
[1]*Benito Menni Psychiatric Hospital, Sant Boi Llobregat, Spain*
[2]*Service de neuropsychologie et de neuroréhabilitation, Hôpital Nestlé CHUV, Lausanne, Switzerland*
[3]*Department of Personality, Barcelona University, Barcelona, Spain*

SPECIFIC GOALS OF THE STUDY: Based on the clinical cases and the other authors, Schnider (2008) considered fantastic mnesic confabulation which have no basis in reality and are intrinsically nonsensical, illogical and implausible in content. Fantastic confabulations was described as a much less common phenomenon that other forms of mnesic confabulations. The aim of this study was to describe the frequency of fantastic confabulations and to provide more empirical evidence on fantastic confabulations in a group of Mild Alzheimer's Dementia.
METHODS: Thirty-three subjects with mild Alzheimer's dementia and 37 elderly without dementia between 65 to 85 years of age participated in this study. We used the Spanish version of Dalla Barba provoked confabulation's interview and other neuropsychological tests or interview: an structured interview of spontaneous confabulations carried out to caregivers; the Spanish version of Grober and Buschke memory test; two tasks of semantic memory and the Spanish version of the Mattis dementia rating scale. We divided the patients group into provoked confabulators and non-confabulators using a statistical criterion.
RESULTS: Thirty percent of the patients compared to eight percent of controls have fantastic confabulations in Dalla Barba provoked confabulation's interview ($p = 0.04$). Provoked confabulators group presented more fantastic confabulations that the elderly without dementia ($p = 0.01$) but no that non-confabulators group ($p = 0.62$); really, when analyzing the results individually, only two patients, provoked confabulators, obtained a number two fantastic confabulations, which is considered above the cut-off.
CONCLUSION: In mild Alzheimer's dementia, fantastic confabulations are not frequents. They seem anecdotal and associated with patients considered provoked confabulators. We would like to note that mnestic fantastic confabulations refer to the content of confabulation, not the methodology of how to get the confabulation (provoked or spontaneous).

Keywords: Memory and Learning, Dementia, Neuropsychological Assessment and Psychometrics

Correspondence address: Dr. Ana Llorente, Benito Menni Psychiatric Hospital, Carrer Doctor Antoni Pujadas, 38, Spain-08830 Sant Boi Llobregat, Spain. Tel.: +34 619181459; E-mail: anallv@copc.es

### P1.13

### Gender specific differences in cognitive profiles of patients with Alzheimer dementia. Results of the Prospective Dementia Registry Austria (PRODEM-Austria)

Gisela Pusswald[1], Lehrner[1], Del-bianco[1], Benke[2], Marksteiner[3], Ransmayr[4] and Schmidt[5]
[1]*Medical University of Vienna, Neurology, Vienna*
[2]*Medical University of Innsbruck, Neurology, Innsbruck*
[3]*Hospital in Hall in Tirol, Psychiatry and Psychotherapy, Hall*
[4]*AKH in Linz, Neurology and Psychiatry, Linz*
[5]*Medical University of Graz, Neurology, Graz*

GOALS: Gender differences have been well documented for healthy individuals in several cognitive tasks. Recent investigations suggested that gender may be an important modifying factor in the development and progression of Alzheimer‘s disease (AD). We here examined gender-specific differences in the pattern of cognitive dysfunction of patients with mild to moderate AD.
METHODS: We examined 417 individuals (mean age 83 (+8.1) years, 59% females) of the prospective registry on dementia in Austria (PRODEM), an ongoing longitudinal multi-center cohort study , conducted in 12 Austrian memory clinics. We analyzed differences in the cognitive profile between male and female AD patients on the CERAD-Plus test battery.
RESULTS: We found gender related differences in the neuropsychological domains of verbal learning, delayed recall, recognition and intrusion errors; the women tended to perform worse than men. There were no significant differences between the two groups in constructional praxis. Comparing the performance of the group of patients with possible to those with probable AD, men and women with probable AD differed only in their verbal learning performance. Controlling the stage, the duration of dementia and the level of education a gender effect of verbal episodic memory remained.
CONCLUSION: There is an interaction between gender and cognitive function, most notable in verbal episodic memory; female patients with AD performed worser on verbal episodic memory than men. This indicates that the gender specificities of neuropsychological functions should be given careful consideration in clinical diagnosis of dementia.

Keywords: Memory and Learning, Dementia, Neuropsychological Assessment and Psychometrics

Correspondence address: Mrs phd Gisela Pusswald, Medical University of Vienna, Neurology, Währinger Gürtel 18-20, a-1090 Vienna, Austria. E-mail: gisela.pusswald@meduniwien.ac.at

### P1.14

### Differences between patients with hydrocephalus and alzheimer on learning and memory

Oget Oktem Tanor
*Istanbul University, IstanbulFaculty of Medicine, Neurology, Istanbul, Turkey*

GOAL: This study aims to investigate the differences between neuropsychological performances of a group of patients with hydrocephalus and a group of Alzheimer disease patients on certain parameters of a Turkish verbal learning and memory test.
METHOD: 233 patients with normal pressure hydrocephalus, 120 patients with early-stage Alzheimer disease, and 779 normal controls were included in this study. A 15-word standardized list learning test named "Oktem Verbal Learning and Memory Procesesses Test" (Oktem Sözel Bellek Surecleri Testi) was performed to all three groups. The evaluated parameters included Learning Score, Highest Learning, Delayed Recall, Delayed Total Remembering (Recall+Recognition) and False Recognition.
RESULTS: Learning Score and Highest Learning Score differed significantly between the three groups. When compared for the Delayed Recall, both patient groups performed significantly lower than normals. As for the Delayed Total Remembering, there was no significant difference between the normals and the patients with hydrocephalus; however, Alzheimer group's performance was significantly lower than both groups. Alzheimer patients were also the only group that had significantly higher False Recognition scores.
DISCUSSION: Our results suggest that the changes in the pressure of CSF in the anterior horns of the lateral ventricules may interfere with the domain of attention in patients with Hydrocephalus, impairing both immediate and delayed recall. As their hippocampus is intact as opposed to Alzheimer's group, the patients with hydrocephalus are able to register all 15 words, and although they have problems with spontaneous recall, they can recognize the remaining words. However, the pathology in hippocampus of the Alzheimer group impairs both recalling and recognizing ability, as demonstrated by their failure to register the words in the first place; the high false recognition scores in this group can also be explained by the same failure.

Keywords: Learning and Memory, Hydrocephalus, Alzheimer

Correspondence address: Prof.Dr. Oget Oktem Tanor, Istanbul University,IstanbulFaculty of Medicine, Neurology, Millet caddesi, Capa, TR-34093 Istanbul, Turkey. Tel.: +90/535 668 60 84; Fax: +90/212/533 85 75; E-mail: ogetoktem@yahoo.com

**P1.15**

**Neuropsychological profiles in familial periventricular nodular heterotopia**

Katrin Kurzbuch[1], Elisabeth Pauli[1], Bernard S. Chang[2], Linsey M. Walker[2] and Burkhard S. Kasper[1]
[1]*Department of Neurology, University of Erlangen-Nürnberg, Epilepsy Center, Erlangen, Germany*
[2]*Dept. of Neurology, Harvard Medical School, Beth Israel Deaconess Medical Center, Boston, USA*

PURPOSE: Periventricular nodular heterotopia (PNH) is a developmental cerebral malformation caused by neural migration failure and often accompanied by epilepsy. Knowledge about cognitive functioning of these patients remains incomplete and is generally described as normal, except for reading impairment. In this study, neuropsychological profiles and anatomical features of an exceptional family with paternal transmission of PNH are reported.
METHOD: Three family members with PNH due to FLNA-mutation and seizures, the father (62), daughter I (31) and daughter II (30), were studied with extensive neuropsychological assessment of intelligence, memory, language, attention and executive functions, as well as structural magnetic resonance imaging (MRI) and volumetrics.
RESULTS: Neuropsychological assessment revealed interesting individual cognitive profiles and significant differences between family members. The father showed above-average intelligence and overall intact cognitive functions; MRI displayed scattered heterotopic nodules. Daughter I showed normal intelligence and specific deficits in working memory and reading; MRI demonstrated mild bilateral PNH. Daughter II showed mental retardation (IQ $< 60$) as well as significant cognitive impairment concerning working memory, attention and language; MRI showed more extensive bilateral PNH. Fractional PNH volumes were 0.68%, 1.18% and 1.97%.
CONCLUSION: Our data illustrated a remarkable variation of cognitive functions within the family and showed a graduation from the unimpaired father to daughter II with significant impairment, contrasting findings about PNH patients being normal or mildly impaired. Cognitive profiles correlated roughly to the degree of PNH, but whether differences are explained by extent of PNH, epilepsy severity, antiepileptic drugs or other factors remains to be clarified. A more systematic approach to neuropsychological testing in patients with PNH is of high importance.

Keywords: Memory and Learning, Epilepsy, Neuropsychological Assessment and Psychometrics

Correspondence address: Mrs Dipl.-Psych. Katrin Kurzbuch, Department of Neurology, University of Erlangen-Nürnberg, Epilepsy Center, Schwabachanlage 6, D-91054 Erlangen, Germany. E-mail: katrin.kurzbuch @uk-erlangen.de

**P1.16**

**Improvement in delayed visual memory in people with mild Alzheimer disease after one year of cognitive stimulation program**

Glòria Chico[1], Noemí Cerulla[2], Maite Garolera[3], Cristina Borras[2], Mariona Rodriguez-Querol[2], Esther Tomas[2] and Carme Civit[2]
[1]*Consorci Sanitari de Terrassa, Hospital Sant Llàtzer, Terrassa, Spain*
[2]*Consorci Sanitari de Terrassa, Hospital Sant Jordi, Terrassa, Spain*
[3]*Consorci Sanitari de Terrassa, Hospital de Terrassa, Spain*

The efficacy of the cognitive stimulation in dementia is well established. The research of cognitive stimulation in Alzheimer's disease has been always criticized because it's assumed that the impairment in memory processes in neurodegenerative diseases is irreversible, but in fact most of the studies are focused in the assessment of verbal memory, leaving aside the assessment of the visual memory. The aim of this study is to compare the performance in delayed visual memory evaluation in people with mild Alzheimer disease after their participation in a cognitive stimulation program for one year provided at a day hospital. The present study consisted in a pre-experimental design with a pre and post intervention measurement that was conducted in a day hospital memory facility. 55 patients with mild Alzheimer disease (30 women, 25 men) were assessed with memory scales from Repeatable Battery of Neuropsychological Assessment (RBANS) before they started the cognitive program and after a year of treatment. The t-test for paired samples in PASW statistics version 20 was used to assess the statistical significance of the differences between memory measures, we set the alpha level to 0.05 and we estimated the effect size (Cohen's d) to measure the magnitude of mean differences. Significant differences were found on the measure of delayed visual memory [t (51) $= -3.14$, $p = 0.03$] with an effect size d that was 0.29, indicative of small effect and no differences were observed on the measure of verbal memory [t (54) $= -0.80$, n.s.] after a year of cognitive training. These results suggest the benefit of cognitive stimulation programs not only in stabilizing cognitive impairment in mild Alzheimer's disease, but also in improving the performance in delayed visual memory. One limitation of this study is the type of design and the results have to be considered preliminary, it needs further investigations to reassure this effect.

Keywords: Memory and Learning, Dementia, Neuropsychological Rehabilitation

Correspondence address: Mrs Glòria Chico, Consorci Sanitari de Terrassa, Hospital Sant Llàtzer, plaça Doctor Robert, 1, S-08221 Terrassa, Spain. Tel.: +34 937367067; Fax: +34 937831555; E-mail: gchico@cst.cat

### P1.17

### The conditions affecting cognitive functions in children with focal epilepsy

Tinatin Gogberashvili and Yuri Mikadze
*Department of clinical psychology, The Russian National Research Medical University named after N.I. Pirogov (RNRMU), Moscow, Russia*

Cognitive deficits are one of the most frequent symptoms in pediatric epilepsy. Research on the factors affecting cognitive disorders in children with focal epilepsy is of great importance for diagnostics, prognosis of cognitive development, and rehabilitation.

In neuropsychological research (A. Luria syndrome analysis) cognitive functions were evaluated in children and teenagers with focal epilepsy at the age of 6–16 years of age. Qualitative and quantitative analysis of different factors influencing cognitive functions (such as age of the child, age of the disease onset, epileptic center localization, etiology of the disease) was carried out.

Presence and severity of some of the cognitive symptoms in focal epilepsy depends on the age of the child. The comparison of neuropsychological research results in two age groups: 6–10 and 11–16 year old proved that there is irregular reduction of the severity of certain symptoms. The reduction is particularly evident in symptoms related to the first brain unit (arousal and inactivation). However regardless of the epileptic center localization the symptoms related to verbal-acoustic and visual-spatial perception does not decrease. Neither do the symptoms of audio and visual memory disorders (the second brain unit – modal and material specific factors).

More severe cognitive disorders were observed in children with early manifestation of the disease (in the age groups of 0–3 and 3–6). This regularity is clearly seen in the juxtaposition of the symptoms connected to the deficiency of all three brain units. In children with later manifestation of the disease the symptoms were less pronounced.

The disease etiology (symptomatic temporal and idiopathic temporal) influences the specifics and severity of cognitive disorder. Cognitive disorders in idiopathic focal epilepsy are less pronounced than in symptomatic epilepsy.

Keywords: Memory and Learning, Epilepsy, Neuropsychological Assessment and Psychometrics

Correspondence address: Mrs Tinatin Gogberashvili, The Russian National Research Medical University named after N.I. Pirogov (RNRMU), Department of Clinical Psychology, Ostrovityanova st.1, +7-117997 Moscow, Russia. E-mail: tinatina2004@mail.ru

### P1.18

### The timescale of accelerated long-term forgetting in patients with Transient Epileptic Amnesia

Serge Hoefeijzers[1], Michaela Dewar[1], Christopher Butler[2], Sergio Della Sala[1] and Adam Zeman[3]
[1]*University of Edinburgh, Human Cognitive Neuroscience, Edinburgh, United Kingdom*

[2]University of Oxford, Nuffield Department of Clinical Neurosciences, Oxford, United Kingdom
[3]University of Exeter, Cognitive and Behavioural Neurology, Peninsula Medical School, Exeter, United Kingdom

Accelerated Long-term Forgetting (ALF) is the process whereby initially intact memories are forgotten more rapidly than usual over periods longer than standard testing intervals (> 30 minutes). ALF has been associated with epilepsy, in particular with Transient Epileptic Amnesia (TEA). Previous work shows that ALF is apparent 24 hours – 1 week after initial memory acquisition. It is unclear however if ALF is apparent even earlier. This study investigates when ALF first becomes apparent. 16 TEA patients and 16 matched controls were presented twice with 4 categorical word lists, each of which was recalled at a different delay interval: 30 minutes, 3 hours, 8 hours and 24 hours. One week after acquisition participants were asked to recall all four word lists again and to complete a word recognition test. TEA patients' initial learning of word lists and their recall at the 30-minute delay interval were normal. Recall became impaired 3 hours after acquisition and declined significantly until the 8-hour delay interval. Recall performance of TEA patients remained poorer at the 24-hours and 1-week delay interval. Though, the 8-hour to 1-week forgetting pattern was similar to that of controls. That is, no forgetting was observed over the 8-hour to 24-hour interval whilst recall performance over the 24-hour to 1-week interval dropped similarly between groups. Moreover, TEA patients performed significantly poorer on the word list recognition test one week after acquisition. These findings show that ALF in TEA becomes apparent within hours of acquisition in the case of word recall. ALF cannot be accounted for by disrupted memory mechanisms during nocturnal sleep but is more likely due to a deficit in early memory consolidation.

Keywords: Memory and Learning, Epilepsy, Neuropsychological Assessment and Psychometrics

Correspondence address: Mr Serge Hoefeijzers, University of Edinburgh, Human Cognitive Neuroscience, 7 George Square, UK-EH8 9JZ Edinburgh, United Kingdom. E-mail: s.hoefeijzers@sms.ed.ac.uk

### P1.19

### Implicit sequential learning in patients with epilepsy-interim results

Anke Hermsen[1], Yu-Ming Chen[1], Silke Leonhard[2], Marcus Belke[1], Susanne Knake[1] and Felix Rosenow[1]
[1]*Philipps-Universität and UKGM Marburg, Neurology, EZH, Marburg, Germany*
[2]*Neurologische Klinik Westend, Bad Wildungen*

Introduction Epilepsy is frequently accompanied by cognitive deficits, especially memory. Explicit and implicit memory (ExM, ImM) form the long term memory. Mesial temporal lobe (mTL) structures -especially the hippocampus- are important for ExM; hippocampal sclerosis (HS) often leads to memory deficits in mTL epilepsy (mTLE). The same structures are discussed as contributing to ImM. Surprisingly, an animal model of mTLE-rats showed better sequential learning (SL, a subform of ImM) performance than control animals. Data on ImM in mTLE-patients is lacking. SL, a form of ImM, is investigated here with SRTT (Serial Reaction Time Task) in epilepsy patients with (+)/without (−) mTLE.

Method In two studies we investigated 1) preoperative patients with focal epilepsy (TLE+HS, TLE-HS, extraTLE (eTLE)), as well as 2) postoperative mTLE-patients (selective Amygdalohippocampectomy (sAHE); sAHE+ Gyrus parahippoc.; TL-Resection; eTLE; planned: resp. $n = 84$, $n = 96$). Included are clinical variables, standardized neuropsychological diagnostic and SRTT.

Results of an interim analysis Patients ($n = 29$, study 1: $n = 17$, study 2: $n = 12$): female 55%, 14 +HS, 11 –HS, 4 unknown with respect to HS. Overall, we found learning in SRTT (F = 6,941, $p < 0.001$). Performance on first vs. last sequence-bloc revealed a significant interaction effect (F(1,27) = 4,82, $p = 0.048$) meaning that eTLE achieve higher speed than TLE. Explorative analysis: ExM (t-Test): +HS ($n = 7$) were worse than–HS ($n = 8$) while showing similar ImM. There was no gender or hemispheric difference in ImM.

Discussion The ImM can be measured by SRTT and all patient groups showed a learning effect. Patients with eTLE showed better performance in ImM than with TLE. TLE-patients with HS are, by explorative analysis, worse in ExM than TLE without HS, but not in ImM. Future Outlook: To correlate this data with MRI-lesion/postoperative size of resection to identify necessary structures for ImM and predict the risk for postoperative deficits in ImM.

Keywords: Memory and Learning, Epilepsy, Neuropsychological Assessment and Psychometrics

Correspondence address: Mrs Anke Hermsen, Philipps -Universität and UKGM Marburg, Neurology, EZH, Baldingerstr, D-35043 Marburg, Germany. E-mail: hermsen@staff.uni-marburg.de

### P1.20

### Minimising sensory stimulation boosts memory over 7 days in patients with amnestic MCI and early Alzheimer's Disease

Jessica Alber, Sergio Della Sala and Michaela Dewar
*University of Edinburgh, Psychology, Human Cognitive Neuroscience, Edinburgh, UK*

A brief rest following new learning can boost memory over short delays in patients with memory disorders, including amnestic Mild Cognitive Impairment (aMCI) and mild Alzheimer's Disease (AD). Here we investigated whether a brief rest following new learning could boost memory over much longer delays in such patients. 14 patients with aMCI/mild AD and 14 healthy controls were presented with 2 short stories. One story was followed by a 10-minute period of wakeful resting (minimal sensory stimulation), the other story was followed by a 10-min period during which participants played a spot-the-difference game (sensory stimulation). Retention of the two stories was assessed after a standard delay (15–30 minute) as well as after a much longer delay (7 days). As in our previous studies, patients were able to recall significantly more material after 15–30 min when story learning had been followed by the wakeful-resting period than when story learning had been followed by the spot-the-difference period. Importantly, this memory boost via a post-learning rest persisted over the 7-day delay. This long-lived memory enhancement via post-learning resting is remarkable given that patients with aMCI/ mild AD often forget new material over just a few minutes. These results strengthen our hypothesis that much of the severe forgetting in aMCI and mild AD is associated with excessive memory interference during early memory consolidation stages, caused by new sensory stimulation. When such sensory stimulation is reduced immediately after learning, new memories can be consolidated to a much higher degree and retained over the long term. This long-term persistence suggests that later consolidation stages are at least partially intact in patients with aMCI and mild AD. Minimal sensory stimulation techniques could be useful in the rehabilitation of memory disorders, by allowing patients to retain new key material over the short term and the long term.

Keywords: Memory and Learning, Amnesia

Correspondence address: Mrs Jessica Alber, University of Edinburgh, Psychology, Human Cognitive Neuroscience, 7 A Gayfield Square, UK-EH1 3NT Edinburgh, UK. Tel.: +44 07972327683; E-mail: j.l.alber @sms.ed.ac.uk

### P1.21

### GOOD OR BAD responders? Markers of clinical response to cognitive stimulation therapy in Alzheimer's disease

Maite Garolera[1], Maria del Mar Martinez[2], Noemí Cerulla[3], Gloria Chico[3], Laura Igual[4], Maria del Mar Vila[4] and Carme Civit[3]
[1]*Hospital de Terrassa-Consorci sanitari de Terrassa, Neuropsychology Unit, Terrassa-Barcelona, Spain*
[2]*Consorci Sanitari de Terrassa, Brain, Cognition and Behaviour Research Group Unitat d'Investigació biomèdica Universitat Autònoma de Barcelona-CST (UIB), Terrassa (Barcelona), Spain*
[3]*Consorci Sanitari de Terrassa, Sant Jordi Day Hospital, Terrassa, Barcelona, Spain*
[4]*Universitat de Barcelona, Departament Matematica Aplicada, Barcelona, Spain*

There is a long tradition of psychological therapies for people with dementia, but rarely have they been evaluated in terms of clinical response. The purpose of this study was to classify patients as "responder" or "non-responder" to a cognitive stimulation program (CSP) offered to patients with Alzheimer's disease (AD) on the basis of clinical significance criteria and to identify which factors may differentiate both groups of responders. 62 patients with mild AD (56%women, mean age at baseline 75 SD 6.4) participating, to a one year day hospital CSP, undergone an assessment at the beginning of the intervention with: Geriatric Depression Scale (GDS), Barthel scale (BS), Lawton and Brody Scale (LB) and memory scales from Repeatable Battery Assessment (RBANS). The response to CSP was assessed by a measure of time related changes on the

Mini Mental State Examination (MMSE after one year treatment minus baseline); this allowed distinguishing Good from Bad responders. T-test independents samples were used to assess whether groups of responders differed on baseline measures (alpha level = 0.05). 24% of patients were classified following the assessment of change (MMSE $\geqslant$ 2 points increase after completing the training) as clinical Good responders. At baseline, Non-responders had not a significant higher depression ($p = 1$); less functional capacity: BS ($p = 0.40$), LB ($p = 0.057$) or worse cognitive functioning: delayed story memory ($p = 0.53$), delayed list learning ($p = 0.41$), MMSE ($p = 0,16$) than responders. In conclusion: in 24% of mild AD CPS is effective in slowing down the apparent clinical progression of AD. However, mood, functional status and memory previous treatment outcomes could not discriminate eventual "good" and "poor" responders after 12 months of cognitive treatment. Further research is needed to characterize the profile of those patients who improve in response to treatment; it will be useful to ameliorate the rehabilitation intervention and its clinical efficacy.

Keywords: Memory and Learning, Neurodegenerative Diseases, Neuropsychological Rehabilitation

Correspondence address: Mrs Doctor Maite Garolera, Hospital de Terrassa-Consorci sanitari de Terrassa (CST), Neuropsychology Unit, ctra. torrebonica s/n, Spain-08227 Terrassa-Barcelona, Spain. Tel.: +34 7310007, E-mail: mgarolera@cst.cat

**P1.22**

**Cognitive dysfunction in Spinocerebellar Ataxia Type 3: A longitudinal analysis**

Sandra Roeske[1], Ina Filla[2], Stefan Heim[3], Katrin Amunts[3], Christoph Helmstaedter[4], Ullrich Wüllner[2], Michael Wagner[5], Thomas Klockgether[1] and Martina Minnerop[2]

[1]*DZNE, Clinical Research, Bonn, Germany*

[2]*University Hospital Bonn, Neurology, Bonn, Germany*

[3]*Research Centre Juelich GmbH, Institute of Neuroscience and Medicine, Juelich, Germany*

[4]*University Hospital Bonn, Epileptology, Bonn, Germany*

[5]*University Hospital Bonn, Psychiatry and Psychotherapy, Bonn, Germany*

The spinocerebellar ataxias (SCAs) are inherited neurodegenerative disorders, characterized by prominent ataxia and cerebellar atrophy. SCA type 3 (SCA3) may present additionally with a broad spectrum of non-cerebellar symptoms. Cognitive dysfunction in SCA3 is common, but it is unknown whether cognition deteriorates with disease progression. We therefore studied cognitive function in SCA3 prospectively.

Eleven patients with SCA3 (male 7, age M = 40.0, SD 9.3 years (y)) and a matched control group were assessed using an extensive neuropsychological test battery. SCA3 patients were retested after 3.5 $\pm$ 0.4 y and rated according to the Scale for the Assessment and Rating of Ataxia (SARA). The neuropsychological test battery covered verbal and figural memory, verbal fluency, naming, reaction time, interference, motor control, and evaluation of depressive symptoms. We performed comparison in cognitive tests between patients and controls, and within the patient group by repeated measurement multivariate analysis of variance (MANOVA; pcorr $< 0.05$).

At baseline, only fine motor coordination differed between SCA3 patients and controls. At follow up, SCA3 patients had compared to controls significant cognitive impairment in verbal learning. Within the patient group MANOVA revealed a significant deterioration of ataxia symptoms as well as cognitive function, including verbal learning, verbal memory, figural memory and motor control during the follow-up period. Fine motor coordination correlated with the increasing SARA-score. No correlation between the deterioration in ataxia and in cognitive tests or an increase of depressive symptoms was observed.

The cognitive deterioration with disease progression suggests that cognitive dysfunction is an integral part of SCA3 and cannot be attributed to progressive cerebellar ataxia as the memory tests did not require motor responses. It can be explained either by extracerebellar pathology or by disruption of cerebellar-cerebral circuitries.

Keywords: Memory and Learning, Neurodegenerative Diseases, Neuropsychological Assessment and Psychometrics

Correspondence address: Dr. Sandra Roeske, DZNE, Clinical Research, Ernst-Robert-Curtius-Str. 12, D-53117 Bonn, Germany. Tel.: +49 228 43302 814; Fax: +49 228 43302 801; E-mail: Sandra.Roeske@dzne.de

### P1.23

### Neural effect of memory training in children

Regula Everts[1], Ines Mürner-Lavanchy[1], Barbara Ritter[1], Schroth Gerhard[2] and Maja Steinlin[1]
[1]*Children's University Hospital, Inselspital, Division of Rehabilitation, Development and Neuropediatrics, Bern, Switzerland*
[2]*University Hospital Inselspital, Institute for Diagnostic and Interventional Neuroradiology, Bern, Switzerland*

It is still unclear whether memory training decreases or increases the effect size of a functional activation during a memory task. The aim of the present study was to investigate differences in percent signal change (PSC) of the visual working memory network after memory training in children born preterm. Formerly very preterm born children (7–12 years) underwent a memory training using restitution (20 $\times$ 10 min of working memory training, $n = 8$) or compensation (4 $\times$ 60 min of memory strategy training, $n = 9$), or no memory training (controls, $n = 5$). Before the training (TP1) and after the training (TP2), memory functions were assessed and fMRI of the visual working memory (WM) network was performed. PSC was calculated over four regions of interest (ROI: left/right frontal; left/right parietal).
Both training groups significantly improved memory performance in different memory domains (resti: verbal recognition $p = 0.03$; visual WM $p = 0.05$; comp: verbal learning $p = 0.004$; verbal recall $p = 0.03$; visual learning $p = 0.004$; visual recall $p = 0.002$; verbal WM $p = 0.004$). No changes in memory performance and no differences in PSC occurred in controls between TP1 and TP2. In both training groups, PSC was significantly lower at TP2 in the left frontal ROI (resti: $p = 0.008$; comp: $p = 0.047$) and in the right frontal ROI (resti: $p = 0.008$). The magnitude of PSC difference between TP1 and TP2 correlated negatively with changes in WM performance between TP1 and TP2 (right frontal $r = -0.493$, $p = 0.031$, left parietal $r = -0.508$, $p = 0.027$), indicating more activation decrease in children with stronger improvement of WM performance after the training.
Memory training can lead to a decrease of the intensity of cerebral activation during a visual WM task, most pronounced in children whose WM performance clearly improved through the training. These data point towards a practice-related change of the cerebral WM network after a short memory training and provide evidence for the high functional plasticity of the child's brain.

Keywords: Memory and Learning, Other Disorder/ Samples, Functional and Structural Neuroimaging/ NIRS

Correspondence address: Dr. Regula Everts, Children's University Hospital, Inselspital, Division of Rehabilitation, Development and Neuropediatrics, Inselspital, CH-3010 Bern, Switzerland. E-mail: regula. everts@insel.ch

### P1.24

### Body Schema manipulation through the activation of the "body" Working Memory

Gerardo Salvato, Anna Sedda and Gabriella Bottini
*University of Pavia, Department of Brain and Behavioural Sciences, Pavia, Italy*

Body Representations (BR) are dynamic, depending on the integration of internal sensory signals and environmental information. The Body Schema (BS) is an unaware component of BR concerning body parts position. Working Memory (WM) permits to manipulate short term information of different categories of stimuli, including body parts. Given the high domain specificity of WM, one might hypothesize the existence of a selective WM storage for BR. We used a dual-task paradigm to explore whether these two components might interact. In the Stocking Test (ST), that concerns body exploration, 12 healthy blindfolded subjects were required to search for targets attached to their body surface while performing a Backward Spelling Task (BST). Half of the subjects were required to manipulate body parts names and half objects names. Our data demonstrate that the group performing the WM task with body related material shows a greater accuracy at the ST ($F(1,9) = 5.941$; $p = 0.038$; partial $\eta 2 = 0.398$). On the contrary, the group performing the object WM test shows an opposite pattern, with a negative influence of the dual task on the accuracy at the ST. These preliminary results suggest that body domain-specific WM positively influences visual imagery skills associated with body exploration, indicating a semantic access to BR. This specific interaction could be taken into account for selective rehabilitation of body representation impairments.

Keywords: Memory and Learning, Other Disorder/ Samples, Other Methods

Correspondence address: Mr Gerardo Salvato, Department of Brain and Behavioural Sciences, University of Pavia, Piazza Botta, 11, IT-27100 Pavia, Italy. E-mail: gerardosalvato@gmail.com

**P1.25**

**Cognitive function in facioscapulohumeral muscular dystrophy**

Gyurgyinka Gergev[1], Ágnes Herczegfalvi[2], Veronika Kercagi[3] and László Sztriha[4]
[1]*Department of Pediatrics, Semmelweis University Budapest, Budapest, Hungary*
[2]*Semmelweis University, Budapest*
[3]*National Institute of Environmental Health, Budapest*
[4]*University of Szeged*

OBJECTIVE: Cognitive impairment is a well-known feature of several muscular disorders, such as Duchenne muscular dystrophy and myotonic dystrophy. Facioscapulohumeral muscular dystrophy is the third most common muscular dystrophy; however our knowledge about its cognitive features is rather poor. It is an autosomal dominant muscle disorder caused by contraction of a critical number of D4Z4 repeats on chromosome 4q35. Previous MRI studies have suggested central nervous system involvement with grey matter loss in the prefrontal cortex and white matter hyperintensities. The aim of this work was to explore the cognitive pattern in four patients with facioscapulohumeral muscular dystrophy.
METHODS: Four patients (2 boys and 2 girls) with mean age of 15 years were included in the study. The diagnosis was confirmed by molecular genetic tests. The patients did not have any major psychiatric or somatic illness. The cognitive functions were assessed in detail by means of a neuropsychological battery.
RESULTS: The cognitive profile varied between normal abilities and mild intellectual disability. All patients however showed deficit in verbal working memory and they had reduced learning capacity.
CONCLUSION: In agreement with a few previous data in the literature some degree of heterogeneity can be observed in the cognitive abilities of patients with facioscapulohumeral muscular dystrophy and they can be affected by learning difficulties. Further studies and long-time follow up are required in order to shed light on the relationship between molecular defects, cerebral structural abnormalities and neuropsychological features in this disorder. Recognition of special pattern of disabilities can help designing proper treatment strategy.

Keywords: Memory and Learning, Other Disorder/ Samples, Neuropsychological Assessment and Psychometrics

Correspondence address: Mrs Gyurgyinka Gergev, Department of Pediatrics, Semmelweis University Budapest, Práter 26. 4/5., Hungary-1083 Budapest, Hungary. Tel.: +36 302262644; E-mail: gyurgyinka@ gmail.com

**P1.26**

**Automatic vs. strategic mechanisms in prospective memory: An fMRI study**

Francesco Barban[1], Giovanni Augusto Carlesimo[2], Francesco Scalici[1], Emiliano Macaluso[3], Carlo Caltagirone[2] and Alberto Costa[1]
[1]*IRCCS Santa Lucia Foundation, Clinical and behavioural neurology, Rome, Italy*
[2]*IRCCS Santa Lucia Foundation; University of Rome "Tor Vergata", Clinical and Behavioural Neurology; Institute of Neurology, Rome, Italy*
[3]*IRCCS Santa Lucia Foundation, Neuroimaging Laboratory, Rome, Italy*

INTRODUCTION: Prospective memory (PM) is a cognitive process that allows the realization of intentions in the future and the hypothesis was advanced that it may be subserved by automatic or strategic mechanisms. Neuroimaging data document that PM performance is related to the activity of frontal and parietal brain regions. However, poor is known about brain networks differentially involved in above PM mechanisms. This study was aimed at investigating the functional neural correlates of automatic and strategic processes within PM with functional magnetic resonance (fRMI).
METHOD: Fourteen healthy subjects were administered an event-based PM paradigm in which automatic vs. strategic demands were manipulated. This was made by fitting conditions in which the PM cues were congruent (focal conditions) or incongruent (nonfocal conditions) with the ongoing task. In particular, each trial included the presentation of two strings. In half of the trials they were real words, while, in the other half, one string was a neologism. Moreover, the two strings could represent in the centre the same or different syllables. The ongoing activity was, in half blocks, a lexical decision task and, in the other half, a syllables matching task. We also manipulated the PM cues that could be in half blocks real words and, in the other half, syllables.
RESULTS: We analysed behavioural and imaging data comparing focal vs. nonfocal conditions. As expected, behavioural results showed faster response times ($p < 0.001$) and better accuracy ($p < 0.05$) on focal than nonfocal conditions. Whole brain fMRI analyses highlighted a main effect of congruency (nonfocal > focal, p-corr < 0.05) selectively in the left intraparietal sulcus.
CONCLUSIONS: The present findings document, for the first time, a specific recruitment of left parietal cortex in nonfocal PM paradigms, likely suggesting a role for this region in the mediation of strategic mechanisms subserving PM.

Keywords: Memory and Learning, Other Disorder/ Samples, Functional and Structural Neuroimaging/ NIRS

Correspondence address: PhD Francesco Barban, IRCCS Santa Lucia Foundation, Clinical and behavioural neurology, Via Ardeatina 306, I-00179 Rome, Italy. Tel.: +39 06 51501547; E-mail: f.barban@hsantalucia.it

**P1.27**

### Serial order memory, attentional control and the learning of English expert words by native Cantonese speakers

Brendan Weekes[1], Jose Cheng[2], Loan Vuong[2] and Steve Majerus[3]
[1]*University of Hong Kong, Laboratory for Communication Sciences, Hong Kong, Hong Kong*
[2]*University of Hong Kong, Hong Kong, China*
[3]*University of Liege, Belgium*

Past research in neuroimaging has shown a strong association between phonological short-term memory and vocabulary learning in first and second (L2) language. Specifically, memory for serial order independently predicts the acquisition of a second language (French) for tertiary students and also activates specific regions of the parietal cortex in studies of German-French bilingual speaking undergraduates [1,2]. These studies also suggest a possible role for attention in vocabulary learning. The aim of this study was to examine the role of serial order memory and attention in the learning of English expert words among Cantonese native speakers. Participants were students in the Division of Speech and Hearing Sciences at Hong Kong University. There were 2 phases of data collection. In Phase 1, expert word learning (via a lexical decision task), attentional control, verbal STM, general cognitive ability, visuo-spatial memory and general language knowledge were assessed. In Phase 2, expert word learning was re-assessed six months later. The results indicate that serial order memory is the strongest predictor of L2 expert word learning as well as number of hours exposed to academic materials. Associations were also observed between attentional control and STM measures and between attentional control and new word learning. We will also report new data from neuroimaging (fMRI, EEG and TMS) showing an association between memory for serial order, new word learning and brain activation in the left inferior parietal lobe.

References

[1] Majerus, S., Poncelet, M., van der Linden, M. & Weekes, B.S. (2008a). Lexical learning in bilingual adults: the prevailing importance of serial order short-term memory mechanisms. Cognition, 107, 395-419.
[2] Majerus, S., Belayachi, S., De Smedt, B., Leclercq, A.L., Martinez, T., Weekes, B., Maquet, P. (2008b). Neural networks for short-term memory for order differentiate high and low proficiency bilinguals. NeuroImage, 42(4), 1689–713.

Keywords: Memory and Learning, Other Disorder/ Samples, Other Methods

Correspondence address: Mr Professor Brendan Weekes, University of Hong Kong, Laboratory for Communication Sciences, University of Hong Kong, D-1111 Hong Kong, China. E-mail: Weekes@hku.hk

**P1.28**

### Predictors of prospective memory impairment in pulmonary diseases

Marta Witkowska
*University of Gdansk, Institute of Psychology, Gdansk, Poland*

Pulmonary disorders, such as asthma (AS) and chronic obstructive pulmonary disease (COPD) are chronic diseases which lead to respiratory failure. Decreased transportation of oxygen to the brain may affect mental efficiency, especially prospective memory (PM), which impairment prevents the patient from living an independent and fulfilling life.
Prospective memory, PM, is a set of processes or abilities to formulate, store and implement the purposes and intents in a set time frame or situation and its functioning can be influenced by various factors, related both to the respiratory failure, the number of cigarettes smoked per day and the use of mnemonics.
The main objective of the research was to establish the general characteristics of PM in pulmonary patients. And as a result - to specify the hypothetical predictors of their prospective memory impairment.
Several tests (i.e. a quasi-experiment based on the Cambridge Prospective Memory Test & BDI) were administered to adult patients with allergic rhinitis (AL, $n = 27$), AS ($n = 27$), COPD ($n = 32$) and control subjects ($n = 30$).
COPD group (mean = 21.97; SD = 5.75) compared with other groups, obtained lower scores in PM tasks ($F(3,112) = 38.55$; $p < 0.001$). At the same time the higher the rate of FEV1 (less respiratory failure), fewer cigarettes smoked per day and lower level of depressive symptoms of COPD (R2 = 0.71; $F(1,30) = 16.5$; $p < 0.001$) and AS patients (R2 = 0.77; $F(2,27) = 44.32$; $p < 0.001$) the better functioning of the PM.
Also the use of mnemonics explained 32% of PM variation in COPD ($F(1,30) = 13.85$; beta = 0.57; $p < 0.01$) and AS patients ($F(1,28) = 13.41$; beta = 0.57; $p < 0.01$) and 43% in the control group ($F(1,25) = 18.62$; beta = 0.65; $p < 0.001$).
There is a significant relationship between the PM and type of illness. The level of respiratory failure, the number of cigarettes smoked per day and the use of mnemonics directly affects prospective memory of pulmonary patients and their ability to cope with the disease.

Keywords: Memory and Learning, Other Disorder/ Samples, Neuropsychological Assessment and Psychometrics

Correspondence address: Mrs Marta Witkowska, University of Gdansk, Institute of Psychology, Bazynskiego 4, 80-952 Gdansk, Poland. Tel.: +48 6046885 02; E-mail: marta.witkowska@ug.edu.pl

### P1.29

### Low frequency rTMS on the right parietal cortex at retrieval increases recognition memory in healthy subjects

Patrizia Turriziani[1], Massimiliano Oliveri[2], Daniela Smirni[2], Renata Mangano[2] and Lisa Cipolotti[2]
[1]*Deparment of Psychology, University of Palermo, Palermo, Italy*
[2]*Department of Psychology, University of Palermo, Palermo, Italy*

Neuroimaging and lesion studies have led to contrasting findings regarding the potential role of the parietal lobe in episodic memory. Imaging studies strongly suggest an important participation of the posterior parietal cortex (PPC) in episodic memory, whereas lesion studies are not conclusive at this regard.
Using off-line 1 Hz rTMS paradigm, we conducted 2 experiments to investigate the role of PPCs in recognition memory. Real or sham rTMS were applied over the left or the right PPC (P3 and P4 of the 10–20 EEG system) of healthy subjects before encoding (Experiment 1) and just before retrieval (Experiment 2) of forced-choice non verbal recognition memory tasks.
rTMS over the left and the right PPC at encoding did not modulate memory performance. Conversely, rTMS over the right ($p < 0.02$) but not left ($p < 0.3$) PPC at retrieval significantly improved recognition memory performance.
These findings suggest that PPC is involved at retrieval but not at encoding of recognition memory. Inhibition of the right PPC at retrieval enhances memory function in healthy subjects. These findings are in line with our recent work showing that rTMS inhibition of the right dorsolateral prefrontal cortex (DLPFC) enhanced recognition memory in healthy subjects as well as in patients with mild cognitive impairment.
We propose that the right DLPFC and the right PPC may be part of a neural network recruited during recognition memory retrieval. Temporary inhibition of these regions enhances memory performance suggesting a cognitive benefit when there is a reduced activity in these regions during recognition memory tasks.
Our findings may give support to an inhibitory control account of the interactions between the DLPFC – PPC and the medial temporal lobe during retrieval of memory information.

Keywords: Memory and Learning, Other Disorder/ Samples, TMS/TCDS

Correspondence address: Mrs Dott Patrizia Turriziani, Deparment of Psychology, University of Palermo, Viale delle Scienze,Ed 15, I-90144 Palermo, Italy. E-mail: patrizia.turriziani@unipa.it

### P1.30

### Improving children's cognitive performance – effect of a memory strategy training in children born very preterm

Regula Everts[1], Barbara Ritter[2] and Maja Steinlin[2]
[1]*Children's University Hospital, Division of Rehabilitation, Development and Neuropediatrics, Bern, Switzerland*
[2]*Children's University Hospital, Inselspital, Division of Rehabilitation, Development and Neuropediatrics, Bern, Switzerland*

To examine the effect of memory strategy training on different aspects of memory in children born very preterm and to determine whether there is a generalization of the training effect to non-trained functions. The influence of individual factors such as age and performance level on the training success will be determined. In a randomized, controlled and blinded clinical trial, 46 children born very preterm (aged 7–12 years) were allocated to a memory strategy training (MEMO-Training, $n = 23$) or a control group ($n = 23$). Neuropsychological assessment was performed before, immediately after the training and at a 6-month follow-up. In the MEMO-Training, five different memory strategies were introduced and practiced in a one-to-one setting (4 hour-long training sessions over 4 weeks, 20 homework sessions).

A significant training-related improvement occurred in trained aspects of memory (verbal and visual learning and recall, verbal working memory) and in non-trained functions (inhibition, mental arithmetic). No performance increase was observed in the control group. At six months follow-up, there was a significant training-related improvement of visual working memory. Age and performance level before the training predicted the training success significantly.

Teaching memory strategies is an effective way to improve different aspects of memory but also non-trained functions such as inhibition and mental arithmetic in children born very preterm. Age and performance level influence the success of memory strategy training. These results highlight the importance of teaching children memory strategies to reduce scholastic problems.

Keywords: Memory and Learning, Other Disorder/ Samples, Neuropsychological Rehabilitation

Correspondence address: Dr. Regula Everts, Children's University Hospital, Division of Rehabilitation, Development and Neuropediatrics, Inselspital, CH-3010 Bern, Switzerland. E-mail: regula.everts@insel.ch

### P1.31

### Cognitive decline in patients with heart failure

Friederike Thiel[1], Stefan Frisch[2], Andrej Teren[3], Frank Beutner[3], Joachim Thiery[4], Arno Villringer[5] and Matthias L. Schroeter[4]
[1]*University Hospital Leipzig, Germany, Dayclinic for Cognitive Neurology, Leipzig*
[2]*University Hospital Frankfurt*
[3]*Heart Center Leipzig*
[4]*University Hospital Leipzig*
[5]*Max Planck Institute for Human Cognitive and Brain Sciences, Leipzig*

About 30–60% of heart failure patients suffer from cognitive impairments; most frequent are memory and attention deficits followed by slowed psychomotor speed and impaired executive functions. The N-terminal prohormone of brain natriuretic peptide (NT-proBNP) is valuable for the diagnosis and the prognosis of heart failure. Despite prior research that has identified associations between cognitive deficits and the NT-proBNP, findings are not conclusive. In this study, we thoroughly examined subjective as well as objective cognitive deficits in heart failure and compared patients with acute increased NT-proBNP plasma levels to those with normal values.

46 patients diagnosed with heart failure underwent a broad neuropsychological assessment including test for attention, memory, executive functions as well as self-report measures. A cardiological examination and a blood sample were included. The neuropsychological test results were converted to standard values based on an age matched normative control group. NT-proBNP was rated as increased or normal according to the laboratory's normative reference values.

Patients reported worsening of cognitive and mental domains predominantly in drive (25%), anterograde memory (20%), mood (15%) and short-/long-term concentration (10%). In psychometric tests heart failure patients showed increased deficits in short-term and long-term memory, in complex problem solving as well as in complex attention demands. The 21 subjects with acute increased NT-proBNP values did not differ significantly in cognitive measures from subjects with current normal NT-proBNP values.
Both subjective and objective results replicate the existence of cognitive impairments among heart failure patients. Increased NT-proBNP values do not seem to be closely associated with cognitive deficits.

Keywords: Memory and Learning, Other Disorder/ Samples, Neuropsychological Assessment and Psychometrics

Correspondence address: Mrs Friederike Thiel, University Hospital Leipzig, Germany, Dayclinic for Cognitive Neurology, Liebigstraße 16,, D-04103 Leipzig. E-mail: thiel@cbs.mpg.de

**P1.32**

**Encoding task and retrieval cue effects on the neural networks involved in source memory**

Sofia Frade, Mara Alves, J Frederico Marques and Ana Raposo
*University of Lisbon, Faculty of Psychology, Lisbon, Portugal*

Remembering the context, or source, of a particular event is a fundamental ability that is highly susceptible to disruption in aging and brain disorders. Source memory depends on processes that take place both at encoding and retrieval, but the conditions that improve such memory are not well-established. This fMRI study aims to explore how the retrieval cue and the encoding task interact during source memory retrieval. In the encoding phase healthy participants performed a semantic task (pleasant word?) or a perceptual task (more than 6 letters?) in a set of words. During retrieval the probe was presented with either a semantic cue (pleasant task?) or a perceptual cue (letter task?). Participants had to decide if that probe appeared in that specific task. The results revealed an encoding task effect during context retrieval. Retrieving probes previously encoded in a semantic manner was associated with significant activation in medial temporal and medial frontal regions, whereas the retrieval of probes encoded in a perceptual manner was linked to posterior occipital activations. Critically, this effect was only found when source memory was elicited by the semantic retrieval cue, but not by the perceptual retrieval cue. These results indicate that only the semantic cue induces activations that are congruent with the neural processes instantiated during encoding. This suggests an important relationship between retrieval cue and encoding task and highlights the critical role of semantic cues in source retrieval. Overall, these findings may elucidate how memory difficulties in amnesic patients and older adults can be partially overcome when the information to be retrieved is prompted by a semantic cue.

Keywords: Memory and Learning, Other Disorder/ Samples, Functional and Structural Neuroimaging/ NIRS

Correspondence address: Mrs Sofia Frade, University of Lisbon, Faculty of Psychology, Alameda da Universidade, 1649-013 Lisbon, Portugal. E-mail: sofia.t.frade@gmail.com

**P1.33**

**Air flow limitation during sleep and verbal memory in Fibromyalgia**

A. Campabadal[1], A. Soler[2], M.J. Masdeu[3], L. Vigil[3], C. Galisteo[3], M. Larrosa[3] and M. Jódar[3]
[1]*Universitat Autònoma de Barcelona, Psicologia Clínica i de la Salut, Barcelona, Spain*
[2]*Universitat Autònoma de Barcelona*
[3]*Corporació Sanitària Parc Taulí, Sabadell*

OBJECTIVE: Some studies have described slight memory difficulties in fibromyalgia patients (FM) in comparison with healthy population. Executive deficits and problems with interference management seem to explain these differences. Besides, many works have found alterations in FM sleep, such as respiratory anomalies. The aim of this study is to examine the presence of air flow limitation (AFL) during sleep and to asses verbal memory in FM.
METHOD: The sample includes: 30 FM, 25 chronic pain patients (CP) and 26 healthy subjects (HS). Verbal memory was assessed using Rey Auditory-Verbal Learning Test (AVLT) and Logic Memory from WMS-III (LM). AFL percentage was measured during an all night polysomnography.
RESULTS: Statistical analysis using an ANOVA with a Bonferroni post-test shows no significant differences between groups in: AVLT short-term, AVLT long-term, AVLT recognition, LM learning slope, LM retention percentage and LM recognition. Significant differences were found between HS and FM in: AVLT first trial ($p = 0.02$), AVLT total learning ($p = 0.02$), AVLT interference ($p = 0.05$); And between HS and both patients groups in: LM immediate memory ($p = 0.00$, $p = 0.05$), LM delayed recall ($p = 0.00, p = 0.00$). FM scores were slightly lower in all variables. Finally, significant differences were found between FM and both other groups in AFL ($p = 0.00$, $p = 0.00$). A general lineal model reveals impact of AFL in simple immediate memory.

CONCLUSION: The current work finds greater AFL during sleep in FM in comparison with CP and HS. In addition, FM shows difficulties in interference management, in immediate verbal memory and in a verbal memory task that requires executive functions. Pain could explain strategy problems in verbal memory, since CP and FM share this difficulty. However, AFL during sleep could be related with FM specific verbal memory deficit. New researches should investigate the relation between sleep disorders and cognitive impairment in FM.

Funded by the Fundació La Marató de TV3

Keywords: Memory and Learning, Other Disorder/ Samples, Neuropsychological Assessment and Psychometrics

Correspondence address: Mrs A. Campabadal, Universitat Autònoma de Barcelona, Psicologia Clínica i de la Salut, Edifici B Campus UAB, D-08193 Barcelona, Spain. E-mail: akad5@hotmail.com

**P1.34**

**First evidence of the relationship between memory and hippocampal volume in dyskinetic cerebral palsy**

Júlia Ballester-Plané[1], Olga Laporta-Hoyos[1], Elida Vázquez[2], Ignacio Delgado[2], Leire Zubiaurre-Elorza[1], Idoia Marqués-Iturria[1], Ana Narberhaus[1], Alfons Macaya[2], Pilar Póo[3], Mar Meléndez[2], Teresa Castelló[2], Violeta Tenorio[4], Mª Eugenia Russi[3], Mª Dolors Segarra[1] and Roser Pueyo[1]
[1]*Universitat de Barcelona, Spain*
[2]*Hospital de la Vall d\Hebron, Barcelona, Spain*
[3]*Hospital de Sant Joan de Déu, Barcelona, Spain*
[4]*Hospital Clínic i Provincial, Barcelona, Spain*

The importance of the hippocampus for declarative memory processes is firmly established. Poor memory has been related to small hippocampal volume in several neurologic disorders. However, the relationship between memory and hippocampus has not been studied in cerebral palsy (CP). Moreover, the hippocampus can be bilaterally damaged after perinatal asphyxia; one of the most frequent causes of dyskinetic CP. But this type of CP is poorly studied from a neuropsychological perspective due to motor severity and communication difficulties. The aim of this study is to investigate if there is a relationship between memory performance and hippocampal structure in people with dyskinetic CP.

Fifteen term-born subjects diagnosed with dyskinetic CP secondary to perinatal asphyxia (mean age: 26.2; sd: 12.04) underwent a neuropsychological test and a T1-weighted resonance magnetic imaging. Specifically, we selected a memory test in which verbal communication was not necessary: Pattern Recognition Memory test of Cambridge Neuropsychological Test Automated Battery (CANTAB). Standardized measures were used in order to control for age effects. Hippocampal structure was analyzed using voxel-based morphometry technique after applying a region of interest through the SPM program. Family-wise-error correction for multiple comparisons was used ($p < 0.05$).

Results showed a significant positive correlation between memory measures and hippocampal volumes. Long term memory bad performance was associated with a greater atrophy in left hippocampus (left hippocampus: $t = 5.02, p = 0.01$, cluster size = 143 voxels), and short term memory bad performance was related to a greater atrophy in hippocampal regions bilaterally (left hippocampus: $t = 4.04$, $p = 0.042$, cluster size = 5 voxels; right hippocampus: $t = 4.14$, $p = 0.041$, cluster size = 7 voxels). These preliminary results show a relationship between memory and hippocampal volume in term-born subjects with dyskinetic CP secondary to perinatal asphyxia.

Keywords: Memory and Learning, Other Disorder/ Samples, Functional and Structural Neuroimaging/ NIRS

Correspondence address: Mrs Júlia Ballester-Plané, University of Barcelona, Psychiatry and Clinical Psychobiology, Campus Mundet. Edifici de Ponent. Pg. Vall d\'Hebron 171, D-08035 Barcelona, Spain. E-mail: julia.ballester.plane@gmail.com

### P1.35

### Relationship between working memory and academic performance of children

Gustavo Siquara, Lais Vilas-Boas, Cassio Lima, Paulo Nascimento, Carolina Sunnano and Neander Abreu
*Federal University of Bahia, Psychology Institute, Salvador, Brazil*

The working memory (WM) refers to our ability to store and manipulate information for a period of time. The cognitive model used was the multicomponent Baddeley. There is evidence of extensive relationship between WM and learning. The aim of this study was investigate the relationship between WM and academic performance. Individual neuropsychological assessment was performed following specific protocol. All testing occurred on school shift in classes of children participants. The study included 116 children aged 7 to 12 (M = 9.82, SD = 1.36) years, 58 females from two private schools in Salvador, Bahia, Brazil. To evaluate the academic performance were used Academic Performance Test (APT) with subtests in reading, writing and arithmetic. In the evaluation of the components of WM and short-term memory were used backward and forward Digit Span and the backward and forward Corsi block-tapping. One test was used to assess the attention concentrated under the name "test of visual attention". The statistical tests used were linear regression analysis (stepwise) and Pearson correlation. Results showed that, best model to explain the academic performance predictors were Corsi Block Backward, Digit Span Backward, Reaction Time and Digit Span Forward tasks. It was checked the hypothesis of non-multicollinearity and verified that constructs were independent (VIF $< 10$ and Tolerance $> 0{,}20$. The explained variance of the model for academic performance was $\Delta R = 0{,}46$. The correlations between the components of WM showed high and significant correlations with the APT. WM test (Digit Span Backward + Corsi Block Backward) and APT had correlation ($r = 0.63^{**}$). The results indicate initially that WM is a good predictor of academic performance. Initially this study is consistent with others in the point where the WM as a predictor of learning or the potential for learning. It appears that WM is a process that is the basis of the ability to learn.

Keywords: Memory and Learning, Other Disorder/ Samples, Neuropsychological Assessment and Psychometrics

Correspondence address: Mr Master Gustavo Siquara, Federal University of Bahia, Psychology Institute, Salvador-Bahia, Bra-44701035 Salvador, Brazil. Tel.: +55 7193699262; E-mail: gustavosiquara@hotmail.com

### P1.36

### Contribution of working memory to skills learning in schizophrenic patients.

Arantzazu Herran-Boix[1], Bernardo Pardo-Ayuso[2], Mireia Gonzalez-Rodriguez[1], Sergio Bodas[1], Mar Ariza[3] and Maite Garolera[1]
[1]*Hospital de Terrassa-Consorci Sanitari de Terrassa, Neuropsychology Unit, Terrassa-Barcelona, Spain*
[2]*Parc Sanitari Sant Joan de Déu., Department of Mental Health, Sant Boi de Llobregat, Spain*
[3]*Brain, Cognition and Behaviour Research Group. Universitat Autònoma de Barcelona-CST (UIB), Unitat d'Investigació biomèdica, Terrassa-Barcelona, Spain*

Accumulating evidence indicates that working memory (WM) deficit is a core feature of schizophrenia (SC). On the other hand, some studies examining non-declarative learning in patients with SC have demonstrated impaired skills learning (SL). Recent studies have shown that WM contributes to motor learning process, although this relationship has not been evidenced in schizophrenic patients. In the present study, we tested the hypothesis that WM impairment contributes to performance on motor learning for schizophrenic patients. The participant sample consisted of 105 patients who met DSM-IV criteria for SC (74% males; 93% right-handed; mean age 32 (SD 8.2); mean years of SC evolution 9 (SD 8.2); mean estimated premorbid intelligence-Vocabulary scalar score 10.4 (SD 2.6) and mean education years 9.8 (SD 2.7). Patients were all receiving antipsychotic medication and they were clinically stable (Positive and Negative Symptoms Scale total score mean 58.3 (SD 15.8)) at the time of testing. Using computers we assessed WM with a subcomponent of the Test of Attentional Performance (TAP) and SL with a rotor pursuit task (RPT). Partial correlations were used to control the influence of age, illness evolution, premorbid IQ and education (Alpha level = 0.01). We identified significant correlation between performance on TAP, specifically in missing and errors of WM (standard score), and total time of contact on RPT. Missing were correlated with time of contact on RPT [$r = 0.254$, $p = 0.010$] and with time out of contact [$r = -0.255$, $p = 0.010$]. Errors were correlated with time of contact on RPT [$r = 0.325$, $p = 0.001$] and with time out of contact [$r = -0.326$, $p = 0.001$]. The relationship found between WM and SL suggests that in SZ failure in WM processes affects acquisition of new motor skills. These findings are consistent with functional neuroimaging data demonstrating overlapping neural substrates (dorsolateral prefrontal cortex and striatal circuitry) for the performance of both cognitive tasks.

Keywords: Memory and Learning, Psychopathology, Neuropsychological Assessment and Psychometrics

Correspondence address: Mrs Arantzazu Herran-Boix, Hospital de Terrassa-Consorci Sanitari de Terrassa, Neuropsychology Unit, Crta.Torrebonica s/n, 08227 Terrassa-Barcelona, Spain. Tel.: +34 937310007 (1557); E-mail: aherran@cst.cat

### P1.37

### Do similar memory functions show similar recovery patterns after stroke?

Sarita Soikkeli, Eija-Inkeri Ruuskanen, Laura Nurmi and Mervi Jehkonen
*University of Tampere, School of social sciences and humanities, psychology, Tampere, Finland*

OBJECTIVE: This study assesses the recovery of memory functions in stroke patients during a six-month follow-up and compares the results of different memory tests. We wanted to find out whether similar memory functions (e.g. verbal and visual memory) recover similarly after stroke. We also wanted to find out whether there is a positive correlation between memory test scores during the follow-up phase.
METHODS: The study group consisted of 50 consecutive patients with a first brain infarct. Neuropsychological and neurological examinations were performed on two occasions: at the acute phase within six days of onset and at six months. Severity of stroke was evaluated by a neurologist using the National Institute of Health Stroke Scale. An MRI examination was carried out to determine the localization of the infarct. The memory functions examined were immediate and delayed verbal and visual memory. They were assessed with Logical Memory and the Word List subtests of the WMS-III, Digit Span subtest of the WAIS-III and the Rey-Osterrieth Complex Figure test.
RESULTS: During the follow-up different immediate verbal memory functions showed similar patterns of recovery, as did different delayed verbal memory functions. Both verbal and visual delayed memory functions showed similar patterns of recovery. There was also a positive correlation between the memory test scores at the acute phase and at six-month follow-up, indicating successful recovery of memory functions in the whole patient group during the follow-up phase.
CONCLUSION: It seems that similar memory functions show similar patterns of recovery after ischemic stroke. In general the results suggest that memory functions recover within six months of stroke. Interestingly, some memory functions (e.g. repetitive memory) did not seem to recover in the same way as other memory areas. This area needs more investigation with larger patient groups.

Keywords: Memory and Learning, Stroke, Neuropsychological Assessment and Psychometrics

Correspondence address: Mrs Sarita Soikkeli, University of Tampere, School of social sciences and humanities, psychology, Tammelan puistokatu 14-16 B 89, FIN-33100 Tampere, Finland. Tel.: +35 8400651714; E-mail: sarita.soikkeli@uta.fi

### P1.38

### Memory impairment in stroke patients without functional disability

Marja Hietanen[1], Hanna Jokinen[1], Raija Ylikoski[1], Tarja Pohjasvaara[2] and Timo Erkinjuntti[2]
[1]*Helsinki University Central Hospital, University of Helsinki, Unit of Neuropsychology, Department of Neurology, Helsinki, Finland*
[2]*Helsinki University Central Hospital, University of Helsinki, Department of Neurology, Helsinki, Finland*

The aim of our study was to investigate the frequency and nature of memory impairment in a cohort of patients with first-ever ischemic stroke and without functional disability.
In Helsinki Stroke Ageing study 409 consecutive patients ages 55–85 years were evaluated with a comprehensive neuropsychological examination three months post-stroke. The following cognitive domains were assessed: attention and executive functions, memory, visuoconstructive and spatial skills, abstract thinking, aphasia, neglect, agnosia, calculation, reading and writing. The modified Rankin Scale (mRS) was used to evaluate the degree of functional disability and dependence in the daily activities.
Of 131 patients with first-ever stroke (N = 332) and without any functional disability (mRS 0–1, mean age 68.8) being able to carry out all usual activities 65 (50%) showed memory impairment. These clinically well recovered patients with memory impairment did not significantly differ from those without memory impairment in age, sex, education or vascular risk factors. The patients with memory impairment performed significantly worse than normal controls in all memory components studied (immediate and delayed recall, implicit, semantic and working memory). The patients with memory impairment had significantly ($p < 0.000$) more decrements also in attention and executive functions (82%) as well as in visuoconstructional and spatial skills (77%) than those without memory.
In conclusion, half of the first-ever stroke patients without any functional disability have memory impairment. Memory impairment in these patients is often a part of a larger cognitive symptomatology including deficits especially in attention and executive functions. This refers to the importance of neuropsychological evaluation in stroke patients without any functional disability taking into account multiple cognitive domains at risk.

Keywords: Memory and Learning, Stroke, Neuropsychological Assessment and Psychometrics

Correspondence address: Dr. Marja Hietanen, Helsinki University Central Hospital, University of Helsinki, Unit of Neuropsychology, Department of Neurology, Paciuksenkatu 21/P.O.Box 302, FIN-00270 Helsinki, Finland. Tel.: +358 9 471 73871; Fax: +358 9 471 74088; E-mail: marja.hietanen@hus.fi

**P1.39**

**Recalling the past and imagining the future interferes with verbal memory consolidation**

Michael Craig, Michaela Dewar and Sergio Della Sala
*University of Edinburgh, Human Cognitive Neuroscience, Edinburgh, Scotland*

BACKGROUND: People remember a higher level of new information when learning is followed by a brief period of wakeful rest than if they attend to other new incoming external information during this time (Cowan et al. 2004; Della Sala et al. 2005; Dewar et al. 2007, 2009, 2012). This benefit is thought to reflect superior memory consolidation of recent memory traces during post-learning wakeful rest (Dewar et al. 2009, 2012). In everyday life we also use our memory system in an internally directed manner, e.g. recalling the past and imagining future scenarios.
AIM: We explored whether internal memory activities interfere with the consolidation of recent verbal memory traces.
METHOD AND RESULTS: We conducted two experiments in which word learning was followed by three nine minute delay conditions: (I) Wakeful resting, (II) Visual search task, (III) Cued recall/imagination task succeeded by a surprise delayed recall test for learned words. A significantly lower level of material was retained when new learning was followed by both visual search and cued recall/imagination tasks compared to resting wakefully. Two follow up experiments demonstrated that presentation of familiar sound cues without instruction to imagine, automatically triggered the recollection of autobiographical memories and imagination of future scenarios providing a significantly higher level of interference than both wakeful rest and presentation of inconsequential "bang" cues.
CONCLUSION: Various activities of the episodic memory system are detrimental to memory consolidation of recent verbal memory traces. When such activities are minimised, an enhancement in memory retention is observed.

Keywords: Memory and Learning

Correspondence address: Mr Michael Craig, University of Edinburgh, Human Cognitive Neuroscience, 7 George Square, EH8-9JZ Edinburgh, Scotland, UK. Tel.: +44 07805645317; E-mail: M.Craig-4@sms.ed.ac.uk

**P1.40**

**Is dementia prediction better with age and educational level corrections? The PAQUID cohort study**

Hind Mokri[1], Céline Meillon[1], Catherine Helmer[1], Jean Bouisson[2], Jean-François Dartgues[1] and Hélène Amieva[1]
[1]*Bordeaux Segalen University, INSERM U 897, Bordeaux, France*
[2]*Bordeaux Segalen University, EA 4139, Bordeaux, France*

There is a well-known effect of demographic variables, such as age and education level on cognitive scores in older population. Cognitive scores of individuals with advanced age or low education level could be spuriously considered as impaired. Thus, it is common to assume that corrections for age and schooling level would improve classification accuracy and reduce error in diagnosis. However, age and education level are major risk factors in dementia. Thus, contrary to common intuition regarding the use of demographic corrected norms for diagnosis of dementia, some studies suggest that corrections for demographic variables do not improve dementia classification accuracy. The purpose of our study is to assess dementia prediction accuracy comparing cognitive test scores with and without age and education corrections. Analyses were conducted in a sample of 1104 non-demented subjects aged 65 years and over, gathered from the fourth follow-up of the PAQUID study, a population-based cohort study conducted in Southern-Western France. Within the 1104 subjects, 92 became demented in the fifth follow-up. Cognitive performances were assessed using the Isaacs Set test, a verbal fluency test and dementia prediction accuracy was assessed using sensibility and specificity indexes. The sample was stratified by age (< 80 years versus 80 years and over) and education level (without school certificate versus school certificate and over). Results show that for lower educated and older subjects, dementia is predicted with a better specificity when the scores are corrected for age and education but with a better sensitivity when the scores are not corrected. However, for younger subjects with high level of education, dementia is predicted with a better sensibility when the scores are corrected but a better specificity when the scores are not corrected. Thus, proceeding to demographic corrections reduces dementia prediction accuracy for subjects with advanced age and low education level.

Keywords: Other Functions/Disorders, Dementia, Neuropsychological Assessment and Psychometrics

Correspondence address: Mrs MSc Hind MOKRI, Bordeaux Segalen University, INSERM U 897, 146 rue Leo Saignat, D-33076 Bordeaux, France. Tel.: +33 557571173; E-mail: hind.mokri@isped.u-bordeaux2.fr

### P1.41

### Spatial working memory impairments predict recancellation behaviour in right brain damaged patients

Robert McIntosh[1] and Nicoletta Beschin[2]
[1]*University of Edinburgh, Psychology, Edinburgh, UK*
[2]*Hospital S. Antonio Abate, Clinical Neuropsychology Unit, Rehabilitation Department, Gallarate, Varese, Italy*

Right brain-damaged patients with neglect omit items on the left side in cancellation tasks, and may also re-visit and re-cancel items on the right. Recancellations and target omissions are exacerbated in 'invisible' cancellation tasks, in which no mark is left on visited items. It has been proposed that recancellation behaviour arises from impairments of spatial working memory, which prevent patients from keeping track of visited locations. This is consistent with the finding of non-lateralised spatial working memory impairments in patients with neglect. However, it has not yet been demonstrated that recancellation rates are predicted by spatial working memory impairments across patients. This is important, because a range of alternative factors might also explain recancellation (e.g. rightwards magnetic attraction, perseveration, disordered search planning). We tested 27 patients with right hemisphere stroke on touch-screen-based visible and invisible cancellation tasks. As expected, recancellations and omissions increased dramatically under invisible conditions. Moreover, the magnitude of the increase correlated negatively with performance on a non-lateralised assessment of spatial working memory (rho (27) $= -0.69$; $p < 0.0005$). This supports the proposal that spatial working memory problems are a principal driver of recancellation behaviour.

Keywords: Visuospatial Abilities, Stroke, Neuropsychological Assessment and Psychometrics

Correspondence address: Dr. Robert McIntosh, University of Edinburgh, Psychology, 7 George Square, UK-EH89JZ Edinburgh, UK. Tel.: +44 131 6503444; E-mail: r.d.mcintosh@ed.ac.uk

### P1.42

### Training of working memory in healthy elderly subjects – a randomized controlled trial

Juliane Weicker[1], Nicole Hudl[2], Enely Marichal[2], Karsten Müller[3], Jöran Lepsien[3], Sabrina Trapp[4], Stephan Frisch[5] and Angelika Thöne-Otto[2]
[1]*Max-Planck-Institute for Human Cognitive and Brain Sciences Leipzig, Neurology, Leipzig, Germany*
[2]*University of Leipzig, Clinic of Cognitive Neurology, Leipzig, Germany*
[3]*Max-Planck-Institute for Human Cognitive and Brain Sciences Leipzig, Germany*
[4]*Max-Planck-Institute for Human Cognitive and Brain Sciences Leipzig, Psychology, Leipzig, Germany*
[5]*J.W. Goethe University, Neurology, Frankfurt/Main, Germany*

Working memory plays a key role in many higher order cognitive functions, hence its functioning affects substantially the quality of everyday life (Johansson and Tornmalm, 2012). A recent meta-analysis by Weicker and Thöne-Otto (2012) showed that training can sustainably increase working memory capacity and produce beneficial effects in cognitive control as well as the appraisal of everyday life functioning. However, despite the wide range of existing interventions there is still a lack of theoretically motivated training programs. Furthermore, crucial task characteristics and the concrete mechanisms of transfer are largely unknown. To approach these issues, in cooperation with HASOMED GmbH we developed a computer-based training program which is able to differentially exercise specific aspects of working memory (e.g. storage or manipulation of items) in a playful way on the basis of a card game. The effectivity of the training was evaluated in a randomized controlled trial with clinically healthy elderly subjects aged 60 to 75. In a pre-/post-test design the adaptive working memory training was compared to a placebo training and a no-training condition. Training took place 3 times a week for 45 minutes over one month. Before and after the training all subjects accomplished a broad neuropsychological test battery which assessed the effect of the training on working memory functions as well as on related domains. By the time of abstract submission data collection was still in progress, but it will be completed in July so that we will be able to present statistically valid results.

Keywords: Other Functions/Disorders

Correspondence address: Mrs Dipl. Psych. Juliane Weicker, Max-Planck-Institute for Human Cognitive and Brain Sciences Leipzig, Neurology, Stephanstr. 1a, D-04103 Leipzig, Germany. Tel.: +49 3419940171; E-mail: weicker@cbs.mpg.de

### P1.43

### Computerized training of working memory for patients with acquired brain injuries – a randomized controlled trial

Juliane Weicker[1], Enely Marichal[2], Nicole Hudl[2], Karsten Müller[3], Jöran Lepsien[3], Sabrina Trapp[4], Stephan Frisch[5] and Angelika Thöne-Otto[2]
[1]*Max-Planck-Institute for Human Cognitive and Brain Sciences Leipzig, Neurology, Leipzig, Germany*
[2]*University of Leipzig, Clinic of Cognitive Neurology, Leipzig, Germany*
[3]*Max-Planck-Institute for Human Cognitive and Brain Sciences Leipzig, Germany*
[4]*Max-Planck-Institute for Human Cognitive and Brain Sciences Leipzig, Psychology, Leipzig, Germany*
[5]*J. W. Goethe University - Neurology, Frankfurt/Main, Germany*

Working memory often is impaired as a consequence of brain injury with major impact on everyday functioning and quality of life (Johansson and Tornmalm, 2012). A recent meta-analysis by Weicker and Thöne-Otto (2012) indicated that especially patients benefit from training, showing not only increased working memory capacity but also improved cognitive control and reduction of disorder symptoms. However, despite an increasing number of studies within the last years, there are only few trials with brain injured patients and most of them lack of methodical quality. Furthermore, current training programs provide no adequate adaptivity mechanisms for patients and fail to offer a theoretical framework why transfer effects may occur. To approach these issues, in cooperation with HASOMED GmbH we developed a computer-based training program which is able to differentially exercise specific aspects of working memory (e.g. storage or manipulation of items) and adapts difficulty in very fine steps to be appropriate for patients. Training effectivity was evaluated in a randomized controlled trial with brain injured patients suffering from stroke or traumatic brain injury. Subjects were included when brain injury occurred at least three month ago and when they showed reduced working memory performance. Exclusion criteria were severe memory or language deficits as well as constricting acute diseases. The adaptive working memory training was compared to a placebo training condition in a pre-/post-test design. Both trainings took place 3 times a week for 45 minutes over one month, the measurements before and after the training consisted of a broad neuropsychological test battery which assessed training effects on working memory as well as on related domains. Transfer to everyday life functioning was assessed by multiple questionnaires. By the time of abstract submission data collection was still in progress, but it will be completed in July so that we will be able to present statistically valid results.

Keywords: Other Functions/Disorders, Traumatic Brain Injuries

Correspondence address: Mrs Dipl. Psych. Juliane Weicker, Max-Planck-Institute for Human Cognitive and Brain Sciences Leipzig, Neurology, Stephanstr. 1a, D-04103 Leipzig, Germany. Tel.: +49 3419940171; E-mail: weicker@cbs.mpg.de

**P1.44**

**Cognitive deficits after stroke: What do medics know about neuropsychology?**

Elizabeth Fowler[1], Simon Hart[2], Sergio Della Sala[1] and Robert D McIntosh[1]
[1]*University of Edinburgh, Human Cognitive Neuroscience, Psychology, Edinburgh, UK*
[2]*Royal Infirmary of Edinburgh, Stroke Unit, Edinburgh, UK*

GOALS: Despite the widespread and often persistent cognitive problems experienced by stroke patients, neuropsychological screening is not routine procedure in acute stroke care. Given the marginal status of cognitive assessment, we were interested in determining what levels of neuropsychological knowledge are possessed by medics and health professionals who work with stroke patients. In particular, it was hypothesised that the less immediately observable cognitive problems, particularly those associated with right hemisphere lesions, would have lower recognition levels than left hemisphere language deficits.
METHOD: To investigate this, a questionnaire was devised and distributed at a major UK stroke conference. Respondents were asked to identify which of 20 cognitive symptoms are more commonly associated with either the right or left cerebral hemisphere, or non-lateralised. Six physical symptoms were included as control questions. Data was collected from 226 medics and health professionals, the majority of whom work directly with stroke patients.
RESULTS: As anticipated, the most widely recognised cognitive symptom was aphasia, with 95% of respondents correctly identifying it as a typically left-hemisphere problem. This was followed by neglect and anosognosia, with 64% of respondents associating these with right hemisphere lesions. However, many other cognitive symptoms were poorly recognised, in some cases only at chance levels. Aphasia aside, this was equally true of right and left hemisphere symptoms, with no overall significant difference between conditions.
CONCLUSION: These preliminary findings suggest that there does seem to be a lack of knowledge of cognitive symptoms among medical professionals and that many neuropsychological findings are not being into assimilated into clinical practice.

Keywords: Stroke

Correspondence address: Mrs Elizabeth Fowler, University of Edinburgh, Human Cognitive Neuroscience, Psychology, 7 George Square, EH8-9JZ Edinburgh, UK. E-mail: s1148032@staffmail.ed.ac.uk

**P1.45**

**The cerebellar role in cognition: Evidence from voxel based morphometry and diffusion imaging**

Giusy Olivito[1], Michael Dayan[2], Valentina Battistoni[3], Silvia Clausi[4], Marco Molinari[5], Maria Leggio[4] and Marco Bozzali[3]
[1]*Department of Psychology, "Sapienza"' University of Rome; Ataxia Laboratory, Santa Lucia Foundation, Rome, Italy, Rome, Italy*
[2]*Neuroimaging Laboratory, Santa Lucia Foundation, Department of Radiology, Weill Cornell Medical College, Rome, New York, Italy, United States of America*
[3]*Neuroimaging Laboratory, Santa Lucia Foundation, Rome, Italy*
[4]*Department of Psychology, "Sapienza" University of Rome, Ataxia Laboratory, Santa Lucia Foundation, Rome, Italy*
[5]*Ataxia Laboratory Santa Lucia Foundation, Rome, Italy*

Over the last decades, the cerebellar role in cognition has been extensively investigated. From an anatomical point of view, strictly contralateral interconnections link the cerebellum to the cerebral cortex. It has been hypothesized that in this network the cerebellum plays a modulatory role in cognitive functions by modulating the cerebral cortex activity. However, the exact cortico-cerebellar interaction mechanisms in subserving higher level functions still remain unclear. As the cerebellum has major afferent and efferent fibers to cerebral cortex areas through the middle (MCP) and superior cerebellar peduncle (SCP), the aim of this study was to assess the specific contribution of cerebellar white matter (WM) damage to cortical grey matter (GM) modifications, and to evaluate the impact of focal cerebellar lesions on cerebral GM. Using diffusion imaging and voxel-based morphometry (VBM), we attempted to clarify the cerebro-cerebellar interaction mechanisms and their anatomo-functional correlates in a cohort of patients with left or right cerebellar lesion as compared to normal controls. MCP and SCP were reconstructed by diffusion tractography, while cortical GM atrophy was assessed by VBM. From the reconstructed tracts, measures of microscopic damage were assessed, while GM volumetrics was investigated voxel-wise. Surprisingly, WM analyses showed that diffusivity changes affected the cerebellar tracts bilaterally in most patients. Accordingly, significant reductions of GM volume were found in the cerebral cortex ipsilaterally as well as contralaterally to the cerebellar lesion. We speculate that impaired transmission via cortico-cerebellar pathways as expressed by bilateral diffusivity changes in WM tracts, accounts for bilateral alterations in the cerebral cortex. This evidence could justify the bilateral cognitive impairments observed in cerebellar patients despite the presence of unilateral lesions.

Keywords: Functional and Structural Neuroimaging/ NIRS

Correspondence address: Mrs Dr Giusy Olivito, Department of Psychology, “Sapienza” University of Rome; Ataxia Laboratory, Santa Lucia Foundation, Rome, Italy, Via Dei Marsi, 78; Via Ardeatina, 306, 00185 Rome, Italy. E-mail: giusyolivito@hotmail.it

### P1.46

### Non-pharmacological treatment in cognitive decline: Cognitive and behavioural outcomes

Valentina Manfredi, Serena Oliveri, Annalisa Parente, Letizia Schifano, Alfredo Raglio and Anna Rita Giovagnoli

*Fondazione IRCCS Istituto Neurologico C. Besta, Department of Diagnostics and Applied Technology, Milan, Italy*

INTRODUCTION: Brain functions can change substantially as a result of practice and experience but there is poor evidence of the influence of non-pharmacological treatment on cognitive decline.
GOALS OF THE STUDY: This study compared the effects of Active Music Therapy (AMT) and Cognitive Training (CT) on cognitive and behavioural aspects in patients with cognitive decline.
METHODS: Patients with early-stage cognitive decline were serially assigned to CT or AMT. Each treatment included 30, 45-minutes group sessions. CT sessions included exercises stimulating attention, information processing, executive functions, and memory. ATM sessions used a non-verbal approach and free sound-music interactions, using rhythmical-melodic instruments.
RESULTS: After CT, the mean test scores in verbal initiative and episodic memory significantly improved, while mood and social relations showed no significant change. After AMT, mood and social relations tended to improve, while no significant changes emerged in cognitive aspects.
CONCLUSIONS: Results reveal that non-pharmacological treatment can contribute to improve cognitive and behavioural aspects in patients with chronic cognitive impairment. Such treatment may contribute to the clinical care of adult and elderly patients.

Keywords: Other Functions/Disorders, Dementia, Neuropsychological Rehabilitation

Correspondence address: PhD Valentina Manfredi, Fondazione IRCCS Istituto Neurologico C. Besta, Department of Diagnostics and Applied Technology, Via Celoria, 11, I-20133 Milan, Italy. E-mail: valentina.manfredi@istituto-besta.it

### P1.47

### Skin autofluorescence as a marker of tissue advanced glycation endproducts, type 2 diabetes, and cognitive performance: Preliminary results of The Maastricht Study

Peggy Spauwen[1], Marcelle Van Eupen[2], Casper Schalkwijk[2], Miranda Schram[3], Coen Stehouwer[3], Frans Verhey[1], Martin Van Boxtel[1]
[1]*Maastricht University Medical Center, Department of Psychiatry and Neuropsychology, School for Mental Health and Neuroscience (MHeNS), Maastricht, Netherlands*
[2]*Maastricht University Medical Center, Laboratory for Metabolism and Vascular Medicine, Department of Internal Medicine, Cardiovascular Research Institute Maastricht, The Netherlands*
[3]*Maastricht University Medical Center, Department of Internal Medicine, Cardiovascular Research Institute Maastricht (CARIM), Maastricht, The Netherlands*

GOALS: Type 2 diabetes (T2D) is associated with an increased risk of cognitive impairment and dementia. However, the underlying mechanisms are not clear yet. One proposed mechanism involves the accumulation of advanced glycation end-products (AGEs). The aim of this study was to examine whether skin autofluorescence (SAF), i.e. a non-invasive marker of tissue AGEs, is associated with lower cognitive performance and whether this association is dependent on diabetes status.
METJODS: 862 participants from The Maastricht Study (aged 40–75 y) were included, of whom by design 253 (29.3%) had T2D. SAF was measured with the AGE reader (DiagnOptics, Groningen) ($N = 831$). Associations between SAF and performance on tests of global cognitive functioning, information processing speed, immediate and delayed word recall, and response inhibition were measured and adjusted for demographics (age, sex, education), diabetes, smoking, alcohol use, and comorbid conditions (hypertension, cardiovascular disease, body mass index, and estimated glomerular filtration rate (eGFR)).
RESULTS: After adjusting for demographics and diabetes, SAF was significantly associated with scores for immediate word recall ($B = -1.39$, $P = 0.03$), delayed word recall ($B = -0.47$, $P = 0.02$) and with (log-transformed) scores for response inhibition ($B = 0.03$, $P = 0.02$). Other cognitive scores were not associated with SAF. After full adjustment, SAF was not significantly associated anymore with immediate word recall, ($B = -1.03$, $P = 0.13$), delayed word recall ($B = -0.38$, $P = 0.07$), and response inhibition ($B = 0.03$, $P = 0.06$). There was no significant interaction between SAF and diabetes on any cognitive score.
CONCLUSION: After adjustment for demographics, diabetes, smoking, alcohol use, and comorbid conditions, SAF, as a marker for tissue AGEs, was not significantly associated with cognitive performance. Our results might indicate that tissue AGEs are not independently associated with the development of cognitive impairment in T2D.

Keywords: Other Functions/Disorders, Dementia, Neuropsychological Assessment and Psychometrics

Correspondence address: Mrs MSc Peggy Spauwen, Maastricht University Medical Center, Department of Psychiatry and Neuropsychology, School for Mental Health and Neuroscience (MHeNS), Dr.Tanslaan 12, 6220 MD Maastricht, Netherlands. Tel.: +31 4338810 49; E-mail: peggy.spauwen@maastrichtuniversity.nl

### P1.48

### Health numeracy in patients with Mild Cognitive Impairment (MCI)

Marie-Theres Pertl[1], Thomas Benke[2], Laura Zamarian[2], Caroline Martini[3], Thomas Bodner[2], Elfriede Karner[2] and Margarete Delazer[2]
[1]*Medical University Ínnsbruck, Clinical Department for Neurology, Innsbruck, Austria*
[2]*Medical University Innsbruck, Clinical Department for Neurology, Innsbruck, Austria*
[3]*Leopold Franzens University Innsbruck, Department for Psychology, Innsbruck, Austria*

Patients with Mild Cognitive Impairment (MCI) are by definition still autonomous in daily life and therefore make their own decisions, for example, concerning their own or their partners' health care. Health care information typically contains complex mathematical constructs like proportions, probabilities and survival rates, which are difficult to understand even for healthy older adults. The purpose of this study was to investigate if patients with MCI have difficulties with understanding health numeracy questions and to explore the impact of declining cognitive functions. The performance of 29 patients with MCI in a health numeracy questionnaire was compared to the performance of a control sample including 164 healthy older adults matched in age and educational level. Participants were asked to convert percentages, assess different probabilities, or understand the dosage of a short patient information leaflet. Additionally neuropsychological background tests were administered (CERAD plus).
Patients with MCI answered less items correctly in the health numeracy questionnaire than controls (Mdn 6.0 vs. 8.0). None of the patients reached a ceiling-effect (in comparison 7,9% of the controls). A correlation analysis showed significant associations between performance in the health numeracy task, years of education, mental arithmetic, executive functions, naming to confrontation and figural recall, respectively. In a regression analysis a model with two predictor variables emerged (psychomotor speed, planning/conceptualization) accounting for 42,2% of the variance.
Patients with MCI show problems in understanding numerical information concerning health care. They even had difficulties in understanding a simple dosage instruction. Since patients with MCI are confronted with several health care decisions, information has to be presented in an easily understandable way (e.g. pictograms).

Keywords: Other Functions/Disorders, Mild Cognitive Impairment, Neuropsychological Assessment and Psychometrics

Correspondence address: Mrs Mag. Marie-Theres Pertl, Medical University Ínnsbruck, Clinical Department for Neurology, Anichstraße 35, A-6020 Innsbruck, Austria. E-mail: marie-theres.pertl@i-med.ac.at

**P1.49**

**Active music therapy influences cognition and behaviour in chronic vascular encephalopathy: A case report**

Serena Oliveri, Valentina Manfredi, Annalisa Parente, Letizia Schifano, Alfredo Raglio and Anna Rita Giovagnoli
*Fondazione IRCCS Istituto Neurologico C. Besta, Department of Diagnostics and Applied Technology, Milan, Italy*

INTRODUCTION: Recent literature supports music therapy as a promising non-pharmacological approach to the behavioural and psychological symptoms in cognitive, social and emotional rehabilitation.
GOALS OF THE STUDY: To provide an operational description of active music therapy (AMT) and its benefits on cognition and behaviour.
METHODS: A patient with cognitive decline associated with Vascular Cognitive Impairment-No Dementia (VCI-ND) underwent a four-months AMT based on creative and interactive music playing. The therapy was conducted simultaneously to the pharmacological therapy. Behavioural, socio-emotional, and cognitive aspects were assessed before and after treatment using a comprehensive neuropsychological and psycho-behavioural battery.
RESULTS: The baseline cognitive pattern was characterized by deficits in object and figure naming, semantic verbal fluency, short and long-term verbal memory, short-term spatial memory, selective attention, and visuo-motor coordination. The patient reported a tendency to feel tense, nervous, and angry and difficulties in verbal memory and visuo-spatial abilities, frequently accompanied by attention drops. The social network was an habitual component of the patient's life, but not a source of sharing personal experiences, safety, or comfort. After completing AMT, the cognitive profile significantly improved in the domains of attention, visuo-motor coordination, and verbal and spatial memory. All of these improvements were confirmed three months after the end of the therapy, demonstrating a stability of the results. A marked increase of interpersonal interactions and reduction of state anxiety were observed at the two follow-ups.
CONCLUSIONS: These results suggest that a structured AMT may have positive effects on cognition and social interactions. In neurological patients with cognitive decline, these effects may facilitate the participation to standard pharmacological and rehabilitation treatments.

Keywords: Other Functions/Disorders, Mild Cognitive Impairment, Other Methods

Correspondence address: Mrs PhD Serena Oliveri, Fondazione IRCCS Istituto Neurologico C. Besta, Department of Diagnostics and Applied Technology, Via Celoria, 11, I-20133 Milan, Italy. E-mail: serena.oliveri@istituto-besta.it

### P1.50

### COGNITIVE screening in parkinson's disease: normation and validation of the panda-f, and comparison with 3 other scales

Anne-Isabelle Gasser[1], Elke Kalbe[2], Pasquale Calabrese[3], Joseph Kessler[4] and Philippe Rossier[1]
[1]*Hôpital fribourgeois, Rehabilitation Unit, Billens, Switzerland*
[2]*University of Vechta, Institute of Gerontology and Center for Neuropsychological Diagnostics and Intervention (CeNDI), Vechta, Germany*
[3]*Neurocenter of Southern Switzerland, Lugano, Switzerland*
[4]*University Clinic Cologne, Department of Neurology, Cologne, Germany*

INTRODUCTION: the Parkinson Neuropsychometric Dementia Assessment (PANDA) is a new screening tool, validated in German, to assess cognition and mood in Parkinson's disease (PD). It includes 5 cognitive tasks and a short mood questionnaire (PANDA-MQ). AIMS: to translate the PANDA into French, normalise it, determine cut-off scores for mild cognitive impairment (MCI), dementia and depression and to compare it with MMSE, Mini Mental Parkinson (MMP) and clock test (CT).
METHODS: the normation was conducted with 61 control subjects, who were compared to the original study's control group. Afterwards, 51 patients with PD were administered the French version of the PANDA (PANDA-F), the MMSE, the MMP and the CT. They were classified as either having normal cognition ($n = 15$), MCI ($n = 20$) or dementia ($n = 16$) on the basis of an extensive neuropsychological battery of tests administered by an examinator blinded to the above mentioned screening test results. The concurrent validity of the PANDA-MQ was determined against the Geriatric Depression Scale (GDS).
RESULTS: our results show that the PANDA-F can be used with the same transformation algorithms as the German version, adding a correction for education. The PANDA-F total score produced excellent discrimination of PD patients with dementia from those without (area under the curve [AUC] = 0.97) and of PD patients with cognitive impairment (MCI and dementia) from those without (AUC = 0.90). With the cut-off scores proposed for the German version, the PANDA-F has a specificity of 94% and a sensitivity of 100% for PD-D and, respectively, 100% and 72% for cognitive impairment. When compared with the three other screening tools, the PANDA-F exhibits the highest AUC for both MCI and dementia. The PANDA-MQ has a strong concurrent validity with the GDS ($p < 0.001$).
CONCLUSION: the PANDA-F is a useful screening tool showing, with the same cut-off scores as the German version, good sensitivity and specificity for dementia and MCI in PD patients.

Keywords: Other Functions/Disorders, Neurodegenerative Diseases, Neuropsychological Assessment and Psychometrics

Correspondence address: Mrs Anne-Isabelle Gasser, Hôpital fribourgeois, Rehabilitation Unit, Rue de l'Hôpital 1, CH-1681 Billens, Switzerland. E-mail: annegasser@bluewin.ch

### P1.51

### Is the Rank Order of WAIS-subtest Scores typical to the Locus of Brain Lesion? An example of the use of diagnostic data base

Harri Koski
*PGI Koski, TAMPERE, Finnland*

The aim of this study was to examine, do the local brain lesions produce a locus typical rank order of WAIS-subtest scores and to report the utility of the diagnostic data base in neuropsychological assessment.
The patients were a) adults, b) right-handed, c) the lesion was limited to the left or right temporal or parietal lobe and d) eight subtest (similarities, arithmetic, vocabulary, digit span, picture completion, picture arrangement, block design and digit symbol) from WAIS or WAIS-R were done. WAIS -subtest rank order of the patients ($N = 57$), including 47 examples from literature, was compared to the results published by McFie (Journal of Nervous and Mental Disease, 1960, 131, 383–393). The comparison group ($N = 162$) was from Finnish WAIS-R standardisation material (age 15–73, no neurological diseases). Spearman rank order correlation was used to test the compatibility between individual test profile and different profiles of lesion groups and a comparison profile. Comparison profile was the averages of standard scores. The highest correlation between test profile and McFie-lesion group was used to describe the compatibility.
The results indicate a statistically very significant connection between patients subtest scores and diagnostic data base (McFie lesion groups). The sensitivity and the specificity were examined. The profiles of patient groups reached to the adequate lesion group profiles and were statistically different from the comparison group. There were differences in selectivity between patient groups. The adequacy to clinical assessment was discussed critically.
The results demonstrate that local brain lesions produce a locus typical rank order of WAIS -subtest scores. Because the sample was quite narrow and the data base would require updating, we should to be careful with further conclusions. However it would be useful to collect up-to-date data bases of specific brain lesion cases.

Keywords: Other Functions/Disorders, Other Disorder/Samples, Neuropsychological Assessment and Psychometrics

Correspondence address: Mr L.A. Psych. Harri Koski, PGI Koski, Kehräsaari B V 9, FI-33200 TAMPERE, Finnland. Tel.: +35 8405012161, Fax: +35 832238989; E-mail: harri.koski@pgikoski.fi

**P1.52**

**Blinking behaviour during cognitively demanding tasks in work-related burnout**

Marianne Leinikka, Laura Sokka, Satu Pakarinen, Jussi Korpela, Andreas Henelius and Minna Huotilainen
*Finnish Institute of Occupational Health, Helsinki, Finland*

Electroencephalogram (EEG) measurement is highly sensitive towards both subject- related and environmental artefacts. Typically these artefacts are unwanted and removed from the data by various methods. Sometimes, however these subject-related artefacts, such as eyeblinks, can provide relevant information on the physiological state of the individual. Mental load, fatigue and anxiety are examples of the mental and emotional factors increasing blinking activity. In this study, we compared the eye blinking behaviour of currently working subjects suffering from different levels of burnout (mild: $N = 24$; severe: $N = 14$) and their healthy controls ($N = 24$). The eye blinking rate (blinks/sec), and number of blinks were calculated from a 4-min. baseline recording in the beginning of a stressful day, and a 30-min. passive mismatch negativity (MMN) paradigm in the end of the same day. The subjects with severe burnout blinked more often already in the beginning of the day as compared to the control and the mild burnout groups, suggesting that the subjects with severe burnout experience heightened stress and fatigue already in the beginning of the workday. Moreover, the blinking rate increased throughout the day in all groups, with the most increase in the group with severe burnout. However, in the beginning of the last task of the day both burnout groups blinked less than controls, possibly due to paying extra effort. But by the end of this task, the blinking rate of the severe burnout group had again exceeded the rate of the controls and the mild burnout group. These results suggest that severe burnout is associated with heightened stress and fatigue already in the beginning of the day and that these detrimental symptoms become stronger with the day passing and the work load increasing. Interestingly, subjects with burnout seem to be able to suppress these symptoms temporarily, which may explain why their condition is not always revealed in brief testing situations.

Keywords: Other Functions/Disorders, Other Disorder/Samples, Electrophysiology (EEG/ERP)

Correspondence address: Mrs Marianne Leinikka, Finnish Institute of Occupational Health, Haartmaninkatu 1 A a, FIN-00290 Helsinki, Finland. E-mail: marianne.leinikka@ttl.fi

**P1.53**

**Baseline cognitive functions among elderly localized breast cancer patients**

Marie Lange[1], Florence Joly[2], Sabine Noal[3], Natacha Heutte[4], Aurélie Daireaux[5], Chantal Rieux[5], Johan Le Fel[6], Olivier Rigal[7], Vincent Roy[6], Bénédicte Clarisse[5], Francis Eustache[8] and Bénédicte Giffard[8]
[1]*INSERM U1077 et U1086, Université de Caen Basse-Normandie, Ecole Pratique des Hautes Etudes, UMR-S1077, CHU de Caen, CAEN, France*
[2]*INSERM U1086, Unité de recherche clinique Centre François Baclesse, CHU de Caen, Service d'oncologie, Caen, France*
[3]*INSERM U1086, Unité de recherche clinique Centre François Baclesse, CHU de Caen, Unité de Recherche Clinique,Centre François Baclesse, Caen, France*
[4]*INSERM U1086, Unité de recherche clinique Centre François Baclesse, UFR de sciences pharmaceutiques,*

*Caen, France*
[5]*Centre François Baclesse, Unité de recherche clinique, Caen, France*
[6]*Université de Rouen, EA4306, Rouen, France*
[7]*Centre Henri-Becquerel, Département d'oncologie médicale, Rouen, France*
[8]*INSERM U1077, Université de Caen Basse-Normandie, Ecole Pratique des Hautes Etudes, UMR-S1077, CHU de Caen, France*

BACKGROUND: Cognitive deficits (CD) are reported among cancer patients receiving chemotherapy (CT), but can also be observed before treatment. Elderly patients are poorly studied although they are more prone to present age-related CD and CD onset or enhancement during CT. This study assessed baseline cognitive functions – i.e. before adjuvant treatment therapy – in elderly localized breast cancer (LBC) patients.
METHODS: Results concern 123 elderly LBC patients (71 $\pm$ 4 years): planned treatment included CT and radiotherapy (RT) for 61 patients and RT only for 62 patients. Episodic memory, working memory, executive functions and information processing speed were assessed by Grober and Buschke procedure, Rey-Osterrieth complex figure test, Working Memory Index – WAIS III, Trail Making test, and verbal fluencies. Validated questionnaires assessed subjective CD, anxiety, depression and fatigue (FACT-Cog, STAI, BDI, FACIT-F). Geriatric assessment was also realized (ADL, IADL, GDS). Objective CD were defined as a score less than 1.5 standard deviation (SD) of normative data on $> 2$ tests, or less than 2 SDs on 1 test.
RESULTS: Before any adjuvant treatment, objective CD were observed in 40% of patients (46% in CT group, episodic memory mainly impaired, and 37% in RT group, executive functions and information processing speed mainly impaired). No relation was observed between cancer stage, geriatric frailty and objective CD. Twenty nine percent of patients presented fatigue, 6% anxiety and 10% depression. These variables were not related to objective CD but they were related to subjective CD.
CONCLUSIONS: More than 40% of elderly LBC patients presented objective CD before any adjuvant therapy that is higher than observed among younger patients (20–30%). It is important to take account in the decision making of adjuvant treatment in elderly patients. More systematic cognitive assessments should be provided in the initial oncological assessment.

Keywords: Other Functions/Disorders, Other Disorder /Samples, Neuropsychological Assessment and Psychometrics

Correspondence address: Mrs Marie LANGE, INSERM U1077 et U1086, Université de Caen Basse-Normandie, Ecole Pratique des Hautes Etudes, UMR-S1077, CHU de Caen, Centre François Baclesse, INSERM 1086, 3 Av. Généréal Harris, FRANCE-14000 CAEN, France. E-mail: m.lange@baclesse.fr

### P1.54

### The neuropsychological assessment of cognitive deficits in consideration of various measures of variability

Celine Tanner-Eggen[1], Christian Balzer[1] and Klemens Gutbrod[2]
[1]*Reha Rheinfelden, Neuropsychology, Rheinfelden, Switzerland*
[2]*University Hospital Inselspital Bern – Neuropsychology, Bern, Switzerland*

In neuropsychological assessment, not only single test scores, but also entire performance profiles should be considered to identify cognitive deficits. Moreover, not only mean levels of performance, but also intraindividual performance variability should be examined when interpreting neuropsychological profiles. There is growing evidence that variability is prevalent also in healthy individuals. Analysing the large normative sample ($n = 569$) of a comprehensive neuropsychological test battery (Materialien und Normwerte für die neuropsychologische Diagnostik; MNND), the purpose of our study is to improve the awareness of normal variability by discussing various types of performance variability. One goal is to exhibit information about the number of low scores in a healthy population. A second goal is to provide base rate information about different levels of dispersion across cognitive abilities. A third goal of the study is to determine the magnitude of laterality patterns (i.e., performance discrepancies between verbal and non-verbal tests or domains). The base rates of low scores were calculated using different cutoffs for abnormality. Dispersion levels were examined by the intraindividual standard deviation (ISD) score. The base rates of different performance discrepancies were analysed forming contrast scores. The results of the analyses showed that intraindividual performance variability is common in the normative sample of MNND. The educational level had a significant effect on the number of low scores and on the performance discrepancies. Therefore, these base rates were stratified by years of education. The base rates are presented in user-friendly tables, allowing clinicians additional interpretive methods to improve accurate identification of cognitive deficits in neuropsychological assessment.

Keywords: Neuropsychological assessment, normal variability, base rates, Other Functions/Disorders, Other Disorder/Samples, Neuropsychological Assessment and Psychometrics

Correspondence address: Mrs lic.phil. Celine Tanner-Eggen, Reha Rheinfelden, Neuropsychology, Salinenstrasse 98, CH-4310 Rheinfelden, Switzerland. Tel.: +41 795125510; E-mail: celineeggen@bluewin.ch

### P1.55

### Longevity and neuropsychology. An experimental study about cognitive empowerment in old adults

Giuseppe Alfredo Iannoccari, Sara Eralti and Adriana Bortolotti
*Assomensana Association, Monza, Italy*

Cognitive stimulation is a neuropsychological intervention strategically oriented toward the general well-being of each one. It allows to reinforce and to maintain the cognitive agility and flexibility by tasks that involve different functions: language, problem solving, memory, attention, etc. The present study explored the effect of annual cognitive training by evaluative test of cognitive efficiency (SPM- Standard Progressive Matrices, TMT A-B, CAiG). Meetings happened in group once a week. During these sessions, subjects were trained with paper-pencil exercises with management of a neuropsychologist. 194 subjects (M = 64, 34; SD = 6,99) participated to the experiment, shared in two groups: one experimental that adhered to the sessions of cognitive stimulation and one of control that, instead, exclusively participated in the evaluative meetings. By comparison of two groups, many meaningful differences occur: repeated measures ANOVA between subjects found a performance increase in attentional and linguistic tasks, of nonverbal intelligence, of short and long-term memory, of working memory and of speed in spatial exploration from the experimental group. In conclusion, we suggest that a cognitive training, based on neuroscience knowledge, can increase the cognitive resources and allow to develop of meta-cognitive abilities and cognitive self-activation skills.

Keywords: Other Functions/Disorders, Other Disorder /Samples, Neuropsychological Assessment and Psychometrics

Correspondence address: Mr Ph.D. Giuseppe Alfredo Iannoccari, Assomensana Association, via Caronni 2, I-20900 Monza, Italy. Tel.: +39 26360914; E-mail: g.iannoccari@assomensana.it

### P1.56

### Predictors for improvement of cognition in patients with idiopathic normal pressure hydrocephalus

Sandra Michaela Lettner and Andreas Kampfl
*Department of Neurology, Ried im Innkreis, Hospital of the Sisters of Charity Ried, Austria*

SPECIFIC GOAL: The main goal was to define changes in specific neuropsychological functions in patients with idiopathic normal pressure hydrocephalus (iNPH) before and after singular lumbar puncture (sLP) and evacuation of cerebrospinal fluid (CSF).
METHOD: All patients with iNPH had a cerebral magnetic resonance tomography (MRT) before sLP. The cognitive testing battery before sLP and at points in time day 1–9 after sLP included: memory, attention, motor activity, reaction time and verbal fluency. All patients had cerebrospinal fluid (CSF) evacuation of 40–50 mls by sLP.
SUMMARY OF RESULTS: 64 inpatients were included in the study. 40,6% were female, the mean age was 86,45 years (SD 12,75 years). The following neuropsychological deficits correlated with the diagnosis of an iNPH: memory, attention, motor activity, reaction time and verbal fluency. Improvement in motor activity, verbal fluency and reaction time was observed in all patients at day 4–9 after CSF evacuation by sLP.
CONCLUSION: Our data suggest that specific cognitive testing is helpful in establishing the diagnosis of an iNPH. In particular, our results indicate that motor activity, verbal fluency and time of reaction improve only at specific time points after sLP in patients with iNPH.

Keywords: Other Functions/Disorders, Other Disorder /Samples, Neuropsychological Assessment and Psychometrics

Correspondence address: Mag. Dr. Sandra Michaela Lettner, Hospital of the Sisters of Charity Ried, Department of Neurology, Schlossberg 1, A-4910 Ried im Innkreis, Austria. Tel.: +43 676 420 8229; Fax: 0043 7752 95044, E-mail: sandra.lettner@bhs.at

**P1.57**

**Cognitive performances of adolescents hospitalized in a For-K Unit**

Gaëlle Grajek[1], Stéphanie Charles[2], Audrey Pauwels[2] and Laurent Servais[2]
[1]*Centre Hospitalier Jean Titeca, Unité Karibu B3, Brussels, Belgium*
[2]*Centre Hospitalier Jean Titeca, Brussels, Belgium*

As patients with schizophrenia often present cognitive disorders (e.g. Medalia and Choi, 2009), it seemed interesting to focus on the neuropsychological functioning of our population, namely juvenile offenders with severe psychotic disorders.
Therefore, we systematically evaluated the cognitive functioning of all patients admitted in the For-K units of the CHJ Titeca (Brussels) since november 2010. All patients ($N = 37$) were male adolescents aged 15 to 18 years, suffering from severe psychiatric disorders and under judicial measure. As a part of the therapeutic support in the units, all adolescents were evaluated with a full cognitive assessment one month after the admission to objectivate and quantify the presence and extent of cognitive deficits and to integrate them in the multidisciplinary therapeutic process. The obtained scores were converted to standardized z scores based on published norms for the tests. One sample t-tests were used to compare stardardized scores with norms. All performances on tests evaluating visual treatment and processing speed (Rey's Complex Figure, Wechsler's subtests Picture Completion, Block Design and Coding) were significantly lower than norms (all $p < 0.001$). Patient's scores on verbal working memory (Digit Span) were also significantly lower than norms ($p < 0.001$). Their performances on a verbal episodic memory task were not different from norms when evaluated with the California Verbal Learning Test (all $p > 0.05$), while they scored significantly lower than norms on the delayed recall and delayed recognition of the Stories CMS (all $p < 0.01$), suggesting a lack of understanding of the stories. Performances on attention, executive functioning and visual episodic memory require a more detailed interpretation.
Our findings highlight the presence of cognitive impairments in adolescents hospitalized in For-K units and confirm the necessity of developping appropriate intervention methods.

Keywords: Other Functions/Disorders, Psychopathology, Neuropsychological Assessment and Psychometrics

Correspondence address: Mrs Gaëlle Grajek, Centre Hospitalier Jean Titeca, Unité Karibu B3, Rue de la Luzerne 11, B-1030 Brussels, Belgium. E-mail: gaelle.grajek@chjt.be

**P1.58**

**Validity of cognitive screening tests in mild acute stroke**

Klaus Hess[1] and Claudia Jansen[2]
[1]*Universitätsklinik Heidelberg, Neurology, Heidelberg, Germany*
[2]*Universitätsklinik Heidelberg, Neurologie, Heidelberg, Germany*

Cognitive screening instruments are widely used in stroke patients. Still the sensitivity and specificy of these instruments in comparison to a complete neuropsychological testing remains unclear.
METHOD: To examine the validity of three Screening Instruments we applied the Montreal Cognitive Assessment (MoCA) and the Addenbrooks Cognitive Examination-Revised (ACE-R) in addition to a standard neuropsychological testing. In a prospective design we included $n = 60$ patients with mild (mean NIHSS: 3,6; range: 0–18) acute stroke who were admitted to the Department of Neurology of the University Hospital Heidelberg. Excluded were patients with neurological deficits that interfered with the testing, and patients with obvious cognitive deficits like aphasia, visual neglect, and patients with depression. The mean age was 60 years (range 32–83). Most frequently patients suffered from a stroke of the MCA ($n = 25$; 41%), followed by stroke of the PCA ($n = 16$; 26,7%). Our standard test battery was comprised of the Hooper Verbal Learning Test (HVLT); Rey Complex Figure, Trail Making Test A + B, Letter-Digit-Test (WAIS),

Block Tapping, and a german word fluency test (RWT Animals). A cognitive deficit was defined by a percentile $< 10$ in selected parameters of each test with the exception of the HVLT (percentile $< 5$). A patient was classified as having a cognitive deficit if he scored lower than this cutoff in at least two test parameters. For the screening tests we used common cutoffs: MMSE $< 27$, MoCA $< 26$ and ACE-R $< 88$.
RESULTS: The ACE-R showed the highest sensitivity (71%), followed by MoCA (58,1%), and the MMSE (35%). On the other hand, specifity ranged from 86,2% (MMSE), 65,5% (MoCA), to 69,0% (ACE-R).
CONCLUSION: No Screening Instrument can substitute a good clinical practice testing in mild acute stroke. In this study the ACE-R showed the highest sensitivity and specifity using the common cutoffs. The MMSE should no longer be applied in patients with mild acute stroke.

Keywords: Other Functions/Disorders, Stroke, Neuropsychological Assessment and Psychometrics

Correspondence address: Dr. Klaus Hess, Universitätsklinik Heidelberg, Neurology, INF 400, D-60123 Heidelberg, Germany. E-mail: klaus.hess@med.uni-heidelberg.de

**P1.59**

**Development of a Cantonese version of the Birmingham Cognitive Screen BCoS for stroke survivors**

Brendan Weekes[1], John Chan[2], Diana Ho[2], Pinky Lam[2], Glyn Humphreys[3], Jane Riddoch[3] and Anthony Kong[4]
[1]*University of Hong Kong, Laboratory for Communication Sciences, Hong Kong, China*
[2]*University of Hong Kong, Laboratory for Communication Science, Hong Kong, China*
[3]*Department of Experimental Psychology, University of Oxford, Oxford, UK*
[4]*Department of Communication Disorders, University of Central Florida, Orlando, US*

The Birmingham Cognitive Screening Test (BCoS) is designed to isolate a wide range of cognitive functions in patients with brain injuries including the assessment of aphasia, apraxia, attention, memory and spatial neglect. The purpose of the present study was to (1) develop a Cantonese version of BCoS by translation with cultural modifications of the English version of BCoS; (2) validate the Cantonese version of the BCoS by comparing the scores on BCoS with scores in the Hong Kong version of the Montreal Cognitive Assessment Test (MoCA), Cantonese version of the MMSE and the Cantonese Aphasia Battery (CAB); and (3) evaluate the ability of BCoS to differentiate between stroke survivors and healthy individuals matched in age, education and gender. Twenty two subjects with aphasia (fourteen male and eight female) were recruited. Subjects had a post-onset time of at least 6 months on first assessment day and were all native speakers of Cantonese. Controls were recruited if they had no history of stroke and matched with each subjects in age, education and gender. Results showed concurrent validity of the BCoS using scores obtained from the sections of orientation, naming, attention, immediate recall, delayed recall, language, visuospatial ability, reading and writing when compared against scores obtained from the corresponding tasks in CAB, C-MMSE and HK-MoCA, according to the Pearson correlation coefficients. Inter-rater and intra-rater reliability of the BCoS was established using videos of four subjects reviewed by 2 native Cantonese speakers. Test-retest reliability of the BCoS was confirmed by inviting 5 patients to complete the BCoS twice within three months (T2) of the first BCoS assessment session with identical instructions. Discriminant validity of the BCoS was indicated by the ability of the BCoS to differentiate between stroke survivors and controls of a similar age using. We conclude that the Cantonese version of the BCoS is a reliable and valid instrument.

Keywords: Other Functions/Disorders, Stroke, Neuropsychological Assessment and Psychometrics

Correspondence address: Mr Professor Brendan Weekes, University of Hong Kong, Laboratory for Communication Sciences, University of Hong Kong, D-1111 Hong Kong, China. E-mail: Weekes@hku.hk

**P1.60**

**Quality of life after stroke: Impact of cognitive deficits**

Clemence Bourlon, Marine Lunven and Christophe Duret
*Clinique Les Trois Soleils, Neurology, Boissise le Roi, France*

Stroke is one of the leading causes of long-term disability and quality of life (QoL) in adults (Bonita,

1992). In recent years, several stroke-specific assessments have been developed that include scales designed to measure, among other health concepts, the concept of communicative functioning (Doyle et al. 2004). However, most stroke outcome measurement tools are focused on neurologic symptom status, physical aspects of functioning (Duncan et al. 2003), social functioning (Weissman et al.1981), psychological distress (Burke et al. 1991), pain (Daut et al. 1983), fatigue (Yellen et al. 1997) and rarely assess other important components of health like cognitive functionning.
We proposed a QoL scale modified from Burden of Stroke Scale (BOSS, Doyle et al. 2004) and developed a more details assessment of cognitive burden of stroke (memory, executive functions, language, perception and attention). We assessed 49 consecutive patients with stroke (1 year after hospital discharge) and 22 controls.
Results showed significant differences between comparison groups (Patients vs. Controls) for total score and particularly for mobility, self-care and social relations. Moreover patients reported more significant difficulties in language and attentional abilities, related to the psychological distress.
These findings support the importance of individual's perceptions of their functioning in all cognitive domains. Quality of life is also dependent on specific cognitive processing.

Keywords: Other Functions/Disorders, Stroke, Neuropsychological Assessment and Psychometrics

Correspondence address: Mrs Dr. Clemence Bourlon, Clinique Les Trois Soleils, Neurology, Rue du Château, F-77310 Boissise le Roi, France. Tel.: +33 1689855558; E-mail: c.bourlon@gmail.com

**P1.61**

**Influence of carotid artery stenosis on cognitive functions and emotional state**

Regula Everts[1], Manuela Wapp[2], Yuliya Burren[2], Frauke Kellner-Weldon[2], Marwan El-Koussy[2], Patrik Michel[3] and Schroth Gerhard[2]
[1]*Children's University Hospital, Division of Rehabilitation, Development and Neuropediatrics, Bern, Switzerland*
[2]*University Hospital Inselspital, Institute for Diagnostic and Interventional Neuroradiology, Bern, Switzerland*
[3]*Department of Neurology, University Hospital, Lausanne, Switzerland*

Patients with carotid artery stenosis (CAS) present reduced blood flow to the brain. It is under debate whether CAS directly relates to cognitive impairment or whether CAS is rather a marker for underlying risk factors, themselves influencing cognition. We determined cognitive performance level and the emotional state of patients with CAS and hypothesized that the underlying symptoms, the hemispheric side of stenosis and the degree of stenosis influence performance level.
Sixty-eight patients (52 male; mean age 68.7, range 51–85), all presenting CAS of $\geqslant$ 70% (right-sided $n = 29$, left-sided $n = 27$, bilateral $n = 12$) were included. Fifty-one patients were asymptomatic (no clinical history of stroke or transient ischemic attack, TIA), 17 patients suffered from stroke or TIA (symptomatic). All patients underwent assessment of language, visual and verbal memory, processing speed, executive functions, motor speed, anxiety and depression.
Patients with CAS showed significantly more often impairments in executive functions (processing speed $p < 0.001$; interference control $p < 0.001$), word production ($p = 0.021$), verbal and visual learning ($p = 0.001$, $p = 0.014$), visual recall ($p = 0.014$) and motor speed ($p < 0.001$) than expected from a normative sample. Anxiety was significantly increased in the patient group ($p = 0.048$). Bilaterality of stenosis influenced processing speed significantly ($p = 0.036$), with worst processing speed in patients with bilateral stenosis. Grade of stenosis and underlying symptoms (symptomatic versus asymptomatic) was not associated with cognition or mood.
Cognitive impairment often occurs in patients with CAS and anxiety is increased. The missing link between cognitive impairment and degree of stenosis and underlying symptoms (symptomatic versus asymptomatic) challenges the view that CAS per se leads to cognitive impairment. It is likely that underlying risk factors additionally contribute to the pattern of cognitive impairment in patients with CAS.

Keywords: Other Functions/Disorders, Stroke, Neuropsychological Assessment and Psychometrics

Correspondence address: Dr. Regula Everts, Children's University Hospital, Division of Rehabilitation, Development and Neuropediatrics, Inselspital, CH-3010 Bern, Switzerland. E-mail: regula.everts@insel.ch

### P1.62

### Talent identification and cognitive functioning

Erik Matser
*Polikliniek Neuropsychologie, Helmond, The Netherlands*

Experts in professional soccer approach player selection mainly in a subjective manner, in which, next to physical characteristics, there is growing emphasis on a player's mental capacity. This mental capacity can be objectively measured by using neuropsychological tests. To provide better understanding in the use of these tests as a selection method, it is studied if successful soccer players perform significantly better on neuropsychological tests than less successful soccer players. Two studies were conducted to test this hypothesis. Data was obtained from players in the Dutch National Team and a prominent European team.

In both studies, an independent-measures between group experimental design was used. In using Mann-Whitney U-tests, the scores on various neuropsychological tests of successful and less successful soccer players are compared. In the first study, data of entry-level players was evaluated 10 years later to assess if the earlier obtained test scores are associated with career success in later life (domains: memory, visual speed of processing, planning, response inhibition, simple response speed). In the second study, the scores of a team's first team players were compared to the scores of reserve team players (domains: working memory, mental speed).

Results of the first study show that successful players performed significantly better than less successful players on various neuropsychological tests in the domains working memory and visual speed of processing. No differences in other domains were found. The results of the second study supported the results of the first study.

The main finding of the present studies is that successful soccer players perform better than lower level soccer players on tests that measure working memory and visual speed of processing. These functions can be objectively measured and are useful tools in the selection of professional soccer players. The results of these studies provide new insight in talent identification.

Keywords: Other Functions/Disorders, Other Disorder /Samples, Neuropsychological Assessment and Psychometrics

Correspondence address: Dr. Erik Matser, Polikliniek Neuropsychologie, Zandstraat 96, NL-5705AZ Helmond, the Netherlands. Tel.: +31 492476003, E-mail: matser@neuro-psychologie.nl

### P1.63

### Cognitive effects of pain and psychiatric symptoms in fibromyalgia

A. Soler[1], A. Campabadal[2], C. Galisteo[3], M. Larrosa[3], M.J. Masdeu[3], L. Vigil[3] and M. Jódar[3]
[1]*Universitat Autònoma de Barcelona, Psicologia Clínica i de la Salut, Barcelona, Spain*
[2]*Universitat Autònoma de Barcelona*
[3]*Corporació Sanitària Parc Taulí, Sabadell*

OBJECTIVE: The present study aims to assess attention, memory and executive function in females with fibromyalgia (FM), and to explore the relationship with affective symptoms and pain. METHODS: The sample includes 25 FM patients, 25 chronic pain patients (CP) and 25 healthy controls (HC). All participants completed neuropsychological tasks of verbal and visual memory (RAVLT, Logical Memory and RCFT), immediate and working memory (Digit Span, Spatial Span, Letter Number and PASAT) and executive functioning (Stroop test and FAS). Effort was measured by the TOMM. Level of anxiety and depression was evaluated using the HAM-A and the BDI. Level of pain was measured by the VAS.

RESULTS: Groups do not differ in mean age or education. None of the participants failed the effort test. Statistical analysis using an ANOVA shows significant group differences in anxiety and depression. There are no significant differences in related pain level between both patient groups. Compared to HC, FM and CP patients show significant poor performance in Digit Span, PASAT and RCFT copy. FM group differ from HC in RAVLT first trial, RCFT short-term recall, immediate and delayed Logical Memory recall, Spatial Span total score, Letter Number and Stroop interference index. Patients with CP perform significantly worse in FAS. After controlling for anxiety and depression using an ANCOVA, all differences in cognitive performance disappear, except in RCFT copy and FAS ($p <$ 0.00).

CONCLUSION: Since FM patients show more difficulties than other CP patients in most of the tasks managed, but they do not perform statically different, pain seems to explain part of cognitive decline in patients with FM. The addition of factors associated with the disorder, such as affective symptoms, explain deficits in attention, verbal immediate and working memory, visual short-term memory and information management difficulties in verbal memory. Short and long-term consolidation of verbal information is not affected.

Funded by the Fundació La Marató de TV3.

Keywords: Other Functions/Disorders, Other Disorder /Samples, Neuropsychological Assessment and Psychometrics

Correspondence address: Mrs A. Soler, Universitat Autònoma de Barcelona, Psicologia Clínica i de la Salut, Edifici B Campus UAB, D-08193 Barcelona, Spain. E-mail: anna.soler@live.com

**P1.64**

**Course of cognitive functioning during stroke rehabilitation**

Sascha Rasquin[1], Caroline Van Heugten[2] and Juul Welten[2]
[1]*Adelante-Rehabilitation Centre, Brain Injury, Hoensbroek, The Netherlands*
[2]*University Maastricht, Neuropsychology and Psychiatry, Maastricht, The Netherlands*

OBJECTIVE: To determine the course of cognitive functioning within the subacute phase (< 4 months) after stroke during rehabilitation.
METHODS: Stroke patients admitted to a rehabilitation centre were submitted to a neuropsychological examination on admission (1 month post stroke) and upon discharge (4 months post stroke). Cognitive domains: attention, executive functioning, memory and visual attention.
RESULTS: Forty-two patients (mean age 57.1 years (sd 7.7)) participated. At admission more than half of the patients showed deficits in attention and memory. Patients improved significantly on these domains; the largest improvement was seen in the domain of visual attention, while executive functioning did not improve significantly. Receiving cognitive rehabilitation was not a predictor for improvement.
DISCUSSION: A differential course of cognitive functioning was found in the subacute phase after stroke. The prognosis of visual attention is the most prominent.

Keywords: Other Functions/Disorders, Stroke, Neuropsychological Rehabilitation

Correspondence address: Mrs phd sascha rasquin, Adelante-Rehabilitation Centre, Brain Injury, Zandbergsweg 111, 6432-cc hoensbroek, The Netherlands. Tel.: +31 455282828; Fax: +31 455282627; E-mail: s.rasquin@adelante-zorggroep.nl

**P1.65**

**Industry studies report larger effects: A meta analysis of donepezil RCTs**

Lewis Killin[1], Tom Russ[2], Sharon Abrahams[1], John Starr[2] and Sergio Della Sala[1]
[1]*University of Edinburgh, Psychology, Edinburgh, United Kingdom*
[2]*University of Edinburgh, Alzheimer Scotland Dementia Research Centre, Edinburgh, United Kingdom*

Donepezil hydrochloride (trade name Aricept) is currently prescribed to patients with mild to moderate Alzheimer's disease (AD). This practice is founded on meta-analyses that demonstrate its efficacy over placebo. However, these analyses do not consider the relationship between trial funding and reported effects as a possible source of bias. We investigated whether there is a difference in the effect size of donepezil in AD between industry-funded and independent trials by using a random-effects meta-analysis of standardised cognitive outcomes (MMSE, ADAS-cog). We updated the 2011 UK National Institute of Health and Clinical Excellence technical appraisal of donepezil through a PubMed search. Inclusion criteria were double-blind, placebo-controlled trials of any length comparing patients diagnosed with probable AD (according to NINCDS-ADRDA/DSM-III/IV criteria) taking any dosage of donepezil. With these selected studies ($N = 14$; 10 industry) donepezil was observed as being significantly more effective than placebo. Critically, industry trials reported a larger effect size on standardised cognitive tests than independent trials (Standardized mean difference; 95% CI; p: industry 0.46; 0.37–0.55; < 0.001 vs. independent 0.33; 0.18–0.48; < 0.001). A sensitivity analysis restricted to trials up to 12 weeks duration did not alter the pattern of our results (industry 0.44; 0.34–0.53; < 0.001 vs. independent 0.35; 0.18–0.52; < 0.001), suggesting that the discrepancy does not exist because of different trial durations between industry and independent studies. Our study therefore demonstrates that the results of donepezil studies appear to be biased by funding source. This complies with a robust phenomenon in pharmaceutical research that can and should be identified when treatment effects are reported.

Keywords: Other Functions/Disorders, Dementia, Other Methods

Correspondence address: Mr Lewis Killin, University of Edinburgh, Psychology, George Square, UK-EH8 9JZ Edinburgh. United Kingdom. Tel.: +44 079687225 41; E-mail: lewiskillin@gmail.com

**P2.01**

**Validation of the computerized Cognitive Basic Assessment test battery (COGBAT): Differences in neuropsychological test profiles of patients with schizophrenia and depression**

Johanna Egle[1], Steffen Aschenbrenner[2], Marcel Berthold[1], Rudolf Debelak[1], Franziska Pisch[1], Katlehn Rodewald[3], Daniela Roesch-Ely[3], Marco Vetter[1] and Matthias Weisbrod[3]
[1]*Schuhfried GmbH, Psychology, Moedling, Austria*
[2]*SRH Klinikum Karlsbad Langensteinbach, Psychiatry, Karlsbad, Germany*
[3]*Heidelberg University Hospital, Psychiatry, Heidelberg, Germany*

OBJECTIVES: Cognitive dysfunction is common in various psychiatric diseases and is linked to impaired social, functional and rehabilitation outcomes. The thorough assessment of a patient's neuropsychological profile is an important first step for planning an intervention. The Cognitive Basic Assessment test battery (COGBAT) is a new computerized neurocognitive test battery that was developed for routine clinical assessment. It provides a comprehensive cognitive profile in a short time. The goal of the present study was to evaluate the validity of COGBAT by comparing the cognitive profiles of patients with schizophrenia and depression with those of healthy controls.
PARTICIPANTS AND METHODS: The COGBAT was administered to 65 patients with schizophrenia and 65 healthy controls as well as to 32 patients suffering from depression and their 32 healthy counterparts. Each control group was matched for age, gender and educational level. To compare the three groups with each other, three matched subsamples ($N = 24$) were subsequently drawn from the two patient groups and the healthy controls.
RESULTS: Selective differences in cognitive function between controls and both clinical subsamples were found. Patients with depression performed significantly less well than the control sample on figural memory, response inhibition and working memory. Patients with schizophrenia performed significantly less well on measures of the aforementioned dimensions as well as on planning, cognitive flexibility and processing speed. Schizophrenic patients were significantly more severely impaired than patients with depression on attention, memory and executive functions.
CONCLUSION: This study indicates two distinct cognitive profiles for patients suffering from depression and schizophrenia. The results suggest that COGBAT is a promising tool for assessing cognition deficits in psychiatric patients and highlight the importance of the assessment of a cognitive profile in clinical practice.

Keywords: Attention, Depression, Neuropsychological Assessment and Psychometrics

Correspondence address: Mrs Mag. Johanna Egle, Schuhfried GmbH, Psychology, Hyrtlstraße 45, A-2340 Moedling, Austria. Tel.: +43 22364231548; E-mail: egle@schuhfried.at

**P2.02**

**Cognitive impairment in a cohort of patients with multiple sclerosis undergoing inpatient rehabilitation**

Susanne Glatzl[1], Regina Anker[2], Judith Bidner[2], Christine Hechenberger[2], Melanie Klingler[2], Christian Brenneis[2] and Rainer Ehling[2]
[1]*Reha-Zentrum Münster, Austria*
[2]*Reha-Zentrum Münster, 6232 Münster, Austria*

BACKGROUND: Multiple sclerosis (MS) is a complex and heterogeneous disease associated with long-term disability. Depending on age, disease subtype, disease duration and testing battery cognitive dysfunction is found in 45 to 65 percent of patients with MS (pwMS) (Rao et. al. 1997). Processing speed is most often affected, other particularly vulnerable functions include memory, complex attention and executive functions, whereas generalized dementia is rare in pwMS.

OBJECTIVE: In a first step to acquire data on cognitive impairment in pwMS undergoing inpatient rehabilitation; in a second step to establish a tailored cognitive test battery.
METHODS: Extensive neuropsychological testing included memory tests (verbal learning memory test and visual memory), tests for executive performance (working memory, word fluency, verbal flexibility) and attention test (alertness, divided attention) as well as questionnaire for depression.
RESULTS: 65 pwMS (64.6% female) with various disease subtypes (clinically isolated syndrome 1.5%, relapsing remitting (RRMS) 40.0%, secondary progressive (SPMS) 36.9% and primary progressive (PPMS) 21.5%) had a mean disease duration of 13.5 years (STD 8.6%) and a mean EDSS of 4.7 (STD 2.4). 84% of the pwMS exhibited any cognitive impairment. 44% of patients showed deficits in verbal long-term memory (36% RRMS, 58% SPMS, 35% PPMS). Moreover we found impairments in divided attention 32% (24% RRMS, 45% SPMS, 21% PPMS), alertness 28% (28% RRMS, 25% SPMS, 36% PPMS), working memory 41% (44% RRMS, 37% SPMS, 41% PPMS) and word fluency 30% (44% RRMS, 25% SPMS, 28% PPMS).
CONCLUSION: Using extensive neuropsychological testing, about 84% of pwMS undergoing inpatient rehabilitation is suffering from any type of cognitive impairment. In accordance with previous studies deficits in verbal long-term memory and executive functions are most common. Percentages of different deficits do not vary between different disease courses. Based on these data a tailored cognitive test battery for pwMS undergoing inpatient rehabilitation will be established.

Keywords: Attention, MS/Demyelination, Neuropsychological Rehabilitation

Correspondence address: Mrs Mag. Dr. Susanne Glatzl, Reha-Zentrum Münster, Gröben 700, A-6232 Münster, Austria. Tel.: +43 5337200045319; E-mail: susanne.glatzl@reha-muenster.at

### P2.03

### Top-down attending to and bottom-up detection of multiple simultaneously presented targets are governed by the right IPS

Bianca De Haan and Hans-Otto Karnath
*Zentrum für Neurologie, Sektion Neuropsychologie, Tübingen, Germany*

The ability to respond to multiple simultaneously presented targets is an essential and distinct human skill, as is dramatically demonstrated in stroke patients suffering from visual extinction. The neural correlates underlying this ability are the topic of continuing debate, with some studies pointing towards the TPJ whereas other studies suggest a role for the IPS. We performed an fMRI study to test the hypothesis that whereas the IPS is associated both with the top-down direction of attention to multiple target locations and the bottom-up detection of multiple targets, the TPJ is predominantly associated with the bottom-up detection of multiple targets. We used a cued target detection task with a high proportion of catch trials to separately estimate top-down cue-related and bottom-up target-related neural activity. Both cues and targets could be presented unilaterally or bilaterally. We performed conjunction analyses to determine the neural anatomy specifically associated with bilateral situations. Whereas we found no evidence of target-related neural activation specific to bilateral situations in the TPJ, we found both cue-related and target-related neural activation specific to bilateral situations in the right IPS, suggesting that both top-down attending to and bottom-up detection of multiple simultaneously presented targets are governed by the right IPS.

Keywords: Attention, Neglect, Functional and Structural Neuroimaging/NIRS

Correspondence address: Mrs Bianca de Haan, Zentrum für Neurologie, Sektion Neuropsychologie, Hoppe-Seyler-Str 3, D-72076 Tübingen, Germany. Tel.: +49 070712985661; E-mail: bianca.de-haan@klinikum.uni-tuebingen.de

### P2.04

### Dissociation in optokinetic stimulation sensitivity between omission and substitution reading errors in neglect dyslexia

Lisa S. Arduino[1], Andrea Albonico[2], Manuela Malaspina[2], Marialuisa Martelli[3], Silvia Primativo[3] and Roberta Daini[2]
[1]*University LUMSA and ISTC-CNR, Human Sciences, Rome, Italy*
[2]*University Milano-Bicocca, Department of Psychology, Milan, Italy*
[3]*University of Rome "La Sapienza" and IRCCS Fondazione Santa Lucia, Department of Psychology, Rome, Italy*

SPECIFIC GOAL: Although omission and substitution errors in neglect dyslexia patients (ND) have been always considered as different manifestations of the same acquired reading disorder, recently, a new dual mechanism model has been proposed (Martelli et al., 2011). While omissions are related to the exploratory disorder that characterizes unilateral spatial neglect (USN, Primativo et al., under revison), substitutions are due to a perceptual integration mechanism known as crowding (Martelli et al., 2011). A consequence of this hypothesis is that specific training for omission-type ND would aim at restoring the oculo-motor scanning and should not improve reading in substitution-type ND.
METHOD: According to this aim we administered an optokinetic stimulation to two brain-damaged patients with both unilateral spatial neglect and neglect dyslexia, MA and EP, who showed ND deficits mainly characterized by omissions and substitutions, respectively. MA also showed an impairment in oculo-motor behaviour in a non-reading task, while EP did not.
RESULTS: The two patients presented a dissociation with respect to their sensitivity to optokinetic stimulation (OKS), so that, as expected, MA was positively affected while EP was not.
CONCLUSION: Our results confirm a dissociation between the two mechanisms underlying omission and substitution reading errors in ND patients. Moreover, they suggest that such a dissociation may extend to the effectiveness of rehabilitative procedures.

Keywords: Attention, Neglect, Neuropsychological Rehabilitation

Correspondence address: Mrs Associate Professor Lisa S. Arduino, University LUMSA and ISTC-CNR, Human Sciences, P.zza delle Vaschette 110, RM-00195 Rome, Italy. E-mail: lisa.arduino@uniurb.it

**P2.05**

**How to differentiate between neglect and hemianopia. Four experiments on healthy subjects**

Matteo Sozzi[1], Silvia Pagani[2] and Michela Balconi[2]
[1]*Casa di Cura del Policlinico, Dept. of Neuro Rehabilitative Sciences, Milan, Italy*
[2]*Catholic University of Milan, Research Unit in Neuropsychology of Language, Milan, Italy*

One of the most frequent difficulty in neuropsychological assessment is the differentiation between neglect and hemianopia. Since there is a wide agreement in implicit information processing for patients with neglect, we hypothesized that a priming word in the neglected field should determine a semantic activation effect even when it is not consciously perceived by the patient. On the contrary if the priming word occurs in a blind hemifield this should not determine any facilitation effect. As first step we tested a "locally-dependent" priming effect on healthy subjects in a semantic categorization task.
We conducted four experiments in which we modulated the presence of a string of "X" contralaterally to prime position, prime duration (150 vs 300 ms) and distance from monitor (50 cm vs 100 cm). Prime occurred in six different positions while target appeared in the center of the screen. Three different prime-target conditions were present: related, unrelated and neutral. Twenty healthy subjects took part to the first experiment (prime at 150 ms, 50 cm from monitor); 17 to the second (prime at 150 ms without contralateral string of X, 50 cm distance); 15 to the third (prime at 300 ms, 50 cm from monitor) and 17 to the fourth (prime at 300 ms, 100 cm from monitor). Results: we obtained a significant main effect of semantic relation ($p < 0.001$) in all experiments but not in the first one. We then found a significant main effect of position ($p < 0.05$); in the fourth experiment (prime at 300 ms) this was significant for all the positions of the prime ($p = 0.001$). Moreover we observed that priming duration significantly affects subjects' performance with slower RTs. No significances arose between the two distances from monitor. These first results confirmed significant semantic activation effect for different prime positions, specifically a higher prime duration seems to provide a more sensitive task to be used as differential between neglect and hemianopia.

Keywords: Attention, Neglect, Neuropsychological Assessment and Psychometrics

Correspondence address: Dr. Matteo Sozzi, Casa di Cura del Policlinico, Dept. of Neuro Rehabilitative Sciences, via Dezza, 48, I-20144 Milan, Italy. E-mail: matteosozzi@gmail.com

**P2.06**

**What is the impact of visuo-spatial neglect on motor recovery of the upper paretic limb after stroke?**

Tanja C.W. Nijboer[1], Boudewijn J. Kollen[2] and Gert Kwakkel[3]

[1]*Utrecht University, Rehabilitation Center de Hoogstraat, Experimental Psychology, Helmholtz Institute, Utrecht, The Netherlands*
[2]*University of Groningen, University Medical Center Groningen, Department of General Practice, Groningen, The Netherlands*
[3]*VU University Medical Center, Rehabilitation Centre READE, Department of Rehabilitation Medicine, Department of Neurorehabilitation, Amsterdam, The Netherlands*

The aim of the current study was to investigate the longitudinal relationship between improvements of synergism and strength of the upper paretic limb (i.e. measured with the Fugl-Meyer-arm (FM-arm) and Motricity Index arm (MI-arm), respectively) and visuo-spatial neglect during the first 52 weeks post-stroke. The longitudinal association between neglect and motor impairment were measured in an intensive repeated measurement design including 18 measurement sessions for each subject. Neglect was assessed using the letter cancelation test applied in a prospective cohort of 101 ischemic, first-ever, hemispheric stroke patients. Of these 101 patients, 51 patients showed neglect. All time-dependent measures were taken weekly, starting within 14 days post stroke. From week 10 to 20 biweekly measurements are obtained. The longitudinal relationship of (bi)weekly time on improvement of motor functions and recovery of neglect was investigated using random coefficient analysis and trend analyses. Results suggest not only that neglect patients show more severe impairment of motor function (i.e. synergism and strength) at stroke onset, but also that time-dependent recovery of motor impairment follows a different pattern with less improvement for neglect patients. Conspicuously, the seemingly inhibitory effect of neglect on motor recovery appears to take place mainly within the time window of spontaneous neurological recovery of first 10 weeks post-stroke. This study is the first to disentangle the unique impact of perceptual-attention networks on the longitudinal courses of motor functions in the first months post stroke.

Keywords: Attention, Neglect, Neuropsychological Assessment and Psychometrics

Correspondence address: Mrs Tanja C.W. Nijboer, Utrecht University, Rehabilitation Center de Hoogstraat, Experimental Psychology, Helmholtz Institute, Heidelberglaan 2, 3584CS Utrecht, the Netherlands. E-mail: t.c.w.nijboer@uu.nl

**P2.07**

### The neural basis of reflexive and volitional saccades: evidence from lesion-symptom mapping with stroke patients

Stephanie Rossit[1], Linn Olsen[2], Stephen Butler[3], Keith Muir[4] and Monika Harvey[2]
[1]*University of East Anglia, School of Psychology, Norwich, UK*
[2]*University of Glasgow, School of Psychology, Glasgow, UK*
[3]*University of Strathclyde, Department of Psychology, Glasgow, UK*
[4]*Southern General Hospital, Institute of Neurological Sciences, Glasgow, UK*

The anti-saccade task is an important task for investigating the flexible control that we have over behaviour. In this task, participants must suppress the reflexive urge to look at a visual target that appears suddenly in the peripheral visual field and must instead look away from the target. This task has been widely demonstrated to be extremely sensitive to brain injury. Previous electrophysiology and human neuroimaging studies suggest that the frontal eye fields (FEF) might be more involved in anti-saccades than pro-saccades. In the current study we investigated pro- and anti-saccade performance in right-hemisphere stroke patients with and without left neglect as well as healthy controls. In addition, lesion-symptom mapping was used to investigate the neural basis of the deficits we observed. As expected, we found that neglect patients took significantly longer to initiate a pro-saccade to a left target when compared to the healthy and non-neglect groups. However, surprisingly, we found a non-lateralized impairment specific to neglect in the anti-saccade task: in response to both left and right targets neglect patients presented a high number of erroneous pro-saccades when compared to healthy controls and patients without neglect. Lesion-symptom analysis revealed that whilst the pro-saccade deficit was associated with posterior lesions, involving the intraparietal sulcus going into the lateral occipital and angular gyri, the anti-saccade deficit was associated with more anterior lesions. In particular, poor anti-saccade performance was related to lesions in and around the inferior frontal sulcus, encompassing white matter in the vicinity of the FEF, the insula and supramarginal and superior temporal gyri. These results indicate that neglect is specifically accompanied by inhibitory/volitional deficits that are not spatially lateralized. Moreover, it also expands our understanding of the brain regions critically involved in the control of reflexive and volitional saccades.

Keywords: Attention, Neglect, Neuropsychological Assessment and Psychometrics

Correspondence address: Mrs Stephanie Rossit, University of East Anglia, School of Psychology, EDU Building, Norwich Research Park, NR4-7TG Norwich, UK. E-mail: rossit.sb@gmail.com

**P2.08**

**Different mechanisms underlie bisection tasks: A prismatic adaptation study**

Mariagrazia Ranzini[1], Remy Schmitz[2], Sophie Antoine[1], Sarah Buck[3] and Wim Gevers[1]
[1]*Université Libre de Bruxelles (ULB,FNRS,CRCN), Bruxelles, Belgium*
[2]*University of Hong Kong, HONG KONG, China*
[3]*Université Libre de Bruxelles (ULB), Bruxelles*

Three main tasks are commonly used to evidence a consistent leftward visuospatial bias within the healthy population. On average, line midpoints are shifted slightly toward the left in the line bisection task (LB), pre-bisected lines are processed as longer on their left side in the landmark task (LDM), and the left side of equiluminescent mirror pairs of stimuli are perceived as darker on the left in the greyscales task (GS). Indivudual differences do exist, with some participants showing initial rightward biases. It is also not clear whether the different bisection tasks are equally sensitive to prismatic adaptation (PA), a procedure used to shift artificially the visual field and affecting the visuospatial bias both in healthy participants and in neglect patients. Here, we investigated the relation between the different bisection tasks by taking into account the direction of the initial attentional bias, and by looking at the influence of leftward and rightward prismatic deviation. Spatial biases were highly consistent across different measurement moments within a task. However, no systematic relation between the different bisection tasks was observed. Also the initial bias had an important role. Participants showing an initial leftward bias were more sensitive to prismatic adaptation than participants showing an initial rightward bias, as revealed by LB and LDM task. Our results demonstrate that partially different (attentional) mechanisms underlie the different bisection tasks, and underline the importance of taking the direction of the initial bias into account when investigating the sensitivity of the attentional bias to prismatic adaptation.

Keywords: Attention, Neglect, Neuropsychological Assessment and Psychometrics

Correspondence address: PhD Mariagrazia Ranzini, Université Libre de Bruxelles (ULB,FNRS,CRCN), Avenue Franklin Roosevelt, 50, 1050 Bruxelles, (Belgium). E-mail: mari.ranzini@gmail.com

**P2.09**

**Neuropsychological functioning in children with migraine**

Dragana Vucinic[1] and Gordana Mijalkovic-Stojiljkovic[2]
[1]*Clinic of Neurology and Psichiatry for Children and Youth, Child neurology, Belgrade, Serbia*
[2]*Clinic of Neurology and Psychiatry for Children and Youth, Child Neurology, Belgrade, Serbia*

Migraine is associated with alterations in brain structure that are detected via conventional and experimental imaging techniques. The clinical significance of these abnormalities is not yet clear. The frequency and chronicity of the disorder have drown attention not anly to the therapeutic issues, but also to prognosis, particularly to the possible cognitive consequences. Several neuropsychological studies reported cognitive changes in children and adolescents with migraine, but the findings are controversial.
AIM: The aim of the study was to investigate the possible cognitive disfunction in children with migraine, to determine the type and intensity of cognitive dysfunction, and to compare the results with controls.
METHODS: We investigate 35 children with migraine, aged 10-18 years, diagnosed according to the International Headache Society criteria. 24 children have migraine without aura, and 11 children migraine with aura. The patients were compared with a control group of 32 children without headache, mached for age, sex and intelligence level based on Wechsler Intelligence Scale for Children (WISC). The battery of neuropsichological tests was designed to investigate different cognitive functions.
RESULTS: The performance of our patients was not homogenous. One third of patients scored within normal limits on all tests. The other migraine patients have lower cognitive performance on tasks evaluating sustained attention, verbal memory and information processing speed. No strong association of clinical parameters (including frequency of migraine attacks and disease duration) with results was concluded.
CONCLUSION: Migraine in children is associated with mild impairment in several cognitive domains. Migraine with aura patients display more prominent cognitive changes. The presence of an association between cognitive problems and migraine headacke may impact academic learning and daily activities on the long term.

Keywords: Attention, Other Disorder/Samples, Neuropsychological Assessment and Psychometrics

Correspondence address: MD Dragana Vucinic, Clinic of Neurology and Psichiatry for Children and Youth, Child neurology, Dr Subotica 6-a Str, SRB-11000 Belgrade, Serbia. E-mail: dragana.vucinic.npk@gmail.com

### P2.10

### The left posterior parietal cortex mediates the effects of prismatic adaptation on the representation of time durations

Barbara Magnani[1], Francesca Frassinetti[2], Giuseppa Renata Mangano[3] and Massimiliano Oliveri[4]
[1]*IRCCS Fondazione Santa Lucia, Department of Clinical and Behavioral Neurology, Rome, Italy*
[2]*University of Bologna, IRCCS Fondazione Maugeri, Castel Goffredo, Department of Psychology; Clinica del Lavoro e della Riabilitazione, Bologna; Castel Goffredo, MN, Italy*
[3]*University of Palermo, Department of Psychology, Palermo, Italy*
[4]*University of Palermo; IRCCS Fondazione Santa Lucia, Department of Psychology; Department of Clinical and Behavioral Neurology, Palermo; Rome, Italy*

In our previous studies we provided evidence for an ascending left-to-right spatial representation of time durations by means of Prismatic Adaptation (PA). PA procedure requires to adapt to the visual shift induced by prismatic lenses with repeated pointing movements. After the adaptation participants show a pointing error to the opposite direction of the visual shift (aftereffect) associated with a shift of spatial attention that can affect spatial representations. Indeed we found that a leftward aftereffect distorted time toward an underestimation, while a rightward aftereffect distorted time toward an overestimation.
The posterior parietal cortex (PPC) has been associated either with different processes of PA procedure or with the formulation of the spatial representation of time. Thus, the aim of the present study was to investigate whether the PPC would have a role in mediating the effects of PA on the spatial representation of time.
Twenty-four right-handed adults performed a time task (reproducing a duration pressing a key with the right index finger), before and after PA inducing a leftward or a rightward aftereffect, adapting with the right upper limb. Moreover subjects were stimulated with inhibitory rTMS on the left or right PPC before PA.
We found an abolition of the effects of PA on time when rTMS was delivered on the left and not on the right PPC. To exclude that this lateral effect of the left PPC was due to the fact that subjects responded and adapted with their right limb, we conducted a control experiment where subjects responded and adapted with their left limb. Again the inhibition of the left PPC only, abolished the effects of PA on time.
This results suggest a direct involvement of the left PPC in mediating the effects of PA on the spatial representation of time that does not depend on motor processes.

Keywords: Attention, Other Disorder/Samples, TMS/TCDS

Correspondence address: Mrs DR Barbara Magnani, IRCCS Fondazione Santa Lucia, Department of Clinical and Behavioral Neurology, Via Ardeatina, 306, D-00143 Rome, Italy. E-mail: barbara.magnani2@unibo.it

### P2.11

### Effects of instructions on hemispheric differences in preparatory attention

Laura Gabriela Fernandez[1], Caroline Bichon[1] and Eric Siéroff[2]
[1]*EA 4468, Neuropsychologie du vieillissement, Paris Descartes University, Boulogne-Billancourt, France*
[2]*EA 4468, Neuropsychologie du vieillissement, Paris Descartes University, Institut Universitaire de France, Boulogne-Billancourt, France*

Preparatory attention (PA) to an impending target-stimulus improves its subsequent processing, preventing the interference from distracting events. The intensity of PA to a target can be reinforced by a simple and appropriate instruction. We investigated the capacity of the left and right cerebral hemispheres to reinforce PA, using a lateralized version of the Attentional Preparation Test (APT), developed by LaBerge, Auclair, and Siéroff (2000). We hypothesized that the left hemisphere plays a crucial role in this instructional reinforcement by enhancing the distinction between target and distractors and the attentional activity directed to the selected target location. Sixty-six neurologically healthy subjects participated in this study. A target (black dot), appearing in the right (RVF) or left visual field (LVF), had to be detected. A distractor might appear in the RVF or LVF, thus directly projecting to the contralateral hemisphere (Experiment 1), or at fixation, thus projecting to both hemispheres (Experiment 2). The proportion of trials containing a distractor varied in three blocks (0%, 33%, 67%), in each experiment. The intensity of PA directed to the target was manipulated by instruction: weak (simple task description), or strong (reinforcing attention). Responses times (RTs) were faster under the strong than the weak instruction. Also, RTs in the strong instruction were faster in the RVF than in the LVF when the distractor was lateralized, and were similar in both visual fields when the distractor was located at fixation. The reinforcement of PA by instruction is more efficient in the RVF than the LVF, specifically when both targets and distractors are lateralized. A possibility is that the LH may be crucial to enhance PA to a selected spatial location, and to reduce the interference from distractors. The cooperation between hemispheres might ameliorate this process in the RH when the distractor is located at fixation, thus projecting to both hemispheres.

Keywords: Attention, Other Disorder/Samples, Other Methods

Correspondence address: Mrs Laura Gabriela Fernandez, EA 4468, Neuropsychologie du vieillissement, Paris Descartes University, 71 avenue Edouard Vaillant, F-92774 Boulogne-Billancourt, France. E-mail: lauragfernandez@gmail.com

### P2.12

### Modulation of attention-related EEG parameters by means of attention training?

Tilmann Klein[1], Hellmuth Obrig[2], Angelika Thöne-Otto[3], Arno Villringer[2] and Stefan Frisch[4]
[1]*Max-Planck-Institute for Human Cognitive and Brain Sciences, Neurology, Leipzig, Germany*
[2]*Day Care Clinic for Cognitive Neurology/Max-Planck -Institute for Human Cognitive and Brain Sciences, Leipzig*
[3]*Day Care Clinic for Cognitive Neurology, Leipzig*
[4]*Clinic of the J.W. Goethe-University, Frankfurt*

Attention plays a critical role in many different cognitive domains. In case of brain damage very often deficits in attention can be observed thereby imposing a need for training of these functions also to allow training in neighboring cognitive domains. The question however, whether or not attention training leads to changes in attention-related electrophysiological processes, remains mostly unaddressed.

We therefore included N = 27 participants in our training study (N = 14 training, N = 13 control). Subjects underwent two sessions of EEG (3-stimulus visual oddball paradigm and Eriksen flanker task) and cognitive testing (attention, memory and executive functions). In between subjects of the training group performed 10 sessions of an adaptive computer based training for selective attention.

While subjects improved their performance in the training task in terms of a reduction of reactions times (in combination with an increase in false positive responses) no effect of training on the cognitive abilities of the participants was observed. Amplitudes of the P300 were also not sensitive to training. However minor differences in peak latency related to Target stimuli at electrode Pz were observed between training and non-training group. Correlation-analysis revealed differential relationships between P300 amplitude and latency and measures of cognitive function and training performance.

It might be speculated that duration and intensity of the attention training might have been not sufficient to promote changes in attention-related electrophysiological processes. Also inclusion of healthy participants might have limited the amount of possible improvements. An alternative explanation might be that the task used for deriving P300 amplitudes was too easy so no benefit from training could be inferred. Replication of the study using more complex tasks and a sample of patients suffering from attention deficits is planned.

Keywords: Attention, Other Disorder/Samples, Electrophysiology (EEG/ERP)

Correspondence address: Dr. Tilmann Klein, Max-Planck-Institute for Human Cognitive and Brain Sciences, Neurology, Stephanstrasse 1a, D-04103 Leipzig, Germany. E-mail: tklein@cbs.mpg.de

**P2.13**

**Sustained attention and real world daily living skills in schizophrenic patients**

Sergio Bodas[1], Arantzazu Herran-Boix[1], Mireia Gonzalez-Rodriguez[1], Mar Ariza[2] and Maite Garolera[1]
[1]*Consorci Sanitari de Terrassa, Spain*
[2]*Unitat d'Investigació biomèdica Universitat Autònoma de Barcelona-CST (UIB), Terrassa, Spain*

Schizophrenia is a highly disabling disorder that hampers the social functioning of the affected individuals. It is well known that cognitive impairment, particularly in the attention domain, is related to this poor outcome, but more research is needed to understand the nature and implications of this association. Therefore, the aim of this study was to explore the relationship between sustained attention and real world daily living skills in these patients. This was a cross-sectional study, carried out with clinically stable patients meeting DSM-IV criteria of schizophrenia. 67 schizophrenic patients (73% males), treated with neuroleptics and stabilized, mostly single (79%) and unemployed (85%), with a mean age of 33 (SD 8 years), were enrolled. Sustained attention was assessed using the Continuous Performance Test (CPT), while daily living functioning was explored with the Living Skills Profile (LSP). Additional demographic and clinical information (Positive and Negative Syndrome Scale, PANSS) was collected to discriminate eventual confusional effects. After processing all data, strong correlations were found between LSP total score and CPT omission errors $r = -0.491$ ($p = 0.0001$), CPT commission errors $r = -0.382$ ($p = 0.001$), CPT perseverations $r = -0.282$ ($p = 0.021$) and CPT detectability $r = 0.336$ ($p = 0.006$). All these results (except for the CPT commission errors) remained significant even after being controlled by the intellectual level (vocabulary, WAIS-III) and clinical symptoms (PANSS positive scale, PANSS negative scale and PANSS general psychopathology). Many studies have demonstrated that schizophrenic patients show deficits in their day living functioning. Our research found significant correlations between this functioning and sustained attention performance. So, we suggest that attention deficits could explain part of this impairment and would be a good measure to predict poor outcome.

Keywords: Attention, Psychopathology, Neuropsychological Assessment and Psychometrics

Correspondence address: Mr Sergio Bodas, Consorci Sanitari de Terrassa, Doctor Pearson, 85 2nd 1, Spain-17527 Terrassa, Spain. Tel.: +34 669931045; E-mail: sergiosbg@hotmail.com

**P2.14**

**RevArte Visual Search Task: A sensitive computerized visual search test using touch-screen technology**

Christophe Lafosse[1], Ilke De Smet[1], Ben Vaessen[2] and Lut Dereymaeker[3]
[1]*Rehabilitation Hospital RevArte and KU Leuven – Neuropsychology, Edegem, Belgium*
[2]*Diagnoseis – Assessment and technology, Maastricht, The Netherlands*
[3]*Rehabilitation Hospital RevArte – Medicine, Edegem, Belgium*

INTRODUCTION: Computer-based tasks provide more sensitive and accurate measurements of different performance indexes or parameters. We developed a computerized visual search (RVST) task by which the patient has to search the presence of some predefined target patterns in a visual display consisting of many distractor-stimuli. As target detectability depends on the spatial properties of the target and the positional uncertainty and the number of distractor stimuli, we hypothesize that the RVST is sensitive for hemispatial differences as a result of lateralized attentional disfunctions in the contralesional hemifield.
METHOD: 20 patients with either a left or right hemispheric stroke, with and without evidence for unilateral neglect, participated in the study. All visuomotor responses were registrated in peri-personal space using a 22 inch digital tablet (DIN A3) (www.diagnoseis.nl). For each trial, subjects were instructed to indicate all the (contrabalanced) predefined target patterns in a visual display. For each trial, response times and spatial co-ordinates of the touched patterns were recorded and registrated. This allowed for the calculation of numerous indexes, such as accuracy and latency indexes, as well as a record of the exploratory pathway in function of the left and right hemifield.
RESULTS: ANOVA, independent sample t-tests and Chi-squared tests on the response times and spatial co-ordinates of the touched patterns, discriminated between the different patient groups with and without neglect or contralesional inattention.
CONCLUSION: The RVST is able to provide quantitative coefficients related to the spatio-temporal aspects of the visuo-motor exploratory behavior of the subjects, which are sensitive for lateralized spatial attentional imbalances.

Keywords: Attention, Stroke, Neuropsychological Assessment and Psychometrics

Correspondence address: Prof. Dr. Christophe Lafosse, Rehabilitation Hospital RevArte and KU Leuven, Neuropsychology, Drie Eikenstraat 659, B-3500 Edegem, Belgium. Tel.: +32 32106090; E-mail: Christophe.lafosse@revarte.be

**P2.15**

**Feature-based attentional cueing modulates neural patterns of conflict processing in the Stroop Task**

Julia Siemann, Manfred Herrmann and Daniela Galashan
*Department of Neuropsychology and Behavioral Neurobiology, University Bremen, Bremen, Germany*

The traditional view on interference effects induced by irrelevant distractors focuses on parallel processing of incoming information and a late locus of attentional selection. However, recent studies have shown that the interference effect will decrease when a cue stimulus indicates the relevant stimulus location. In the present study, the efficacy of feature cues directing attention to one feature of a multidimensional stimulus array was examined. A variant of the color-word Stroop task was applied, and the color of the target stimulus was validly or invalidly cued. It was hypothesized that previous knowledge about the relevant dimension would lead to an earlier inhibition of processing of irrelevant information. EEG data from 12 participants were analyzed. Both Stroop-related ERP components (N450 and parietal (conflict) slow positivity (SP)) were expected to be altered by the cue validity. The behavioral data analysis did not show an advantage for valid cues, presumably due to a ceiling effect. In contrast to invalid cues, valid cues resulted in an N450-like component but no conflict SP. The absence of the conflict SP may suggest that conflict processing could successfully be inhibited at an early stage of information processing. This finding corroberates the assumption that focused feature-based attention might lead to more effective attention selectivity.

Keywords: Attention, Electrophysiology (EEG/ERP)

Correspondence address: M.Sc. Julia Siemann, Department of Neuropsychology and Behavioral Neurobiology, University Bremen, Hochschulring 18, 28359 Bremen, Germany. Tel.: +49 0421 218 68743; E-mail: julia.siemann@uni-bremen.de

**P2.16**

**Does lateralized neglect change towards generalized inattention during a six-month follow-up?**

Laura Nurmi and Mervi Jehkonen
*University of Tampere, School of Social Sciences and Humanities, University of Tampere, Finland*

OBJECTIVE: The aim was to find out whether lateralized neglect changes towards generalized inattention during a six-month follow-up by comparing the location of omissions in Behavioural Inattention Test (BIT) cancellation tasks in two neglect groups after brain infarct.
METHODS: The study included 33 infarct patients with visual neglect and 62 healthy subjects (H). The location of omissions was compared in three BIT cancellation tasks. Based on location a laterality index ranging from 0 to 1 was calculated for each subject at the acute phase and at six months. The neglect patients were divided into two groups according to their acute phase laterality index. LN group patients showed lateral performance, while in the NLN group performance was symmetrical. The sum scores of the three BIT cancellation tasks were compared between the three groups to determine the severity of inattention.
RESULTS: The LN group included 12 patients and the NLN group 21 patients. At the acute phase sum scores were significantly lower in the LN group than in the NLN group. The laterality indices of the LN and the

NLN groups differed significantly at the acute phase, but no longer at six months. The LN group showed a significant change from lateral towards symmetrical performance during the follow-up. However, no generalized inattention was found in the LN group after six months, as the sum scores for the LN group and the H group did not differ significantly after the follow-up. In the case of two LN patients who according to the sum scores still showed visual inattention after six months, performance changed from lateral towards symmetrical during the follow-up.
CONCLUSION: In most patients, lateral neglect recovered fully during the follow-up and neither lateralized nor generalized inattention was found after six months. However, in those cases where inattention was still present at six months, lateralized neglect had changed towards generalized inattention during the follow-up.

Keywords: Attention, Neglect, Neuropsychological Assessment and Psychometrics

Correspondence address: Mrs MA Laura Nurmi, University of Tampere, School of Social Sciences and Humanities, Kalevantie 5, D-33014 University of Tampere, Finland. Tel.: +358 0503549432; E-mail: laura.j.nurmi@uta.fi

**P2.17**

### Transcranial magnetic stimulation: A new rehabilitation tool for neglect

Elena M. Marron[1] and Diego Redolar-Ripoll[2]
[1] *Universitat Oberta de Catalunya, Psychology-Neurosciencies, Barcelona, Spain*
[2] *Universitat Oberta de Catalunya, Neurosciences, Barcelona, Spain*

For many years clinical research has been conducted to develop new and effective rehabilitation strategies for neglect. Nowadays, rTMS is an important tool in this regard, and it should be considered as a therapeutic intervention in combination with conventional therapeutic approaches.
The positive outcomes of treatment of neglect and extinction based in the stimulation over the contralesional hemisphere started in the last 90' and first years of the XXI century (e.g. Brighina et al., 2003; Oliveri et al., 2001) and they still continue (e.g. Koch et al., 2008; Nyffeler et al., 2009; Shindo et al., 2006; Song et al., 2009).
Oliveri et al. have conducted numerous studies focused on ameliorating the symptoms of neglect in brain damage patients both with single pulse TMS (e.g. Oliveri et al., 1999; Oliveri et al., 2000) and rTMS (e.g. Oliveri et al., 2001; Brighina et al., 2003). Applying high rTMS online protocol, they observed that rTMS over parietal intact hemisphere reduced symptoms in neglect patients (Oliveri et al., 2001).
Brighinia et al. (2003) employed low frequency rTMS and applied it to neglect patients over two weeks. The performance in attentional tasks was better after treatment and this improvement remained stable 15 days later.
Shindo and co-workers (2006) and Song et al. (2009) also found a significant improvement with low frequency rTMS in attentional tasks and daily living activities in neglect patients. These benefits lasted 2–6 weeks.
Although there is quite evidence for consider TMS as a new therapeutic tool, further research with both healthy subjects and clinical samples remains essential to know the real TMS potential in the rehabilitation of neglect.

Keywords: Attention, Neglect, TMS/TCDS

Correspondence address: Mrs Elena M. Marron, Universitat Oberta de Catalunya, Psychology-Neurosciencies, Roc Boronat 117, D-08018 Barcelona, Spain. E-mail: emunozmarr@uoc.edu

**P2.18**

### Linguistic and visuo-perceptual modulations in sentence bisection: Evidence from patients with spatial neglect

Laura Veronelli[1], Maria T. Guasti[2], Lisa S. Arduino[3] and Giuseppe Vallar[2]
[1] *Casa di Cura Privata del Policlinico, Neuro-Rehabilitative Sciences, Milan, Italy*
[2] *University of Milano-Bicocca, Psychology, Milan, Italy*
[3] *Lumsa University, Rome, Italy*

In a line bisection task right-brain-damaged patients with unilateral spatial neglect (USN) show a rightward deviation with respect to the objective midpoint of the stimulus. By contrast, unimpaired participants (UP) report a slight leftward bias ('pseudoneglect'). Recent studies with USN patients and UP (Arduino et al.,

2010; Veronelli et al., in press) suggested the existence of partially independent mechanisms involved in word bisection, not only linguistic but also visuo-perceptual. Furthermore, the linguistic information contained in the final part of a word was shown to modulate the bisection performance in both UP and USN patients. The present study investigated whether and how linguistic features modulate manual bisection of sentences in USN patients. In Experiment I, 8 USN patients (N+), 8 right brain-damaged patients without neglect (N-) and 8 matched controls (C) were asked to set the subjective mid-point on sentences differing in their syntactic structure (wh- questions with the mandatory object in final or initial position of a sentence, yes-no questions, affirmative sentences), as well as letter strings and lines of comparable length, as control conditions. In Experiment II, 6 N+ patients and 6 C were required to bisect affirmative clauses, interrogative clauses, sentences with lexical and syntactic violations, as well as letter strings and lines. In both experiments, N+ patients showed an overall rightward error, larger than that exhibited by controls, modulated by stimulus type. Post-hoc tests revealed strong rightward deviations with lines, moderate with letter strings and slight with all types of sentences, even those in which lexical and syntactic violations were introduced. In conclusion, the linguistic nature of the stimulus induces facilitation in USN patients during bisection, reducing the rightward bias found with lines. Globally, both linguistic and visuo-perceptual mechanisms influence the bisection of sentences in spatial neglect.

Keywords: Attention, Neglect, Neuropsychological Assessment and Psychometrics

Correspondence address: Mrs Laura Veronelli, Casa di Cura Privata del Policlinico, Neuro-Rehabilitative Sciences, via Dezza 48, I-20144 Milan, Italy. E-mail: laura.veronelli@gmail.com

**P2.19**

**Attention allocation is impaired in job burnout**

Laura Sokka, Satu Pakarinen, Marianne Leinikka, Jussi Korpela, Kiti Müller and Minna Huotilainen
*Finnish Institute of Occupational Health, Brain and Technology, Helsinki, Finland*

Job burnout is a significant cause of decreased working capacity, concerning over 25% of working people. In working life, demands on various cognitive functions such as attention and working memory are present on a daily basis. However, studies on cognition and burnout are scarce, and have mainly concentrated on traditional neuropsychological assessment methods. The findings reported have been non-specific to burnout. Studies on neural mechanisms in burnout are almost absent. In our research, we studied attention and memory, by means of neuropsychological tests, and by recording electroencephalogram/event-related potentials (EEG/ERP) with a series of ERP paradigms. The participants ($N = 67$) were currently working people from two groups: burnout ($N = 41$), and healthy controls ($N = 26$). The groups were matched on age, gender, education, and working experience. For pre-attentive auditory processing, a newly modified version of the multi-feature MMN paradigm was used to record change-detection responses to various changes in natural speech sounds. In addition, we added novelty-like emotional variants of the standard to achieve an attention-catching effect. The ERP results showed that, between the two groups, there were differences in the P3a amplitudes and latencies, but not in the MMN responses. In neuropsychological tests, performance in recalling a word list after hearing it only once was significantly impaired in the burnout group as compared with the controls. No other differences were found between the groups in working memory, arithmetic tasks, vocabulary or delayed recall. We conclude that the pre-attentive change-detection is intact, whereas the attention allocation to novel auditory information is altered in burnout. The results indicate that the current clinical neuropsychological methods seem to be insensitive to verify the subjectively experienced symptoms of job burnout, but the reduced attention allocation can be shown by methods of brain research.

Keywords: Attention, Other Disorder/Samples, Electrophysiology (EEG/ERP)

Correspondence address: Mrs MA (Psych.), Researcher Laura Sokka, Finnish Institute of Occupational Health, Brain and Technology, Topeliuksenkatu 41 a A, FIN-00250 Helsinki, Finland. Tel.: +358 43 824 5039; E-mail: laura.sokka@ttl.fi

### P2.20

### Frontal volume reductions related to inattention and impulsivity in obese adolescents

Idoia Marqués-Iturria[1], I. García-García[1], P. González-Tartière[1], M.A. Jurado[1], M. Garolera[2], B. Segura[1], M. Vernet[2], M.J. Sender[2] and R. Pueyo[1]
[1]*University of Barcelona, Spain*
[2]*Consorci Sanitari de Terrassa*

Increasing evidence suggests frontal dysfunctions related to obesity. In addition, eating disorders present comorbidity for inappropriate attention and impulsiveness. The aim of this study was to assess frontal brain volumes in adolescent obesity and to investigate whether frontal structure was related to attention and impulsivity. Twenty-five adolescents (16 obese and 9 controls) between 12 and 19 ages (obesity: 13.88 ± 2.16 years, 5 males/11 females; control: 14.78 ± 2.22 years, 4 males/5 females) participated in this study. Frontal volumes were obtained with the automated procedure implemented on Freesurfer for cortex parcellation and then corrected dividing each volume by corresponding intracranial volume. Number of omissions and commissions on the Continuous Performance Test were used as attention and impulsivity scores. There were no group differences on demographic characteristics, yet age was included as covariate in all analyses in order to minimize its effect on adolescent changing brain. Frontal volumes were compared between groups by ANCOVA, and partial correlation analyses were performed to assess attention/impulsivity scores' relation with frontal structure. Results with $p \leqslant 0.01$ were considered significant. Lateral orbitofrontal, medial orbitofrontal and pars orbitalis in right hemisphere were reduced in obese group compared to controls. Regarding attention/impulsivity, there were no group differences in CPT performance (obesity: omissions = 11.88 ± 12.75, commissions = 23.69 ± 7.19; controls: omissions = 3.33 ± 3.00, commissions = 17.78 ± 8.18). In obese group medial orbitofrontal and pars orbitalis were negatively related to number of commissions ($r = -0.705$, $p = 0.003$; $r = -0.693$; $p = 0.004$), while lateral orbitofrontal volumes were negatively related to number of omissions ($r = -0.641$; $p = 0.010$). All in all, our study shows reduced frontal brain regions related to inattention and impulsivity that could be explained by a deficient cognitive control mechanism evident in obese adolescence.

Keywords: Attention, Other Disorder/Samples, Functional and Structural Neuroimaging/NIRS

Correspondence address: Mrs Idoia Marqués-Iturria, University of Barcelona, Pg Vall d'Hebron 171, 08035 Barcelona, Spain. E-mail: imarques@ub.edu

### P2.21

### Effects of broken affordance on visual extinction

Melanie Wulff[1] and Glyn W. Humphreys[2]
[1]*University of Birmingham, School of Psychology, Birmingham, United Kingdom*
[2]*Department of Experimental Psychology, University of Oxford, Oxford, UK*

Previous studies have shown that visual extinction can be reduced if two objects are positioned to 'afford' an action (Riddoch, Humphreys, Edwards, Baker and Willson, 2003). Here we tested if this affordance effect was disrupted by 'breaking' the affordance – if the object actively used in the action had a broken handle. We assessed the effects of broken affordance on recovery from extinction in five neuropsychological patients with pairs of objects positioned for left-handed and right-handed actions. Patients viewed object pairs that were or were not commonly used together. In the unrelated pair conditions, either two tools or two objects were presented. To disrupt the effect of action relations, we presented object pairs either with or without a broken handle. In line with previous research (e.g., Riddoch et al., 2006), recovery from extinction was greater for action-related than for unrelated pairs. Interestingly, the advantage for action-related objects over unrelated tools was apparent only when both handles were intact (unbroken handle condition) but not when the tool handle was broken (broken handle condition). Notable the advantage for action-related objects over unrelated objects was not affected by the handle completeness. In addition, patients reported more pairs when the stimuli were oriented for left-handed (incongruent) than for right-handed (congruent) actions, regardless of the object pair condition. Surprisingly, the effect of hand congruence was absent with broken stimuli. The data provide further support for visual and motor-related responses to affordance, with the effect being sensitive to handle completeness.

Keywords: Attention, Stroke, Neuropsychological Assessment and Psychometrics

Correspondence address: Mrs Melanie Wulff, University of Birmingham, School of Psychology, Edgbaston, UK-B15 2TT Birmingham, United Kingdom. E-mail: mxw127@bham.ac.uk

**P2.22**

**Subtle attentional problems and elevated postconcussive symptoms in children after mild traumatic brain injury**

Martina Studer[1], Barbara Goeggel Simonetti[2], Alexander Joeris[2], Steffen Berger[2], Maja Steinlin[2], Claudia Roebers[3] and Theda Heinks[2]
[1]*Department of Pediatric Neurology, University Children, Development and Rehabilitation, Bern, Switzerland*
[2]*University Children's Hospital, Bern*
[3]*University of Bern*

Although recent studies have repeatedly shown that mild traumatic brain injuries (mTBI) only subtly affect children's neurocognitive functioning, there is growing evidence, that children with mTBI display more postconcussive symptoms (PCS) in terms of somatic, cognitive and emotional complaints in the weeks after injury. The aim of this ongoing prospective short-term longitudinal study was to focus on working memory and attentional skills as well as on the trajectory of PCS in children (age 6–16 years) after mTBI and mild orthopedic injuries (OI). PCS were rated by the parents of mTBI children ($n = 46$, mean age at injury: 10.9 years) and OI children ($n = 35$, mean age at injury: 10.3 years) at four points in time T0 = at injury, T1 = 1 week after, T2 = 4 weeks after, T3 = 16 weeks after injury. Working memory performance and sustained attention were measured at T2 and T3. Preliminary results of this study reveal that in the first weeks after the injury, parents of children after mTBI observe significantly more PCS compared with parents of the OI patients. While working memory performance is comparable between mTBI and OI children at both times (T2, T3), mTBI children present with more commission errors during the sustained attention test. Furthermore, a significant negative relationship between the PCS score and working memory performance at T3 has been found for the mTBI group only. The results of this study show that in the weeks following the incident, mTBI children present with subtle attentional problems but no working memory impairment in standardized cognitive testing. The negative relationship between the elevated PCS ratings of parents of mTBI children and working memory performance at T3 might indicate that in everyday life working memory problems actually may exist and possibly affect school performance in some children.

Keywords: Attention, Traumatic Brain Injuries, Neuropsychological Assessment and Psychometrics

Correspondence address: Mrs Martina Studer, Department of Pediatric Neurology, Development and Rehabilitation, University Children, Inselspital, CH-3010 Bern, Switzerland. E-mail: martina.studer@insel.ch

**P2.23**

**Functional impairment of lateral occipital cortex contralateral to the lesion in a patient with visual object agnosia**

Radek Ptak[1], Francois Lazeyras[2], Armin Schnider[2], Stephane Simon[2] and Marie Di Pietro[2]
[1]*University Hospital Geneva, Division of neurorehabilitation, Geneva 14, Switzerland*
[2]*University Hospital Geneva, Switzerland*

BACKGROUND: Patients with visual object agnosia (VOA) fail to recognize the identity of visually presented objects despite preserved semantic knowledge. Current accounts of the disorder postulate subtle visual impairments, a deficit of form integration, or a semantic deficit confined to visual information. Though most patients have damage involving right or left occipitotemporal cortex it is unknown to what extent specific extrastriate areas contribute to the expression of VOA.
METHODS: We tested 4 healthy participants agematched to patient AL, who has VOA following a stroke affecting the left fusiform, lingual and inferior occipital gyri. We measured fMRI BOLD responses evoked by objects, faces or scrambled pictures.
RESULTS: The patient identified 46% of 260 visually presented line drawings, compared to 95% correct responses to verbal description of the same items. He produced satisfactory copies of objects that he was unable to identify. When asked to decide which of three objects belonged to the same category (e.g. two fruits), he was biased toward choosing the two visually similar objects (e.g. an orange and a ball). In addition, when asked to indicate whether an array of objects contained a given item (e.g. a button), he mostly chose a visual distracter (e.g. a wheel), but rarely a semantic or neutral distracter. While healthy participants showed robust activations of the lateral occipital cortex (LO) to objects as compared to scrambled pictures no such activation was observed either in the left (damaged) or right (preserved) LO of AL.
CONCLUSION: These results show that VOA is associated with a bilateral dysfunction of the LOC, even if structural damage is unilateral. The finding that visual identification errors mainly reflected global visual similarity suggests that the LOC contains representations of distinct object classes organised according to their form.

Keywords: Perception, Agnosia, Functional and Structural Neuroimaging/NIRS

Correspondence address: Mr PhD Radek Ptak, University Hospital Geneva, Division of neurorehabilitation, 26, av. de Beau-Sejour, CH-1211 Geneva 14, Switzerland. Tel.: +41 223823524; Fax: +41 223828338; E-mail: radek.ptak@hcuge.ch

### P2.24

### Mirror writing in MCI patients and normal elderly

Clara Calia[1], Robert D McIntosh[2], Maria Fara De-Caro[3] and Sergio Della Sala[2]
[1]*Queen Margaret University, Division of Psychology and Sociology, Edinburgh, UK*
[2]*University of Edinburgh, Human Cognitive Neuroscience, Psychology,, Edinburgh, UK*
[3]*University of Bari, Department of Basic Medical Sciences, Neurosciences and Sense Organs, Bari, Italy*

OBJECTIVE: The aim of this study was to evaluate the presence of MW in patients with Mild Cognitive Impairment (MCI) compared to elderly volunteers
MATERIALS AND METHODS: 24 MCI and 21 healthy controls underwent a screening for MW.
RESULTS: No group difference emerged. MW of individual letters was observed in 3 MCI patients, but also in 6 controls. However, one individual (F.C.) with MCI showed overt and severe MW when writing with her left hand. She (50 y. old, 7 y. of education) was not an outlier in terms of severity of cognitive impairment or any other clinical or demographic variable. She and a matched control group of 6 women were further assessed with a comprehensive battery of tests aimed at investigating her MW. She was much slower than the controls but as accurate in all tests. She did not commit more errors in mirror reading and reading backwards text on paper, in executing directional actions, and in left-right discrimination, nor did she perform differently when asked to write under the surface of a table or in writing with transparent titles. However, under dictation (8 words) she reversed two individual letters and one whole word, whereas the performance of the controls was flawless.
CONCLUSION: MW is a rare occurrence in MCI. The case of F.C. cannot be interpreted as resulting from a perceptual impairment, her performance is more easily accounted for by a deficit in the motor transformation of the engrams to be written with the non dominant hand or an impairment of the monitoring of directional errors.

References

[1] McIntosh R.D., Della Sala S. (2012). Mirror-writing. The Psychologist, 25: 742–6
[2] Balfour S, Borthwick S, Cubelli R, and Della Sala S. (2007). Mirror writing and reversing single letters in stroke patients and normal elderly. Journal of Neurology, 254: 436–441

Keywords: Perception, Mild Cognitive Impairment, Neuropsychological Assessment and Psychometrics

Correspondence address: Mrs Clara Calia, Queen Margaret University, Division of Psychology and Sociology, Queen Margaret University Drive, Musselburgh,, UK-EH21 6UU Edinburgh, UK. Tel.: +44 07906194175; E-mail: ccalia@qmu.ac.uk

### P2.25

### Effects of repetitive galvanic vestibular stimulation on neglect

Ingo Keller[1] and Katharina Volkening[2]
[1]*Schoen Klinik Bad Aibling, Neuropsychology, Bad Aibling, Germany*
[2]*Ludwig-Maximilians University, Department of General and Experimental Psychology, Munich, Germany*

Patients suffering from spatial neglect, exhibit none or deficient orienting and responding to stimuli in contralesional space. This greatly hampers the rehabilitation process. Recent studies have demonstrated that symptoms are alleviated during galvanic vestibular stimulation (GVS). However, only online and single-session effects were examined. This randomized controlled study investigated the effects of repetitive GVS on spatial neglect.

Treatment consisted of 10–12 sessions of GVS (1.5 mA, 20 min) and simultaneous visual search training, with three stimulation conditions: cathode on the right mastoid (CR), on the left mastoid (CL) and sham stimulation. Stimulation effects were assessed with the following measures: Behavioral Inattention Test (BIT), a visual-tactile search task (VST), the subjective visual (SVV) and tactile vertical (STV). Measures were assessed before, immediately after, as well as 2 and 4 weeks after the stimulation period.
Preliminary results of 16 patients (6 CL, 5 CR, 5 Sham) show similar improvements on the BIT and VST immediately after treatment. However, additional improvements were found for both GVS conditions until 2 weeks after stimulation. Repetitive left-, and right-cathodal GVS combined with visual search training led to immediate and long-term alleviations of neglect symptoms. Hence, transmastoid GVS seems to be a promising approach to treat spatial neglect.

Keywords: Perception, Neglect, TMS/TCDS

Correspondence address: Prof. Dr. Ingo Keller, Schoen Klinik Bad Aibling, Neuropsychology, Kolbermoorerstrasse 72, D-83043 Bad Aibling, Germany. Tel.: +49 8061 -9031590; E-mail: IKeller@Schoen-Kliniken.de

**P2.26**

**PD patients show intact action segmentation – a suport vector machine approach on clinical data investigating uncompromised abilities**

Anne-Marike Schiffer[1], Alejo J Nevado-Holgado[1] and Ricarda I Schubotz[2]
[1]*Department of Experimental Psychology, University of Oxford, Oxford, UK*
[2]*Westfaelische Wilhelms – Universitaet – Institut fuer Psychologie, Muenster, Germany*

In extention of predictive accounts of perception it has been proposed that segmenting the incoming sensory stream into discrete events relies on a mismatch between current predictions and the sensory input. In reference to dopaminergic reward prediction errors, it has been suggested that the hypothesized mismatch signals are dopamine-dependent. Since Parkinson's Disease (PD) results from a loss of dopaminergic midbrain cells, PD patients could be impaired in event segmentation.
However, midbrain dopaminergic or striatal activity has never been reported in fMRI studies on event segmentation. There is no obvious connection between cognitive symptoms of Parkinson's Disease and event segmentation.
The present study therefore tested the hypothesis that PD patients are not disorganized or compromised in their segmentation behaviour. To that end, 16 PD patients - tested both on and off L-Dopa medication - and 16 healthy matched controls repeatedly segmented a set of action movies in an internally guided self-paced fashion.
For each movie, we estimated the probability of a response at any given time, using a Fourier basis to generate the probability density function. To avoid a null-hyopthesis testing fallacy, we then trained a collection of common classifiers (e.g. support vector machines) on these probability functions, and tested their predictive accuracy through cross-validation. Additionally we calculated within session correlations for healthy controls and PD patiens on and off – medication.
The classifier results indicate that the PD patient's response behaviour is not different from healthy controls and does not differ according to medication status. The high correlation values show that segmentation behaviour is not less organized in PD, challenging the notion that action segmentation depends on dopaminergic prediction errors.

Keywords: Perception, Neurodegenerative Diseases, Neuropsychological Assessment and Psychometrics

Correspondence address: Dr. Anne-Marike Schiffer, Department of Experimental Psychology, University of Oxford, South Parks Road, GB-OX13UD Oxford, UK. E-mail: anne-marike.schiffer@psy.ox.ac.uk

**P2.27**

**Unseen fearful faces influence early facial encoding**

Caterina Bertini[1], Roberto Cecere[2], Martin Maier[2] and Elisabetta Làdavas[2]
[1]*Department of Psychology, University of Bologna, Bologna, Italy*
[2]*University of Bologna, Italy*

Threat-related signals are processed not only via striatal cortical areas, but also via a subcortical colliculo-pulvinar-amydala pathway, which presumably mediates implicit processing of fearful stimuli. To investigate the role of implicit fear processing mediated by the subcortical pathway at the early stages of facial encoding, we examined the ERPs elicited by fearful and happy faces presented to the intact visual field of right and left hemianopic patients, while fearful, happy or neutral faces were concurrently presented in their blind field. The results showed that in patients with lesions to the left hemisphere, the N170 amplitude was significantly increased only when a fearful face was presented in the blind visual field concurrently with a happy face in the intact visual field. This enhancement of facial encoding suggests a specialization of the subcortical pathway in processing unseen fearful stimuli. In addition, the lack of modulation with concurrent seen fearful faces reveal that the conscious perception of fear might inhibit the activation of the subcortical pathway. The modulation was only observed in patients with left hemisphere lesions, which favors the hypothesis that implicit processing of fearful signals (mediated by the subcortical pathway) is possible only when the right hemisphere is intact.

Keywords: Perception, Other Disorder/Samples, Electrophysiology (EEG/ERP)

Correspondence address: Mrs Caterina Bertini, Department of Psychology, University of Bologna, Viale Berti Pichat 5, D-40127 Bologna, Italy. E-mail: caterina.bertini@unibo.it

**P2.28**

**The effects of a multisensory training for visual field defects on spatial attention**

Caterina Bertini[1], Neil Dundon[2], Martin Maier[2] and Elisabetta Làdavas[2]
[1]*Department of Psychology, University of Bologna, Bologna, Italy*
[2]*University of Bologna, Italy*

The aim of the study is to assess the neural mechanisms underlying the recovery of visual function after multisensory stimulation (MS) in hemianopic patients. As a result of a hyperactivation of the intact hemisphere, with concurrent hypoactivation of the damaged hemisphere, patients focus their attention on the ipsilesional field and the contralesional field lacks sufficient attentional resources. The unbalanced excitability of the two hemispheres can be restored by a multisensory stimulation, mediated by the collicular-extrastriate pathway, which largely contributes to spatial orienting behaviors. We administered a course of MS treatment (Bolognini et al, 2005) to a sample of seven hemianopia participants for 4 hours daily, over a period of nearly 2 weeks and we collected both behavioural and electrophysiological data, before and after the intervention, in order to index the stimulation effects. The results showed an improvement of visual oculomotor exploration that allowed patients to efficiently compensate for the loss of vision. In addition, amplitudes of P3 event related potentials elicited by a simple visual detection paradigm were significantly reduced after the treatment when the stimuli were presented to the intact field. The behavioural improvement in the hemianopic field, and ERP amplitude reduction in the intact field, can be explained as driven by a shift in spatial attention away from the hyperactivated intact visual field.

Keywords: Perception, Other Disorder/Samples, Electrophysiology (EEG/ERP)

Correspondence address: Mrs Caterina Bertini, Department of Psychology, University of Bologna, Viale Berti Pichat 5, D-40127 Bologna, Italy. E-mail: caterina.bertini@unibo.it

**P2.29**

**Interhemispheric transmission in hemianopic patients: A role in visual recovery?**

Alessia Celeghin[1], Matteo Bendini[2], Tommaso Poscoliero[1], Silvia Savazzi[1] and Carlo Alberto Marzi[1]
[1]*University of Verona, Department of Neurological, Neuropsychological, Morphological and Movement Sciences, Verona, Italy*
[2]*Treviso Hospital, Department of Neuroradiology, Treviso, Italy*

In the present study we assessed the possibility that an important role for recovery of homonymous hemianopia resulting from damage to the visual cortical areas might be played by a cross-talk between the intact and the lesioned hemisphere through the cerebral commissures. One specific question that we tackled was the extent of interhemispheric transfer of visual information as a function of the location of the lesion causing hemianopia. Together with healthy control participants, we tested two chronic hemianopic patients: LS with a lesion roughly restricted to V1 and AE with a more anterior lesion including V1 and extrastriate areas. The behavioural test used was the Poffenberger Paradigm that enables to measure interhemispheric transfer time with a simple manual reaction time to lateralized visual stimuli. During behavioural performance we recorded visual event-related potentials (ERPs) to compare latency and amplitude of the ERPs components evoked by the direct or the commissural (callosal) pathway. We found that visual stimulation of the intact hemifield evoked a normal P1-N1 response in the intact contralateral hemisphere of both patients. However, when recording the indirect callosal response in the ipsilateral lesioned hemisphere we found a different result as a function of the site of the lesion. In LS there was a good indirect N1 response similar to that of controls while this was not the case in AE. A likely explanation of this dissociation is that in LS the widespread callosal connections adjoining the extrastriate areas in the two hemispheres are substantially spared while they are damaged in AE. Evidence for that was provided by diffusion tensor imaging (DTI). In conclusion, we think that the presence of a good visual commissural input to a lesioned hemisphere indicates a favourable prognosis for recovery of visual function in hemianopia. Preliminary results with these patients are encouraging in this regard.

Keywords: Perception, Other Disorder/Samples, Electrophysiology (EEG/ERP)

Correspondence address: Mrs Alessia Celeghin, Department of Neurological, University of Verona, Neuropsychological, Morphological and Movement Sciences, Strada Le Grazie, 8, I-37134 Verona, Italy. E-mail: alessia.celeghin@univr.it

**P2.30**

**Tactile body image in obesity**

Federica Scarpina[1], Gianluca Castelnuovo[2] and Enrico Molinari[2]
[1]*Istituto Auxologico Italiano IRCCS, San Giuseppe Hospital, Psychology Research Lab, Piancavallo (Oggebbio), Italy*
[2]*Department of Psychology, Catholic University of Milan, Milan, Italy*

Mental body representations are constructed from and reciprocally influenced by bottom-up sensory input (Serino and Haggard, 2010) and also by top-down cognitive and affective representations (Dijkerman and De Haan, 2007); for example higher level of body dissatisfaction was found to be related to severe tactile body image disturbance in Anorexia Nervosa (Keizer et al., 2011).

The aim of the present work was to observe possible disturbance in tactile aspects of body image in obese people, who were reported to show high level of dissatisfaction (Docteur et al., 2010).

Morbid obese subjects (BMI $> 40$), severe obese subjects (range of BMI $= 35$–$40$) and healthy weight subjects (range of BMI $= 22$–$25$) took part at the present study. They were asked to estimate the distance between two simultaneous tactile stimuli using their thumb and index finger, in absence of visual input; two different body parts were tested: the arm and the abdomen (Tactile Estimation Task – TET; Keizer et al., 2011). Moreover they were asked to state the level of satisfaction for their different body parts.

Preliminary results indicated that participants tended to overestimate perceptual distances on the arm compared to those on the abdomen; specifically morbid obese subjects appeared to be less precious respect to the two other groups. About psychological component, severe obese people expressed higher level of dissatisfaction respect to morbid obese subjects and healthy ones.

In obesity, body image disturbance extended to somatosensory perception; moreover affective components influence the construction of tactile body representation.

Keywords: Perception, Other Disorder/Samples, Other Methods

Correspondence address: Mrs PhD Federica Scarpina, Istituto Auxologico Italiano IRCCS, San Giuseppe Hospital, Psychology Research Lab, Via Cadorna, 90, Italy-28824 Piancavallo (Oggebbio), Italy. Tel.: +39 0323514353; E-mail: federica.scarpina@gmail.com

**P2.31**

**Learning modulates temporo-parietal processes during global recognition – from visual integration to object perception?**

Johannes Rennig[1], Marc Himmelbach[1], Elisabeth Huberle[2] and Hans-Otto Karnath[1]
[1]*University of Tübingen, Division of Neuropsychology, Tübingen, Germany*
[2]*Kantonsspital Luzern, Department of Neurology, Luzern, Switzerland*

Recent neuroimaging studies and reports from neurological patients with simultanagnosia suggested a crucial involvement of temporo-parietal brain areas in processing of hierarchical organized visual material. In the present study we asked the question if activity in temporo-parietal brain areas associated with global recognition can be modulated by extensive exposure to hierarchical stimuli. We performed an fMRI study with 24 healthy subjects including two neuroimaging sessions (pre- and post-training) with four psychophysical training sessions between them. In the two fMRI sessions four classes of stimuli were applied: two kinds of global recognition tasks and two additional tasks requiring recognition of local elements and regular objects. In the perceptual training sessions an adaptive psychophysical procedure with one class of global stimuli was conducted. Subjects showed improved behavioral performances in all four classes of stimuli with a particular advantage in both global recognition tasks. In a region of interest analysis in temporo-parietal brain activation clusters derived from the present investigation and a recent neuroimaging study we found significant integration related activation changes. These activation changes were specific to global stimuli while no systematic BOLD response changes were observable for recognition of local elements and regular objects. We address the observed activity modulations to a more efficient or generally different strategy of the brain in processing of global stimuli. We further conclude that perceptive learning of complex figures may be the connection between visual integration and object perception.

Keywords: Perception, Other Disorder/Samples, Functional and Structural Neuroimaging/NIRS

Correspondence address: Mr Dipl.-Psych. Johannes Rennig, University of Tübingen, Division of Neuropsychology, Hoppe-Seyler-Str. 3, D-72076 Tübingen, Germany. E-mail: johannes.rennig@uni-tuebingen.de

**P2.32**

**Occipital lesions disrupt the crossmodal enhancement of visual orientation sensitivity by looming sounds: A case study**

Làdavas[1], Roberto Cecere[1] and Vincenzo Romei[2]
[1]*University of Bologna, Italy*
[2]*University of Essex, Colchester, UK*

Approaching or looming sounds are potentially threatening stimuli with particular impact on visual processing. The early onset of crossmodal effects by looming sounds (Romei et al., 2009) and their impact on low-level visual processing (Leo et al., 2011) suggests that these interactions may take place already within low-level visual cortices. To investigate the contribution of early visual areas in mediating the effects of looming sounds on visual processing, we tested a patient with bilateral occipital lesion and spared residual portions of V1/V2 (SDV). Accordingly, SDV's visual perimetry revealed blindness of the central visual field with some residual peripheral vision. We tested the influence of looming, receding and static sounds on SDV line discrimination ability in the relatively preserved visual field or in the blind visual field. We found that SDV's visual orientation sensitivity was significantly enhanced when visual stimuli were paired with looming sounds, compared to other types of sound or presented alone. Crucially, this selective crossmodal effect was found when visual stimuli were presented in the relatively preserved visual field whereas no modulation in visual orientation sensitivity was found for stimuli presented in the blind visual field. These results provide direct evidence that primary visual areas are critically involved in crossmodal modulation of visual sensitivity. Although visual pathways (e.g. retino-colliculo-extrastriate) bypassing V1 can mediate the crossmodal enhancement of basic visual abilities such as detection of spots of lights (Làdavas, 2008), the crossmodal impact on more complex functions such as line orientation discrimination require functional activation of early visual areas.

Keywords: Perception, Other Disorder/Samples, Other Methods

Correspondence address: Mrs Làdavas, University of Bologna, Viale Berti Pichat 5, D-40127 Bologna, Italy. E-mail: elisabetta.ladavas@unibo.it

**P2.33**

**L-POST: development, validation and norming of a new screening test for perceptual organization**

Kathleen Vancleef[1], Elia Acke[1], Katrien Torfs[2], Christophe Lafosse[3], Johan Wagemans[1] and Lee De-Wit[1]
[1]*University of Leuven, Laboratory of Experimental Psychology, Leuven, Belgium*
[2]*University of Louvain, Psychological Sciences Re-*

*search Institute, Louvain-la-Neuve, Belgium*
[3]*University of Leuven, Belgium*

Neuropsychological tests of visual perception mostly assess high-level processes like object recognition. Object recognition however, relies on distinct mid-level processes of perceptual organization that are only implicitly tested in classical tests. To fill this gap, we developed the Leuven Perceptual Organization Screening Test (L-POST), in which a wide a range of mid-level phenomena are measured as independently as possible. The L-POST consists of 15 sub-tests screening for various aspects of perceptual organization, such as shape perception, figure-ground segmentation, and is sensitive to several grouping cues, such as common fate, collinearity, proximity and closure. To reduce cognitive load, a matching-to-sample task is used for all test items. Our free online test can be administered in 20–30 minutes, and a neglect-friendly version is available. The test has been validated with 40 brain-lesioned patients in which performance on the L-POST was compared to standard clinical tests of visual perception and other measures of cognitive function to evaluate convergent and divergent validity, respectively. The L-POST showed high sensitivity to visual dysfunction and decreased performance was specific to visual problems. In addition, we collected a norming sample of more than 1200 healthy control participants. In this sample, we observed a decline of L-POST scores with increasing age. No difference was observed between participants who filled in the test with and without supervision of a research assistant. We are now exploring the use of the L-POST in other neurological and psychiatric disorders that have been associated with altered visual perception (e.g. schizophrenia, dementia, autism). In conclusion, the L-POST is a valuable screening test for perceptual organization. It offers a useful tool for researchers and clinicians to get a broader overview of the mid-level processes that are preserved or disrupted in a given patient.

Keywords: Perception, Stroke, Neuropsychological Assessment and Psychometrics

Correspondence address: Mrs Kathleen Vancleef, University of Leuven, Laboratory of Experimental Psychology, Tiensestraat 102 box 3711, B-3000 Leuven, Belgium. E-mail: kathleen.vancleef@psy.kuleuven.be

### P2.34

### Cross-notational semantic priming between symbolic and non-symbolic numerosity in children

Mafalda Mendes and J. Frederico Marques
*Faculty of Psychology, University of Lisbon, Center for Psychological Research, Lisboa, Portugal*

Behavioral and neuroimaging studies have shown that the precision of numerical representations increases with age. Recent studies using the priming distance effect (faster responses to numerical targets when they are preceded by a numerical close prime) as a measure of numerical representations development, have found no differences in this effect between children and adults for both symbolic and non-symbolic notation, when prime and target stimuli were of the same notation. In this study, we tested whether children and adults exhibited cross-notation priming distance effects. Two groups of subjects, adults ($n = 23$) and 9–10 years-old children ($n = 19$) participated in this study. Three primed number comparison paradigms were performed in which subjects had to compare a given numerical target to the standard numerosity five: (1) Non-symbolic, in which prime and target were both non-symbolic (2) Symbolic, in which prime and target were both symbolic and (3) Cross-notation in which the prime and target were of different notation: prime symbolic and target non-symbolic or vice-versa. Numerosities used as primes and targets were 1 to 9 (except 5), presented as Arabic digits or collections of dots. The numerosities of prime and target pairs were manipulated in such a way as to create numerical distances from 0 to 4. Results revealed that priming effects were present and were quantitatively similar in both children and adults when the prime and target were of the same notation and when the prime was symbolic and the target non-symbolic. However, when the prime was non-symbolic and the target symbolic, priming only occurred in adults. Contrary to previous studies comparing priming effects in children and adults, this study suggests that there may be subtle differences in numerical representations between 10 years-old children and adults that were only evident from cross-notation priming effects.

Keywords: Other Functions/Disorders, Other Disorder /Samples, Other Methods

Correspondence address: Mrs Mafalda Mendes, Faculty of Psychology, University of Lisbon, Center for

Psychological Research, Alameda da Universidade, 1649-013 Lisboa, Portugal. E-mail: mmendes7@gmail.com

**P2.35**

**Body perception impairments in children with parieto-occipital lobe epilepsy**

Laurent Auclair[1], Christine Bulteau[2], Olivier Delalande[3], Georg Dorfmuller[3] and Isabelle Jambaque[4]
[1]*University Paris Descartes, INSERM U663, Institut of Psychology, Boulogne-Billancourt, France*
[2]*Foundation Ophtalmologique Adolphe de Rothschild, Pediatric Neurosurgery Department and INSERM U663, Paris, France*
[3]*Foundation Ophtalmologique Adolphe de Rothschild, Pediatric Neurosurgery Department, Paris, France*
[4]*University Paris Descartes, Institut of Psychology and INSERM U663, Paris, France*

OBJECTIVE: Neuropsychological studies consider that human body processing involve distinct body representations. However, there are few systematic researches on the development of the perception of the human body and its deficits in children. Here we investigated whether children with epilepsy primarily affecting the parieto-occipital (PO) cortex exhibited impairments in body representations.
METHODS: Twelve participants with PO lobe epilepsy (POLE) aged from 5 to 15 years were included All of them underwent presurgical or post surgical evaluation for partial epilepsy. Epilepsy onset in the first year of age (mean = 3 years) and originated from right (N = 6) or left side of the PO lobe. Their performances were compared to matched control children. Different tasks designed to assess multiple body representations such as lexico- semantic body representation, body structural representation and body schema were proposed. The experimental tasks covered naming objets and body parts, left-right body localization, hand and finger postures imitation, assembling body parts, body parts localization (with and without visual control), human figure drawing.
RESULTS: We showed that 89% of patients were impaired relative to controls on at least one task. No clear side differences emerged (left vs. right side epilepsy) for most of the task. However difficulties were more pronounced for right than for left POLE.
CONCLUSION: Early-onset posterior epilepsy can compromise the development of body representations. Further exploration of the deficits of human body perception and human body representation in children may have important theoretical as well as clinical implications.

Keywords: Visuospatial Abilities, Epilepsy, Neuropsychological Assessment and Psychometrics

Correspondence address: Dr Laurent AUCLAIR, University Paris Descartes, INSERM U663, Institut of Psychology, Av. Edouard Vaillant, 71, F-92100 Boulogne-Billancourt, France. E-mail: laurent.auclair@parisdescartes.fr

**P2.36**

**Cognitive functioning of adolescents with multiple sclerosis**

Gordana Mijalkovic Stojiljkovic and Dragana Vucinic
*Clinic for neurology and psychiatry for children and youth, Child neurology, Belgrade, Serbia*

PURPOSE: Multiple sclerosis (MS) that starts in adolescence makes problems of developmental changes and neurological aspects of illness. Hronicity of MS needs focus on cognitive functions because of intensive tasks in school achievement, academic skills, learning and adaptation processes.
AIM: Assessment of cognitive profile and abilities of adolescents with MS in the start of illness. Results could be used in design of neuropsychological screening and treatment.
METHODS: Global intellectual functioning in 16 MS patients from Serbia was investigated. Adolescents (male 5, female 11), aged 13–18 years, with patients with MS duration $>$ 2 years, and 2 groups depending on starting symptoms zone. Wecshler intelligence scales standardized in Serbian population were administered: REVISK (up to 15;11 years) and VITI (from 16 years>).
RESULTS: The testing performance was mostly in the category of high average intelligence (11/16), with the mean score IQ tot-109 (IQ tot spread: from 84–126). No strong association of clinical parameters with test results was concluded. The number of impaired test results correlates with acute phase of MS attack and duration of recovery period. Approximately 10% young patients showed impairment on 4 sub tests. Deficits were found on sub tests measuring visual analysis, perception accuracy, visual- constructive ability and cognitive speed. Better verbal achievements is evident in whole group of patients, and few error types were identified.
CONCLUSION: In this group of patients global cognitive functioning of adolescents with MS are preserved and on high functional level in most of the patients. Difficulties are registered in visual analysis, cognitive processing speed and perception accuracy. Some indicators of cognitive vulnerability during MS attack and recovery period needs support and following. Neuropsychological testing should be oriented to visually-constructive and visually-spacial abilities with wider battery of test.

Keywords: Visuospatial Abilities, MS/Demyelination

Correspondence address: Mrs Gordana Mijalkovic Stojiljkovic, Clinic for neurology and psychiatry for children and youth, Child neurology, Dr Subotica 6a, RS-381 Belgrade, Serbia. E-mail: gogo@sbb.rs

**P2.37**

**Comparing the effectiveness of Visuo-motor Training and Optokinetic Stimulation with Standardized Exploration Training in patients with hemispatial neglect**

Michael Opolka[1], Monika Harvey[2], Georg Kerkhoff[3] and Hendrik Niemann[1]
[1]*Neurologisches Rehabilitationszentrum Leipzig – Neuropsychologie, Bennewitz, Germany*
[2]*University of Glasgow, School of Psychology, Glasgow, UK*
[3]*Universität des Saarlandes, Arbeitseinheit Klinische Neuropsychologie, Saarbrücken, Germany*

We are currently running a randomized controlled study aiming to compare the effectiveness of three different interventions aimed at rehabilitating hemispatial neglect: (1) Visuo-motor Training (VMT) (2) Optokinetic Stimulation (OKS) and (3) Standard Exploration Training (SET) (control group).
A total of 60 patients with neglect following either stroke or intracerebral bleeding are recruited from a German Neurological Rehabilitation Centre and randomly assigned to one of the three groups. Each intervention consists of 10 training sessions conducted on 10 consecutive days.
Rehabilitation effects are measured immediately after the training and at a 3-month-follow-up. Outcome measures include visual exploration abilities (Neglecttest (Fels and Geissner, 1997), general attention (Testbatterie zur Aufmerksamkeitsprüfung (TAP) (Zimmermann and Fimm, 2009)), reading (Saarbrücker Lesetexte (Kerkhoff, Wimbauer, Reinhart, 2012), awareness (Catherine Bergego Scale (CBS) (Azouvi et al., 1996)), activities of daily living (Stroke Impact Scale Version 3.0 (Duncan et al., 1999) and an assessment of depression (Hospital Anxiety and Depression Scale – German Version (Herrmann-Lingen, Buss and Snaith, 1995).
While data collection is ongoing, first results on about half of the intended sample size show improvements depending on intervention method applied. As the SET treatment is similar in its nature to cancellation tasks, patients in this group show large benefits in visual paper-pencil exploration tasks, while patients in the VMT intervention group improve most in computerized tasks assessing omissions. The VMT group members also benefit most in line bisection tasks. Reading abilities show the greatest improvement in both OKS and SET patients, while OKS patients also become increasingly aware of their neglect symptoms.
Data from the remaining patients should strengthen the reported effects and we also hope to demonstrate distinct effects on activities of daily living and depression.

Keywords: Visuospatial Abilities, Neglect, Neuropsychological Rehabilitation

Correspondence address: Mr Michael Opolka, Neurologisches Rehabilitationszentrum Leipzig – Neuropsychologie, Muldentalweg 1, D-04828 Bennewitz, Germany. Tel.: +49 03425 888231; E-mail: michael_opolka@web.de

**P2.38**

**Prose reading in neglect**

Roberto Cubelli[1], Carlo Cisari[2], Sergio Della Sala[3] and Nicoletta Beschin[4]
[1]*University of Trento, Psychology and Cognitive Science, Rovereto, Italy*
[2]*b) Physical and Rehab Medicine, Piemonte Orientale University, Novara, Italy*
[3]*University of Edinburgh, c) Human Cognitive Neuroscience, Psychology, Edinburgh, UK*
[4]*Hospital S. Antonio Abate Gallarate, d) Clinical Neuropsychology Unit, Somma Lombardo (Va), Italy*

BACKGROUND: Reading sentences or texts has been shown to be a very sensitive measure of neglect [1]. However, most clinical and theoretical studies focused on reading single words or non-words [2] rather than prose reading (PR).
AIMS: To assess PR in right hemisphere damaged patients and the relationship between PR and other measures of neglect and its severity, or between PR and neglect dyslexia.
METHODS: 30 stroke patients showing clear symptoms of spatial neglect were asked to read aloud a short prose passage (130 words in 11 lines, 142 mm long) and 105 single words. They underwent 14 conventional tests for spatial neglect.
RESULTS: 21/30 participants (70%) omitted words or letters at the beginning of the text (left end) showing PR neglect, they on average attended to 108.4 mm of text omitting text on the left end side in 5.5 lines, showing no top-bottom gradient in reading individual lines. The two subgroups did not differ significantly in age, education, days post-onset, or MMSE, Verbal Judgments and Cognitive Estimations scores as well as presence of anosognosia. The anatomical substrates of PR neglect are centered on the insula, putamen, superior temporal lobe and Rolandic operculum. No specific lesion emerged which differentiates individuals presenting with and without prose PR neglect. No specific tests assessing neglect could predict the presence of PR neglect, however neglect severity was associated to PR neglect. Clear double dissociations emerged between PRN and neglect dyslexia.
CONCLUSION: A considerable proportion of patients with spatial neglect omitted the beginning of the lines in reading text. The findings provide evidence for the functional independence of word processing and space exploration while reading, and argue for the inclusion of prose reading in standard neglect assessment.

References

[1] Caplan B. (1987) Assessment of unilateral neglect:a new reading test. JCEN, 9: 359-64
[2] Vallar G. et al. (2010) Neglect dyslexia: A review of the neuropsychological literature. Exp Brain Res, 206: 219-235

Keywords: Visuospatial Abilities, Neglect, Neuropsychological Assessment and Psychometrics

Correspondence address: Mr Prof Roberto Cubelli, University of Trento, Psychology and Cognitive Science, Via Rovereto, D-21020 Rovereto, Italy. E-mail: roberto.cubelli@unitn.it

**P2.39**

**Displacement of small numbers on a vertical number line in spatial neglect**

Urszula Mihulowicz[1], Hans-Christoph Nuerk[2], Elise Klein[3], Klaus Willmes[4] and Hans-Otto Karnath[5]
[1]*University of Tübingen, Division of Neuropsychology, Center of Neurology and Hertie Institute for Clinical Brain Research, Tübingen, Germany*
[2]*Department of Psychology/IWM-KMRC Knowledge Media Research Center, University of Tuebingen, Tuebingen, Germany*
[3]*IWM-KMRC Knowledge Media Research Center, Tuebingen, Germany*
[4]*University Hospital RWTH Aachen, Section Neuropsychology, Department of Neurology, Aachen, Germany*
[5]*University of Tübingen, Division of Neuropsychology, Center of Neurology and Hertie Institute for Clinical Brain Research, Tuebingen, Germany*

Previous studies investigating the mental number line in neglect patients came to inconsistent conclusions about its spatial representation. This could be due to confounding factors of working memory or the indirect way of investigating magnitude representation in the number interval bisection paradigm. Here we explored the spatial representation of numbers independently of the working memory load. Moreover, due to the vertical orientation of the number lines presented, physical and representational aspects could be disentangled.
Sixty-six acute stroke patients (31 LBD, 29 RBD without, and 6 RBD with neglect) performed a vertical number line task, in which they were asked to place 13 two-digit numbers on vertically oriented lines with 0 marked at the bottom and 100 at the top. Performance of the LBD patients and RBD patients without neglect did not differ at any of the number points. As commonly reported in the healthy adult population, the representation was nearly linear, whereby numbers closest to the provided (0 and 100) as well as to the strategic (50) anchors were indicated most precisely. In contrast, RBD patients with neglect tended to overestimate the position of numbers under 50, which are assumed to be placed on the left side of the mental number line, thus corresponding to the idea of shifting them to the imaginary right side. Importantly, this was not the case for numbers close to the anchors. For larger numbers (right side of the mental number line), RBD patients with neglect did not differ from the other groups.

The results argue for a spatial character of the mental representation of numbers, which is, however, influenced by spatial-numerical task strategies. In case of neglect patients, this representation seems to be biased towards the ipsilesional side; the latter proves to be independent of the physical orientation of the task stimuli.

Keywords: Visuospatial Abilities, Neglect, Neuropsychological Assessment and Psychometrics

Correspondence address: Mrs Urszula Mihulowicz, University of Tübingen, Division of Neuropsychology, Center of Neurology and Hertie Institute for Clinical Brain Research, Hoppe-Seyler-Str. 3, D-72076 Tübingen, Germany. E-mail: urszula.mihulowicz@uni-tuebingen.de

**P2.40**

**Bilateral 2mA tDCS stimulation induces only weak shifts in attentional bias**

Gemma Learmonth, Christopher Benwell, Monika Harvey and Gregor Thut
*University of Glasgow, School of Psychology, Glasgow, Scotland*

tDCS over parietal cortex induces transient, neglect-like effects in healthy participants, and more so with dual parietal than right parietal tDCS, informing models of spatial attention (Giglia et al., Brain Stimulation, 4, 2011).
We employed dual parietal tDCS to further study the time-course of these effects using an online approach. A computerised landmark task was presented to 18 right-handed adults, which involved deciding which end of a pre-bisected horizontal line was shorter. Participants completed a total of 10 blocks (1 pre-tDCS, 6 on-line tDCS, 3 post-tDCS). Each subject performed 3 sessions $>$ 24 hrs apart: 1) Left-anode right-cathode 2) Left-cathode right-anode 3) Sham. TDCS consisted of 2mA stimulation over P5/6 for 20 minutes.
In contrast to Giglia et al. (2011), we only found a weak left to rightward shift in spatial attention driven by the left-anode right-cathode montage compared to Sham, as well as a slight increase in left bias with right-anode left-cathode montage. These small non-significant effects were only briefly evident at the end of 20 min stimulation, and returned to baseline by the subsequent block 4 min later. To study whether this may be due to online stimulation (and possible functional compensation during tDCS), we ran the same paradigm, but using off-line stimulation, in a further 6 participants, which also failed to show effects.
We conclude that the dual parietal tDCS approach does not necessarily give rise to clear effects on spatial biases, even if tDCS parameters are above previously effective parameters (2 vs 1 mA and 20 vs 15 min, in our vs Giglia et al.'s study). This indicates that tDCS effects are not linearly related to stimulation parameters. We hope that these findings will inform the design of future attentional tDCS studies and are currently investigating different density and stimulation sites to also inform potential tDCS trials on patients suffering from hemispatial neglect (see also Sparing et al., Brain, 132, 2009).

Keywords: Visuospatial Abilities, Neglect, TMS/ TCDS

Correspondence address: Mrs Ms Gemma Learmonth, University of Glasgow, School of Psychology, 58 Hillhead Street, UK-G12 8QB Glasgow, Scotland. E-mail: gemmal@psy.gla.ac.uk

**P2.41**

**Limb Activation Treatment and Contralesional Arm Vibration: When two is better than one in reducing left-neglect signs**

Marco Pitteri[1], Giorgio Arcara[1], Laura Passarini[1], Francesca Meneghello[1] and Konstantinos Priftis[2]
[1]*IRCCS San Camillo Hospital, Laboratory of Neuropsychology, Lido-Venice, Italy*
[2]*Department of General Psychology, University of Padova, Padova, Italy*

One of the most disabling neuropsychological syndromes following right-hemisphere lesion is left neglect (LN). LN patients fail to respond, report, or orient to stimuli in the left contralesional side of space (Heilman et al., 2003). Although some spontaneous recovery occurs in the majority of LN patients after stroke, LN signs remain severe in many patients and may persist in the chronic phase. Combining different rehabilitation techniques may increase the effectiveness of cognitive treatments (e.g., Kerkhoff and Schenk, 2012). In the present study, we evaluated the effects of the Limb Activation Treatment (LAT) alone and in combination with the Contralateral Arm Vibration (CAV). We conceived them as techniques that both prompt the joint activation of the lesioned right hemisphere because of the activation (with the LAT as an active technique) and the stimulation (with the CAV as a passive technique) of the left hemibody. To test the effect of the simultaneous use of these two techniques (i.e., LAT and CAV) on visuo-spatial aspects of LN, we describe the case of an LN patient (GR), who showed high intra-individual variability (IIV) in performance. Given the high IIV of GR, we used an ABAB, repeated-measures design to better define the effectiveness of combining LAT and CAV, as a function of time. The results showed an improvement of GR's performance on the Bells test during the combined application of LAT and CAV with respect to the application of LAT alone. No effect on line bisection and on picture scanning tests was observed. Our preliminary positive results might provide some new evidence on the possibility to obtain additive effects of cognitive rehabilitation procedures, if they share a common theoretical framework and, consequently, a common network of neural activation subserving the target function.

Keywords: Visuospatial Abilities, Neglect, Neuropsychological Rehabilitation

Correspondence address: PhD Marco Pitteri, IRCCS San Camillo Hospital, Laboratory of Neuropsychology, Via Alberoni, 70, IT-30126 Lido-Venice, Italy. E-mail: marco.pitteri@ospedalesancamillo.net

**P2.42**

**Comparison among Visual Scanning Training, Limb Activation Treatment, and Prism Adaptation for rehabilitating left neglect: No winner, all winners in the competition**

Marco Pitteri[1], Laura Passarini[1], Cristina Pilosio[1], Francesca Meneghello[1] and Konstantinos Priftis[2]
[1]*IRCCS San Camillo Hospital, Laboratory of Neuropsychology, Lido-Venice, Italy*
[2]*University of Padova, Department of General Psychology, Padova, Italy*

Left neglect (LN) is one of the most frequent and disabling neuropsychological syndromes following right-hemisphere damage. LN patients fail to report, orient to, or verbally describe stimuli in the contralesional, left side of space (Heilman et al., 2003). Although some spontaneous recovery occurs in the majority of LN patients after stroke, LN remains severe in many patients and may persist in the chronic phase (Nijboer et al., 2013). Thus, LN is one of the major factors underlying poor functional outcome (Buxbaum et al., 2004). Over the past 60 years, many different treatments for rehabilitating LN have been conceived and tested (Riestra & Barrett, 2013). We compared, for the first time, the overall and possible differential effects of three of the most widely-used LN treatments: Visual Scanning Training (VST), Limb Activation Treatment (LAT), and Prism Adaptation (PA). Thirty-two LN patients were assigned in quasi-random order into the three groups (VST, LAT, PA). LN patients' performance on everyday life tasks was assessed four times: A1 and A2 (i.e., the two pre-treatment assessments); A3 and A4 (i.e., the two post-treatment assessments). LN patients were treated for the same number of sessions (i.e., 20), for a period of two weeks. The results showed that improvements were present in the majority of the tests assessing the peripersonal space in everyday life activities after each rehabilitation treatment. No generalization of positive improvement to the personal and extrapersonal spaces was observed. Our findings were independent of unspecific factors and lasted for at least two weeks following the end of the treatments. Nonetheless, there were no interactions between LN treatments and assessments, suggesting that none of the three treatments is better than the others in treating LN. We suggest that VST, LAT, and PA can be considered as valid rehabilitation interventions for LN and can be used for ameliorating LN signs.

Keywords: Visuospatial Abilities, Neglect, Neuropsychological Rehabilitation

Correspondence address: Ph.D. Marco Pitteri, IRCCS San Camillo Hospital, Laboratory of Neuropsychology, Via Alberoni, 70, IT-30126 Lido-Venice, Italy. E-mail: marco.pitteri@ospedalesancamillo.net

**P2.43**

**Object-centered spatial neglect: The effects of the principal axis and the canonical orientation**

Eric Siéroff[1], Feriel Batouche[2], Delphine Levy-Bencheton[3], David Nyckees[1], Farah Stefano[4] and David Adams[2]
[1]*Paris Descartes University, Institute of Psychology, Boulogne-Billancourt, France*

[2]*Hopital Kremlin-Bicetre, Neurology, Kremlin-Bicetre, France*
[3]*INSERM, Centre de Recherche en Neurosciences de Lyon, France*
[4]*Paris Descartes University, Institute of Psychology, Paris, France*

The coordinates of the spatial deficit in unilateral spatial neglect have been largely debated. Although most studies describe the deficit in terms of egocentric coordinates, several cases of patients have been described with a deficit at the level of allocentric (or intrinsic) coordinates. These cases are described as object-centered spatial neglect, or object neglect. Such patients with a right hemisphere lesion ignore the left part of objects even if objects have been rotated, so that this left part is at least partially located in the right space of patients. In the present study, we evaluated the occurrence of object neglect in a group of 12 patients showing left unilateral spatial neglect (diagnosed with usual test batteries); 12 controls also participated to the study. We used drawings of 40 objects, which contours were highlighted in different colors for each of the 4 quadrants centered on the object; 16 possible colors were used. Objects were either presented in normal orientation or rotated by 45° on the right. Participants had to name the different colors. By comparing naming efficiency of the different quadrants in normal and rotated objects, we could determine the existence of some object neglect. Ten out of our 12 patients had object neglect, showing that this deficit is not rare. Also, we used different types of objects, varying the principal axis (vertical, horizontal or absent) and the strength of the canonical orientation (strong, like in a refrigerator or a cup; or weak, like in scissors or a strawberry), with the hypothesis of stronger object neglect in objects showing a vertical axis, and a strong canonical orientation. However, results were rather heterogeneous among the different patients. Finally, we selected in our sample the most appropriate drawings of objects for the diagnosis of object neglect, and proposed that this smaller sample could constitute a good clinical test of object neglect.

Keywords: Visuospatial Abilities, Neglect, Neuropsychological Assessment and Psychometrics

Correspondence address: Mr Professor Eric Siéroff, Paris Descartes University, Institute of Psychology, 71, avenue Edouard-Vaillant, F-92100 Boulogne-Billancourt, France. Tel.: +33 1 55 20 59 29; E-mail: eric.sieroff@parisdescartes.fr

**P2.44**

**A curious impairment of manual motor imagery in a skilled deliberate mirror-writer**

Robert McIntosh, Yin-Wen Kuo and Sergio Della Sala
*University of Edinburgh, Human Cognitive Neuroscience, Psychology, Edinburgh, UK*

KB is a man who has practiced mirror-writing for more than fifty years. He writes fluently forward or backward with either hand, both upright and inverted. He is most practiced and adept at left-handed upright mirror writing. We explored whether his unusual motor abilities are associated with unusual performance on mental rotation tasks. On a task requiring speeded judgments of whether letters at various orientations are forward or reversed, KB performed within age-matched limits. But when required to judge whether a picture of a hand or foot, presented at various orientations, was from the left or the right side of the body, he showed a surprising impairment. He was abnormally slow and inaccurate at judging the laterality of hands, but not feet, and this was reliable across multiple sessions. This laterality judgment task is widely used to assess motor imagery, as it is known that people solve the task by mentally rotating their own hand or foot to confirm a match to that in the picture. We tentatively suggest that KB's ability for (and/or history of) executing right hand actions with the left hand (i.e. mirror writing) may entail an abnormal degree of overlap in the neural motor representations of his hands, creating introspective confusion over which hand he has mentally rotated to make the match.

Keywords: Visuospatial Abilities, Other Disorder/ Samples, Neuropsychological Assessment and Psychometrics

Correspondence address: Mr Dr Robert McIntosh, University of Edinburgh, Human Cognitive Neuroscience, Psychology, 7 George Square, UK-EH89JZ Edinburgh, UK. Tel.: +44 131 6503444; E-mail: r.d.mcintosh@ed.ac.uk

### P2.45

### Neuroanatomy of space, body and posture perception in patients with right hemisphere stroke

Arnaud Saj[1], Marc Rousseaux[2], Jacques Honoré[2] and Patrik Vuilleumier[3]
[1]*University hospital of Geneva, Neurology, Geneva, Switzerland*
[2]*Department of Neuro-rehabilitation, Lille University Medical Center, Lille, France*
[3]*Neuroscience Department, University of Geneva, Geneva, Switzerland*

OBJECTIVE: To specify the neuroanatomical correlates of biases in the representations of the gravitational vertical (subjective vertical, SV) and body axis (subjective straight ahead, SSA), as well as postural difficulties, in patients with hemispheric stroke.
METHODS: The analysis focused on right hemisphere lesions in 21 neglect patients and 21 non-neglect patients (using MRIcro® software) and related performance in two experimental tasks (SV and SSA) and a clinical balance assessment. Voxel-based lesion-symptom mapping was used to highlight brain areas in which lesions best explained the severity of task biases ($p < 0.01$).
RESULTS: The bias in the representation of body orientation was found to be strongly related ($p < 0.01$) to lesions of the anterior parietal cortex and the middle part of the superior temporal gyrus. The SV errors were associated with more widespread lesions of the posterior parietal and temporal cortices. Imbalance was preferentially associated with lesions of the posterior insula and the adjacent temporoparietal cortex.
CONCLUSION: This constitutes the first report of cortical dissociation of biases of body-centered and gravitational vertical representations, which may reflect the differential involvement of these brain regions in spatial information processing. The lesions involved in representation biases (especially of the SV) and postural difficulties overlapped to some extent in the temporoparietal, superior temporal and posterior insular regions of the cortex.

Keywords: Visuospatial Abilities, Stroke, Functional and Structural Neuroimaging/NIRS

Correspondence address: Dr Arnaud Saj, University hospital of Geneva, Neurology, Rue Grabrielle-Perret-Gentil, CH-1820 Geneva, Switzerland. E-mail: arnaud.saj@unige.ch

### P2.46

### Temporal dynamics of error correction in a double step task in patients with a lesion to the lateral intra-parietal sulcus

Stefan Van der Stigchel[1], Robert D. Rafal[2] and Janet H. Bultitude[3]
[1]*Utrecht University, Experimental Psychology, Helmholtz Institute, Utrecht, The Netherlands*
[2]*Bangor University, School of Psychology, Bangor, United Kingdom*
[3]*University of Oxford, Centre for Functional Magnetic Resonance Imaging of the Brain, Oxford, United Kingdom*

Five patients with lesions involving intra-parietal cortex were tested in a rapid version of the double step paradigm to investigate the role of the intra-parietal cortex in the rapid, online, updating of a saccade program. Saccades were executed to a single target in either the contra- or the ipsilesional visual field. In two thirds of the trials, a step change in target position required that the saccade shifted to a new location within the same field but in the contra- or the ipsilesional direction, allowing us to investigate whether patients are able to update their saccade program given new exogenous information about the required endpoint of the saccade. This set-up resulted in three types of initial saccades: saccades to the target on no-step trials, uncorrected saccades to the original target location on step trials and corrected saccades to the new target location on step trials. Furthermore, if the updating of the original eye movement program failed, patients performed a second saccade to the new target location that required a rapid error correction. The analysis of the double-step task indicated that latencies for all trial types were larger when saccades were directed to the contralesional versus the ipsilesional field. Furthermore, longer latencies were required for patients to initiate a corrective second saccade after making an uncorrected first saccade in their contralesional compared to ipsilesional field. There were no differences in the ultimate landing positions of the eye movements for such corrected saccades. These results reveal that deficits in updating of saccade programs only seem to be present after the gaze shifts to a new location, pointing to a role of intra-parietal cortex in the processes involved in updating information when a new reference frame is required. In conclusion, the paradigm deployed in the current study allows for a refinement of the role of the intra-parietal cortex in the updating of saccade programs.

Keywords: Visuospatial Abilities, Stroke, Other Methods

Correspondence address: Mr Stefan Van der Stigchel, Utrecht University, Experimental Psychology, Helmholtz Institute, Heidelberglaan 2, 3523PK Utrecht, the Netherlands. E-mail: s.vanderstigchel@uu.nl

### P2.47

### Visuo-spatial and motor imagery impairments following left posteromedial lesion: a case study

Gennaro Ruggiero[1], Francesca Frassinetti[2], Alessandro Iavarone[3] and Tina Iachini[1]
[1]*Department of Psychology, Second University of Naples, Caserta, Italy*
[2]*Department of Psychology, University of Bologna, Bologna, Italy*
[3]*Neurology and Stroke Unit, CTO Hospital, Napoli, Italy*

In this study we present a 69 year-old right-handed man (M.S.) who, after a stroke on the left hemisphere, reported lesions to the posteromedial areas, such as the posterior cingulate gyrus (BA 30) and the precuneus (BA 31). Left precuneus and its surrounding areas seem to be relevant in visuo-spatial processing, action simulation, mental imagery and mental navigation processes. Given the rarity of M.S.'s lesion and due to his good performance on a wide-ranging neuropsychological assessment, this patient may help us to shed more light on the role played by this neural network in visuo-motor/visual/motor imagery processes. To this end, the performance of M.S. and 12 healthy controls were compared on three imagery tasks requiring different levels of visual and motor control. After walking on a path (there were three paths of different length), participants were submitted to the three imagery tasks: (i) 'imagine a disc moving on the path' (i.e., visual mental scanning requiring visuo-spatial control over a spatial trajectory); (ii) 'imagine walking on the path' (i.e., visuo-motor imagery requiring visuo-motor and motor cooperation); (iii) 'imagine running in place' (motor imagery requiring action simulation without visuo-spatial control). Imagined movement time was analyzed by means of Crawford and Garthwaite's modified t-test to small control sample size. Results showed that the lesion influenced motor imagery and visuo-motor imagery, but not visuo-spatial imagery. This finding reveals that these cerebral structures, and in particular the precuneus, play a key role in simulation processes linked to motor imagery but not visuo-spatial imagery. The theoretical implications of these results will be discussed.

Keywords: Visuospatial Abilities, Stroke, Neuropsychological Assessment and Psychometrics

Correspondence address: Mr Gennaro Ruggiero, Department of Psychology, Second University of Naples, Viale Ellittico, 31, D-81100 Caserta, Italy. E-mail: gennaro.ruggiero@unina2.it

### P2.48

### Egocentric impairment in episodic memory following parietal damage

Paresh Malhotra[1], Anna Sofia Musil[2], Korina Li[1] and Charlotte Russell[2]
[1]*Imperial College London, Division of Brain Sciences, London, UK*
[2]*Department of Psychology, Brunel University, London, UK*

Although there is a great deal of support from functional imaging studies for parietal lobe involvement in episodic memory, until now there has been very little experimental evidence from neuropsychological studies with patients suffering from focal parietal damage to suggest that parietal integrity is necessary for episodic memory function. Given the key role of frontoparietal networks in visuospatial representation, we hypothesised that the parietal lobe may play a crucial role in episodic memory with respect to the recollection of details from an egocentric perspective. We tested two patients with right parietal stroke, and compared them with control subjects, on a novel experimental task utilising a head-mounted camera to record images from the participants' perspective. This enabled us to directly evaluate the integrity of memory from the individuals' own personal point of view. All participants also carried out standard episodic memory tasks. We found that parietal patients were able to recall information as well as control subjects when they were asked to recollect whether particular items had been present in an array or where items had been presented in relation to each other. They were also equivalently accurate in all standard tests of episodic memory. However, these patients were severely impaired when attempting to judge from which perspective they had viewed the scenes. This specific impairment was striking and in contrast to the relative ease of this condition for the control groups. These results provide the first evidence for a specific deficit in episodic memory in patients with parietal damage, and highlight the importance of the original spatial context in this type of memory.

Keywords: Visuospatial Abilities, Stroke, Neuropsychological Assessment and Psychometrics

Correspondence address: Dr Paresh Malhotra, Imperial College London, Division of Brain Sciences, St Dunstan, W6-8RP London, UK. E-mail: P.malhotra@ imperial.ac.uk

**P2.49**

**Analysis of movement trajectories using a dynamic time warping algorithm: neuropsychological relevance of similarities of movement patterns**

David Flentge[1], Kohsik[2], Dyck[2], Schäbitz[3], Hellbach[4], Botsch[2] and Piefke[5]
[1]*University of Bielefeld, Faculty of Psychology and Sports Science, CITEC, Bielefeld, Germany*
[2]*University of Bielefeld*
[3]*Clinic for Neurology EvKB, Bielefeld*
[4]*University of Applied Sciences, Dresden*
[5]*Witten/Herdecke University, Witten/Herdecke*

There is evidence from clinical neuropsychology that visual-spatial abilities can be diagnosed and trained with optimum experimental control using Virtual Reality (VR) technologies (Bohil et al. 2011). The analysis of movement trajectories in real life-like VR scenarios may complement neuropsychological data by introducing important information on strategies of navigation, spatial knowledge of the virtual environment, and executive control of task performance. However, methods for the analysis of movement trajectories that go beyond measures of the length of trajectories and the time needed to accomplish a task are still lacking. We here used a dynamic time warping (DTW) algorithm for the analysis of similarities between movement trajectories in healthy human subjects ($N = 7$) and patients after stroke ($N = 7$) during an 8-day training of shopping in a VR supermarket. We expected that knowledge about similarities between trajectories of good and bad performers in the virtual supermarket may give diagnostic information on visual-spatial capabilities and executive functions complementing traditional neuropsychological measures. We found that similarity of trajectories increased across training days for both patients and controls. Explorative analyses indicated that similarity measures may differentiate between good and bad performers in the VR supermarket. Similarity values did not correlate with standard neuropsychological measures. Although DTW data show some evidence that similarities of movement trajectories are relevant for the analysis of visual-spatial abilities it remains unclear how this finding can be integrated into neuropsychological approaches. We conclude that more detailed trajectory analyses are needed to answer the question whether similarity between movement patterns can be considered as a key aspect in the diagnosis and treatment of visual-spatial dysfunctions. Bohil et al. Virtual reality in neuroscience research and therapy. Nat Rev Neurosci. (2011).

Keywords: Memory and Learning

Correspondence address: Mr Dipl.-Biol. David Flentge, University of Bielefeld, Faculty of Psychology and Sports Science, CITEC, Universitätsstraße 21-23, D-33615 Bielefeld, Germany. E-mail: flentge@gmail.com

**P2.50**

**Left ventral premotor cortex subserves matching of hand posture to object use**

Guy Vingerhoets[1], Jo Nys[2], Pieterjan Honoré[2], Elisabeth Vandekerckhove[2] and Pieter Vandemaele[2]
[1]*Department of Experimental Psychology, Ghent University, Ghent, Belgium*
[2]*Ghent University, Ghent, Belgium*

Visuomotor transformations for grasping have been associated with a fronto-parietal network in the monkey brain. Whereas the human homologue of the parietal monkey region (AIP) has been identified as the anterior part of the intraparietal sulcus (aIPS), the putative human equivalent of the monkey frontal region (F5) is less well documented. Results from animal studies suggest that monkey F5 is involved in the selection of appropriate hand postures relative to the constraints of the task. The present study aimed to identify modulation in brain areas sensitive to the difficulty level of tool object – hand posture matching. Seventeen healthy right handed participants underwent fMRI while observing pictures of familiar tool objects followed by pictures of hand postures. The task was to decide whether the hand posture matched the functional use of the previously shown object. Mismatch conditions were manipulated for level of difficulty. Compared to a picture matching control task, the tool object – hand posture matching conditions conjointly showed increased modulation in several left hemispheric regions of the superior and inferior parietal lobules (including aIPS), the middle occipital gyrus, and the inferior temporal gyrus. Comparison of easy versus hard conditions selectively modulated the left inferior frontal gyrus with peak activity located in its opercular part (Brodmann area (BA) 44). The results of the present study support the ventral premotor cortex (vPMC), in particular the opercular region, as the putative human homologue of monkey F5. We suggest that in the human brain, vPMC/BA44 is involved in the matching of hand posture configurations in accordance with visual and functional demands.

Keywords: Other Functions/Disorders, Functional and Structural Neuroimaging/NIRS

Correspondence address: Mr Prof. Dr. Guy Vingerhoets, Ghent University, Department of Experimental Psychology, Henri Dunantlaan 2, B-9000 Ghent, Belgium. E-mail: guy.vingerhoets@ugent.be

**P2.51**

**Verbal emotional memory in a case of bilateral amygdala damage**

Claire Mayor Dubois[1], Sophie Déglise[1], Claudia Poloni[1], Philippe Maeder[2] and Eliane Roulet Perez[1]
[1]*CHUV, Pediatric Department, Lausanne, Switzerland*
[2]*CHUV, Radiology Department, Lausanne (Switzerland)*

The amygdala nuclei appear to be critically implicated in the modulation of emotional memory. Issues regarding the role of valence (i.e., the positive versus negative nature of the stimuli) and of arousal (i.e. the affective intensity of the stimulus) on amygdala emotional processing remain however debated. We studied verbal emotional memory (word list paradigm) in a young woman presenting with bilateral amygdala damage. Results showed specific memory deficits for the emotional stimuli at encoding, and, even when encoding was controlled for, at consolidation. Deficits however only appeared after a one week (but not one hour) delay, showing the crucial involvement of amygdala in long-term consolidation of emotional stimuli. Valence effects were observed, as only the negative (and not positive) words were impaired during encoding whereas consolidation was impaired for both positive and negative stimuli. Finally, the role of the amygdala in processing the intensity (arousal) of the negative stimuli could be evidenced, as only the high-arousing, but not low-arousing, negative words were impaired. Although the negative words were not perceived as emotionally arousing by the patient, these words were specifically poorly encoded and consolidated, suggesting separate cerebral networks involved in emotional processing.

Keywords: Emotion, Amnesia, Neuropsychological Assessment and Psychometrics

Correspondence address: Dr Claire Mayor Dubois, CHUV, Pediatric Department, Rue du Bugnon 46, CH-1011 Lausanne, Switzerland. E-mail: Claire.Mayor@chuv.ch

**P2.52**

**Body postures recognition in TLE: Beyond facial expressions**

Anna Sedda[1], Pina Scarpa[2], Stefano Francione[3], Giorgio Lorusso[3] and Gabriella Bottini[2]
[1]*Departemnt of Brain and Behavioural Sciences, University of Pavia, Pavia, Italy*
[2]*A.O. Niguarda Cà Granda, Cognitive Neuropsychology Centre, Milano, Italy*
[3]*A.O. Niguarda Cà Granda, Epilepsy Surgery Centre "C. Munari", Milano, Italy*

Facial emotion recognition (FER) has been widely investigated in Temporal Lobe Epilepsy (TLE). Studies highlighting prosodic recognition deficits in TLE suggest that emotional impairments might not be modality dependent. However, much less is known – in TLE – about processing of emotions from postures. In this preliminary study, we administered a sample of 10 (4 left and 6 right) drug resistant TLE patients with a task comprising both facial expressions and body postures, that allows to directly compare the two sets of stimuli.

We also recruited a control group of 10 healthy individuals, matched for gender, education and age. All participants have been administered with a facial emotion recognition task (Ekman and Friesen, 1976 Consulting Psychologists Press) and a body postures recognition task – BEAST (de Gelder and Van den Stock, 2011 Frontiers in Psychology). In both tasks, subjects were asked to choose which emotion was displayed among four possible alternatives: fear, anger, sadness and happiness. Disgust is excluded as the BEAST does not contain this posture. Our results show that both right and left TLE patients are impaired respect to controls on fear, anger and happiness recognition, only at the BEAST task (Mann-Whitney U test, all ps $< 0.05$). No significant correlations between age at epilepsy onset and performance at the BEAST task have been found. These results suggest that full displays of facial expressions might not be sensitive enough in TLE (Sedda et al. 2013 CABN). FER seems to be more related to clinical variables such as age at onset and lateralization contrary to body posture recognition which probably explored other aspects rather than basic emotional processes.

Keywords: Emotion, Epilepsy, Neuropsychological Assessment and Psychometrics

Correspondence address: Dr Anna Sedda, Departemnt of Brain and Behavioural Sciences, University of Pavia, piazza Botta 11, IT-27100 Pavia, Italy. Tel.: +39 3490579823; E-mail: anna.sedda@unipv.it

**P2.53**

### Perception of body expression in school-aged children with temporal lobe epilepsy

Laurent Auclair[1], Mélanie Rymer[2], Clémence Lalande[2], Georg Dorfmuller[3], Christine Bulteau[4] and Isabelle Jambaque[5]
[1]*University Paris Descartes, INSERM U663, Institut of Psychology, Boulogne-Billancourt, France*
[2]*University Paris Descartes, Institut of Psychology, Boulogne-Billancourt, France*
[3]*Foundation Ophtalmologique Adolphe de Rothschild, Pediatric Neurosurgery Department, Paris, France*
[4]*Foundation Ophtalmologique Adolphe de Rothschild, Pediatric Neurosurgery Department and INSERM U663, Paris, France*
[5]*University Paris Descartes, Institut of Psychology and INSERM U663, Paris, France*

OBJECTIVE: Children's ability to identify others' emotions is fundamental to emotional regulation and cognitive control. Research on the development of emotion recognition has been dominated by studies on the processing of facial expression; however whole bodily expression is important for other representation and also supports social perception. Children with temporal lobe epilepsy (TLE) frequently have emotional and social disorders including autism spectrum disorders. Here we assess emotional body categorization in children presenting TLE.
METHODS: Nine children with TLE aged from 8 to 15 years old took part of the study. All were involved in presurgical or post operative epilepsy surgery program. Photos of emotional body expression (anger, fear, happiness, sadness and neutral) were displayed one at a time on a computer screen. Children were instructed to categorize the emotion expressed by the whole body stimuli in pointing to the corresponding smiley placed on the right side of the display. Patients' performances were compared to matched healthy children.
RESULTS: Children with TLE identified significantly less emotional body postures than healthy children. Moreover, patient had more difficulties to recognize sadness body postures, followed by neutral, whereas fear was the easiest.
CONCLUSION: These preliminary findings showed that early TLE could also modulate the perception of bodily expressions. Results will be discussed in light of the social perception difficulties observed in children with TLE.

Keywords: Emotion, Epilepsy, Neuropsychological Assessment and Psychometrics

Correspondence address: Dr Laurent AUCLAIR, University Paris Descartes, INSERM U663, Institut of Psychology, Av. Edouard Vaillant, 71, F-92100 Boulogne-Billancourt, France. E-mail: laurent.auclair@parisdescartes.fr

**P2.54**

### Motivational conflict processing in patients with juvenile myoclonic epilepsy: Neurostructural correlates of social decision-making

Martin Peper[1], Markus Ramm[1], Marcus Belke[2], Susanne Knake[2] and Mira-Lynn Chavanon[1]
[1]*Philipps-Universitaet Marburg, Department of Psychology, Experimental and Biological Psychology,*

*Neuropsychology Section, Marburg, Germany*
[2]*Philipps-Universitaet Marburg, Department of Neurology, Marburg, Germany*

Juvenile myoclonic epilepsy (JME) is the most common idiopathic generalized epilepsy and characterized by structural and functional abnormalities of the frontal lobes. It is charecterized by motivational and emotional abnormalities such as rapidly changing affect, unsteadiness, lack of drive and endurance. Approach or withdrawal tendencies as well as motivational conflicts can be studied within the context of Reinforcement Sensitivity Theory (RST). Patients with JME are expected to show elevated reward-related activity (Behavioral Approach System, BAS) and decreased conflict processing (Behavioral Inhibition System, BIS) being associated with poor behavioral inhibition. These changes are expected to be associated with structural brain integrity of the orbitofrontal cortex (OFC) and the anterior cingulate (ACC) as this is the basis of appropriate motivational functioning. Here, we compared 12 patients with JME with 14 matched controls. Motivation was assessed in the context of a cross-modal Pavlovian-Instrumental Transfer (PIT) paradigm inducing a conflict between instrumentally and classically conditioned cues (i.e., "Social Talent Show Task" corresponding to a probabilistic decision-making procedure). Regional brain volumes were assessed with voxel-based MRT morphometry. During conflict, participants with JME showed greater reward but no reduced BIS sensitivity. In JME patients, mOFC white matter volume was associated with lower distraction by conflictual cues ($r = -0.60$; $p = 0.01$; controls: $r = -0.22$). Performance differences during conflict were associated with rostral ACC volume in controls ($r = 0.49$; $p < 0.05$), but not in JME ($r = 0.10$). The results are compatible with the view that the structural integrity of ACC and mOFC is predictive of reward sensitivity and motivational conflict in social decision-making.

Keywords: Emotion, Epilepsy, Functional and Structural Neuroimaging/NIRS

Correspondence address: Mr Prof. Dr. Dr. Martin Peper, Philipps-Universitaet Marburg, Department of Psychology, Experimental and Biological Psychology, Neuropsychology Section, Gutenbergstr. 18, D-35032 Marburg, Germany. Tel.: +49 6421 282 3830; E-mail: peper@uni-marburg.de

### P2.55

### Short term cognitive-behavioral therapy in pseudoepileptic seizures – preliminary results.

Urszula Stolarska-Werynska[1] and Marek Kaciński[2]
[1]*Children's University Hospital Cracow, Pediatric Neurology, Cracow, Poland*
[2]*Children's University Hospital Cracow, Head of the Department of Pediatric Neurology, Chair of Pediatric and Adolescent Neurology, Cracow, Poland*

In the Pediatric Neurology Department we encounter various disorders demanding a differental diagnosis to establish a possible psychogenic cause. Among those the diagnosis of pseudoepileptic seizures is one of the most difficult and often controversial, especially in children with history of antiepileptic pharmacotherapy. Psychotherapy is considered the only effective treatment of psychogenic disorders, while we lack data supporting the effectivenes of one therapeutic schedule over another. Current research argues that psychotherapy of conversion disorders is inevitably a long term process, some of the cases remaining unsuccesfull. We challenge this statement by conducting a study on the effects of cognitive- behavioral therapy in pseudoepileptic seizures.
GOAL OF THE STUDY: To establish whether short term cognitive-behavioral therapy is effective in pseudoepileptic seizures.
METHODS: Short term (8 to 10 sessions, 1 hour each) cognitive behavioral psychoterapy in teenagers with pseudoepileptic seizures. Each patient undergoes video-eeg with placebo trial, and a neuropsychological test battery. The psychotherapeutic sessions include behavioral and cognitive strategies, each session takes 60 minutes, one a day.
MATERIAL: The study has so far included 12 inpatients, aged 15–18, 11 girls, 1 boy Summary of the results: 11/12 patients left the ward without further seizues as observed during the hospitalization, although only a long term follow up may possibly confirm the this result. Two of the children are seizure free for 12 months since finishing the treatment.
CONCLUSION: The preliminary results are very promising, suggesting that an intensive, short term cognitive behavioral therapy may be an effective treatment of psychogenic non epileptic seizures. If proven so in a long term follow up this would be one of the most cost- and time-effective treatment of a disorder that greatly disables not only the patient but his family life, school and total everyday functioning.

Keywords: Emotion, Epilepsy, Neuropsychological Assessment and Psychometrics

Correspondence address: Mrs clinical neuropsychologist Urszula Stolarska-Werynska, Children's University Hospital Cracow, Pediatric Neurology, Wielicka 265, Poland-30-663 Cracow, Poland. Tel.: +48 697608 186; E-mail: ustolarska@interia.pl

**P2.56**

**Influence of the relevant acoustic features on the recognition of emotional prosody following subthalamic nucleus deep brain stimulation in Parkinson's disease**

Julie Péron[1], Sezen Cekic[2], Claire Haegelen[3], Paul Sauleau[3], Dominique Drapier[3], Marc Vérin[3] and Didier Grandjean[2]
[1]*University of Geneva, Psychology and Swiss center for Affective Sciences, Geneva, Switzerland*
[2]*Dprt of Psychology, and Swiss Center for Affective Sciences, Geneva University, CH., Geneva, Switzerland*
[3]*Behavior and Basal Ganglia EA4712 unit, Rennes 1 University and University Hospital of Rennes, F., Rennes, France*

This study focus on the impact of several acoustical features known to be important for the recognition of emotional prosody following subthalamic nucleus (STN) deep brain stimulation (DBS) in Parkinson's disease (PD) and how STN DBS might impact at different levels of emotional auditory processing.
The demonstration of the emotional effects of STN DBS in PD pointed for the first time to the involvement of the STN in the cerebral network subtending emotions and notably the recognition of emotion from voices i.e., emotional prosody (see Péron, Frühholz et al. in press for a review). Nevertheless, the exact contribution of the STN in emotional prosody remains to be clarified, and the question of the influence of acoustic features in the recognition of emotional prosody of STN DBS PD has not been adequately accounted for to date.
We re-analyzed the performances in the recognition of emotional prosody in the PD patients in a pre and in a post-operative conditions and matched healthy controls (Péron, Grandjean et al. 2010) including relevant acoustic features as dependent variables in our statistical modelisation (GLM zero inflated method). We focused the analyses on the significant results that we initially found to be specifically impaired in the post-operative group compared to the pre-operative and control groups.
Results revealed that the biased ratings in the post-operative condition compared to the two other groups were correlated to the maximum of loudness for fear recognition while the biases for anger recognition were correlated to the standard deviation of intensity, the mean, maximum, minimum, and SD of pitch.
There is a significant influence of the relevant acoustic features on the disturbed emotional prosody recognition following STN DBS in PD. Nevertheless, this influence is not sufficient to explain the emotional prosody disturbances observed following STN DBS and suggest that higher levels of emotional prosody processing are also impaired by this surgery.

Keywords: Emotion, Neurodegenerative Diseases, Invasive Techniques

Correspondence address: Dr Julie Péron, university of Geneva, Psychology and Swiss center for Affective Sciences, 40 bd du Pont D'Arve, D-1205 Geneva, Switzerland. E-mail: julie.peron@unige.ch

**P2.57**

**Reduction of neuropsychiatric symptoms in patients with Alzheimer's disease following psychoeducation programs for their caregivers**

Tatiana Aboulafia Brakha[1], Radek Ptak[1] and Deborah Suchecki[2]
[1]*Hôpitaux Universitaires de Genève (HUG), Division of Neurorehabilitation- Department of Clinical Neurosciences, Geneva, Switzerland*
[2]*Department of Psychobiology, Universidade Federal de Sao Paulo, Sao Paulo, Brazil*

BACKGROUND: Neuropsychiatric symptoms such as aggressiveness, hallucination and delusion are commonly found in patients in the moderate stage of Alzheimer's disease (AD). These symptoms are frequently treated with neuroleptics and anxiolytics but patients may suffer from many unpleasant side effects. As an alternative, interventions for caregivers of AD patients have been shown to be useful in the management of neuropsychiatric symptoms.
OBJECTIVES: To assess changes in neuropsychiatric symptoms of patients with moderate AD after two types of psychoeducation interventions for their primary caregivers.
METHODS: Twenty-four primary caregivers (relatives) were assigned to two types of programs that consisted of once-a-week group sessions during eight weeks: Group 1: psychoeducation only ($n = 13$), Group 2: psychoeducation with cognitive-behavioural therapy ($n = 11$). Before and after the intervention, caregivers completed a semi-structured interview (Behave-AD) assessing neuropsychiatric symptoms of the AD patient.
RESULTS: A $2 \times 2$ ANOVA for Behave-AD scores revealed a significant effect of time (F (1,23) = 14.09, $p < 0.01$, etasq = 0.39, $P = 94\%$), but no significant interaction between time and intervention. Indeed, in both groups, neuropsychiatric symptoms significantly decreased after intervention (Psychoeducation only: F (1,12) = 12.96, $p > 0.001$; Psychoeducation + CBT: F (1,10) = 5.36, $p = 0.04$).
CONCLUSION: Psychoeducation for caregivers, regardless of modality, may be useful to promote reduction of neuropsychiatric symptoms in patients with moderate AD. This may be explained by an increased understanding of the disease by caregivers, probably leading to better interaction between them and the AD patient, with more understanding and affect. These findings have significant implications for the treatment of neuropsychiatric symptoms in dementia, since it may reduce the need for administering psychotropic medication.

Keywords: Emotion, Neurodegenerative Diseases

Correspondence address: Mrs Tatiana Aboulafia Brakha, Hôpitaux Universitaires de Genève (HUG), Division of Neurorehabilitation, Department of Clinical Neurosciences, AV. Beau-Séjour, 26, CH-1207 Geneva, Switzerland. E-mail: tatiana.aboulafia@hcuge.ch

**P2.58**

**Top-down interference and cortical responsiveness in face processing: a TMS-EEG study**

Mattavelli[1], Mario Rosanova[2], Adenauer Casali[2], Cosatanza Papagno[1] and Leonor Romero Lauro[1]
[1]*Department of Psychology, University of Milano-Bicocca, Milan, Italy*
[2]*Department of Biomedical Sciences "L. Sacco", University of Milan, Milan, Italy*

Neuroimaging and electrophysiological studies have shown the involvement of a fronto-temporo-occipital network in face processing, but the functional relation among these areas remains unclear. We used transcranial magnetic stimulation combined with electroencephalography (TMS-EEG) to explore the local and global cortical excitability at rest and during two different face processing behavioural tasks. Single-pulse TMS was delivered (100 ms after face stimulus onset) over the right medial prefrontal cortex (mPFC) during a face identity or a face expression matching task, while continuous EEG was recorded using a 60-channel TMS-compatible amplifier. We examined TMS effects on the occipital face-specific ERPs component and compared TMS-evoked potentials (TEPs) recorded during tasks performance and a passive point fixation. TMS reduced the P1-N1 component recorded at the occipital electrodes. Moreover, performing face tasks significantly modulated TEPs recorded at the occipital and temporal electrodes within the first 30 ms after right mPFC-TMS, with a specific increase of temporal TEPs in the right hemisphere for the facial expression task. Furthermore, in order to test the site-specificity of the reported effects, TMS was applied over the right premotor cortex (PMC) as a control site using the same experimental paradigm. Results showed that TMS over the right PMC did not affect ERPs components in posterior regions during the face tasks and TEPs amplitude did not change between task and no task condition, either at fronto-central electrodes near the stimulation or at temporal and occipital electrodes. These findings support the notion that the frontal cortex exerts a very early influence over the occipital cortex during face processing tasks and that excitability across right fronto-temporal cortical regions is significantly modulated during explicit facial expression processing.

Keywords: Emotion, Other Disorder/Samples, TMS/TCDS

Correspondence address: Mrs Mattavelli, University of Milano-Bicocca, Department of Psychology, P.za Ateneo Nuovo 1, IT-20126 Milan, Italy. E-mail: g.mattavelli2@campus.unimib.it

### P2.59

### rTMS stimulation improves the facial mimicry and detection responses in an empathic emotional task

Michela Balconi and Ylenia Canavesio
*Department of Psychology, Catholic University of Milan, Milan, Germany*

Facial expression detection and facial mimicry behavior in response to an empathic situation were analyzed in the present research. We supposed a "simulation mechanism" may be related to empathic response, and that it could be supported by prefrontal cortical structures. High frequency rTMS (repeated Transcranial Magnetic Stimulation) was applied to MPFC to induce an increased response to facial expression of emotions when subjects ($N = 16$) were required to empathize with the emotional stimuli. The stimulus valence was also varied (negative vs positive vs. neutral faces) to explore also the emotional content effect on empathic behavior. Autonomic (facial zygomatic and corrugator EMG subjective response) and detection (correct recognitions, CRs; RTs, response times) measures were found to be modulated by MPFC activity. Specifically, when prefrontal structures were activated (in comparison with sham effect and control site stimulation) an increased performance was observed in terms of increased CRs and reduced RTs for face recognition from one hand; of increased emotion-specific EMG response for the other hand. In fact, zygomatic muscle was more responsive in case of positive emotion (happiness), whereas corrugator activity was related to negative emotions (fear, anger, disgust). A higher effect was revealed for negative, and potentially aversive, faces in comparison respectively with positive and neutral faces. Finally, a direct correlation was found between the autonomic and detection measures. Taken together, these results suggest a "simulation mechanism" underlying empathic situations that includes both EMG and behavioral responses. This mechanism appears to be supported and regulated by MPFC area.

Keywords: Emotion, Other Disorder/Samples, TMS/ TCDS

Correspondence address: Mrs prof michela balconi, Department of Psychology, Catholic University of Milan, largo gemelli 1, D-20123 milan, Germany. E-mail: michela.balconi@unicatt.it

### P2.60

### Conscious and unconscious face recognition is improved by high-frequency rTMS on pre-motor cortex

Balconi[1] and Adriana Bortolotti[2]
[1]*Catholic university of milan, Milan, Germany*
[2]*Department of psychology, Catholic university of milan, Milan*

Simulation process and mirroring mechanism appear to be necessary to the recognition of emotional facial expressions. Prefrontal areas were found to support this simulation mechanism. The present research analyzed the role of premotor area in processing emotional faces with different valence (positive vs. negative faces), considering both conscious and unconscious pathways. High-frequency rTMS (10 Hz) stimulation was applied to prefrontal area to induce an activation response when overt (conscious) and covert (unconscious) processing was implicated. Twenty-two subjects were asked to detect emotion/no emotion (anger, fear, happiness, neutral). Error rates (ERs) and response times (RTs) were considered in response to the experimental conditions. ERs and RTs decreased in case of premotor brain activation, specifically in response to fear, for both conscious and unconscious condition. The present results highlight the role of the premotor system for facial expression processing, supporting the existence of two analogous mechanisms for conscious and unconscious condition.

Keywords: Emotion, Other Disorder/Samples, TMS/ TCDS

Correspondence address: Mrs balconi, catholic university of milan, largo gemelli 1, D-20123 milan, Germany. E-mail: michela.balconi@unicatt.it

### P2.61

### Obesity modulates the relationship between COMT Val158Met gene and harm avoidance

Isabel Garcia-Garcia[1], Pilar González-Tartiere[2], Idoia Marqués-Iturria[2], Roser Pueyo[2], María Ángeles Jurado[2], Bàrbara Segura[2], Imma Hernán[3], María Consuelo Sánchez-Garre[3] and Maite Garolera[3]
[1]*University of Barcelona, Psychiatry and Clinical Psychobiology, Barcelona, Spain*

[2]*University of Barcelona*
[3]*Consorci Sanitari de Terrassa*

Obesity is a health problem in modern societies which can be best summarized as the interaction of genetic susceptibility and environmental factors. Dopamine has been widely implicated in its aethiology. The Val158Met polymorphism of the COMT gene is a source of individual variations in dopamine availability at the prefrontal cortex. Both obesity and the Val158Met polymorphism seem to have an impact on personality measures, although their possible interaction has yet to be examined. We examined two dimensions of the Temperament and Character Inventory Revised (TCI-R), novelty seeking and harm avoidance, in 67 participants with obesity (12–39 years, 60% females) and 53 lean subjects (12–40 years, 64% females). We also obtained COMT genotype from all the participants. Data was analyzed with a 2 (obese versus lean) $\times$ 2 (Val+ versus Val-) analysis of variance. In comparison with controls, participants with obesity scored higher on harm avoidance ($p = 0.047$). We also found an effect of COMT in novelty seeking, with Val+ subjects obtaining higher scores ($p = 0.022$). Furthermore, there was a 'group' x 'COMT' interaction on harm avoidance ($p = 0.028$). Examination of this interaction revealed that obesity altered the normative directionality of the effect of COMT genotype on harm avoidance. This is the first study on the relationship between obesity, the COMT gene and personality traits. The results suggest that obesity is a modulatory factor for effect of COMT genotype on a temperament dimension with a fundamental role in emotional equilibrium.

Keywords: Emotion, Other Disorder/Samples, Neuropsychological Assessment and Psychometrics

Correspondence address: Mrs Isabel Garcia-Garcia, University of Barcelona, Psychiatry and Clinical Psychobiology, Passeig Vall d\, D-08035 Barcelona, Spain. E-mail: isabel.garciagarcia@ub.edu

**P2.62**

**A brief intervention for facial emotion recognition in schizophrenia**

Stella Tsotsi[1], Vassilios P. Bozikas[2], Konstantinos Fokas[2] and Mary H. Kosmidis[1]
[1]*Aristotle University of Thessaloniki, Psychology, Thessaloniki, Greece*
[2]*Aristotle University of Thessaloniki, 1st University Psychiatry Clinic, Thessaloniki, Greece*

Deficits in facial emotion recognition (FER) in schizophrenia are rather ubiquitous. While some studies have suggested that these deficits reflect impaired affective processing, there is increasing evidence of the contribution of attention impairment in poor FER in these patients. Our main objective was to explore this discrepancy employing interventions for either affect recognition or attention to crucial facial features, such as the eyes and mouth. Thirty nine outpatients with schizophrenia (DSM-IV-based diagnosis; 19 men) were randomly assigned to one of three groups: 1) facial emotion intervention (EI), 2) attention to facial features intervention (AI), 3) no intervention (NI). We assessed attention to faces (AF) through a series of stimuli we developed requiring the detection of details in facial features; similarly, we assessed FER with an additional set of newly developed stimuli consisting of photographs of faces with emotional expressions. All patients were assessed for AF and FER pre- and post-intervention (with alternative sets of stimuli). Although pre-intervention AF performance predicted FER accuracy, the AI did not result in improved FER performance at post-intervention. Repeated-measures ANOVAs (with time of assessment as the within-subject factor) showed that only the EI group improved significantly with respect to FER accuracy, particularly on the perception of anger and fear; the AI group improved only on AF accuracy. We found no improvement in affect processing (FER) speed in any group. These findings highlight the prominence of affect processing dysfunction in FER impairment in schizophrenia. Despite the predictive value of attention to facial features in FER accuracy, attention impairment in patients with schizophrenia more likely aggravates, rather than explains poor FER. In conclusion, assessment of facial emotion recognition in patients with schizophrenia may prove a useful addition to any comprehensive remediation program.

Keywords: Emotion, Psychopathology, Neuropsychological Assessment and Psychometrics

Correspondence address: Mrs Stella Tsotsi, Aristotle University of Thessaloniki, Psychology, P.O.Box D3030, 57001 Thessaloniki, Greece. Tel.: +30 694654 6922; E-mail: sttsotsi@yahoo.gr

### P2.63

### A differential effect of a brief intervention on facial affect recognition in patients with first psychotic episode

Vassilios P. Bozikas[1], Stella Tsotsi[2], Aikaterini Dardagani[2], Athanasios Lagoudis[1], Eleni Nazlidou[1], Aikaterini Giazkoulidou[1] and Konstantinos Fokas[1]
[1]*Aristotle University of Thessaloniki, 1st Psychiatry Clinic, Thessaloniki, Greece*
[2]*Aristotle University of Thessaloniki, Psychology, Thessaloniki, Greece*

Facial emotion recognition (FER) is deficient in schizophrenia (SCH) across all stages, including the first episode. Although the importance of FER in SCH has been emphasized by its mediation of the relationship between cognition and social functioning, up-to-date no attempt to improve these deficits in patients with first psychotic episode (FP) has been reported. In this context, our main goal was to investigate the effect of a brief facial emotion intervention in FP. The sample consisted of 20 FP (9 men) and 12 healthy controls (4 men; HC), matched for age and education. The Positive and Negative Symptom Scale (PANSS) was administered for symptom severity. FER was assessed twice, before and after the intervention, via two alternative sets of newly developed stimuli depicting expressions of the six basic emotions. Two scores were obtained: accuracy and reaction time. FER intervention comprised training to recognize changes in facial features, characteristic of certain emotion expressions. No significant correlation was noted between symptom dimensions and FER scores. As evidenced by repeated-measures ANOVA with time as the within-subject factor, both groups showed improved FER at post-intervention, particularly for angry and disgusted facial expressions. In sad faces only a group effect was observed with HC performing better that FP. For fear, an interaction of group and time emerged; while HC performance improved significantly, FP exhibited stable performance between measurements. No improvement was observed in response speed. Overall, these findings suggest that FP can benefit from brief interventions for FER, especially in anger and disgust, in a similar manner to HC. Yet, persistence of impairment in the recognition of fear by the FP is in line with previous studies, suggesting that poor recognition and manipulation of threat-related information may comprise a susceptibility factor for developing the full range of schizophrenia symptoms.

Keywords: Emotion, Psychopathology, Neuropsychological Rehabilitation

Correspondence address: Mrs Stella Tsotsi, Aristotle University of Thessaloniki, Psychology, P.O.Box D3030, 57001 Thermi, Greece. Tel.: +30 6946546922; E-mail: sttsotsi@yahoo.gr

### P2.64

### Changes in perceived anger and psychophysiological response in TBI patients following different anger regulation strategies: implications for psychotherapeutic treatment.

Tatiana Aboulafia Brakha and Radek Ptak
*Hôpitaux Universitaires de Genève (HUG), Division of Neurorehabilitation, Department of Clinical Neurosciences, Geneva, Switzerland*

BACKGROUND: Excessive irritability and anger are frequently reported following traumatic brain injury (TBI). From a cognitive-behavioural perspective, an emotion can be understood in relation to its context, associated thoughts, behaviour and psychophysiological reactions. A few experimental studies with healthy participants have shown that different cognitive strategies for anger regulation interfere with psychophysiological reactions and the perception of angry emotion. Whether similar mechanisms affect pathological anger following TBI is unknown. Objectives: To verify experimentally whether angry feelings and associated psychophysiological reactions following TBI may be modulated by alternative ways of thinking about an anger-inducing event.
METHODS: Nine TBI patients and eight healthy controls participated in an anger recall task and were submitted to four experimental recall conditions: neutral, spontaneous, rumination and reappraisal. Electrodermal activity (EDA) was recorded during each condition, after which, participants answered a self-report questionnaire on subjectively perceived anger (STAXI-State).
RESULTS: One-way ANOVAs revealed a significant effect of condition in both groups, for both EDA (controls: $F(3,21) = 4.42$, $p = 0.015$, TBI: $F(3,24) = 5.124$, $p < 0.01$) and STAXI-state (controls: $F(3,21) = 11.06$, $p < 0.01$, TBI: $F(3,24) = 3.55$, $p = 0.03$). Paired-contrasts showed that EDA was similarly modulated by recall conditions in both groups, but STAXI-State scores were more differentiated in controls than in TBI patients.

CONCLUSION: Physiological correlates of angry feelings may be influenced by the way one thinks about the situation, in both TBI patients and controls, while TBI patients are less able to evaluate their subjective state of angry arousal. These findings suggest that TBI patients may potentially benefit from cognitive-behavioural strategies to improve their anger management, such as the cognitive reappraisal of the anger-inducing situation.

Keywords: Emotion, Traumatic Brain Injuries

Correspondence address: Mrs Tatiana Aboulafia Brakha, Hôpitaux Universitaires de Genève (HUG), Division of Neurorehabilitation- Department of Clinical Neurosciences, AV. Beau-Séjour, 26, CH-1206 Geneva, Switzerland. E-mail: tatiana.aboulafia@hcuge.ch

**P2.65**

**Emotional and uncontrolled eating are associated with lower basal ganglia volumes in obese people**

P. González-Tartière[1], I. Marqués-Iturria[2], I. García-García[2], M. Garolera[3], R. Pueyo[2], B. Segura[2], M.J. Sender[3], M. Vernet[3] and M.A. Jurado[2]
[1]*Department of Psychiatry and Clinical Psychobiology, University of Barcelona, Barcelona, Spain*
[2]*University of Barcelona*
[3]*Consorci Sanitari de Terrassa*

It is well established that basal ganglia mediate goal directed behaviors (Haber and Knutson, 2010), and are known as key brain regions for food motivation and reward (Lawrence et al., 2012). The aim of this study was to assess the relations between eating behavior and basal ganglia volumes in obesity. The sample was composed of 65 participants between 12 and 40 years of age, 35 of which were obese (11 males, 24 females; 24.97 ± 10.95 years of age; BMI) and 30 controls (13 males, 17 females; 26.37 ± 9.24 years of age).
Eating behaviors were determined using the Three Factor Eating Questionnaire (TFEQ), composed of three subscales: cognitive restraint, uncontrolled eating and emotional eating. Basal ganglia volumes were estimated using the automated procedure proposed by Freesurfer for subcortical structures, and then each volume was divided by its corresponding intracranial volume. We performed group comparisons using Student's t and Mann-Whitney's U, and correlations using Pearson's and Spearman's tests. There were no group differences in demographic variables. Compared with control participants, obese subjects showed higher scores on the three subscales of the TFEQ (all $p < 0.001$). Basal ganglia volumes did not differ between groups. Uncontrolled eating was negatively correlated with right nucleus accumbens volume ($r = -0.358$; $p = 0.035$), and emotional eating was negatively correlated with left ($r = -0.336; p = 0.049$), right putamen ($r = -0.374, p = 0.027$) and right nucleus accumbens ($r = -0.368; p = 0.030$) volumes in the obese group. These relations were not found in the lean group. Results show that higher scores in emotional and uncontrolled eating were related to lower basal ganglia volumes in obese people. Our results suggest the important role of the basal ganglia on eating behavior in obesity.

Keywords: Other Functions/Disorders, Other Disorder/Samples, Functional and Structural Neuroimaging/NIRS

Correspondence address: Mrs P. González-Tartière, Department of Psychiatry and Clinical Psychobiology, University of Barcelona, Passeig Vall d'Hebron, 171, D-08035 Barcelona, Spain. Tel.: +34 687817299; E-mail: pilartartiere@gmail.com

**P2.66**

**Fatigue in Multiple Sclerosis patients – a result of reduced histamine modulation in the brain?**

Katrin Hanken[1], Anja Gossmann[2], Pia Lehmann[2], Helmut Hildebrandt[2] and Andreas Kastrup[2]
[1]*Department of Neuropsychology, University of Bremen, Bremen, Germany*
[2]*Klinikum Bremen-Ost, Neurology, Bremen, Germany*

Although fatigue is one of the most common and disabling symptoms of multiple sclerosis (MS), little is known about its pathophysiology. The aim of this study was to investigate if the posterior hypothalamus (considered as the wake center) is involved in the underlying mechanisms of MS-related fatigue. The study evaluated 49 patients with relapsing-remitting MS and 14 healthy controls using diffusion tensor imaging (DTI) to obtain information about fiber integrity. DTI parameters were calculated for fibers between the posterior (as well as anterior) hypothalamus and the prefrontal cortex, the mesencephalon and the pons. Fatigue severity was assessed using the fatigue scale for motor and cognitive functions. The level of alertness was investigated using a testbattery for alertness and the Beck's Depression Inventory (BDI) was used to determine a clinical relevant depression. In total 87.8% of patients suffered from cognitive fatigue, 89.8% reported motor fatigue and 63.3% had an impaired alertness level. Multiple analysis of variance showed significant differences in DTI parameters between groups, with fatigued patients revealing lower axial and radial diffusivity values for fibers between the right posterior hypothalamus and the mesencephalon than non-fatigued patients and healthy controls (cognitive fatigue: $p = 0.047$; motor fatigue: $p = 0.026$). Considering alertness, patients with normal alertness showed smallest values for axial and radial diffusivity regarding the same fibers ($p = 0.048$), but there was no difference between impaired MS patients and healthy controls. DTI parameters for fibers of the anterior hypothalamus revealed significant associations with the BDI (defined as covariate). These findings suggest that reduced fiber integrity between the right posterior hypothalamus and the mesencephalon might be involved in the pathophysiology of MS-related fatigue. Also, there might be relationship between the anterior hypothalamus and the level of depression.

Keywords: Other Functions/Disorders, MS/Demyelination, Other Methods

Correspondence address: Mrs Katrin Hanken, Department of Neuropsychology, University of Bremen, Hochschulring, D-28359 Bremen, Germany. E-mail: k.hanken@gmx.de

**P3.01**

**Second language proficiency induces white matter changes in the left parietal lobe in aging populations**

Matteo Canini[1], Pasquale Della Rosa[2], Lo Ping Sheung[3], S.Brendan Weekes[3] and Jubin Abutalebi[3]
[1]University San Raffaele, Center for Cognitive Neuroscience, Milan, Italy
[2]IBFM-CNR, Institute of Molecular Bioimaging and Physiology, Milan, Italy
[3]Department of Speech and Hearing Sciences, University of Hong Kong, Hong Kong, China

There's a growing body of evidence demonstrating that mastering a second language (L2) exerts positive effects on brain structure and brain integrity. It was also reported that these plastic changes are not strictly related to language processing but generalize to other cognitive functions such as executive control (Abutalebi et al., 2012).
Structural grey matter plasticity associated to multilingualism has been highlighted already in young children in a brain area not only involved in language processing but also recruited for attentional demands, namely the left inferior parietal cortex (LIPL) (Della Rosa et al., 2013). Interestingly, grey matter changes in this area have been also related to the degree of proficiency of second language in a group of early adult bilinguals (Mechelli et al., 2004) .
In the present study we investigated by means of structural neuroimaging (MR imaging) a group of 34 aging bilingual subjects from Hong Kong. In detail, we explored with the VBM (voxel based morphometry) technique the relationship between whole-brain white matter density and language proficiency. We found that values in fiber tracts connecting LIPL correlated to the relative language proficiency of the aging population. This result shows that fiber tracts connecting LIPL to both temporal and frontal language areas (i.e. the arcuate fasciculus) are influenced by second language expertise and confirm the role of LIPL as a crucial area for multilingualism and its interface with other cognitive functions.

Keywords: Language, Aging, Functional and Structural Neuroimaging/NIRS

Correspondence address: Mr BS Matteo Canini, University San Raffaele, Center for Cognitive Neuroscience, Via Olgettina 59, IT-20100 Milan, Italy. E-mail: universiteo@gmail.com

**P3.02**

**Second language naming performance predicts temporal pole integrity in aging bilinguals**

Jubin Abutalebi[1], Matteo Canini[2], Pasquale Della Rosa[3], Lo Ping Sheung[4], David W. Green[5] and Brendan S. Weekes[4]
[1]*University of Hong Kong, Division of Speech and Hearing Sciences, Hong Kong, China*
[2]*University San Raffaele Milan, Centre for Cognitive Neuroscience, Milan, Italy*
[3]*IBFM-CNR National Research Council, Milan, Italy*
[4]*The University of Hong Kong, Division of Speech and*

Hearing Sciences, Hong Kong, Hong Kong
[5]University College London, Cognitive, Perceptual and Brain Sciences, London, UK

There is now abundant evidence showing that the left temporal pole (TP) is involved in language processing, and especially in naming. The left TP is believed to function as a hub for linking modality-specific conceptual properties of objects (Jefferies and Lambon Ralph, 2006) and to be involved in lexical retrieval (Tranel, 2009). Interestingly, both TP structure (in terms of grey matter density) and naming abilities decrease in aging populations.
The purpose of the present study was to investigate in a group of aging bilingual subjects whether the grey matter density of the TP is preserved and whether this is due because of their language proficiencies in L1 (first language) and L2 (second language).
36 aging bilinguals from Hong Kong (Cantonese-English) and (Cantonese-Mandarin) bilinguals participated in this study and underwent structural neuroimaging. Behavioral testing was conducted in order to investigate their performance on picture naming in L1 and L2. Subjects were also assessed on the MMSE and the SES (Socio-Economical Status) was determined. Grey matter density was analyzed with the VBM (Voxel based Morphometry) technique.
Grey matter (GM) density in the left TP, as expected, negatively correlated with the age of the subjects. However, there was a positive correlation between picture naming performance overall and GM density in the left TP. Interestingly, this correlation was significant only for L2 naming and not L1 naming. Noteworthy, MMSE and SES scores did not correlate with the TP grey matter.
In conclusion, our findings suggest that in bilinguals the structural integrity of the left TP during aging may be protected by better second language naming performances.

Keywords: Language, Aging, Functional and Structural Neuroimaging/NIRS

Correspondence address: Mr Prof Jubin Abutalebi, University of Hong Kong, Division of Speech and Hearing Sciences, Pok Fulam, HK-00000 Hong Kong, China. E-mail: abutalebi.jubin@hsr.it

### P3.03

### Effects of Transcranial Direct Current Stimulation (tDCS) on language and cognitive functions in patients with Alzheimer's disease: A pilot study

Cristina Fonte[1], Marialuisa Gandolfi[1], Giorgia Corradini[2], Luca Ambroso[2], Elisabetta Verzini[2], Valentina Varalta[1], Carlo Miniussi[3] and Nicola Smania[4]
[1]*University of Verona, Department of Neurological, Neuropsychological, Morphological and Movement Sciences, Verona, Italy*
[2]*University of Verona, Italy*
[3]*IRCCS Fatebenefratelli, Brescia, Italy*
[4]*Department of Neurological, Univerity of Verona, Neuropsychological, Morphological and Movement Sciences, Verona, Italy*

Alzheimer's disease (AD) affects from 2 to 5.8% of subjects over the 65 years old and the prevalence increases with age to reach 15–20% in subjects over 80 years old. To date, several techniques have been used in order to attempt to slow down Alzheimer's progress, and stimulate the residual cognitive function.
The aim of this study is to investigate the impact of both anodal tDCS and sham tDCS on language and cognitive functions in AD patients after brain stimulation on the left dorsolateral prefrontal cortex. The effects of tDCS on speech's disorder, specifically naming and comprehension of objects and actions, have been evaluated for the first time in this project, since they have never been assessed for this aim. Also, the effects of tDCS stimulation on the remaining cognitive functions such as attention, short-term memory, working memory, recognition memory and verbal fluency have been investigated.
Each subject received both tDCS sessions, anodal and sham, and performed a cognitive assessment pre-stimulation, an assessment during stimulation, and one at 48 hours after the end of stimulation (follow-up).
The patients were evaluated with two BADA's subtest (comprehension and naming), backward and forward Digit Span, Rivermead Behavioral Memory Test, Attentional Matrix Test and Phonemic Verbal Fluency.
The preliminary analysis showed different results in the first three subjects; in particular the real tDCS technique has proven to be more effective in improving language functions in subjects with moderate AD than in patients with mild AD.
This study could have a great importance in the clinical setting since it would offer tools to slow the cognitive impairment's progression in Alzheimer's disease with a positive effect on maintaining autonomy in daily life.

Keywords: Language, Aging, TMS/TCDS

Correspondence address: Mrs Cristina Fonte, Department of Neurological, University of Verona, Neuropsychological, Morphological and Movement Sciences, piazzale L.A. Scuro 10, I-37134 Verona, Italy. E-mail: cristina.fonte@univr.it

### P3.04

### Effects of grammatical class on confrontation naming in Chinese speakers with dementia

Brendan Weekes[1], Diana Ho[2], Anthony Kong[3], LW Chu[4] and Joyce Ha[4]
[1]*University of Hong Kong, Laboratory for Communication Sciences, Hong Kong, China*
[2]*University of Hong Kong, Laboratory for Communication Science, Hong Kong, China*
[3]*Department of Communication Disorders, University of Central Florida, Orlando, US*
[4]*University of Hong Kong, Medicine, Hong Kong, China*

Selective impairments to action and object naming are often observed in patients who have dementia of the Alzheimer type (DAT). Typically, action naming is more impaired and declines earlier than object naming although the reverse dissociation is also reported in a handful of case studies. The dissociation between action and object naming in aphasia suggests that verbs and nouns are independent forms in the lexicon although this is not a universally held view. These patterns of dissociation have been reported in Chinese speaking patients following stroke. However, no study has tested the hypothesis that action and object naming might be selectively impaired in Chinese speakers who have dementia. We tested this hypothesis with 50 native Cantonese speakers who have DAT according to scores on Chinese versions of the MMSE, MOCA and locally developed tests in Hong Kong. Patients ranged in age from 58 to 90 years. Males and females participated. Some patients were bilingual having learned English or Mandarin as a second language. All patients were presented with action and object pictures for naming in Cantonese. Pictures were selected according to several criteria. First, all items were familiar to native Chinese speakers. Second, all action pictures were verbs without a nominal component and object pictures were nouns without a verbal component. Finally, items were matched for critical psycholinguistic variables. The results revealed a greater impairment in action naming than object naming i.e. there was an effect of grammatical class on confrontation naming with more impairment to spoken word production of verbs (actions) than nouns (objects) similar to reports of other patients with DAT who speak languages in different linguistic families. Taken together with other evidence of neural specificity for verbs and nouns in brain imaging studies (fMRI) of Chinese speakers, our conclusion is that grammatical class is a universal organising principle of the language system.

Keywords: Language, Dementia, Other Methods

Correspondence address: Professor Brendan Weekes, University of Hong Kong, Laboratory for Communication Sciences, University of Hong Kong, D-1111 Hong Kong, China. E-mail: Weekes@hku.hk

### P3.05

### Reading problems in children with Frontal Lobe Epilepsy

Emilio Verche[1], Mariana Cairós[1], Ruth Marrero-Abrante[2] and Sergio Hernández[1]
[1]*University of La Laguna, Psychobiology and Methodology, La Laguna, Spain*
[2]*Hospital Universitario de Canarias, Neurology, La Laguna, Spain*

INTRODUCTION: Children with epilepsy show academic difficulties, especially in arithmetic and reading. Antiepileptic drugs side effects could explain these deficits. However, we suggest a reading deficit directly related with the epilepsy, where the frontal focus could interfere in this basic instrumental skill.
OBJECTIVE: To study reading performance (speed, accuracy and comprehension) in children with frontal lobe epilepsy (FLE).
METHODS: 13 children with FLE and 13 healthy controls (aged 10–19) participated. They all completed 3 subtests from "Batería de Evaluación de Procesos Lectores": word reading task, pseudoword reading task and texts comprehension task. Trail Making Test-A was used as a covariable. In the reading tasks, the number of words read correctly and the reading time was measured as well as an accuracy index was calculated.

In the comprehension task, the number of correct answers to some questions about the text was registered.
RESULTS: We found significant differences in all the variables, except in number of pseudowords correct read, with a worse performance in FLE group and with no effect of the processing speed.
CONCLUSIONS: Results suggest reading problems in children with FLE in both lexical and semantic level, independently of the processing speed. This highlights the need for early intervention in reading skills to prevent these deficits and school failure.

Keywords: Language, Epilepsy, Neuropsychological Assessment and Psychometrics

Correspondence address: Mr Emilio Verche, University of La Laguna, Psychobiology and Methodology, Campus de Guajara s/n, Spain-38071 La Laguna, Spain. Tel.: +34 616865574; E-mail: evercheb@ull.edu.es

**P3.06**

**The cumulative semantic interference effect in normal and pathological ageing**

Claudio Mulatti[1], Clara Calia[2], Maria Fara De Caro[3] and Sergio Della Sala[4]
[1]*Università di Padova, Dipartimento di Psicologia dello Sviluppo e della Socializzazione, Padova, Italy*
[2]*Queen Margaret University, Edinburgh, Scotland*
[3]*Università di Bari, Italy*
[4]*University of Edinburgh, Scotland*

BACKGROUND: People affected by Alzheimer Dementia (AD) or Mild Cognitive Impairment (MCI) present with impairments in lexical/semantic processing. Picture naming could be seen as a lexical/semantic task which rests on the activation of perceptual, semantic, and phonological representations. The poor performance of MCI/AD individuals in picture naming has been accounted for in terms of deficits of either the perceptual, semantic, or phonological stages (e.g., Faust, Balota, and Multhaup, 2004)
AIM: To disentangle the source of this deficit we compared the cumulative semantic interference effect (CSIE; Howard, Nickels, Coltheart, and Cole-Virtue, 2006) and the repetition priming effect (RPE) obtained with a sample of 20 healthy elderly to that obtained with a sample of 20 MCI/AD elderly.
METHOD: The RPE refers to an increase in performance for repeated items compared to unrepeated items. The CSIE defines a linear increase in the naming RTs which is function of the already named pictures belonging to the same semantic category to which the named picture belongs.
RESULTS: CSIE was present in the healthy sample and absent in the MCI/AD sample. Instead, both groups showed comparable repetition priming effects.
CONCLUSION: The impairment in picture naming exhibited by MCI/AD individuals is due to an inefficient semantic access.

References

[1] Faust, M. E., Balota, D. A., & Multhaup, K. S. (2004). Neuropsychology, 18, 526-536.
[2] Howard, D., Nickels, L., Coltheart, M., & Cole-Virtue, J. (2006). Cognition, 100, 464–482.

Keywords: Language, Mild Cognitive Impairment, Other Methods

Correspondence address: Mr Claudio Mulatti, Università di Padova, Dipartimento di Psicologia dello Sviluppo e della Socializzazione, via Venezia 8, I-35131 Padova, Italy. E-mail: claudio.mulatti@gmail.com

**P3.07**

**Dual task effect on mirror writing**

Natascia De Lucia[1], Rob Mcintosh[2] and Sergio Della Sala[2]
[1]*Second University of Naples, Psychology, Neuropsychology Laboratory, Caserta, Italy*
[2]*University of Edinburgh, Human Cognitive Neuroscience, department of Psychology, Edinburgh, UK*

INTRODUCTION: Mirror writing (MW) occurs when the orientation and position of single letters or whole words are reversed [1]. MW has been often described in young children or brain damaged patients. Only a few studies have focused on normal healthy individuals. It has been suggested that left-handers are better able to reverse writing direction than right-handers [2]. We aimed to test whether left-handers were advantaged for MW compared to right-handers, and to investigate the influence of a cognitively demanding concurrent task upon MW.
METHODS: We recruited 20 right-handed and 20 left-handed normal adults. Participants were required to write, to dictation, asymmetrical letters in forward or mirrored direction with their preferred and nonpreferred hand, in single-task and dual-task (combined with an auditory 1-back task) conditions. Planning time and error rate were recorded.
RESULTS: Planning times and error rates increased when participants wrote in a mirrored direction, with their non-dominant hand, and in dual task conditions. Error rates were especially high for MW in the dual task condition. No evidence was found to support the view that left-handers are better at MW than right-handers.
CONCLUSION: Our findings suggest that MW ability is unrelated to left-handedness, at least amongst normal adults. MW is a cognitively demanding task for normal adults, as indicated by the increased MW errors in the dual task condition.

References

[1] Della Sala S., Cubelli R. (2007). 'Directional apraxia': A unitary account of mirror writing following brain injury or as found in normal young children. Journal of Neuropsychology, 1: 3-26
[2] Tankle R., Heilman K. (1983). Mirror-writing in right-handers and left-handers. Brain and Language, 19: 115-23

Keywords: Language, Neuropsychological Assessment and Psychometrics

Correspondence address: Mrs NATASCIA DE LUCIA, Second University of Naples, Psychology, Neuropsychology Laboratory, VIALE ELLITTICO, IT-80100 Caserta, Italy. E-mail: natascia.delucia@unina2.it

**P3.08**

### A detailed case of deliberate mirror writing

Rob McIntosh[1], Natascia De Lucia[2] and Sergio Della Sala[1]
[1]*University of Edinburgh – Human Cognitive Neuroscience, department of Psychology, Edinburgh, UK*
[2]*Second University Of Naples, Psychology, Neuropsychology Laboratory, Caserta*

INTRODUCTION: Mirror writing (MW) refers to striking phenomenon of the production of letters or words from right to left [1]. MW is mainly observed in children learning to write and in left brain-damaged patients writing with their left hand [2]. Deliberate MW has also been described in healthy individuals but only anecdotally and a systematic analysis of deliberate MW has never been reported.
METHODS: We described the case of K.B., an ambidextrous German academic who deliberately practiced MW with either hand for more than fifty years. We assessed K.B. with several handwriting tasks in forward and backward direction, vertically upright and inverted, with his right or left hand acting alone and simultaneously. Naïve observers rated the similarity of K.B.'s productions to his standard right hand forward handwriting.
RESULTS: K.B.'s Left-hand MW was rated as more similar to his normal writing (right hand forward) than was right-hand MW, in both upright and inverted writing. Moreover, when writing bimanually, K.B.'s performance is improved when the two hands make mirror-image movement (producing opposite scripts) than if they work in the same direction to produce similar scripts.
CONCLUSION: Our findings are consistent with the hypothesis that MW represents the untransformed left-hand execution of learned right-hand actions.

References

[1] McIntosh R.D., Della Sala S. (2012). Mirror-writing. The Psychologist, 25: 742-6
[2] Della Sala S., Cubelli R. (2007). 'Directional apraxia': A unitary account of mirror writing following brain injury or as found in normal young children. Journal of Neuropsychology, 1: 3-26

Keywords: Language, Neuropsychological Assessment and Psychometrics

Correspondence address: Mr Rob McIntosh, University of Edinburgh, Human Cognitive Neuroscience, department of Psychology, George Square, UK-EH89JZ Edinburgh, UK. E-mail: r.d.mcintosh@ed.ac.uk

**P3.09**

### Specifics of left neglect dyslexia? Word reading errors in neglect patients and healthy controls

Christiane Weinzierl[1], Georg Kerkhoff[2], Lucia Van Eimeren[3], Ingo Keller[4] and Prisca Stenneken[1]
[1]*Bielefeld University, clinical linguistics, Bielefeld, Germany*
[2]*Saarland University, Clinical Neuropsychology Unit, Saarbrücken, Germany*
[3]*Department of Surgery, University of Western Ontario, Ontario, Canada*
[4]*Schön clinic Bad Aibling, Neuropsychology, Bad Aibling, Germany*

OBJECTIVE: Left visuo-spatial neglect due to right hemispheric lesions is often associated with neglect dyslexia (ND), a complex reading disorder. In word reading, ND can affect two aspects of visual word recognition: processing of letter identity – resulting in different error types, – and positional processing of segmental information – resulting in different error positions. Patients with ND often show left substitutions and left omissions of letters. The question remains which reading errors are specific for ND and which are not just a sign of generally reduced reading performance. Since the number of reading errors in healthy controls is usually too low for proper analyses, this question was difficult to address so far.
METHODS: We present a study with quantitative and qualitative analyses of reading errors in single word reading – both in 18 neglect patients and 11 healthy, age-matched controls. The task difficulty between groups was matched via individually adjusted durations of stimulus presentation in order to achieve comparable error rates in both groups.
RESULTS: Results show an equal number of substitutions in both groups but a significantly higher number of omissions in ND than in controls. The spatial distribution of reading errors over the letter positions within the word in ND followed an almost linear increase from the end to the beginning of the word. Results of controls replicate a typical, inverted u-shaped error gradient with most errors in the middle of the word.
CONCLUSION: The present study allows differentiating between error types associated with reduced word reading performance in general and error types that are characteristic of ND and thus highlight the underlying mechanisms in impaired word reading. Omissions and left-sided reading errors clearly are specific for ND. In line with well-established models of word recognition, we assume a sublexical serial word processing deficit in ND.

Keywords: Language, Neglect

Correspondence address: Mrs Christiane Weinzierl, Bielefeld University, clinical linguistics, Universitätsstraße 25, D-33615 Bielefeld, Germany. E-mail: christianeweinzierl@gmx.de

### P3.10

### Neglect Dyslexia: A matter of good looking

Silvia Primativo[1], Lisa S. Arduino[2], Maria De Luca[3], Roberta Daini[4] and Marialuisa Martelli[1]
[1]*University of Rome "La Sapienza" and IRCCS Fondazione Santa Lucia, Department of Psychology, Rome, Italy*
[2]*University LUMSA and ISTC-CNR, Human Sciences, Rome, Italy*
[3]*IRCCS Fondazione Santa Lucia, Neuropsychology unit, Rome, Italy*
[4]*University Milano-Bicocca, Department of Psychology, Milan, Italy*

STUDY GOALS: Right-sided brain-damaged patients with Unilateral Spatial Neglect (USN) often make left-sided errors in reading single words or pseudowords (Neglect Dyslexia, ND). The more common reading errors, omissions and substitutions, have been recently shown to depend from two different mechanisms, the exploratory disorder which characterizes unilateral spatial neglect (USN) and a perceptual integration mechanism, respectively (Martelli et al., 2011). The relation between ND and the more general unilateral spatial neglect (USN) is still controversial (Vallar et a., 2010, for a review). In the present study the proposal is made that both left neglect and low fixation accuracy account for reading errors in Neglect Dyslexia.
METHOD: Eye movements were recorded in USN patients with (ND+) and without (ND-) neglect dysl- exia and in a matched control group of right brain-damaged patients without neglect (USN-). Results. Unlike ND- and controls, ND+ patients showed left lateralized omission errors and a distorted eye movement pattern in both a reading aloud task and a non-verbal saccadic task. During reading, the total number of fixations was larger in these patients independent of visual hemispace, and most fixations were inaccurate. Similarly, in the saccadic task only ND+ patients were unable to reach the moving dot. A third experiment addressed the nature of the left lateralization in reading error distribution by simulating neglect dyslexia in ND- patients. ND- and USN- patients had to perform a speeded reading-at-threshold task that did not allow for eye movements. When stimulus exploration was prevented, ND- patients, but not controls, produced a pattern of errors similar to that of ND+ with unlimited exposure time (e.g., left-sided errors).
CONCLUSION: We conclude that the reading disorder in neglect dyslexia is the behavioural expression of the exploratory deficit in USN when the fine eye movements required for reading are altered.

Keywords: Language, Neglect, Neuropsychological Assessment and Psychometrics

Correspondence address: Mrs Ph.D student Silvia Primativo, University of Rome “La Sapienza” and IRCCS Fondazione Santa Lucia, Department of Psychology, Via dei Marsi 1, RM-00185 Rome, Italy. E-mail: lisa.arduino@uniurb.it

**P3.11**

**Spontaneous speech and discourse organization in fronto-temporal degeneration: A single case study**

Federica Lucchelli and Eleonora Martini
*Alzheimer’s Center, Passirana di Rho, Italy*

A deficit in discourse organization has been previously reported as the most striking deficit in cases of dysexecutive fronto-temporal degeneration (FTD) (Ash et al., 2006). We investigated language functions in a lady (AB) affected by FTD with prominent executive impairment. A F18-FDG PET study showed left temporal and less severe fronto-basal hypometabolism. Relatives reported that onset, about one year before, was characterized by “confusion” and inability to follow the thread while speaking. Speech abnormalities were evident on clinical examination and consisted of an abundant verbal production, with unfinished sentences, sudden jumps from one point to another, inability to follow a logical sequence of arguments and to build up a coherent narrative, to the point that verbal communication became exceedingly laborious. General cognitive assessment showed significant executive deficits, with relative sparing of memory. An extensive language assessment, carried out by standardized tests, demonstrated significant naming difficulties on confrontation tests, with spared comprehension and repetition. Spontaneous speech and discourse were sampled by several tasks, including scene description, story recall, life narratives and picture story narration. Analyses of appropriateness of contents, story coherence and completeness were performed; number of words, narrative duration, occurrence of anomic or syntactic errors were also taken into account. The overall severity of impairment varied among different tasks: life narratives turned out to be the most effective procedure to elicit discourse disorganization, followed by picture story narration. These differences may be attributed to differential tasks requirements in terms of executive constraints related to discourse planning and monitoring. Moreover, the association of anomic difficulties (in the absence of semantic impairment) points out to the occurrence of mixed patterns of speech/language disturbances in FTD.

Keywords: Language, Neurodegenerative Diseases, Neuropsychological Assessment and Psychometrics

Correspondence address: Dr Federica Lucchelli, Alzheimer’s Center, via Settembrini 1, I-20017 Passirana di Rho, Italy. E-mail: flucch@libero.it

**P3.12**

**Reorganization of cerebral language-associated areas in a 65-year-old female with anaplastic astrocytoma as evidenced by functional magnetic resonance imaging and intraoperative direct electrocortical stimulation**

Margit Jehna[1], Gord Von Campe[2], Markus Hoffermann[2], Lukas Bruckmann[2], Gernot Reishofer[1], Franz Payer[3], Christian Enzinger[3] and Franz Ebner[1]
[1]*Department of Radiology, Medical University of Graz, Graz, Austria*
[2]*Department of Neurosurgery, Medical University of Graz, Graz, Austria*
[3]*Department of Neurology, Medical University of Graz, Graz, Austria*

This is a case study of a right handed 65-year-old woman with an anaplastic astrocytoma grade III in the superior frontal cortex with intra-axial extensions to the corpus callosum. Presurgical functional MR imaging (fMRI) was done to visualize language network distribution. Intraoperative direct cortical stimulation (DCS) was accordingly done to prevent eloquent functional regions from resection. FMRI results showed a distributed language network including the left inferior frontal gyrus (pars opercularis), the left and right frontal orbital cortices, the left insular cortex, the left superior and middle temporal gyri, the left precentral and postcentral gyri, and the supplementary area. Additionally, statistically stable but unusual activation was found in the left superior frontal gyrus (SFG). During surgery, DCS demonstrated that the stimulation of the SFG – involved in language function according to the fMRI – led to anomic disturbances. During the assessment of Boston Naming Test, the patient could say "This is a...", but the retrieval of words was disrupted. These deficits in word finding were persistently reproducible when stimulating the same area. Afterwards, tumor removal was performed under navigation and fluorescence guidance as well as subcortical electrostimulation, thus preserving eloquent motor and language areas and tracts. Regarding the results of the fMRI and DCS in this patient, it can be speculated that the altered distribution of eloquent language areas could be the result of a tumor-induced reorganization process. This case study demonstrates that presurgical fMRI was able to identify a localized unusual language network distribution, which was validated with direct cortical mapping during awake surgery. It underlines the importance of careful presurgical planning, keeping in mind the limitations of all used methods.

Keywords: Language, Other Disorder/Samples, Functional and Structural Neuroimaging/NIRS

Correspondence address: Mrs PhD Margit Jehna, Medical University of Graz, Department of Radiology, Auenbruggerplatz 9, AUT-8036 Graz, Austria. E-mail: margit.jehna@medunigraz.at

**P3.13**

**Phonology without semantics in immediate serial recall**

Annalisa Benetello, Carlo Cecchetto and Costanza Papagno
*Universita di Milano-Bicocca, Department of Psychology, Milan, Italy*

The influence of long-term knowledge on verbal short-term memory (STM) performance has been demonstrated in various studies. However, long-term knowledge facilitation could be of two different types: STM performance could be enhanced only by familiarity with the phonological form, or by semantic information about the word.
The aim of this study was to verify whether the pattern of verbal STM performance described for a patient suffering from semantic dementia is found also in normal subjects. In particular, we wanted to replicate data from an SD patient, who did not differ in immediate serial recall of known words and words whose phonological form was available, but not the meaning (see Papagno et al., 2012).
Participants to this study were trained on two different lists of 10 nonwords each (participants were told that these words belonged to a foreign language they were unfamiliar with). Participants were trained to learn both the semantics and the phonology of words in list 1 (they were taught the meaning of the word in the foreign language), while they were trained only on phonology for words in list 2. A list of 10 nonwords (list 3) was also included. After the training, participants completed three serial recall tasks, one on each list.
Preliminary results show that performances in serial recall on lists 1 and 2 are comparable, while performance on list 3 is significantly worse, indicating that the phonological representation of a word, even in absence of access to the semantics of the word itself, is enough to give facilitation during a STM task.
This suggests that long-term knowledge facilitates immediate serial recall due to familiarity with the phonological form of the word rather than with its meaning.

Keywords: Language, Other Disorder/Samples

Correspondence address: Mrs Annalisa Benetello, Department of Psychology, Universita di Milano-Bicocca, Piazza Ateneo Nuovo, 1, IT-20126 Milan, Italy. E-mail: a.benetello@campus.unimib.it

**P3.14**

**Effects of the linguistic environment on the right visual field superiority in reading: A study in French-Hebrew bilinguals**

Nathalie Haehnel and Eric Sieroff
*Université Paris Descartes, Boulogne-Billancourt, France*

A word is better identified when it is presented in the right (RVF) than the left visual field (LVF). This RVF superiority (RVFS) might be explained by the role of the left hemisphere (LH) in reading (direct projection from RVF to LH, and/or attentional bias in the LH/RVF in a linguistic task). It might also be explained by the reading direction in left-to-right scripts, favoring the visual field where reading progresses (attentional bias). However, several studies using Hebrew words (right-to-left script) also obtained a RVFS, even though it was usually smaller than with words in left-to-right scripts. It has also been suggested that the linguistic environment and the practice of the language have an effect on RVFS. In this study, we evaluated RVFS with French and Hebrew words in 4 groups of 24 neurologically healthy participants each, all reading French and Hebrew as a first or second language: Native French speakers in France, Native French speakers in Israel, Native Hebrew speakers in Israel, Native Hebrew speakers in France. All groups had equivalent knowledge of the second language. Words were tachistoscopically presented in the RVF or the LVF. They had to be identified by naming the word or by reporting some of its letters. Results of correctly identified letters showed a RVFS for the 4 groups in both languages. Also, RVFS was larger with French (RVFS of 13.8%) than with Hebrew words (9.0%). However, the RVFS was larger with French than Hebrew words only for the Native French speakers in Israel and the Native Hebrew speakers in France. For Native French speakers in France, and for Native Hebrew speakers in Israel, no significant difference of RVFS were found between languages, and the RVFS was larger in France (15.5%) than in Israel (8.6%). These results are in favor of an effect of the linguistic environment on the RVFS. Whatever the exact nature of this effect, it may produce a modulation of attentional biases in reading.

Keywords: Language, Other Disorder/Samples, Other Methods

Correspondence address: Mrs Nathalie HAEHNEL, Université Paris Descartes, 71 avenue Edouard Vaillant, F-92100 Boulogne-Billancourt, France. E-mail: nathalie.haehnel@gmail.com

**P3.15**

**Representation of the incorrect use of an object is supported by the DLPFC. The tDCS effect on ERP N400 for visual and linguistic stimuli**

Balconi[1] and Silvia Vitaloni[2]
[1]*Deprtment of Psychology, Catholic University of Milan, Milan, Italy*
[2]*Department Of Psychology, Catholic University of Milan, Milan*

In the present study, we explored the representation of an incongruent action (instrumentally incorrect use of an object) in comparison with sentences ending with an incongruent action word, taking into account the role of left dorsolateral prefrontal cortex (DLPFC) activation. This activity was appositely modulated by tDCS (transcranial direct current stimulation). The effect of tDCS when subjects processed congruent/incongruent object-related actions (Experiment 1) or sentences (Experiment 2) was verified by measuring changes in the ERP (event-related potential) N400, ERs (Error Rates) and RTs (Response Times). In Experiment 1, thirty subjects performed the detection task within a dynamic context (video tapes representing a sequence of four action frames). In Experiment 2, twenty-eight subjects read sentences that represented object-related actions. The stimulation effect (a cathode applied to the DLPFC and an anode to the right supraorbital region) was analysed by comparing the ER, RT and ERP profiles before and after stimulation (or sham treatment). A significant reduction of the N400 was observed for incongruent stimuli in the case of cathodal (inhibitory) stimulation of the DLPFC compared with pre-stimulation conditions for Experiment 1, but not Experiment 2. Moreover, ERs were increased, and RTs were reduced in response to incongruent conditions after tDCS, but not after sham stimulation in Experiment 1. It was suggested that perturbation of the DLPFC may limit the ability to analyse a semantically anomalous action sequence, with a reduced N400 ERP effect and increased random responses being observed. Finally, the contribution of the frontal area to the semantic processing of action was discussed.

Keywords: Language, Other Disorder/Samples, TMS/TCDS

Correspondence address: Mrs Prof Balconi, Deprtment of Psychology, Catholic University of Milan, largo gemelli 1, D-20123 milan, Italy. E-mail: michela.balconi@unicatt.it

**P3.16**

**Proper name anomia after left temporal lobectomy: A long-term effect of uncinate fasciculus resection**

Federica Lucchelli[1], Costanza Papagno[2], Graziella Ghirardi[1], Paola Saletta[1], Alessandra Casarotti[2] and Lorenzo Bello[3]
[1]*AO G. Salvini, Servizio di Riabilitazione Neuropsicologica, Passirana di Rho, Italy*
[2]*Università di Milano-Bicocca - Dipartimento di Psicologia, Milan*
[3]*Università degli Studi di Milano - Istituto Clinico Humanitas, Milan*

GDP, a 49-year-old lady, with a degree in Pharmaceutics, underwent a left temporal lobectomy for a low grade mesio-temporal glioma. Pre-surgery neuropsychological assessment was normal. Immediate post-surgery evaluation showed verbal memory impairment and generalized naming deficits. After 6 months, verbal learning, though considerably improved, was still impaired. By then, naming of objects, actions, concrete and abstract items was in the normal range, while her performance in famous face naming was still significantly impaired. Moreover, she spontaneously complained of persistent difficulties to retrieve proper names of people as well as geographical names. An extensive evaluation of proper names was carried out, including familiar/unknown judgment tasks, retrieval of semantic information and production of names of celebrities in the visual modality (pictures) and on verbal description, as well as comprehension of proper names (pointing tasks). Geographical and famous monuments names were also investigated by picture presentation and in response to verbal definitions. In contrast with her almost perfect performance in familiarity judgment, retrieval of semantic information and comprehension, GDP was specifically and severely impaired when required to produce proper names, a pattern which qualifies as a post-semantic deficit. Geographical names were also affected, although to a much lesser extent than celebrities' names. The profile appears to be stable after more than 9 months from surgery. The MRI study showed a large resection area involving the anterior and ventral portion of left temporal lobe. Diffusion-tensor imaging fibre-tracking technique (DTI-FT) demonstrated amputation of the inferior part of the uncinate fasciculus (UF) in its temporal portion. This case adds to the previously published series by Papagno et al. (2011), further emphasizing the long-term effects of UF resection on proper names retrieval.

Keywords: Language, Other Disorder/Samples, Neuropsychological Assessment and Psychometrics

Correspondence address: Mrs dr Federica Lucchelli, AO G. Salvini, Servizio di Riabilitazione Neuropsicologica, via Settembrini 1, I-20017 Passirana di Rho, Italy. E-mail: flucch@libero.it

### P3.17

### Repetition priming in picture naming is mainly perceptual

Evelyn Mohr
*Durham University, Psychology, Hamburg, Germany*

INTRODUCTION: We were interested whether the priming behavior of patients with aphasia during a repeated naming task differs from that of healthy controls. Pictures of common objects were manipulated by colour, congruency of the background, and by the time of the delay between study and test.
METHOD: We tested 25 German-speaking patients with aphasia in the post-acute state who were diagnosed with a moderate-medium severe anomia and a control group of 10 healthy subjects matched in age (Ø 55 y). The stimulus set consisted of a picture set containing 140 pictures of common objects of different categories. Each of the pictures was presented in different variations: colour vs gray scaled; plain vs noisy background; and congruent vs incongruent coloured background. We measured RTs at study and at test. The 25 German-speaking patients and the 10 healthy German controls named the pictures again after a delay of 10 days. In another part of the experiment, we used three groups of English-speaking participants (Ø 30 y) with 20 participants in each group. The groups named the same pictures again after a delay of one, three or six months. We analyzed the priming effects of both parts of the experiment according to: picture-design; category effects, subject variables, and time of the delay using repeated measures ANOVAs.
RESULTS: Aphasic subjects showed the same priming effects as the age matched control group and the three English groups with faster naming times of on average 83ms. Priming effects were unaffected by the visual manipulations of the pictures and unrelated to the category of the object. Furthermore, priming effects were unaffected by the time of the delay, by linguistic variables (English, German), by the difficulty to name the picture, and by subject variables such as age, and aphasia.
CONCLUSION: Our results may indicate that priming effects during repeated picture naming (of the same picture) are mainly perceptual and unaffected by linguistic variables.

Keywords: Language, Stroke, Neuropsychological Assessment and Psychometrics

Correspondence address: Dr. phil. Evelyn Mohr, Durham University, Psychology, Olendörp 2, D-22335 Hamburg, Germany. Tel.: +49 4081987275; E-mail: dr.evelynmohr@googlemail.com

### P3.18

### When ordered words make someone silent: A case with selective difficulties in accessing the phonological output buffer when pronouncing ordinal information

Jean-Philippe Van Dijck[1], Katleen Vandeput[2], Christophe Lafosse[2], Rob Hartsuiker[1] and Wim Fias[1]
[1]*Ghent University, Experimental Psychology, Ghent, Belgium*
[2]*Rehabilitation Hospital Revarte, Scientific Unit, Antwerp, Belgium*

We describe a woman suffering from (subtle) damage to the white matter beneath the frontal cortex, who presented her to the rehabilitation hospital with severe word finding problems (i.e. complete blocking) in the spontaneous production of spoken numbers. Standardized evaluation revealed that these problems were also present when reading Arabic digits aloud and also extended to other information when assessed in an ordinal context (e.g. backward recitation of days, months and the retrieval of letters stored in serial order working memory). Interestingly, (number) word reading, writing, comprehension and calculation were flawless. More detailed investigations revealed an intact phonological (output) lexicon but a selective deficit accessing the phonological output buffer. These findings extend previous observations by demonstrating that not only numbers but also other types of ordinal information have a special status up to the final levels of spoken language production. Currently she undergoes TDCS therapy over the frontal cortices and on-going investigations will evaluate whether or not this will improve these production difficulties.

Keywords: Language, Stroke, Neuropsychological Assessment and Psychometrics

Correspondence address: Jean-Philippe van Dijck, Ghent University, Experimental Psychology, H. Dunantlaan 2, B-9000 Ghent, Belgium. Tel.: +32 9264264 33; E-mail: jeanphilippe.vandijck@ugent.be

### P3.19

### Normalization of the Verbal Fluency Test in Polish population

Daria Biechowska, Dariusz Dziegielewski and Ksenia Slawinska
*Institute of Psychiatry and Neurology, Warsaw, Poland*

INTRODUCTION: Verbal fluency is the ability to form and express words compatible with required criteria. Verbal fluency is necessary for optimal communication and for normal social and occupational functioning. The Verbal Fluency Test is a good indicator of frontal lobe dysfunction, particularly of the left frontal cortex.
MATERIAL AND METHODS: The aim of the study was to compare verbal fluency in healthy subjects ($n = 250$), patients with organic lesions of the central nervous system (CNS) ($n = 100$), and patients with type 2 diabetes ($n = 50$). There were no differences between male and female performance. Age was significantly related to performance in the Verbal Fluency Test.
RESULTS: Healthy subjects and patients with diabetes achieved the highest results in all categories of the Verbal Fluency Test. Patients with organic lesions of the central nervous system achieved significantly lower results. Sten norms of the Verbal Fluency Test were developed for the general population. Using these norms it was found that subjects with organic lesions of the CNS had very poor results more often and very high results less frequently compared to healthy subjects and also to patients with diabetes.
CONCLUSION: We have demonstrated that in patients with type 2 diabetes verbal fluency is comparable with healthy subjects. The results are compatible to the literature and, thought preliminary, they may be used as reference in research and clinical settings in our country.

Keywords: Language, Stroke, Neuropsychological Assessment and Psychometrics

Correspondence address: Dr Daria Biechowska, Institute of Psychiatry and Neurology, Sobieskiego 9, PL-02-957 Warsaw, Poland. E-mail: dbiechowska@ipin.edu.pl

### P3.20

### The brainstem and cognitive (dys) functions: From neural disconnection to behavioral disregulation

Marco Pitteri[1], Martina Garzon[2] and Silvia Albanese[2]
[1]*IRCCS San Camillo Hospital, Laboratory of Neuropsychology, Lido-Venice, Italy*
[2]*IRCCS San Camillo Hospital, Lido-Venice, Italy*

The traditional view on the link between brainstem lesion and cognitive functions has recently been challenged by results from neuroimaging and clinical studies on groups (Baillieux et al., 2010) and single cases (Maeshima et al., 2010). The potential for brainstem lesions to produce severe and chronic cognitive impairments is not widely recognized. We describe a 46yearold, righthanded man (BS), with 13 years of education, who suffered from a lesion in the right pontine area. In the postacute phase, BS presented with dysarthria, diplopia, ataxic gait, deficit of fine motility of the right hand, and a constellation of neuropsychological deficits. These signs remained unchanged after physical and speech therapy. At about one year from lesion onset, MRI disclosed a small hemorrhage involving the right mesencephalic tegmentum and tectum, without affecting the red nucleus. The superior cerebellar peduncle was also involved, leading to pseudohypertrophy of the left inferior olivary nucleus because of transynaptic degeneration. A PET scan showed hypometabolism of the brainstem, the right cerebellum, and the left cerebral hemisphere, particularly in the thalamus, the insula, and the frontal area. BS had cognitive and behavioural disorders and mild speech defects. His prosody was altered in cadence and voice intonation, suggesting a plausible foreign accent syndrome (Dankovičová and Hunt, 2011). In addition, with respect to other cases already described in the literature, BS showed a pronounced pragmatic deficit, characterized by difficulty taking turns in the conversation, scarce pertinence on the argument, poor exhaustivity of verbal messages, and sudden changes of argument during conversation. To the best of our knowledge, this is the first report of a patient affected by severe pragmatic disorder after brainstem lesion. We suggest that diaschisis-related disorders should be carefully contemplated in clinical practice.

Keywords: Language, Stroke, Neuropsychological Assessment and Psychometrics

Correspondence address: Mr PhD Marco Pitteri, IRCCS San Camillo Hospital, Laboratory of Neuropsychology, Via Alberoni, 70, IT-30126 Lido-Venice, Italy. e-mail: marco.pitteri@ospedalesancamillo.net

### P3.21

### Functional recovery in subcortical crossed and non-crossed aphasia

Virginia Maria Borsa[1], Pasquale Della Rosa[2], Matteo Canini[3], Stefano Francesco Cappa[4] and Jubin Abutalebi[3]
[1]*Vita-Salute San Raffaele University, Cognitive Neuroscience, Milano, Italy*
[2]*IBFM-CNR, Institute of Molecular Bioimaging and Physiology, Milan, Italy*
[3]*Vita-Salute San Raffaele University, Cognitive Neuroscience, Milan, Italy*
[4]*Neurorehabilitation Unit, San Raffaele Scientific Institute; Vita-Salute San Raffaele University, Clinical Neuroscience; Cognitive Neuroscience, Milan, Italy*

Crossed aphasia usually defines an acquired language disorder due to a lesion in the non-dominant right hemisphere in a natural right-hander with a structurally intact left-hemisphere and with no familiar history of left-handedness, early brain damage or epilepsy.
There are very few longitudinal studies in literature (Heiss et al., 1999; De Boissezon et al., 2005) investigating functional recovery in stroke patients affected by subcortical lesions, and to the best of our knowledge there are no longitudinal follow up studies of subcortical crossed aphasia.
The aim of our study was to investigate functional recovery in a patient with subcortical crossed aphasia and in an age-matched control patient with aphasia due to a similar lesion but located in left subcortical areas. Both patients were severely anomic and underwent the same type of speech therapy, i.e., a deficit specific speech therapy for approximately six weeks that was followed by a more general speech therapy (see Vitali et al., 2007, for details). Both patients were enrolled in the same follow-up protocol and were tested and scanned with fMRI for three times (i.e. at T0 before being enrolled in the experimental study, at T1 after the deficit specific speech therapy and at T2 after the global speech therapy).
fMRI results showed the similar pattern of reorganization (i.e. major involvement of contralateral language areas) of the neural substrates underlying overall naming activations in both the crossed and uncrossed aphasia patient. Furthermore, fMRI results pointed out the grounding role for homologue naming-specific areas (i.e. LIFG or RIFG) in determining the progressive pattern of behavioural naming recovery throughout different disease phases.
Functional recovery paralleled with an improvement in behavioural performance for naming in both crossed and uncrossed aphasia, seems to be strictly related to recruitment of homologues language-specific areas in the hemisphere opposite to the lesion.

Keywords: Language, Stroke, Functional and Structural Neuroimaging/NIRS

Correspondence address: Mrs phd student Virginia Maria Borsa, Vita-Salute San Raffaele University, Cognitive Neuroscience, via Olgettina 60, ITALY-20100 Milano, Italy. E-mail: virginia.borsa@gmail.com

**P3.22**

**Effects of repeated theta burst stimulation on aphasia recovery**

Rahel Schumacher[1], Dario Cazzoli[2], Klemens Gutbrod[1], Monica Koenig[3], Tim Vanbellingen[4], Thomas Nyffeler[5] and René Müri[1]
[1]*Inselspital Bern University Hospital, and University of Bern, Department of Neurology, Division of Cognitive and Restorative Neurology, Bern*
[2]*University of Oxford, Nuffield Department of Clinical Neurosciences, Oxford, United Kingdom*
[3]*Spitalzentrum Biel, Logopädie, Biel, Switzerland*
[4]*Inselspital, Bern University Hospital and University of Bern, Department of Neurology and Department of Clinical Research, Perception and Eye Movement Laboratory, Bern, Switzerland*
[5]*Luzerner Kantonsspital, Department for Internal Medicine, Center of Neurology and Neurorehabilitation, Luzern, Switzerland*

Aphasia is common after left-sided stroke and is characterized by partial or total loss of language functions. Functional imaging studies examining language recovery after stroke often find an overactivation of the non-damaged right hemisphere. One hypothesis is that the overactivation is dysfunctional which is explained within the framework of interhemispheric inhibition. Non-invasive brain stimulation techniques such as transcranial magnetic stimulation (TMS) allow modulating cortical activity and may thereby offer novel therapeutic opportunities. A repetitive TMS protocol, the so-called theta burst stimulation (TBS), is increasingly used in clinical research. It has the advantage of a short application time combined with prolonged aftereffects. In a previous study we found better naming performance in aphasic patients after the application of one train of TBS over the right Broca's homologue. Applying TBS trains repeatedly can furthermore disproportionately prolong the effects, as was for example shown in neglect patients. The aim of the present study is to evaluate the effects of repeated TBS on several language tasks over a longer time span. Repeated trains of TBS are applied in a randomized, sham controlled, cross-over design. After comprehensive baseline testing, eight TBS trains and eight sham stimulation trains are applied over the right Broca's homologue on two consecutive days separated by one week. On the second days of stimulation, several short language tests are administered. A follow-up with comprehensive language testing takes place one month after the stimulation. Preliminary results of this ongoing study will be presented.

Keywords: Language, Stroke, TMS/TCDS

Correspondence address: Mrs Rahel Schumacher, Inselspital Bern University Hospital, and University of Bern, Department of Neurology, Division of Cognitive and Restorative Neurology, Freiburgstrasse, CH-3010 Bern. E-mail: rahel.schumacher@dkf.unibe.ch

**P3.23**

**The dual-route approach to object processing supported by voxel-based morphometric (VBM) analysis: Evidence from the BCoS assessment**

Johnny King Lau[1], Wing-ling Bickerton[1], Glyn Humphreys[2], Carmel Mevorach[1] and Pia Rotshtein[1]
[1]*University of Birmingham, Psychology, Birmingham, United Kingdom*
[2]*University of Oxford, Experimental Psychology, Oxford, United Kingdom*

Evidence from neuropsychological and imaging research suggests that knowledge of actions regarding object manipulation (how an object is used) can dissociate from knowledge of objects (what the object is). On the other hand, there is also evidence suggesting that both object recognition and retrieval of manipulation information draw on a common pool of semantic representations. The aim of our study was to identify the similarities and differences in neural correlates of object recognition and actual object use. 247 right hand sub-acute stroke patients (128F, 70.43 ± 14.51 yos) completed visual object naming (ObjN) and multi-object use (MOU) tasks taken from the BCoS battery. Clinical neuroimaging data – CT scans were obtained to assess grey matter integrity. To isolate the underlying cognitive components, we performed a principal component analysis (PCA) on the scores of the 2 tasks. Correlations of grey matter and performances were tested using the raw task scores as well as the identified principal component scores. Performances on the ObjN and MOU tasks were highly correlated ($r = 0.49$, $p < 0.0001$). PCA identified a shared component that explained 74.5% of the data and a component that distinguished between the two tasks accounting for 25.6% of the data. Lesions to the left anterior temporal, left inferior frontal and medial visual cortices could lead to impairment in both tasks. Specific ObjN deficits were related to lesions to the left fusiform while deficits in the MOU task were predicted by lesions to the left parieto-occipital junction.
CONCLUSION: Two object processing routes were identified: 1) visual object naming was associated with lesions to ventral visual stream; 2) actual use of objects was associated with lesions to dorsal visual stream. Despite these dissociated routes, lesions to semantic associated regions e.g. anterior temporal gyrus could lead to impairment in both tasks.

Keywords: Language, Stroke, Functional and Structural Neuroimaging/NIRS

Correspondence address: Mr Johnny King Lau, University of Birmingham, Psychology, Edgbaston, B15-2TT Birmingham, United Kingdom. Tel.: +44 0788193 7960; E-mail: johnnykinglau@gmail.com

**P3.24**

**Prognostic factors of aphasia recovery: A study on 196 stroke patients**

Valentina Varalta[1], Marialuisa Gandolfi[1], Barbara De Giovannini[2], Cristina Fonte[1] and Nicola Smania[1]

[1]*Neuromotor and Cognitive Rehabilitation Research Centre, Neurological, Neuropsychological, Morphological and Movement Science, University of Verona, Italy*
[2]*Neurological, Neuropsychological, Morphological and Movement Science, University of Verona, Neurological, Neuropsychological, Morphological and Movement Science, University of Verona, Italy*

Aphasia affects up to 38% of stroke survivors and it is one of the major sources of disability after cerebrovascular accidents. Aphasia is persistent, debilitating and has wide-ranging negative impacts on the social and emotional well being of the stroke survivor, family and community. Anomia is one of the most common and disabling symptoms of aphasia. Recovery from aphasia following stroke varies considerably and current knowledge on its recovery is still not sufficient. Accordingly, the identification of specific prognostic factors is important in determining the recovery of aphasia. On this base, the aim of the present study is to investigate the linguistic factors that predicted the recovery of anomia at one year after stroke. We conducted a retrospective study on 196 stroke patients with aphasia. We included the data of 59 patients who were assessed 3 times over one year by a standardized test for the diagnosis of aphasia (Aachen Aphasia Test – AAT). The linguistic performances have been analyzed as a function of naming ability. The results shown a correlation between the language comprehension ability in the acute phase and recovery of anomia one year after stroke. Thus the comprehension ability in the early stage after stroke may be a prognostic factor of naming recovery. This result could allow for a better evaluation of the ability of the aphasic patient in the acute phase after stroke. Furthermore it could allow identifying which language functions treat specifically to obtain the best recovery in aphasic patients.

Keywords: Language, Stroke, Neuropsychological Assessment and Psychometrics

Correspondence address: Mrs Valentina Varalta, Neuromotor and Cognitive Rehabilitation Research Centre, Neurological, Neuropsychological, Morphological and Movement Science, University of Verona, Piazzale LA Scuro, 10, IT-37134 Verona, Italy. E-mail: valentina.varalta@univr.it

### P3.25

### Delimiting the borders of Pure Word Deafness: A single case report

Chiara Maffei[1], Marco Catani[2], Flavio Dell'Acqua[3], Michel Thiebaut de Schotten[4], Rita Capasso[5], Giulia Cazzoli[5] and Gabriele Miceli[6]
[1]*Universita degli Studi di Trento, Cimec, Center for Mind/Brain Sciences, Rovereto, Italy*
[2]*Institute of Psychiatry, King, Forensic and Neurodevelopmental Science, London, United Kindom*
[3]*Institute of Psychiatry, King, Forensic and Neurodevelopmental Science Neuroimaging NIHR BRC for Mental Health, London, United Kingdom*
[4]*Institute of Psychiatry, King, Natbrainlab, Department of Forensic and Neurodevelopmental Sciences, London, United Kingdom*
[5]*Universita Degli Studi di Trento, Centro Interdipartimentale Mente/Cervello-CIMEC, Rovereto, Italy*
[6]*Universita Degli Studi di Trento, Dipartimento di Psicologia e Science Cognitive, Centro Interdipartimentale Mente/Cervello-CIMEC, Rovereto, Italy*

In rare cases, brain damage can result in so-called Pure Word Deafness (PWD), ie, the selective loss of the ability to recognise spoken language, in the absence of a similar deficit for written language recognition and of relevant spoken and written production impairments. The disorder is of interest because of its high specificity and of its potential role in understanding the normal mechanisms of language perception and their neural underpinnings. However, results of lesion studies are inconsistent - lesions responsible for PWD are frequently large and bilateral, typically involving the grey and white matter of the temporal lobe, and the auditory processing disorders underlying PWD vary across subjects. As a consequence, even though several hypothesis have been put forward, the neurofunctional mechanisms underlying PWD are still a matter of debate.
We combined structural and diffusion imaging with deterministic tractography to analyse a single case of a woman affected by Pure Word Deafness after left unilateral cerebrovascular accident. Contrary to other cases, this patient presents with a very small lesion localised in the primary auditory cortex. The aim of the study was to precisely delimit the cortical and subcortical boarders of the lesion, with specific emphasis on the white matter pathways involved in language and auditory processing. Results from tractography reconstruction and neuropsychological tests suggest that PWD in our case results from a pre-phonemic disorder due to a unilateral lesion of the left temporal lobe. Results invite the conclusion that left primary auditory cortex implements the very first stages of processing of auditory language comprehension.

Keywords: Other Functions/Disorders, Agnosia, Other Methods

Correspondence address: Mrs Chiara Maffei, Universita degli Studi di Trento, Cimec, Center for Mind/ Brain Sciences, Palazzo Fedrigotti, corso Bettini 31, Rovereto (TN), Italy-38063 Rovereto, Italy. E-mail: chiaramff@gmail.com

### P3.26

### A study on implicit and explicit self-voice recognition

Elisa Zamagni[1], Francesco Ruotolo[2], Michela Candini[3], Santa Iachini[2] and Francesca Frassinetti[3]
[1]*University Of Bologna, Psychology, Bologna, Italy*
[2]*Second University Of Naples, Laboratory Of Cognitive Science And Immersive Virtual Reality, Department Of Psychology, Napoli, Italy*
[3]*Department Of Psychology, University Of Bologna, Bologna, Italy*

In the ongoing debate about the notion of "self" a dissociation between implicit and explicit self body knowledge has been demonstrated (Frassinetti et al., 2011; Ferri et al, 2011). A facilitation in processing visual images representing our body as compared to other people's body emerges when an implicit but not when an explicit recognition of our own body is required.
Regarding other aspects of self-recognition, a recent study on self face and self voice recognition showed that seeing one's own face and listening to one's own voice activate the same cerebral region, thus suggesting that this region is concerned with processing self-related stimuli across multiple sensory modalities and that it may contribute to an abstract self-representation (Kaplan et al., 2008).
The aim of the present study was to verify whether also for self voice recognition there is a dissociation between implicit and explicit self voice processing and whether a facilitation in discriminating self versus other people's voice emerges in implicit but not in the explicit task.

Healthy subjects were required to listen to couple of stimuli (words or non-words) that could be pronounced by participant's voice or by a familiar or unfamiliar person. Afterwards, they had to judge if the voice was the same or different (implicit task) and if his/her own voice was present or not (explicit task). Results showed a dissociation in implicit and explicit self voice recognition, that is modulated by meaningful vs. meaningless stimuli (words vs non-words).

Keywords: Other Functions/Disorders, Other Methods

Correspondence address: Dr. PhD Elisa Zamagni, University of Bologna, Psychology, Via Berti Pichat, 5, 40141 Bologna, Italy. E-mail: elisa.zamagni5@unibo.it

**P3.27**

**Age-related reduction of prefrontal cortex integrity during spatial working memory retrieval**

Max Toepper[1], Hans Markowitsch[2], Christina Haag[2], Bahar Saradjuk[2], Helge Gebhardt[3], Thomas Beblo[1], Eva Bauer[3], Stefan Kreisel[4], Friedrich Woermann[5], Christine Thomas[6], Martin Driessen[7] and Gebhard Sammer[3]
[1]*Evangelic Hospital Bielefeld (EvKB), Research Department, Bielefeld, Germany*
[2]*Department of Psychology, University of Bielefeld, Bielefeld, Germany*
[3]*Justus-Liebig-University of Giessen, Cognitive Neuroscience at Centre for Psychiatry, Giessen, Germany*
[4]*Department of Gerontology, Evangelic Hospital Bielefeld, Bielefeld, Germany*
[5]*Mara Hospital, Bethel Epilepsy Center, Bielefeld, Germany*
[6]*Evangelic Hospital Bielefeld (EvKB), Department of Gerontology, Bielefeld, Germany*
[7]*Evangelic Hospital Bielefeld (EvKB), Department of Psychiatry and Psychotherapy Bethel, Bielefeld, Germany*

Healthy aging is accompanied by a spatial working memory decline that is related to functional and structural cerebral changes within the spatial working memory network. Ambiguity remains whether these changes reflect functional deficits or compensation and whether changes within prefrontal cortex particularly affect dorsolateral parts, ventrolateral parts, or their connections to other brain regions. To address these issues, functional magnetic resonance imaging (fMRI) and diffusion tensor imaging (DTI) were used to examine brain activation during spatial working memory retrieval and white matter integrity in 18 older individuals and 18 younger individuals. Spatial working memory was assessed with a modified version of the Corsi Block-Tapping test (CBT) that requires the storage and reproduction of spatial target sequences. Activation differences between the two age groups were analyzed for all and only correct retrieval attempts. Functional connectivity analysis was performed using a seed region approach. DTI data were analyzed by tract-based spatial statistics (TBSS). Older individuals showed lower CBT performance accuracy than younger individuals. Brain data analyses revealed particularly right-hemispheric dorsolateral prefrontal 'underactivation' in older individuals. Moreover, older individuals showed decreased functional connectivity between right dorsolateral prefrontal cortex and other frontal brain regions as well as decreased white matter integrity of fronto-parietal and inter-hemispheric connections. The present results confirm a working memory dysfunction in old age indicated by right-hemispheric dorsolateral prefrontal 'underactivation' and lower performance accuracy. Together with a decreased functional and structural connectivity between different frontal brain regions in older individuals, these findings suggest an age-related reduction of prefrontal cortex integrity which may restrict working memory control processes.

Keywords: Executive Functions, Aging, Functional and Structural Neuroimaging/NIRS

Correspondence address: Dr. Max Toepper, Evangelic Hospital Bielefeld (EvKB), Research Department, Remterweg 69-71, D-33617 Bielefeld, Germany. E-mail: max.toepper@evkb

**P3.28**

**Naturalistic multitasking: Effect of normal aging and CIND**

Thomas Benke
*Klinik für Neurologie, MUI, Innsbruck, Austria*

In multitasking (MT), multiple different tasks have to be completed in a limited time period and by switching from one to another. Little is known about the demographical and cognitive correlates of MT in aging subjects. MT was studied in a group of cognitively normal controls (NCs; $n = 374$, age 66.4, education 13.1, MMSE 28.9) and a group of nondemented subjects with slight cognitive impairment (cognitive impairment, no dementia/CIND; $n = 42$, age 74.6, education 11.4, MMSE 26.6). Two naturalistic tasks were used: 1) a shopping task (participants were given recipes and asked to find the cheapest ingredients from among 6 supermarket flyers and to calculate the 'bargain' meal costs), and 2) a bus trip task (planning serial two-part bus rides in a virtual town to catch an appointment). A prospective memory task was also included (spontaneous report of information after 5 and 10 mins, respectively). The time limit for task achievement was 15 min, and lapse of time was visible on a clock located on the desk. Only properly solved tasks were scored. Bonus points were given if tasks from more than one category were resolved. Subjects with CIND performed significantly poorer than NCs on all three MT tasks, including prospective memory. NCs switched tasks significantly more often than CIND subjects. Multiple linear regression analyses showed age, education and diagnostic group, but not depression, gender or independent living as significant predictors for MT performance (R2 = 0.42). Best neuropsychological predictors were Trail B, word fluency, a health numeracy task, the FAB and a memory screen score (R2 = 0.49). In sum, naturalistic MT showed substantial decrements related to age, education and cognitive functions. A clear relationship of MT performance and age related cognitive changes of memory, speed and executive functions was found. Patients with CIND are significantly impaired in naturalistic MT as compared to normal aging subjects.

Keywords: Executive Functions, Aging, Neuropsychological Assessment and Psychometrics

Correspondence address: Prof.Dr. Thomas Benke, Anichstr. 35, A-6020 Innsbruck, Austria. E-mail: thomas.benke@i-med.ac.at

**P3.29**

### Goal maintenance, fluid intelligence and cognitive ageing

Matthew Iveson[1], Sarah E. MacPherson[2], Mike Anderson[3] and Sergio Della Sala[2]
[1]*The University of Edinburgh, Department of Psychology, Edinburgh, United Kingdom*
[2]*The University of Edinburgh, Human Cognitive Neuroscience, Department of Psychology, Edinburgh, United Kingdom*
[3]*The University of Western Australia, Neurocognitive Development Unit, School of Psychology, Perth, Australia*

BACKGROUND: Goal Maintenance is the process by which the rules that guide task-relevant behaviour are kept active so as to exert their control. When this process fails, an individual may ignore a rule while performing the task despite being able to describe it after task completion. Duncan and colleagues have shown that high fluid intelligence correlates with an improved ability to maintain a complex set of rules, and neglect-like errors increase with lower fluid intelligence abilities, particularly when the complexity of the task instructions is high.
AIM: This study examined instructional complexity independently of maintenance duration and task complexity.
METHODS: The first study involved 48 adults aged 40–70 years performing two sub-task blocks of a rapid serial visual presentation task. In the second study, we tested 37 younger (aged 18–35 years) and 35 older (aged 60–80 years) adults where 3- or 4-instructions were given for one task block, with the extra instruction being irrelevant to the task.
RESULTS: In the initial study, we observed a significant interaction between fluid intelligence and instructional complexity, with low-intelligence individuals committing more neglect-like errors than high-intelligence individuals but only when concurrently maintaining instructions for both sub-task blocks. In contrast, there was no effect of instructional complexity in the second study. Despite significant main effects of age group and intelligence group, there was no interaction between intelligence group and instructional complexity in either age group.
CONCLUSION: Goal Maintenance abilities may decline with age, but fluid intelligence may not directly relate to the ability to maintain complex rules.

Keywords: Executive Functions, Aging

Correspondence address: Mr Matthew Iveson, The University of Edinburgh, Department of Psychology, George Square, EH8 9JZ Edinburgh, United Kingdom. E-mail: M.Iveson@sms.ed.ac.uk

### P3.30

### Heterogeneity of cognitive functionind and its disorders in elderly people

Natalia Treder
*University of gdansk, psychology, Gdansk, Poland*

Neuropsychological literature suggests that there is a link between changes occurring in one's brain as a result of aging and cognitive processes. Age-related cognitive functioning variation does not follow a one specific pattern. The rate of aging of particular neurophysiological and cognitive processes has interindividual disharmonies and interindividual differences. The purpose of the study was to characterize the cognitive processes in the elderly people.
The presented research involves 79 people who are divided into two groups: experimental and control one. Experimental group ($N = 44$) consists of people who are 65 years of age. Control group ($N = 35$) includes the adults in the 40 to 55 age band.The methods used in the research are popular neuropsychological tests: Mini Mental State Examination, Rey Osterrieth Compex Figure Test, Controlled Oral Word Association Test, Digit Span from the Wechsler Adult Intelligence Scale, Block Designe Test from the Wechsler Adult Intelligence Scale, Trail Making Test. The thesis shows a global level assessment of working, immediate, long-term, visual-spatial memories functioning, attention, executive processes and abilities of visual-spatial synthesis.
In the presented research done profile analysis. Transformation of the raw results into counted and expressed on standard scale. Cognitive problems were common in elderly people but they had individual and varied character. There was regularity that in the elderly people was the prevalence of executive dysfunctions. The most problematic task was the one involving working memory, the one requiring divisibility of attention, switching and concentration of attention.

Keywords: Executive Functions, Aging, Neuropsychological Assessment and Psychometrics

Correspondence address: Mrs master Natalia Treder, University of gdansk, psychology, Bażyńskiego 4, 80-952 Gdansk, Poland. Tel.: +48 660434667; E-mail: psynt@ug.edu.pl

### P3.30

### Intraoperative monitoring of executive functioning in brain tumor patients

Martine Van Zandvoort[1], Irene Huenges Wajer[2], Carla Ruis[2], Marike Broekman[3], Anouk Smits[2], Tom Snijders[2] and Pierre Robe[3]
[1]*Utrecht University/University Medical Centre, Experimental Psychology/ Neurology, Utrecht, Netherlands*
[2]*University Medical Centre, Utrecht, Neurology, Utrecht, Netherlands*
[3]*University Medical Centre, Utrecht, Neurosurgery, Utrecht, Netherlands*

Awake brain surgery allows extensive intraoperative monitoring of not only motor and sensory functions and language, but also of executive functioning. The Stroop effect is assumed robust and sensitive to the frontal cortex. Feasibility and implications of adapted Stroop paradigm are investigated and related to neuronavigation screen captures and pre and post-surgery neuropsychological examination.
52 patients (38 left and 13 right hemisphere primary brain tumors; 29 low-grade and 23 high-grade gliomas) underwent debulking in an awake-awake-awake procedure and had intraoperative cognitive monitoring according to clinical care as usual. Patients are grouped on Stimulation effect (Stroop error (including slowness) or not) and the influence of lesion side and site (screen captures superimposed on normalized brain) and histotype will be analyzed (nonparametric). In addition, neuropsychological performance pre and 6 months post surgery will be taken into account (ajusted anova ).
The Stroop paradigm was applied intraoperatively in 32 (10 right hemisphere) of 52 patients. In 13 patients the Stroop was not feasible to pre-existing deficits (4 language impairments, 1 language barriere, 2 executive impairments, 1 fatigue) or clinical priorities on language or motor functioning. In 7 patients the Stroop was not applied (debulking of parietal/occipital tumors). Deficits on stimulation was found in 75% of the patients and in 62% of the patients influenced resection. No significant differences are found between left and right hemisphere. Feasibility level was in favor of low-grade gliomas.
The intraoperative Stroop paradigm is feasible in both high and low-grade frontal primary brain tumor patients in whom pre-operative Stroop is feasible. No differences in either feasibility nor elicited Stroop effect between right and left frontal tumors was found. Findings with respect to site of the lesion and relation with overall neuropsychological functioning will be discussed.

Keywords: Executive Functions, Other Disorder/ Samples, Invasive Techniques

Correspondence address: PhD Martine van Zandvoort, Utrecht University/ University Medical Centre, Experimental Psychology/Neurology, Heidelberglaan 2, NL-3584 CS Utrecht, Netherlands. E-mail: m.vanzand voort@uu.nl

### P3.31

### The impact of institutionalization upon cognition and behavior in institutionalized patients with Alzheimer's disease

Munteanu Cornelia-Eugenia
*CMDT - -, Bucharest, Romania*

A family can hardly experience and handle the sudden news that a beloved is diagnosed with Alzheimer dementia (AD), and that the latter's long-term care requires patience, moral and physical energy and extended time. Where the family fails, institutionalization becomes a solution. This paper aims at spotting total care effects upon cognition and behavior in AD patients. It is obvious the medical staff's tendency to overcame patients who are no longer allowed to do what they could, thus inducing cognitive distortion "I don't know, I can't". 56 AD patients ($M = 78.32$ years, $SD = 2.51$ years) were functionally assessed at institutionalization and six months later, analyzing the items to detect if patients could perform routine activities without help: eat and drink, stand up and sit down on chairs or bed, use the bathroom, do their toilet, get upstairs or downstairs, get dressed or undressed, use the remote control or the phone. Results indicated that 72% of all AD institutionalized patients behaved according to the thought "I don't know I can't" and not as a consequence of cognitive deterioration. Time pressure upon medical staff's duty fulfillment causes a distorted reality to patients: they accept being fed instead of eating themselves, wearing diapers instead of going to toilet, etc. Care of AD institutionalized patients must count what abilities they still have, let them perform routine activities, and offer help only if necessary. Patients must think and behave according to their own reality and not to an induced one.

Keywords: Executive Functions, Dementia, Neuropsychological Assessment and Psychometrics

Correspondence address: PhD Munteanu Cornelia-Eugenia, CMDT - -, -, Ro– Bucharest, Romania. E-mail: c_e_munteanu@yahoo.com

### P3.32

### Neural processing of interference control in elderly males with and without amnestic mild cognitive impairment

Margarethe Korsch[1], Helmut Hildebrandt[2], Sascha Frühholz[3] and Manfred Herrmann[4]
[1]*Department of Neuropsychology and Behavioral Neurobiology, Bremen University, Bremen, Germany*
[2]*Oldenburg University, Oldenburg*
[3]*University of Geneva*
[4]*Bremen University, Bremen*

Amnestic Mild Cognitive Impairment (aMCI) represents a transitional state between healthy ageing and clinical conditions such as dementia, and is characterized by predominant memory impairments. However, recent research also demonstrates executive dysfunctions to be highly affected in subjects with aMCI. Here, we mainly focused on interference processing as impairments of inhibition might cause or increase memory dysfunction. To investigate whether different aspects of interference control are altered in persons with aMCI, 9 elderly male participants with aMCI and 10 healthy male controls performed a combined Simon and Flanker task during fMRI recording. With regard to behavioral performance, no differences could be detected between both groups. However, imaging data demonstrated group-related differences in frontal and parietal areas. These findings might indicate that the transition of healthy to pathological ageing goes along with modulations of the neural networks supporting interference control.

Keywords: Executive Functions, Mild Cognitive Impairment, Functional and Structural Neuroimaging/ NIRS

Correspondence address: Mrs Margarethe Korsch, Department of Neuropsychology and Behavioral Neurobiology, Bremen University, Hochschulring 18, D-28359 Bremen, Germany. E-mail: korsch@uni-bremen.de

### P3.33

### The particular features of mental ontogenesis of preschool children with signs of subcortical brain structures dysfunction on electroencephalogram

Alfiya Sultanova and Irina Ivanova
*Russian Academy of Education, Institute for psychological and pedagogical problems of childhood, Moscow, Russia*

Studies of the relationship between psycho-physiological characteristics of children and the features of children's mental development are of high importance. This research is dedicated to the analysis of 5–7 years old children with the most common EEG abnormalities – dysfunctions of subcortical-stem structures of the brain. We applied the following research methods: battery of neuropsychological Luria's tests, parental and kindergarten personnel surveys, observational research methods. 80 children have been examined: 40 children in the main group and 40 – in the control group (control group – healthy children without any abnormalities in EEG data).
According to the research data, the most common deviations of the children in the main group were neurodynamic disorders: fatigue, psychic inertness, decrease in working capacity, fluctuation of speed, and efficiency of mental activity. Many of these children were showing the signs of the deterioration of audio-verbal and visual memory, phonemic hearing retardation, disorders of spatial gnosis and praxis, tactile perception, and motor development. Particularly, it should be noted that our research revealed the reduced self-control capability and the lag in the development of executive functions carried out by the prefrontal cortex area in all the children (although the EEG data did not reveal the failure of these brain structures).
Consequently the results of the study are useful to specify the role of development (influence? interrelationship?) of subcortical brain structures and frontal areas of cortex.
The above-mentioned features lead to difficulties in mastering a number of skills, developmental delay of game activity, impede their social adaptation, and lead to the risk of school failure in the future.
Thus, the functional failure of subcortical structures has a specific influence on the course of mental ontogenesis.

Keywords: Executive Functions, Mild Cognitive Impairment, Neuropsychological Assessment and Psychometrics

Correspondence address: Mrs Alfiya Sultanova, Russian Academy of Education, Institute for psychological and pedagogical problems of childhood, Eniseiskaya, 32-1-108, RU-129281 Moscow, Russia. Tel.: +7 9057049353; E-mail: alfiya_sultanova@mail.ru

### P3.34

### Characterization of executive functions profile in patients with cerebellar damage

Silvia Clausi[1], Claudia Iacobacci[2], Anna Maria Tedesco[3], Giusy Olivito[3], Marco Molinari[4] and Maria Leggio[3]
[1]*Department of Psychology, "Sapienza" University of Rome, Ataxia Laboratory, Santa Lucia Foundation, Roma, Italy*
[2]*Department of Psychology, "Sapienza" University of Rome, Rome, Italy*
[3]*Department of Psychology, Sapienza University of Rome, Ataxia Laboratory, Santa Lucia Foundation, Rome, Italy*
[4]*Ataxia Laboratory, Santa Lucia Foundation, Rome, Italy*

"Executive functions" (EFs) refers to the ability to orchestrate different cognitive tasks to attain specific goals. There is a consensus that the lateral PFC and basal ganglia together with the cerebellum play a critical role in EFs. The cerebellar involvement in EFs is supported by the existence of specific anatomical connections with the lateral PFC, but its nature remains unclear.
Aim of the present study was to extensively investigate the EFs subcomponents in patients affected by cerebellar damage to characterize the executive components that need of the cerebellar modulation.
Twenty-four patients affected by cerebellar atrophy (CA) or focal cerebellar damage (FCD), and 44 matched healthy controls were studied. Each patient underwent MRI examination and a motor scale. Intellectual level (IL) and cognitive domains were assessed in all subjects by an extensive neuropsychological evaluation. A large EFs battery, also including ecological tasks, was used to assess specific subcomponents: working memory, planning and problem solving, multitasking, and inhibition. A one-way Anova and Tukey's post hoc test were performed; a Spearman correlation between motor and cognitive scores was also done.
The patients showed good IL without significant correlation between motor impairment and cognitive scores. In general, patients' cognitive performances were in line with Cerebellar Cognitive Affective Syndrome (Schmahmann & Sherman, 1998) and Cerebellar Cognitive Profile (Tedesco et al 2011) characteristics. Specifically regarding EFs, all patients were particularly affected in tasks that required new and more complex strategies and sequencing processing. Furthermore, differences were observed between FCD and CA groups, with the former performing worse than the latter.
This is the first systematic study that describes the "cerebellar dysexecutive syndrome" and further supports the idea of sequencing as the basic operational mode of the cerebellum (Molinari et al 2008).

Keywords: Executive Functions, Neuropsychological Assessment and Psychometrics

Correspondence address: Dr. Silvia Clausi, Department of Psychology, "Sapienza" University of Rome; Ataxia Laboratory, Santa Lucia Foundation, Via dei Marsi, 78; Via Ardeatina, 306, IT-00185 Roma, Italy. Tel.: +39 0651501115; E-mail: silvia.clausi@gmail.com

**P3.35**

**Neuropsychological findings in Episodic Ataxia type2**

Elfriede Karner, Wolfgang Nachbauer, Margarete Delazer and Sylvia Boesch
*Innsbruck Medical University, Clinical Department of Neurology, Innsbruck, Austria*

Episodic Ataxia type2 (EA2) belongs to the group of dominantly inherited neurodegenerative ataxias. It is characterized by recurrent attacks of ataxia often accompanied by nystagmus, which can last from several hours to several days. Attacks are often triggered by stress or physical effort. Clinical overlap exists with allelic disorders, namely familiar hemiplegic migraine type 1 and spinocerebellar ataxia type 6, both characterized by additional slowly progressive pure cerebellar atrophy. Little is known about the cognitive and behavioural impairments of episodic ataxias. Learning deficits due to abovementioned triggers and panic attacks mostly later in the course of disease have been described. Thus, no study so far has systematically assessed cognitive functions and mood in episodic ataxias. Five EA2 patients out of a genetically assigned family agreed to be tested in a wide range of cognitive functions. Performance was classified according to standardized norms. 4 out of 5 patients showed good verbal intelligence, one patient had a verbal IQ of 70. All patients had variable impairments in executive functions including verbal working memory, verbal fluency, cognitive flexibility and set shifting. Additionally, deficits were found in verbal and visual memory, in attention, in visuo-constructional abilities, as well as in fine motor manipulations and syllable repetition. Anxiety score was high in one patient, most patients tended to have mild depression. Results of the present study suggest that EA2 is associated with cognitive deficits. These results are in line with other investigations showing that long-term functional impairment of cerebrocerebellar circuits may lead to cognitive deficits. Findings of the study suggest that patients affected by EA2 should undergo neuropsychological assessment. Still, more studies in larger patient groups are needed to highlight the relationship between the frequency of attacks and neuropsychological outcome.

Keywords: Executive Functions, Neurodegenerative Diseases, Neuropsychological Assessment and Psychometrics

Correspondence address: Mrs Master Elfriede Karner, Innsbruck Medical University, Clinical Department of Neurology, Anichstrasse 35, A-A Innsbruck, Austria. Tel.: +43 51250423869; E-mail: elfriede.karner@uki.at

**P3.36**

**Frontal white matter lesions contribute more than grey matter changes in determining cognitive deficits in patients with non-amnestic mild cognitive impairment**

Mario Torso[1], Laura Serra[1], Roberta Perri[2], Lucia Fadda[3], Barbara Spanò[1], Nadia Tini[2], Diana Castelli[4], Camillo Marra[2], Carlo Caltagirone[5] and Marco Bozzali[1]
[1]*IRCCS Fonsazione Santa Lucia, Neuroimaging Laboratory, Roma, Italy*
[2]*IRCCS Fonsazione Santa Lucia, Department of Clin-*

ical and Behavioural Neurology, Roma, Italy
[3]IRCCS Fonsazione Santa Lucia, Department of Neuroscience, University of Rome 'Tor Vergata', Roma, Italy
[4]Institute of Neurology, Università Cattolica, Roma, Italy
[5]IRCCS Fonsazione Santa Lucia, Department of Clinical and Behavioural Neurology, Department of Neuroscience, University of Rome 'Tor Vergata', Roma, Italy

BACKGROUND: Non-amnestic mild cognitive impairment (na-MCI) represents a peculiar clinical phenotype that differs, in terms of clinical evolution, from the amnestic MCI form. White Matter Hyperintensities (WMHs) are more frequent in patients with MCI than in healthy subjects (HS). Nevertheless, their putative role in neurodegeneration remains unclear.
AIMS: To investigate the role of WMHs in accounting for the clinical/neuropsychological features of na-MCI.
METHODS: Fifteen na-MCI patients (all with executive dysfunctions and Hachinsky score $< 4$) and 15 HS underwent clinical/neuropsychological assessment and MRI at 3T, including T1-weighted volumes for quantification of grey matter parenchymal-fraction (GMPF) and proton-density/T2-weighted scans for WMH anatomical localization and volume (WMHvol) assessments. WMHs were first classified as periventricular (PVH) or deep (DWMH), and then as frontal (FWMH), temporo-insular (TIWMH), parieto-occipital (POWMH), or in the basal ganglia (BGWMH). Regression models were used to assess the contribution of GMPF, lesion characteristics, and vascular risk factors in accounting for patients' characteristics.
RESULTS: na-MCI patients were not significantly different from HS in GMPF. Conversely, they showed a significantly higher WMHvol in the whole brain, and a greater DWMH extension in the frontal lobes. Regression analyses revealed the presence and extension of FWMH as best predictors for patients' performance at tests for executive functions.
CONCLUSION: The present study suggests a prominent role for FWMH in the pathophysiology of na-MCI patients. This contribution is apparently unrelated with patients' demographic characteristics and vascular risk factors. Further studies, using quantitative MRI techniques are needed to better clarify the relationship between FWMH and neurodegeneration.

Keywords: Executive Functions, Neurodegenerative Diseases, Functional and Structural Neuroimaging/ NIRS

Correspondence address: Mr Mario Torso, IRCCS Fonsazione Santa Lucia, Neuroimaging Laboratory, Via Ardeatina 306, D-00179 Roma, Italy. Tel.: +39 0651502547; E-mail: m.torso@hsantalucia.it

### P3.37

### Impaired executive functioning in amyotrophic lateral sclerosis (ALS) with or without frontotemporal dementia (FTD)

Elisabeth Kasper[1], Christina Schuster[2], Judith Machts[3], Maria Veit[3], Stefan Vielhaber[4], Reiner Benecke[5], Stefan Teipel[2] and Johannes Prudlo[5]
[1]German Center for Neurodegenerative Diseases (DZNE), Site Rostock/Greifswald, Rostock, Germany
[2]German Center for Neurodegenerative Diseases (DZNE), Rostock, Germany
[3]German Center for Neurodegenerative Diseases (DZNE), Magdeburg, Germany
[4]Department of Neurology, University of Magdeburg, Magdeburg, Germany
[5]Department of Neurology, University of Rostock, Rostock, Germany

OBJECTIVES: Due to the understanding of ALS as multisystem disease and its overlap with FTD cognitive and behavioural disturbances are considered increasingly (Goldstein, 2013). In particular, impairment of executive functions seems to be a negative prognostic factor in the disease process (Elamin, 2011). Our study aimed to characterize the cognitive profile and behaviour of ALS patients from a large cohort with emphasis on executive functions and their relevance to everyday's functioning.
METHODS: We investigated 92 ALS patients and 11 patients with ALS-FTD in comparison to 71 age and education matched healthy controls, using a comprehensive neuropsychological test battery adapted to motor incapabilities, including a behavioural questionnaire. Executive test variables were assigned to their corresponding level of cognitive regulation (basic, complex, social and emotional) and frequency distribution has been analyzed.
RESULTS: 19% of patients without FTD and all with FTD showed executive function impairment. The extent of basic function disorders (e.g. shifting) ranged from 28% to 78% in patients without FTD, from 45% to 100% in ALS-FTD subjects. In contrast, complex function disorders (e.g. monitoring) were much rarer (27% regardless of FTD), difficulties in problem solving occurred even only in cases of FTD. Behavioural disorders (e.g. disorganization or disinhibition) were apparent in 16% of ALS patients and 54% of subjects with ALS-FTD.
CONCLUSION: Executive dysfunctions have a significant relevance in the disease process of ALS patients. In contrast to a high frequency of basal regulatory disorders, complex processes as well as dysexecutive behavioural disorders are affected much less. This discrepancy can explain the relative moderate disturbances observed in clinical practice. These seem to be dependent on both the specific pattern of basic regulation deficits as well as the degree of complex regulatory disorders.

Keywords: Executive Functions, Neurodegenerative Diseases, Neuropsychological Assessment and Psychometrics

Correspondence address: Mrs Elisabeth Kasper, German Center for Neurodegenerative Diseases (DZNE) – Site Rostock/Greifswald, Gehlsheimer Straße 20, D-18147 Rostock, Germany. E-mail: elisabeth.kasper@dzne.de

**P3.38**

**The functional precursors of the relationship between multilingualism and cognitive control: A longitudinal fMRI (l-fMRI) study in multilingual children**

Pasquale Della Rosa[1], Matteo Canini[2], Virginia Borsa[2], Gerda Videsott[3], David Green[4], Rita Franceschini[3] and Jubin Abutalebi[2]
[1]*IBFM-CNR, Institute of Molecular Bioimaging and Physiology, Milan, Italy*
[2]*University San Raffaele, Center for Cognitive Neuroscience, Milan, Italy*
[3]*Free University of Bozen, Faculty of Education and Language Study unit, Bozen, Italy*
[4]*University College London, Cognitive, Perceptual and Brain Sciences, London, England*

There is recent neuro-imaging evidence showing that experience in dealing with two or more languages may have a lifelong positive impact on the brain. For instance, Della Rosa et al. (2012) have highlighted the relationship between grey matter changes in the left inferior parietal lobule and the interaction between the natural development of a multilingual talent and the capacity to resolve cognitive conflicts. As to fMRI studies, Abutalebi et al. (2011) reported that bilingual adults not only resolve cognitive conflicts with less neural activity but their brain seems also to be better tuned to monitoring cognitive conflicts.
However, the functional precursors in developmental terms of the relationship existing between multilingualism and the neural activity elicited for resolving cognitive conflicts (i.e. the conflict effect) still remain to be identified.
To this aim, we used a longitudinal event-related functional magnetic resonance imaging (l-fMRI) to investigate the effects of multilingual competence on the executive control attentional network over two years (at T1 and T2) in a group of 15 multilingual children.
SPM8 was used for slice-timing, coregistration, realignment, unwarping and noise removal processes on all functional data for both time-periods prior to longitudinal subject-specific normalization and smoothing.
A contrast coding the conflict effect independently at T1 and T2 was first computed before creating differential time-period contrast images (T2-T1) and performing correlations with multilingual competence differential scores (T2-T1).
fMRI data analysis revealed that lower levels of multilingual competence at T2 correlated with higher functional brain activity in a frontal-subcortical loop, involving the left dorsolateral frontal cortex, the head of left caudate nucleus and the left putamen, a network of brain regions which have been found to subtend cognitive control for both attention and language (Abutalebi and Green, 2007).

Keywords: Executive Functions, Other Disorder/Samples, Functional and Structural Neuroimaging/NIRS

Correspondence address: PhD Pasquale Della Rosa, IBFM-CNR, Institute of Molecular Bioimaging and Physiology, Via F.lli Cervi, 93, IT-20100 Milan, Italy. E-mail: dellarosa.pasquale@hsr.it

**P3.40**

**Disruption of social cognition in McArdle disease**

Nicky Edelstyn
*Keele University, Psychology, Staffordshire, England*

McArdle disease (GSDV) is characterised by abnormalities in glycogen accumulation in the muscles during exercise. Glycogen is also an important energy source for neurons, and brain imaging evidence suggests that abnormal glycogen accumulation also occurs in the centrum semiovale (CS), a white matter tract linking the cerebellum, prefrontal and parietal areas (Salvan, Vion-Dury, Confort-Gourny et al., 1997). Structural abnormalities in the CS and impairments in social functioning have been reported in people with autism spectrum disorder (Barnea-Goraly, 2004), raising the possibility that GSDV patients may display a cognitive profile reminiscent of that reported in autism – impaired social cognition and executive dysfunction. Three demographic- and IQ-matched groups were recruited, consisting of 9 patients with GSDV, 8 neuromuscular control patients and 8 healthy controls (HC). Both musculoskeletal patient groups had significantly higher scores on the Hospital Anxiety and Depression Scale and McGill Pain Questionnaire, and were therefore covaried the subsequent statistical analyses.All participants completed tests of verbal fluency, problem-solving, reasoning (Hayling-Brixton Tests), strategic memory (source memory), social functioning (Autism Quotient Questionnaire [AQQ]).
Compared to the HC, the GSDV group showed significant impairments in strategic memory (source misattribution), and 3 subscales of the AQQ (social skill, attention switching, imagination, total quotient). There were no significant differences between the patient groups, although the GSDV patients' performance was consistently poorer. There were no other significant differences.
These findings imply that GSDV is at one end of a continuum of cognitive dysfunction with other neuromuscular patients not as severely affected. A fully powered study, which includes more sensitive tests of executive function and social cognition, and brain imaging, will help explore these issues further.

Keywords: Executive Functions, Other Disorder/Samples, Neuropsychological Assessment and Psychometrics

Correspondence address: Mrs Professor Nicky Edelstyn, Keele University, Psychology, Keele, ST5-5BG Staffordshire, England. Tel.: +44 07951943078; E-mail: n.edelstyn@keele.ac.uk

### P3.41

### Executive function in first episodes of schizophrenia: A 1-year clinical stability

Mireia Gonzalez-Rodriguez[1], Mar Ariza[2], Arantzazu Herran-Boix[3], Sergio Bodas[3], Cristina Enero[3] and Maite Garolera[3]
[1]*Hospital de Terrassa, Consorci Sanitari de Terrassa, Neuropsychology Unit, Terrassa- Barcelona, Spain*
[2]*Brain, Cognition and Behaviour Research Group, Unitat d'Investigació biomèdica Universitat Autònoma de Barcelona CST (UIB), Terrassa- Barcelona, Spain*
[3]*Hospital de Terrassa-Consorci Sanitari de Terrassa, Neuropsychology Unit, Terrassa-Barcelona, Spain*

Longitudinal studies of first-Episode (FE) patients are of special relevance when it comes to evaluating the course of cognition in schizophrenia, since they provide a baseline measure of cognitive performance and relevant confounders associated with chronicity are also controlled. Impairment in executive functioning is considered a primary deficit in schizophrenia. They occur early in the course of the disorder and there is still a lack of prospective data specifically describing the trajectory of this executive deficit. The aim of this study was to examine the longitudinal course of executive functioning in a group of patients with a diagnosis of FE. 27 patients (74% men; mean age at baseline 25 (SD 6.4), education years 10 (SD 2.6) following a DSM-IV schizophrenia diagnosis (made at baseline and confirmed at 12-month follow-up) without comorbidity with other Axis I disorders (including drugs). All patients were under antipsychotic treatment and, were assessed after achieving clinical stability at baseline and one-year follow-up. Four standardized test measured executive functions of verbal fluency, reasoning, planning and mental flexibility (Verbal fluency, block design, Trail Making Test-B, Wisconsin Card Sorting Test). T-test for paired samples was used to assess the statistical significance of the differences between measures, and we estimated the effect size (Cohen's d) to measure the magnitude of mean differences. Alpha level was set at 0.01. Significant differences were found on verbal fluency ($p = 0.002$) with a medium effect size (0.54). Our results revealed a specific executive function improvement during early stages. It suggests a different timing in the evolutionary changes of diverse executive functions. However, our study reveals limitations (e.g. practice-effect, short period of follow-up) to establish conclusions, it needs further investigation to discern the different patterns of executive function changes over time in schizophrenia.

Keywords: Executive Functions, Psychopathology, Neuropsychological Assessment and Psychometrics

Correspondence address: Mrs Mireia Gonzalez-Rodriguez, Hospital de Terrassa- Consorci Sanitari de Terrassa, Neuropsychology Unit, Ctra. torrebonica s/n, S-08227 Terrassa-Barcelona, Spain. Tel.: +34 937310007 1557; E-mail: mgonzalezr@cst.cat

### P3.42

### Intra-individual modulation of action monitoring in patients with obsessive-compulsive disorder

Julia Preuß[1], Julia Klawohn[1], Tanja Endrass[2], Rosa Grützmann[1], Anja Riesel[1] and Norbert Kathmann[1]
[1]*Humboldt-Universität zu Berlin, Clinical Psychology, Berlin, Germany*
[2]*Otto-von-Guericke-Universität Magdeburg, Neuropsychology, Magdeburg Germany*

Obsessive-compulsive disorder (OCD) is a severe mental disorder, characterized by distressing intrusive thoughts (obsessions) and recurrent, ritualized behaviors (compulsions). Action monitoring has repeatedly been shown to be hyperactive in patients with OCD, as indexed by an enhancement of the error-related negativity (ERN) and the correct-related negativity (CRN) of the event-related brain potential. These brain potentials are generated in the anterior cingulate cortex, a region implicated in the pathophysiology of OCD. While evidence suggests that this hyperactivity is independent of symptom-state in OCD, it remains unclear whether it can be modulated by situational requirements. In the current study, we tested whether ERN and CRN amplitudes can be reduced by manipulating attentional demands. Therefore, patients and matched control participants were examined in a dual-task paradigm, combining a response conflict flanker task and a working memory n-back task. In the single task condition, participants solely responded to compatible and incompatible flanker stimuli, while presented with alternating letters preceding each flanker trial. In the dual task conditions, they additionally responded to target letters of the n-back task and thus had to monitor their actions simultaneously in two task contexts. We hypothesized that error monitoring in the flanker task would be reduced with increased difficulty of the concurrent n-back task. ERN amplitudes indicated an intra-individual down-regulation of error monitoring in OCD patients as well as healthy individuals in the more difficult flanker n-back condition. The present results suggest that changing the attentional focus can modifiy hyperactive action monitoring in OCD. On the basis of such findings new treatment approaches for OCD may be developed, e.g. attentional trainings.

Keywords: Executive Functions, Psychopathology, Electrophysiology (EEG/ERP)

Correspondence address: Mrs Julia Preuß, Humboldt-Universität zu Berlin, Clinical Psychology, Rudower Chaussee 18, D-12489 Berlin, Germany. Tel.: +49 01629621611; E-mail: julia.preuss@hu-berlin.de

### P3.43

### Profile-based analysis of dysexecutive syndrome in diabetic and after-stroke patients

Daria Biechowska[1] and Edyta Orłowska[2]
[1]*Institute of Psychiatry and Neurology, Warsaw, Poland*
[2]*Institute of Psychology, University of Gdansk, Poland*

Both diabetes and ischemic stroke are related to dysexecutive syndrome. Though typologically different both involve vascular changes in the brain. Some studies suggest different cause of poor results in executive functioning tests in diabetic and after-stroke patients, as ischemic episode results in general slowing of mental processing. The goal of this study was to establish whether there are any differences in conduct of different tests measuring various aspects of executive functions between type 2 diabetic and after-stroke patients. Neuropsychological assessment was carried out on 30 type 2 diabetic patients and 30 post-stroke patients in hospital environment up to 7 days after admission. Several dysexecutive syndrome measures were used including WCST, TMT and BADS. Age, education, gender, mood and dementia were controlled factors. Statistical analyses showed few statistically significant differences between groups in general measures mainly in Six Elements Test and TMT part B. Quantitative analysis showed however different types of mistakes made by diabetic and after-stroke patients including time spent on planning and executing tasks, perseverations, switching between tasks. Gathered results show different patterns of executive functions deficit connected with diabetes and ischemic stroke.

Keywords: Executive Functions, Stroke, Neuropsychological Assessment and Psychometrics

Correspondence address: Mrs dr Daria Biechowska, Institute of Psychiatry and Neurology, Sobieskiego 9, PL-02-957 Warsaw, Poland. E-mail: dbiechowska@ipin.edu.pl

**P3.44**

**Structural damage to the posterior limb of internal capsule in stroke patients and its association with cognitive functions**

Rosalia Dacosta[1], Manuel GrañA[2], Auer Tibor[3], Marina Fernandez-Andujar[1], Monica Millan[4], Elena Lopez-Cancio[4], Cynthia Caceres[4], Nuria Bargallo[5], Maite Barrios[6], Maite Garolera[7], Imma Clemente[8], Maria Hernandez[9], Josep Munuera[9], Antoni Davalos[4] and Maria Mataro[8]
[1]*University of Barcelona, Psychiatry and Clinical Psychobiology, BARCELONA, Spain*
[2]*University of the Basque Country, Group of Computational Intelligence, San Sebastian, Spain*
[3]*MRC Cognition and Brain Sciences Unit, Cambridge, England*
[4]*Hospital Germans Trias i Pujol, Neuroscience, Badalona, Spain*
[5]*Hospital Clínic, Radiology, BARCELONA, Spain*
[6]*University of Barcelona, Methodology of Behavioural Sciences, BARCELONA, Spain*
[7]*Consorci Sanitari de Terrassa, Neuropsychology Unit, TERRASSA, Spain*
[8]*Univdersity of Barcelona, Psychiatry and Clinical Psychobiology, BARCELONA, Spain*
[9]*Hospital Germans Trias i Pujol - Institut de Diagnostic per la Imatge, Badalona, Spain*

Following a focal stroke, the region adjacent to the injury undergoes structural changes and anterograde or retrograde degeneration of axons connecting distant regions with the lesion may occur. Sixteen patients with right hemispheric ischemic stroke and 18 matched healthy controls (HC) underwent neuropsychological assessment and diffusion tensor imaging (DTI) conducted at 3T. We performed a whole-brain analysis with TBSS of the FA index for the two groups and Pearson correlations between the FA values extracted from the tracts showing significant between-group differences and the scores of the cognitive tests. Stroke subjects demonstrated significantly reduced FA ($< 0.001$; TFCE corrected) only in the posterior limb of the internal capsule (PLIC) when compared to HC. For the stroke group, reduced FA values were negatively associated with perseverations in the Rhythm subtest (rp $= -0.576$; $p = 0.025$), and positively associated with the Semantic Fluency Test (rp $= 0.503$; $p = 0.047$) and the contrast 1 of the List of Words test (rp $= 0.698$; $p = 0.006$). Although damage to the PLIC has been mostly proposed as a predictive marker of motor outcome after stroke, this study suggests that disruptions to the right PLIC, due to its fronto-striatal connections, may contribute to cognitive deficits affecting executive and attentional functions.

Keywords: Executive Functions, Stroke, Functional and Structural Neuroimaging/NIRS

Correspondence address: Mrs Rosalia Dacosta, University of Barcelona, Psychiatry and Clinical Psychobiology, Mundet, D-08227 Barcelona, Spain. Tel.: +34 937870518; E-mail: rdacostaa@gmail.com

**P3.45**

**Prevalence and long-term prognostic value of post-stroke neuropsychological symptoms**

Hanna Jokinen[1], Marja Hietanen[2], Niku Oksala[3], Johanna Ojala-Oksala[4], Susanna Melkas[5] and Timo Erkinjuntti[5]
[1]*Helsinki University Central Hospital and University of Helsinki, Unit of Neuropsychology, Department of Neurology, HUS, Helsinki, Finland*
[2]*Helsinki University Central Hospital and University of Helsinki, Unit of Neuropsychology, Department of Neurology, Helsinki, Finland*
[3]*Tampere University and Tampere University Hospital, Division of Vascular Surgery, Department of Surgery, Tampere, Finland*
[4]*University of Tampere, School of Medicine, Surgery, Tampere, Finland*
[5]*Helsinki University Central Hospital and University of Helsinki, Department of Neurology, Helsinki, Finland*

Cognitive impairment is a common consequence of stroke, but the prevalence and long-term significance of the multifaceted neuropsychological symptoms are still not known in detail. We investigated the frequency and prognostic value of domain-specific cognitive deficits in a large cohort of elderly ischemic stroke patients. In total, 409 consecutive patients admitted to the Stroke Unit of the Helsinki University Central Hospital (Finland), aged 55-85 years, were evaluated with a comprehensive clinical and neuropsychological assessments 3 months post-stroke. Functional independence was evaluated with the modified Rankin scale (mRS) 15 months after stroke, and survival of the patients was followed-up for up to 12 years. Of the total sample, 339 (83%) patients showed impairment in at least one cognitive domain as evaluated against age-appropriate normative data. 203 (50%) cases were impaired in multiple ($\geqslant 3$) domains. In cases with good clinical recovery at baseline (mRS $\leqslant 2$), the prevalence of any cognitive deficit was still 194/255 (76%). Most commonly, the affected cognitive domains were memory, visuoconstuctional/spatial skills and executive functions. Only 29% of the total sample and 13% of the cases with good clinical recovery scored $< 25$ in the MMSE. Cognitive deficits at 3 months significantly predicted poor functional outcome in follow-up after 15 months. Furthermore, they had independent predictive value for mortality. However, years of education was related to a beneficial effect on survival. In conclusion, cognitive deficits as evaluated with detailed neuropsychological assessment are highly frequent in stroke survivors even with good clinical recovery. Typically multiple domains are impaired affecting complex cognitive abilities. MMSE is insensitive in detecting these symptoms. Cognitive deficits strongly predict functional dependence and mortality. However, educational background has a protective effect as a surrogate marker for cognitive reserve.

Keywords: Executive Functions, Stroke, Neuropsychological Assessment and Psychometrics

Correspondence address: Mrs Hanna Jokinen, Helsinki University Central Hospital and University of Helsinki, Unit of Neuropsychology, Department of Neurology, PO box 302, FIN-00029 HUS, Helsinki, Finland. Tel.: +35 8405942286; E-mail: hanna.jokinen@helsinki.fi

**P3.46**

**Fetal alcohol spectrum disorders (FASD) children with adhd have imparied working memory and behavioral problems compared to idiopathic ADHD**

Alba Corredera[1], Anna Campabadal[2], Cristina Boix[3], Roser Colomé[3], Anna López[3] and Anna Sans[3]
[1]*Universitat Autonoma de Barcelona, Psicología Clínica i de la Salud, Barcelona, Spain*
[2]*Universitat Autònoma de Barcelona, Psicología Clínica i de la Salud, Barcelona, Spain*
[3]*Sant Joan de Deu's Hospital, Neurology's Department, Barcelona, Spain*

OBJECTIVE: Prenatal alcohol exposure can cause alterations to neurodevelopmental brain. Several studies have described difficulties in many cognitive functions and more specifically in IQ, attention and executive functions. Individuals with prenatal alcohol exposure are at increased risk for ADHD. According to the ADHD ethology, children show different cognitive and behavioral profiles. As reported, children with ADHD related to prenatal alcohol consumption show greater severity and incidence of social adjustment difficulties in comparison to idiopathic ADHD. The present study aims to assess working memory as well as behavioral problems in children with FASD/ADHD in comparison with idiopathic ADHD.
METHODS: 17 adopted children diagnosed of FASD/ADHD, age ranged between 6 and 10 years, were compared with 17 idiopathic ADHD children. Both groups were matched by the same subtype of ADHD, age and sex. Working memory was assessed using Digits, Letter and Number and Arithmetic subtests of the WISC-IV. Behavior problems were assessed using the Achenbach's Child Behavior Checklist for Parents and Teachers (CBCL).
RESULTS: Statistical analysis using T student shows significant differences between both groups in Digits ($p < 0.004$), Letter and number ($p < 0.003$) and Arithmetic ($p < 0.000$) WISC-IV subtest. In related to behavioral problems, we found only significant differences in social problems subscale in teacher's CBCL ($p < 0{,}037$).
CONCLUSION: FASD/ADHD children tend to have more cognitive and behavioral problems compared to probable idiopathic ADHD. This profile is the one described in the Fetal Alcohol Spectrum Disorders. These differences in neuropsychological profile can contribute to improve the differential diagnosis, the intervention and outcome between both groups. We recommend a routine monitoring FASD/ADHD to implement therapeutic interventions as early as possible.

Keywords: Executive Functions, ADHD, Neuropsychological Assessment and Psychometrics

Correspondence address: Mrs graduate alba corredera, Universitat Autonoma de Barcelona, Psicología Clínica i de la Salud, Campues de Bellaterra-Edificio

B, D-08193 Barcelona, Spain. Tel.: +34 619968865; E-mail: alba.corpe@gmail.com

### P3.47

### Predictors of leisure participation after aneurysmal subarachnoid hemorrhage (aSAH)

Anne Buunk[1], W.S. Veenstra[1], R.J.M. Groen MD[2] and J.M. Spikman[3]
[1]*University Medical Center Groningen, Neurology, Groningen, The Netherlands*
[2]*University Medical Center Groningen, Neurosurgery, Groningen, The Netherlands*
[3]*University of Groningen, Clinical and Developmental Neuropsychology, Groningen, The Netherlands*

Occurring at a relatively young age with a peak incidence between 40 and 60 yrs, aneurysmal subarachnoid hemorrhage (aSAH) can have an immense impact on the life of the patient (de Rooij et al., 2007). Even patients with good neurological outcome experience cognitive and psychosocial deficits which influence daily functioning and life satisfaction. Leisure is defined as freely chosen non-work activities which can improve social interaction (Bundy, 1993), awareness, and enjoyment, also in people who sustained brain injury (Lyons, 1993). The aim of this study is to describe leisure participation and to investigate the factors affecting
participation in leisure activities after aSAH. 437 patients suffered from aSAH and were admitted to the UMCG between 2002 and 2009. Of the 301 survivors 25 were excluded because of comorbidity. 206 patients were willing to participate (range 17–90 yrs, mean age 58.9 yrs, 36.4% male, 63.6% female). Cognitive complaints, mood, anxiety and executive functioning in everyday life were evaluated by using questionnaires (HADS, HISC, DEX). Furthermore, all patients received a semi-structured interview by telephone with a focus on resumption of work, social, leisure activities and mobility (RRL). More than 50% of patients reported disrupted leisure participation. Changes consisted of a reduction of activities, activities at a lower level or more guided by others. Return to leisure activities is predicted by a higher HADS depression score, a greater change in mobility, and more executive problems mentioned by significant others.
Leisure participation is significantly reduced after aSAH. Factors underlying restricted participation in leisure activities are depressive mood, reduction in mobility, and dysexecutive function problems according to significant others. These factors are of great importance in designing rehabilitation to promote leisure participation which can improve social interaction and feelings of enjoyment in aSAH survivors.

Keywords: Executive Functions, Stroke, Other Methods

Correspondence address: Mrs MSc Anne Buunk, University Medical Center Groningen, Neurology, Hanzeplein 1, 9700 RB Groningen, the Netherlands. Tel.: +31 630418682; E-mail: buunkanne@gmail.com

### P3.48

### Neuronatomical correlates of processing speed impairments related to a plasma marker of thrombosis

Julia Miralbell[1], Juan Jose Soriano-Raya[1], Elena Lopez-Cancio[2], Juan Francisco Arenillas[3], Nuria Bargallo[4], Amparo Galan[5], Cynthia Caceres[2], Maite Barrios[6], Maite Alzamora[7], Guillem Pera[7], Pere Toran[7], Maite Garolera[8], Antoni Davalos[2] and Maria Mataro[1]
[1]*University of Barcelona, Psychiatry and Clinical Psychobiology, Barcelona, Spain*
[2]*Germans Trias i Pujol University Hospital, Neurosciences, Barcelona, Spain*
[3]*Neurology Service, Stroke Unit, Hospital Clinico Universitario, Valladolid, Spain*
[4]*Hospital Clinic, Radiology, Barcelona, Spain*
[5]*Germans Trias i Pujol University Hospital, Biochemistry, Barcelona, Spain*
[6]*University of Barcelona, Methodology and behavioural sciences, Barcelona, Spain*
[7]*IDIAP Jordi Gol, Primary Healthcare Reseach Support Unit Metropolitana Nord, Barcelona, Spain*
[8]*Consorci Sanitari de Terrassa, Neuropsychology Unit, Terrassa, Spain*

Plasma markers of thrombosis have been related to white matter (WM) integrity loss and lower processing speed (Miralbell et al., 2011). Our aim is to assess the specific WM tracts involved in the association between a marker of thrombosis and cognition. 86 participants from the Barcelona-ASIA Neuropsychology study were included. Subjects were 50-65 years old, free from dementia and without history of vascular disease. Plasma levels of Plasminogen Activator Inhibitor −1 (PAI-1) were used as a marker of thrombosis. Processing speed was measured with the Grooved Pegboard Test (Gpeg). First, we assessed correlations of levels of PAI-1 and performance on Gpeg with fractional anisotropy (FA) measurements of DTI. Second, we investigated the overlap between tracts involved in performance on Gpeg and those affected by higher levels PAI-1. Results show that increasing levels of PAI-I levels and lower performance on Gpeg were associated with white matter (WM) integrity loss in cortico-thalamic pathways and association fibres of frontal and temporal lobes. WM tracts involved in Gpeg performance overlapped with those affected by PAI-1. In conclusion, our findings suggest that the location of WM correlates of processing speed is largely consistent with those WM tracts affected by increased levels of markers of thrombosis. This may provide the specific neuroanatomical basis underlying this association.

Keywords: Other Functions/Disorders, Stroke, Neuropsychological Assessment and Psychometrics

Correspondence address: Mrs PhD Julia Miralbell, University of Barcelona, Psychiatry and Clinical Psychobiology, Pg. Vall d\, D-08300 Barcelona, Spain. Tel.: +34 666118649; E-mail: jmiralbell@ub.edu

**P3.49**

**Facial emotion recognition capacity in mild Alzheimer's disease: Labelling or perceptive dysfunction?**

Noemí Cerulla[1], Glòria Chico[1], Maite Garolera[2], Cristina Borras[1], Mariona Rodriguez-Querol[1], Esther Tomas[1] and Carme Civit[1]
[1]*Consorci Sanitari de Terrassa, Sant Jordi Day Hospital, Terrassa, Spain*
[2]*Consorci Sanitari de Terrassa, Hospital de Terrassa, Spain*

Little is known about the underlying processes involved in facial emotion recognition in Alzheimer's disease (AD). Two major hypotheses have been studied: 1) the impairment is associated to a semantic memory deficit and 2) the impairment is related to deficit in visual perception. The aim of this research was to study the relationship between the performance in facial emotion, recognition and memory and visual perception tasks in mild AD. 30 mild AD patients (19 women, 11 men; age mean 76 SD 5.3) and 21 healthy controls (13 women, 8 men; age mean 68 SD 6.2) were assessed with The Facial Expression of Emotion test (FEEST), Boston Naming Test (BNT), categorical fluency, figure copy and line orientation of the RBANS, Benton's visual form discrimination test, Poppelreuter figures and the Geriatric Depression Scale. We compared mean differences for two groups with independent sample t-test and estimated the effect size (Cohen's d). Pearson Correlation was used to measure relationship between cognitive results and bivariate partial correlation to control the effect of other variables. We set the alpha level to 0.05 to define statistical significance. Significant differences between groups (AD and controls) were found on all cognitive measures with a large and huge effect size, being the overall worse performance on AD. In AD, total FEEST was correlated with BNT [$r = 0.38$, $p = 0.038$], figure copy [$r = 0.449$, $p = 0.013$] and Poppelreuter [$r = 0.382$, $p = 0.037$]. After controlling for age and depression, total FEEST remained statistically significant correlated with BNT [$r = 0.391, p = 0.044$] and figure copy [$r = 0.445$, $p = 0.020$] In conclusion, these results suggest that both semantic memory and visual perception are involved in facial recognition impairment in AD.

Keywords: Social Cognition, Dementia, Neuropsychological Assessment and Psychometrics

Correspondence address: Mrs Noemí Cerulla, Consorci Sanitari de Terrassa, Sant Jordi Day Hospital, Edison, 15-37, s-08224 Terrassa, Spain. Tel.: +34 937336253; Fax: +34 937831555; E-mail: ncerulla@cst.cat

**P3.50**

**Differential involvement of right anterior temporal lobe in processing social concepts: Convergent TMS and neuropsychological evidence**

Gorana Pobric[1], Mathew Lambon Ralph[2] and Roland Zahn[2]
[1]*University of Manchester, Psychological Sciences, Manchester, United Kingdom*
[2]*University of Manchester*

Studies of semantic dementia, imaging and repetitive TMS have suggested that the bilateral anterior temporal lobes (ATLs) underpin a modality-invariant representational hub within the semantic system. However, questions remain regarding functional specialization across a variety of knowledge domains within the ATL

region. Specifically, it has been proposed that right superior anterior temporal cortex is crucial in processing of social concepts. The goal of this study was to provide causal evidence for the functional relevance of superior ATLs. In the rTMS study, participants made synonym judgments about social and nonsocial abstract words. Both social and abstract words were matched for psycholinguistic (frequency, imagibility and semantic diversity) and behavioural variables. We also examined performance on number magnitude judgment task as our control task. Offline rTMS was applied at 1 Hz for 10 min at 62% of machine output over right superior ATL (53, 8, −13), left superior ATL (−52, 8, −13) and occipital pole as a control site. After right ATL TMS stimulation, performance was selectively impaired for processing of social concepts, but not to equally demanding abstract concepts and nonsemantic cognitive tasks. In contrast, left ATL stimulation slowed down both social and nonsocial processing. In the neuropsychology part of the study, we used a semantic discrimination task which probed knowledge of social and animal function concepts in a patient with right frontotemporal dementia RFTD (rATL hypometabolism) and another patient with SD (lATL hypometabolism). Both patients were impaired on processing social and animal concepts compared to agematched controls. While the SD patient was equally impaired on processing both social and animal concepts, RFTD patient showed more pronounced impairments on social concepts than the SD patient. These convergent TMS and patient findings confirm that right ATL is part of a neural network supporting social conceptual knowledge.

Keywords: Social Cognition, Dementia, TMS/TCDS

Correspondence address: Dr Gorana Pobric, University of Manchester, Psychological Sciences, Oxford Road, M13-9PL Manchester, United Kingdom. E-mail: gorana.pobric@manchester.ac.uk

**P3.51**

**Fear perception and social behavioral problems in children with epilepsy: An explorative study.**

Maraike Coenen[1], Ceciel Aarnoudse[2], Oebo Brouwer[3] and Wencke Veenstra[2]
[1]*University of Groningen, Clinical and Developmental Neuropsychology, Groningen, Netherlands*
[2]*University Medical Center Groningen, Neurology/ Neuropsychology, Groningen, Netherlands*
[3]*University Medical Center Groningen, Neurology, Groningen, Netherlands*

Children with epilepsy often show emotional and behavioral problems, which are thought to be caused by impaired social competence (Nassau & Drotar, 1997). Golouboff et al. (2008) found that impaired recognition of fear is related to deviant behavior in children with temporal lobe epilepsy. However, in neuropsychological assessment of children with epilepsy, tests for emotion perception and social cognition are not commonly used. The aim of the present study is to investigate fear recognition in children with epilepsy in relation to social behavior, reported by their parents. 55 children with diagnosed epilepsy underwent neuropsychological assessment in the University Medical Center of Groningen between 2007 and 2013. In 18 of these children, social behavior was measured with the Child Behavior Checklist 6–18 (CBCL; Achenbach, 1991). For 7 children (3 boys, 4 girls, M = 10.34, SD = 2.42 range: 7.3–13.2 yrs) also a Dutch shortened version of the Facial Expression of Emotions – Stimuli and Tests (FEEST-36; Young et al. 2002) was used with an age matched healthy control group ($n$ = 64). Next to a variety of problems, 56% of the parents observe deviant social competences and social problems in their child. For all children at least one problem in social behavior and aggression is reported. Concerning fear recognition, children with epilepsy are less accurate than healthy controls. This difference is not significant but impaired fear recognition is significantly correlated with problems of social behavior, attention, thought and total behavioral problems. In an explorative study of children with epilepsy, parents reported social problems for all children, which were related to impaired fear recognition. Impaired fear perception can indicate problems understanding other people's intentions, which can lead to problems in interpersonal interactions.

Keywords: Social Cognition, Epilepsy, Neuropsychological Assessment and Psychometrics

Correspondence address: Mrs BSc Maraike Coenen, University of Groningen, Clinical and Developmental Neuropsychology, Grote Kruisstraat 2/1, NL-9712 TS Groningen, Netherlands. E-mail: m.a.coenen@umcg.nl

### P3.52

### Cerebellar role in social cognition

Anna Maria Tedesco[1], Silvia Clausi[1], Giusy Olivito[1], Marco Molinari[2] and Maria Leggio[1]
[1]*Ataxia Laboratory, Santa Lucia Foundation; Department of Psychology, "Sapienza" University of Rome, Rome, Italy*
[2]*Ataxia Laboratory, Santa Lucia Foundation, Rome, Italy*

It is well known that the cerebellar function by far exceed motor coordination, involving also modulation of cognitive and behavioural functions. Indeed, the cerebellum is part of a neural network including motor and associative regions. Moreover, findings of bidirectional pathways linking cerebellar and limbic structures have given rise to the suggestion that the cerebellum may play a role also in emotional processing. It is known that the limbic areas and specific portions of the frontal lobe are involved in Social Cognition. These findings have led to hypothesize a involvement of the cerebellum in this domain, but the studies carried out so far have not yet clarified whether and how the cerebellum is involved in specific aspects of social cognition.
Aim of the study was to investigate the cerebellar role in some aspects of social cognition. In particular recognition of social emotions and inference of mental states have been analysed in cerebellar patients.
A battery composed by 4 tasks has been administered: the "Reading the mind in the eyes test" and "Emotion attribution test" to assess the ability to recognize emotions; the "Theory of mind" task and the "Faux Pas" task to assess cognitive and affective components of the theory of mind.
This battery has been administered to cerebellar patients and healthy subjects well-matched for age and education. A T-test Student has been used to evaluate eventual differences between patients' and controls' performances.
Although the cerebellar damage impairs both the emotional recognition and the ability to attribute mental states, the patients showed significantly worse performance than the control group specifically in "Faux Pas" test.
These results indicate that the cerebellum plays a different weight in processing different social cognition components, and that it is particularly involved in the ability to attribute emotion, intentions and beliefs to other people in order to explain and predict their behaviour.

Keywords: Social Cognition, Neuropsychological Assessment and Psychometrics

Correspondence address: Dr Anna Maria Tedesco, Ataxia Laboratory, Santa Lucia Foundation, Department of Psychology, "Sapienza" University of Rome, via ardeatina 306; via dei Marsi 78, it-00179 Rome, Italy. Tel.: +39 0651501116; E-mail: tedescoannam@libero.it

### P3.53

### Oscillatory brain activity in reaction to object-directed and object-averted gazes in infants

Christine Michel[1], Manuela Stets[2], Eugenio Parise[3], Vincent M. Reid[4], Tricia Striano[5] and Stefanie Hoehl[6]
[1]*Heidelberg University, Heidelberg, Germany*
[2]*Department of Psychological & Brain Sciences, Indiana University, Bloomington, USA*
[3]*Central European University, Cognitive Development Center, Budapest, Hungary*
[4]*Department of Psychology, Lancaster University, Lancaster, UK*
[5]*Department of Psychology, Hunter College, New York, USA*
[6]*Department of Psychology, Heidelberg University, Heidelberg, Germany*

The direction of eye gaze is an important cue guiding attention and facilitating object processing already early in life.
In an ERP study with 4-month-old infants, pictures of a person looking at an object lead to greater positive slow wave (PSW) activity than pictures of a person looking away from an object. Together with previous results, this suggests that infants build stronger memory representations for gaze-cued objects compared to objects that are not looked at (Hoehl, Reid, Mooney, & Striano, 2008).
In adults, alpha oscillations were recently associated with access to a knowledge system in the human brain (Klimesch, 2012). Oscillations in the alpha range may thus be involved in infants' encoding of novel objects. Here, alpha oscillations in reaction to object-directed and object-averted eye gaze were investigated in 16 4-month-old infants.
Data from Hoehl et al. (2008) were reanalyzed with regard to oscillatory brain activity. Infants were presented photographs of a person either looking at or looking away from an object and EEG was recorded continuously. Pictures were presented for 1000 ms and alpha band oscillations time-locked to stimulus onset were analyzed. Mean amplitude of the frequency range 5–7 Hz, 400–600 ms after stimulus onset on fronto-central electrodes was used as the dependent variable. Desynchronization of alpha band activity in comparison to the baseline was found for object-directed gaze (t(15) = $-2.2$, $p = 0.042$), and no changes in alpha band activity were found for object-averted gaze (t(15) = $-0.8$, $p = 0.497$) in 4-month-old infants. Additionally, data of 2- 6- and 9-month-olds that are currently being analyzed will be presented.
Alpha desynchronization in infants may reflect processing of the relation between eye gaze direction and related objects. Similar to the PSW, alpha desynchronization might be a neural correlate of forming memory representations in infants, possibly through access to a knowledge system as described in adults.

Keywords: Social Cognition, Other Disorder/Samples, Electrophysiology (EEG/ERP)

Correspondence address: Mrs Christine Michel, Heidelberg University, Hauptstraße 47-51, D-69117 Heidelberg, Germany. E-mail: Christine.Michel@psychologie.uni-heidelberg.de

**P3.54**

**Selective theory of mind impairment and cerebellar atrophy: A case report**

Annalisa Parente, Valentina Manfredi, Anna Tarallo, Ettore Salsano, Alessandra Erbetta, Davide Pareyson and Anna Rita Giovagnoli
*Fondazione IRCCS Istituto Neurologico C. Besta, Department of Diagnostics and Applied Technology, Milan, Italy*

INTRODUCTION: The cerebellum is important to the modulation of affect and behavior, but there is scarce knowledge of the cognitive pattern underlying psychobehavioral changes in cerebellar syndrome.
GOALS OF THE STUDY: This study describes a selective association between social cognitive impairment and cerebellar atrophy in an 65 years old adult, helping clarify the role played by the cerebellum in cognitive disorders.
METHODS: The patient underwent detailed neuropsychological testing for executive functions, language and memory.
As for social cognition, the Faux Pas (FPT), Strange Stories, and Reading the Eyes in the Mind (REMT), the Emotion Attribution Task (EAT), and the Social Situation (SST) and Moral/Conventional Distinction tasks (MCDT) were administered.
The Beck Depression Inventory (BDI), State Trait Anxiety Inventory (STAI), and Symptom Check List-90 (SCL-90) assessed depression, anxiety, and emotional-behavioral distress. The Multiple Ability Self-report Questionnaire (MASQ) explored perceived functioning. The Lubben's scale assessed the number and efficacy of social relationships.
RESULTS: Neuropsychological examination evidenced significant deficits in theory of mind (ToM), a component of social cognition and verbal fluency, with preservation of all of the other functions. The inventories yielded normal scores in mood and behavior.
CONCLUSIONS: The results demonstrate that acquired cerebellar damage can impair ToM, highlighting a role for the cerebellum in the modulation of social cognition. This suggests a redefinition of the affective-cognitive cerebellar syndrome.

Keywords: Social Cognition, Other Disorder/Samples, Neuropsychological Assessment and Psychometrics

Correspondence address: Mrs Annalisa Parente, Fondazione IRCCS Istituto Neurologico C. Besta, Department of Diagnostics and Applied Technology, Via Celoria, 11, I-20133 Milan, Italy. E-mail: annalisa.parente@istituto-besta.it

**P3.55**

**Cognitive biases and neurocognition as independent domains in first-episode schizophrenia patients**

Marc Turon[1], Sol Fernandez-Gonzalo[2], Esther Pousa[3], Diego Palao[3] and Mercè Jodar[4]
[1]*Parc Taulí Hospital, Research Department, Sabadell, Spain*
[2]*Parc Tauli Hospital, Research Department, Sabadell, Spain*
[3]*Parc Tauli Hospital, Mental Health Department, Sabadell, Spain*
[4]*Parc Tauli Hospital, Neurology Department, Sabadell, Spain*

Individuals with schizophrenia present specific cognitive biases that may play a role in the development and exacerbation of symptoms and may impair overall functioning. In chronic schizophrenia samples, studies about the overlapping between these cognitive biases with the neuropsychological functions have shown contradictory results. Therefore, in order to shed light on the underpinnings of these biases, the present study aims at exploring the relationship between the most commonly studied cognitive biases (Jumping to Conclusions, JTC, and Bias Against Disconfirmatory Evidence, BADE) and the main deficits related to schizophrenia (executive functions, attention and memory) in a first-episode remitted sample. Thirty-six first-episode schizophrenia patients were evaluated by a comprehensive battery of neuropsychological and cognitive biases measures. A Principal Component Analysis was performed to determine the pattern of relationships among cognitive biases (JTC and BADE) and neuropsychological measures (abstraction, phonetic fluency, attention and verbal memory). The Principal Component Analysis with Varimax Rotation resulted in a three component solution that account for 76% of the total variance. The first component was dominated by high loadings on verbal memory and phonetic fluency. Abstraction and attention loaded in the second component, whereas JTC and BADE loaded in a third component, independent to other neuropsychological constructs. In our first-episode schizophrenia sample, the principal component analysis showed the independence of cognitive biases compared to other neuropsychological functions. This suggests that these cognitive biases could have a neuroanatomical substrate independent of other neurocognitive functions, and seem to result from thinking distortions and unconscious processing preferences rather than impairment in neuropsychological functions.

Funded by Fundació la Marató de TV3

Keywords: Social Cognition, Psychopathology, Neuropsychological Assessment and Psychometrics

Correspondence address: Mr MD Marc Turon, Parc Taulí Hospital, Research Department, Parc Tauli 1, D-08208 Sabadell, Spain. E-mail: mturon@tauli.cat

### P3.56

### A new social and non-social cognitive rehabilitation computarized program for schizophrenia/schizoaffective disorders in early stages: preliminary efficacy findings

Sol Fernandez-Gonzalo[1], Marc Turon[1], Esther Pousa[2], Diego Palao[2] and Mercè Jodar[3]
[1]*Parc Tauli Hospital, Research Department, Sabadell, Spain*
[2]*Parc Tauli Hospital, Mental Health Department, Sabadell, Spain*
[3]*Parc Tauli Hospital, Neurology Department, Sabadell, Spain*

People with schizophrenia and schizoaffective disorders present neuropsychological deficits in almost every cognitive domain. These deficits are observed before illness onset and they present a significant decline on the first episode. Neuropsychological deficits are linked with the daily-living functioning, but recently, authors have demonstrated that social cognitive impairments have a greater impact in functional outcomes. Cognitive rehabilitation has demonstrated consistent effects improving cognitive performance as well as ameliorating symptoms and psychosocial functioning. However, the active key of these interventions for generalization remains unknown. The aim of this study is to present the design and preliminary efficacy results of a new social and non-social cognitive computerized program for people with schizophrenia/schizoaffective disorder in early stages of the illness.
16 patients that met inclusion criteria were randomized between treatment and control groups. A comprehensive assessment of social and non-social neuropsychological variables was carried out before and after treatment.
Mann-Whitney test showed significant differences between groups in Digits Span Backward ($p < 0.01$), Logical Memory Learning Curve ($p < 0.01$), Execution Time of the Tower of London ($p = 0.04$), 2nd Order ToM Task ($p = 0.03$) and Bias Against Disconfirmatory Evidence ($p = 0.04$)
A tendency to signification was observed in Digits Span Forwards ($p = 0.07$), Spatial Span Backwards ($p = 0.08$) and Violation Time of Tower of London ($p = 0.07$)
This new intervention showed preliminary efficacy results for logical learning, verbal working memory, processing speed, theory of mind and cognitive biases as well as a tendency to improve attention and visual working memory. Social and non-social cognitive rehabilitation in first-episode schizophrenia patients could be a relevant clinical tool improving cognitive alterations and even preventing functional impairment.

Funded by Fundació la Marató TV3

Keywords: Social Cognition, Psychopathology, Neuropsychological Assessment and Psychometrics

Correspondence address: Mrs MD Sol Fernandez-Gonzalo, Parc Tauli Hospital, Research Department, Parc Tauli 1, D-08208 Sabadell, Spain. E-mail: msfernandez@tauli.cat

**P3.57**

**Long-term follow-up after extensive bilateral frontal lesions: A case study**

Roxane Pizzo, Thierry Deonna, Véronique Davidoff, Anne.Lise Ziegler, Eliane Roulet and Claire Mayor Dubois
*CHUV – Pediatric, Lausanne, Switzerland*

There are only a few longitudinal studies on children who suffered from a severe TBI in early childhood, and the cognitive and behavioral long-term impact of early extensive bilateral frontal lesions remains scarce. Furthermore, controversial data are reported, especially regarding executive functions and moral, social behavior. We report a prospective longitudinal study of a boy presenting with an early severe TBI at 14 months old resulting in extensive bilateral frontal lesions. The 9 successive assessments from 27 months to 21 years old included 1) the general intellectual development (developmental and intelligence scales) 2) the executive functions (inhibition, shifting, initiation, planning) and 3) socio-cognitive skills (behavior questionnaires, theory of mind, social reasoning, and gambling task). Results showed a borderline intelligence up to 7 years old whereas scores situated in the mild mental retardation range were noted from 8 years old. Executive impairment also appeared at 8 years old and increased with time, with deficits on all executive domains. There were no social, moral or behavioral disorders at any moment, but deficits in social reasoning, TOM and gambling task were observed. Mild intellectual retardation, with stable intellectual quotients over childhood and adolescence was also reported by other authors. In our case, it is worth noting that the intellectual development slowed down at the moment in which more complex, abstract analogical reasoning should appear, between 8 and 12 years old. Executive impairments also appeared at that time. Damage to the frontal lobes would thus critically impair the development of abstract reasoning and executive functions expected to develop at that age. Finally, contrary to other reports, our patient exhibited no behavior disorder. We observed however dissociated skills, with preserved everyday life's behavior and poor socio-cognitive knowledge, suggesting separate socio-cognitive networks.

Keywords: Social Cognition, Traumatic Brain Injuries, Neuropsychological Assessment and Psychometrics

Correspondence address: Dr Roxane Pizzo, CHUV – Pediatric, Bugnon 46, CH-1011 Lausanne, Switzerland. E-mail: roxane.pizzo@chuv.ch

**P3.58**

**Neural underpinnings of impaired predictive motor timing in children with Developmental Coordination Disorder**

Julie Debrabant[1], Freja Gheysen[2], Karen Caeyenberghs[3], Hilde Van Waelvelde[4] and Guy Vingerhoets[2]
[1]*Ghent University, Rehabilitation siences and physiotherapy, Ghent, Belgium*
[2]*Ghent University, Experimental Psychology, Ghent, Belgium*
[3]*Ghent University, Rehabilitation Sciences and Physiotherapy, Movement and Sports Sciences, Ghent, Belgium*
[4]*Ghent University, Rehabilitation Sciences and Physiotherapy, Ghent, Belgium*

Developmental Coordination Disorder (DCD) is a clinical label that describes an impaired acquisition and clumsy approach of motor activities including ball skills, writing, and sports. A dysfunction in predictive motor timing is put forward to underlie DCD-related motor problems. Predictive timing allows for the preselection of motor programs in order to decrease processing load and facilitate reactions. Using functional magnetic resonance imaging (fMRI), this study investigated the neural correlates of motor timing in DCD ($n = 17$, mean age 9.4 $\pm$ 0.6 years) and typically developing children ($n = 17$, mean age 9.2 $\pm$ 0.9 years).

The task involved motor responses to sequences of visual stimuli with predictive or unpredictive interstimulus intervals (ISIs). DCD children responded with a smaller reaction time (RT) advantage to predictive ISIs compared to typically developing children. Typically developing children exhibited higher activation in the right dorsolateral prefrontal cortex (DLPFC) and right inferior frontal gyrus (IFG) for responses at unpredictive as opposed to predictive ISIs, whereas activations in DCD children were non-differentiable. Moreover, DCD children showed less activation than typically developing children in the right DLPFC, the left posterior cerebellum (crus I) and the right temporo-parietal junction (TPJ) for this contrast. Notably, activation in the right temporo-parietal junction (TPJ) positively correlated with RT as an indicator of processing load in both groups. These data indicate that motor performance in DCD children requires extra processing demands due to impaired predictive encoding.

Keywords: Other Functions/Disorders, Other Disorder/Samples, Functional and Structural Neuroimaging/NIRS
Correspondence address: MS Julie Debrabant, Ghent University, Rehabilitation siences and physiotherapy, Campus Heymans (UZ) De Pintelaan 185, B-9000 Ghent, Belgium. Tel.: +32 478395799; E-mail: julie.debrabant@ugent.be

### P3.59

### Dissociable neural systems in implicit motor imagery tasks

Paola Invernizzi[1], Laura Zapparoli[1], Martina Gandola[2], Antonio De Santis[3], Alberto Zerbi[3], Giuseppe Banfi[3] and Eraldo Paulesu[4]
[1]*University of Milano-Bicocca, Psychology, Milan, Italy*
[2]*University of Pavia, Brain and Behavioral Sciences, Pavia, Italy*
[3]*IRCCS Galeazzi, Milan, Italy*
[4]*University of Milano-Bicocca, Pyshcology, Milan, Italy*

Motor imagery (M.I.), the active cognitive process during which movements are mentally rehearsed without overt actions, is a useful paradigm for the study of different aspects of motor control. A variety of tasks have been used in behavioural and neurofunctional experiments. M.I. tasks may be explicit in that subjects are asked to imagine themselves while performing a given action; conversely, in implicit M.I. tasks, subjects are involved in decisions that imply the mental simulation of an action. The implicit M.I. tasks most frequently used are the Hand Laterality Task (HLT: decisions are made on whether a hand portrayed in a picture is a left or a right one) and the Grip Selection Task (GST: participants indicate the most natural grip for grasping an object).

Here we tested the hypothesis that these two implicit M.I. tasks, normally treated as equivalent, are subserved by partially dissociable brain systems. We compared the brain patterns associated with HLT and GST in 13 healthy subjects (mean age: 26 years) in a event-related fMRI experiment.

We found that, beyond a common fronto-parietal network typically described in M.I. studies, there were task specific brain activations: the HLT showed a wider recruitment of occipital and sensorimotor regions (especially in the right hemisphere); the GST was specifically associated with the left fusiform gyrus in a region previously associated with tool processing. Correlations between fMRI and RT data confirmed these dissociations suggesting a direct link with the mental manipulation time rather than earlier object identification.

Our results show the complexity of M.I.: motor simulation relies on motor specific brain system and domain specific systems that can be dissociated depending on the task in hand. In addition, we support and provide anatomical specificity to previous observations (1) on brain damaged patients that suggested a double hemispheric dissociation for these tasks.

Reference
[1] Daprati et al (2010).

Keywords: Other Functions/Disorders, Other Disorder/Samples, Functional and Structural Neuroimaging/NIRS

Correspondence address: Mrs Paola Invernizzi, University of Milano-Bicocca, Psychology, Piazza dell' Ateneo Nuovo, 20126 Milan, Italy. E-mail: paolainv81@gmail.com

**P3.60**

**The physiology of motor delusions: Functional anatomical patterns in anosognosia for hemiplegia**

Martina Gandola[1], Paola Invernizzi[2], Laura Zapparoli[2], Margherita Verardi[2], Roberto Sterzi[3], Ignazio Santilli[3], Maurizio Sberna[4], Gabriella Bottini[1] and Eraldo Paulesu[2]
[1]*Department of Brain and Behavioral Sciences, University of Pavia, Pavia, Italy*
[2]*Department of Psychology, University of Milano-Bicocca, Milano, Italy*
[3]*Neurology Department, Niguarda Ca' Granda Hospital, Milano, Italy*
[4]*Neuroradiology Department, Niguarda Ca' Granda Hospital, Milano, Italy*

Patients with right brain damage might not complain about left-sided paralysis, or they could deny that the left side is paralysed or even claim that they have just moved a paralysed limb. This condition is known as anosognosia for hemiplegia (Babinski, 1914). The denial of the motor deficit has been explained on the grounds that a focal lesion within the motor/premotor system reduces intentional motor behaviour. If one has no intention to move, he/she would not notice any discrepancy between the missing intention and the consequent lack of motion (Heilman, 1991; 1998). However, it remains unclear how patients can claim that they have moved the paralysed limb unless one postulates that there is a residual and perhaps misinterpreted motor brain activity (Frith et al., 2000). These are the alternative hypotheses of two well-known models of motor control in anosognosia. Behavioural experiments suggest that some residual intentionality might be present in patients with anosognosia (Berti et al., 2007; Fotopoulou et al., 2008; Garbarini et al., 2012; Pia et al., 2012). Here, we used functional magnetic resonance imaging (fMRI) to study patients with and without anosognosia for hemiplegia during the execution of a simple movement task. We provide the missing direct neuroimaging evidence that the delusional belief of having moved is preceded by brain activation of cortical regions that are implicated in motor control in the intact hemisphere and in spared motor regions of the right hemisphere; patients with hemiplegia and without anosognosia did not present with the same degree of activation of the cortical motor system. We conclude that the false belief of movement in patients with hemiplegia is associated with a combination of strategically placed brain lesions and the preceding residual neural activity of the fronto-parietal motor network. These findings provide evidence that the activity of motor cortices contributes to our beliefs about the status of our motor system.

Keywords: Other Functions/Disorders, Stroke, Functional and Structural Neuroimaging/NIRS

Correspondence address: Mrs Martina Gandola, University of Pavia, Department of Brain and Behavioral Sciences, Piazza Botta 11, IT-27100 Pavia, Italy. E-mail: martina.gandola@unipv.it

**P3.61**

**Visual aids reduce framing effects in older adults**

Laura Zamarian, Angelika Kuprian, Veronika Prantner and Margarete Delazer
*Medical University Innsbruck, Clinical Department of Neurology, Innsbruck, Austria*

BACKGROUND: Patients as well as healthy persons need to be adequately informed to actively participate in medical care and make informed decisions. Framing effects influence how people interpret information and make choices. Typically, people show a more favorable attitude towards positively framed treatments (70% chance of survival) than towards negatively framed treatments (30% chance of mortality). In this study, we investigated (a) whether positively framed medications are rated more favorably than negatively framed medications, (b) whether this effect can be countered by using different types of visual aids (icon arrays vs. pies), and (c) whether age modulates framing effects and the efficacy of visual aids.
METHOD: Thirty young-old adults (age range 51–70 years) and 30 old-old adults (age range 71–89 years) rated medications on a color-based, 7-point scale from negative (red) to positive (green). Medications were described either in positive terms (e.g., 84% of the cases taking medication XY showed improvement) or in negative terms (e.g., 16% of the cases showed worsening). Framing effects were defined by the difference in ratings between positively framed conditions and negatively framed conditions. Participants rated first medications without any visual aid, then medications where either an icon array or a pie chart was added to numerical information.
RESULTS: Overall, there were no significant effects of age. Participants showed significant framing effects when no visual aid was presented. The use of visual aids dramatically reduced framing effects. However, pie charts were more effective than icon arrays in decreasing the effect of framing.
CONCLUSIONS: Visual aids are a promising method to reduce framing effects and help older people to understand medical information and make informed decisions. [Acknowledgements: TWF-2010-1-993.]

Keywords: Decision making, Aging, Neuropsychological Assessment and Psychometrics

Correspondence address: Mrs Dr. Laura Zamarian, Medical University Innsbruck, Clinical Department of Neurology, Anichstr. 35, AT-6020 Innsbruck, Austria. E-mail: laura.zamarian@gmail.com

**P3.62**

**Exploring the fronto-striatal contributions to economic decision-making**

Claire O'Callaghan[1], S.J.G. Lewis[2], Maxime Bertoux[3], J.R. Hodges[1] and Michael Hornberger[1]
[1]*Neuroscience Research Australia, Sydney, Australia*
[2]*Brain and Mind Research Institute, Sydney, Australia*
[3]*University Pierre and Marie Curie, Paris VI, Sorbonne Universités, Paris, France*

Impulsive decision-making is a hallmark feature of behavioural variant frontotemporal dementia (bvFTD) and is presumed to relate to pervasive ventromedial prefrontal degeneration in these patients; however, objective tests of decision-making have not been extensively employed in this population. In contrast, Parkinson's disease (PD) patients have been shown to demonstrate impulsive decision-making related to striatal dysfunction. In the current study, we contrast these two disease groups using an economic decision-making measure to assess for impulsive decision-making behaviour. Ten bvFTD patients, eighteen PD patients and fourteen age-matched Controls were administered a delay discounting task, which required them to choose between varying amounts of money that were either immediately available or available at a certain point in the future. Individual discounting rate parameters (k rates) were calculated, with larger k values corresponding to more impulsive choice. Results showed a significant overall group difference, with bvFTD patients having higher k values than both Controls and PD patients; however, the k values of Controls and PD patients were equivalent. Interestingly, these group differences were only found when the choices involved medium to large amounts of money, suggesting that bvFTD patients do not show the typical 'magnitude effect', whereby discount rates decrease with increasing magnitude of reward. Overall, these findings suggest that bvFTD patients demonstrate impulsive decision-making on a delay discounting economic decision-making task regardless of the reward magnitude employed, which corroborates the clinical symptoms of the patients. By contrast, PD patients were not impaired on the delay discounting task, which suggests that this task is likely mediated predominately by ventromedial prefrontal dysfunction in bvFTD.

Keywords: Decision making, Dementia, Other Methods

Correspondence address: Mrs Claire O'Callaghan, Neuroscience Research Australia, Barker St, 2047 Sydney, Australia. E-mail: c.ocallaghan@neura.edu.au

**P3.63**

**Decision making and numeracy – avoiding risks or taking chances?**

Margarete Delazer[1], Ida Gradl[2], Nina Blume[2], Marie-Theres Pertl[2], Johannes Schiebener[3] and Matthias Brand[3]
[1]*Medizinische Universität Innsbruck, Klinik für Neurologie, Innsbruck, Austria*
[2]*Leopold Franzens Universität, Innsbruck*
[3]*Universität Duisburg-Essen, Duisburg*

Though a detrimental effect of low numeracy on decision making has been described, the exact relation between the two domains has not been investigated so far. The present study aims at estimating the effect of numerical abilities, executive functions and sensation seeking on decision making under risk. We used the Game of Dice Task Double which assesses decision making in two steps – first, selecting an alternative associated with different winning probabilities, second, betting on the chosen alternative (doubling the possible gains/losses or not). This task allows us to distinguish between advantageous, but risk avoiding behavior and optimal behavior with a higher bet. 120 healthy participants aged between 18 and 65 took part in the study and performed tests of decision making, mental calculation, health numeracy, subjective numeracy, verbal working memory, numerical problem solving, psychomotor speed, set-shifting and sensation seeking. Results of the decision making task suggest that participants are able to identify non-risky alternatives (79.38%) and often select the most advantageous alternative (46.06%) in the first step. However, they do not always optimize their choices in the second step and choose the best strategy in only 31.27% of all decisions. Good cognitive capacities (numerical abilities, executive functions) are associated with advantageous and conservative decisions. Regression analysis shows that performance in the numeracy task predicts avoidance of risky, disadvantageous choices, but not choosing the best option when this option is associated with a higher bet. In conclusion, advantageous decision making of cognitively well performing individuals seems to be grounded in rather conservative, risk avoiding behaviour. Good numerical abilities predict avoidance of disadvantageous risky choices. Future studies may apply the Game of Dice Task Double in order to understand the specific difficulties of cognitively impaired patients.

Keywords: Decision making, Other Disorder/Samples, Neuropsychological Assessment and Psychometrics

Correspondence address: Mrs Prof. Margarete Delazer, Medizinische Universität Innsbruck, Klinik für Neurologie, Anichstrasse 35, A-6020 Innsbruck, Austria. Tel.: +43 0512 504 23870; E-mail: margarete.delazer@i-med.ac.at

**P3.64**

**Electrophysiological correlates of probabilistic reversal learning in obsessive-compulsive disorder**

Marie Klippel[1], Tanja Endraß[2], Anja Riesel[1], Julia Gotthart[1] and Norbert Kathmann[1]
[1]*Department of Psychology, Humboldt Universität zu Berlin, Berlin, Germany*
[2]*Department of Psychology, Otto-von-Guericke-Universität Magdeburg, II, Magdeburg, Germany*

Obsessive-compulsive disorder (OCD) is associated with alterations in frontostriatal loops, involving the orbitofrontal cortex (OFC) and the anterior cingulated cortex (ACC). These brain regions play a specific role in reversal processing. In fMRI investigations of probabilistic reversal learning OCD patients repeatedly showed reduced activity in the OFC and other areas. Therefore reversal learning seems a promising target when investigating neuropsychological functions in OCD. The aim of this study was to gain better understanding of reversal learning in OCD by investigating electrophysiological correlates of feedback processing (the feedback related negativity, generated in the ACC). OCD patients and matched healthy controls were examined with a modified probabilistic reversal learning task. Subjects learned by feedback which one of two geometrical stimuli was correct. After the choice of one stimulus was rewarded for an unpredictable number of trials, this stimulus choice suddenly received negative feedback, either attributable to a stable change of reward contingencies or to a single invalid negative feedback. Probabilities of contingency changes and invalid feedbacks were equal and therefore feedback validity was unpredictable. Behavioral performance did not differ between OCD patients and healthy controls. However, in OCD patients, the feedback-related negativity was less modulated by feedback valence than in controls, when they had stayed with the previously selected stimulus after an initial negative feedback. This is interpreted as reduced sensitivity to external feedback in trials with perseverative responding. In conclusion, feedback processing during reversal learning appears to be affected not in general but only under specific circumstances.

Keywords: Decision making, Psychopathology, Electrophysiology (EEG/ERP)

Correspondence address: Mrs Marie Klippel, Department of Psychology, Humboldt Universität zu Berlin, Rudower Chaussee 18, D-12489 Berlin, Germany. E-mail: marieklippel@gmail.com

**P3.65**

**Neuropsychological profile in asymptomatic children with Sickle Cell Disease**

Sophie Déglise[1], Roxane Pizzo[2], Valérie Leclair Rezbach[3], Cécile Jérôme Choudja[3], Maja Beck Popovic[4], Eliane Roulet Perez[2] and Claire Mayor Dubois[2]
[1]*CHUV, Pediatric Department, Lausanne, Switerland*
[2]*CHUV, Pediatric Departement, 1011 Lausanne, Switzerland*
[3]*CHUV, Pediatric Hematology Oncology Unit, 1011 Lausanne, Switzerland*
[4]*CHUV, Pediatric Hematology Oncology Departement, 1011 Lausanne, Switzerland*

Over the past years, studies on children with Sickle Cell Disease (SCD) have well documented the cognitive impairments due to cerebral vascular injury (overt strokes and silent infarcts). However, less is known about the cognitive profile of children without cerebral damage and we therefore planned to investigate neurologically asymptomatic paediatric patients with SCD.
Out of a cohort of 14 patients, we included eight children (aged 5 to 13 years, mean = 7.8, 4 girls and 4 boys) with HbSS genotype, living since their birth in Switzerland. We excluded children with mental retardation (IAG<70, General Aptitude Index) and those with infarct and with abnormal/conditional Transcranial Doppler Ultrasonography. Our assessment included standard tests targeting general cognitive functioning (Wechsler Intelligence scale), attention skills (CPT-II), memory (CMS word-list, Rey Complex Figure Test, Rey 15 signs-list), executive functions (Stroop, Mazes, Color Trail, verbal and figural Fluency, multiple classification tests), academic achievement (BREV, ODEDYS), sensory motor skills (NEPSY, Purdue Pegboard).
Cognitive results showed average to borderline quotients (PRI and VCI = average, PSI = low average, WMI = borderline). Seven out of the eight children exhibited attention deficits, half of them had working memory impairments, all but one showed a significant delay in their reading skills and all of them had poor visuo-motor coordination. Moreover, we found correlations between the hematocrit values and the attentional skills, verbal working memory and manual dexterity. Noteworthy, a few other studies also reported relationships between the IQ and hematocrit values.
Our results showed thus that even asymptomatic children with SCD are vulnerable to cognitive deficits and are at risk for academic difficulties. Reduction in the oxygen-carrying capacity of red-blood cell may thus be responsible for such neuropsychological deficits irrespective of brain infarction.

Keywords: Other Functions/Disorders, Other Disorder /Samples, Neuropsychological Assessment and Psychometrics

Correspondence address: Mrs Sophie Déglise, CHUV, Pediatric Department, Rue Bugnon, CH-1011 Lausanne, Switerland. Tel.: +41 213143682; E-mail: sophie.deglise@chuv.ch

### P3.66

### Psychometric Problems and possible solutions for the classification of mild cognitive impairment

N. Bucholtz[1], G. Lämmler[1], E. Steinhagen-Thiessen[1] and M. Niedeggen[2]
[1]*Charité, Research Group on Geriatrics, Berlin, Germany*
[2]*Freie Universität Berlin, Department of Education and Psychology*

The consideration of Mild Cognitive Impairment (MCI) as a prodromal stage of dementia has become one main focus in neuropsychological research. However, MCI is a heterogeneous syndrome and has been described distinctly in various concepts. To unify these definitions Winblad et al. (2004) have introduced a model that contains different aspects of MCI. They distinguish a) amnestic and non-amnestic subtypes and b) if cognitive capacity is impaired in one or multiple domains. Even so, there is no consensus in how to operationalize MCI. Studies still consider different cognitive abilities and use different test parameters to assess these domains: For instance, problems of intercorrelations and underlying latent factors of test parameters have not been addressed. Therefore, the aim of this study is a comprehensive psychometric analysis of the widely used test battery of the Consortium to Establish a Registry for Alzheimer's Disease (CERAD Plus). N = 1014 participants at the age of 60 years and older of the Berlin Aging Study II were assessed using the CERAD Plus battery consisting of 11 subtests (semantic word fluency, Boston Naming Test, word list learning, word list recall, word list recognition, word list intrusions, constructional praxis, constructional recall, phonemic fluency, Trail Making Test A, Trail Making Test B). Psychometric properties of the test battery were examined using exploratory (EFA) and confirmatory factor analysis (CFA). Preliminary results of an EFA reveal best fit for a four factor solution ($\chi^2$ = 9.176, *df* = 6, $p$ = 0.164, *CFI* = 0.998, *RMSEA* = 0.023). A model with four correlated factors could be confirmed in CFA ($\chi^2$ = 92.009, *df* = 36, $p < 0.01$, *CFI* = 0.968, *RMSEA* = 0.039). Factors can be interpreted as verbal memory, contextual praxis, verbal abilities and executive function. Based on these results we report prevalence rates compared to other studies and discuss implications for MCI classification.

Correspondence address: Mrs Nina Bucholtz, Charité, Research Group on Geriatrics, Berlin, Germany. E-mail: nina.bucholtz @charite.de

**P3.67**

**Ageing-related changes in relational and conjunctive working-memory binding**

Roy Kessels[1], Bonnie Van Geldorp[2] and Mario Parra[3]
[1]*Radboud University Nijmegen, Neuropsychology and Rehabilitation Psychology, Nijmegen, Netherlands*
[2]*Radboud University Nijmegen, The Netherlands*
[3]*University of Edinburgh, UK*

The ability to form associations (i.e. binding) is critical for memory formation. Recent studies suggest that ageing specifically affects some types of binding: relational binding (associating separate features, for example object and location) declines with advancing age, whereas conjunctive binding (integrating features within an object) is insensitive to ageing. One possible explanation is that functional integrity of the hippocampus is required for relational binding, but not for conjunctive binding. However, this hippocampal involvement may be explained by the use of spatial information in most studies. Alternatively, relational binding may simply require more attentional resources than conjunctive binding.
Both types of binding were examined in a working memory (WM) task using the same (non-spatial) features: shape and color. We used an articulatory suppression task during encoding and maintenance to study the effect of attentional resources. Thirty-one young adults (mean age 22.4), 30 middle-aged adults (mean age 54.8) and 30 older adults (mean age 70.3) performed the task. We expected that ageing would affect relational binding more than conjunctive binding. If attentional resources are the mediating factor, relational binding would be more affected by the interfering task than conjunctive binding. Results show an effect of age group F(2,88) = 14.7, $p < 0.001$) and type of binding (F(1,88) = 16.5, $p < 0.01$), but no interaction between type of binding and age (F(2,88) = 1.4, $p = 0.25$). The interaction between type of binding and interference was significant (F(1,88) = 4.71, $p = 0.03$). This suggests that relational binding requires more attentional resources than conjunctive binding. We hypothesize that a general decline in WM resources associated with frontal dysfunction underlies deficits in WM binding.

Keywords: Memory and Learning, Aging, Neuropsychological Assessment and Psychometrics

Correspondence address: Prof. Dr. Roy Kessels, Radboud University Nijmegen, Neuropsychology and Rehabilitation Psychology, Montessorilaan 3, NL-6533 ZR Nijmegen, Netherlands. E-mail: r.kessels@ donders.ru.nl
